# Non-malarial febrile illness: a systematic review of published aetiological studies and case reports from Africa, 1980–2015

**DOI:** 10.1186/s12916-020-01744-1

**Published:** 2020-09-21

**Authors:** Jeanne Elven, Prabin Dahal, Elizabeth A. Ashley, Nigel V. Thomas, Poojan Shrestha, Kasia Stepniewska, John A. Crump, Paul N. Newton, David Bell, Hugh Reyburn, Heidi Hopkins, Philippe J. Guérin

**Affiliations:** 1 Infectious Diseases Data Observatory, University of Oxford, New Richards Building,Old Road Campus,Headington, Oxford, OX3 7LG UK; 2 Centre for Tropical Medicine and Global Health, Nuffield Department of Clinical Medicine, University of Oxford, Oxford, UK; 3 Lao-Oxford-Mahosot Hospital-Wellcome Research Unit, Mahosot Hospital, Vientiane, Laos; 4 Centre for International Health, University of Otago, Dunedin, New Zealand; 5 London School of Hygiene and Tropical Medicine, London, WC1E 7HT UK; 6 Independent consultant, Issaquah, Washington USA

**Keywords:** Fever, Febrile illness, Malaria, Non-malarial febrile illness, Microbiology, Africa, Aetiology, Diagnosis

## Abstract

**Background:**

The availability of reliable point-of-care tests for malaria has heralded a paradigm shift in the management of febrile illnesses away from presumptive antimalarial therapy. In the absence of a definitive diagnosis, health care providers are more likely to prescribe empirical antimicrobials to those who test negative for malaria. To improve management and guide further test development, better understanding is needed of the true causative agents and their geographic variability.

**Methods:**

A systematic review of published literature was undertaken to characterise the spectrum of pathogens causing non-malaria febrile illness in Africa (1980–2015). Literature searches were conducted in English and French languages in six databases: MEDLINE, EMBASE, Global Health (CABI), WHO Global Health Library, PASCAL, and Bulletin de la Société Française de Parasitologie (BDSP). Selection criteria included reporting on an infection or infections with a confirmed diagnosis, defined as pathogens detected in or cultured from samples from normally sterile sites, or serological evidence of current or past infection. A number of published articles (rather than incidence or prevalence) reporting a given pathogen were presented.

**Results:**

A total of 16,523 records from 48 African countries were screened, of which 1065 (6.4%) met selection criteria. Bacterial infections were reported in 564 (53.0%) records, viral infections in 374 (35.1%), parasitic infections in 47 (4.4%), fungal infections in nine (0.8%), and 71 (6.7%) publications reported more than one pathogen group. Age range of the study population was not specified in 233 (21.9%) publications. *Staphylococcus aureus* (18.2%), non-typhoidal *Salmonella* (17.3%), and *Escherichia coli* (15.4%) were the commonly reported bacterial infections whereas Rift Valley fever virus (7.4%), yellow fever virus (7.0%), and Ebola virus (6.7%) were the most commonly reported viral infections. Dengue virus infection, previously not thought to be widespread in Africa, was reported in 54 (5.1%) of articles.

**Conclusions:**

This review summarises the published reports of non-malaria pathogens that may cause febrile illness in Africa. As the threat of antimicrobial resistance looms, knowledge of the distribution of infectious agents causing fever should facilitate priority setting in the development of new diagnostic tools and improved antimicrobial stewardship.

**Trial registration:**

PROSPERO, CRD42016049281

## Background

The introduction of malaria antigen-based rapid diagnostic tests (RDTs) has heralded a paradigm shift in the management of febrile illnesses in malaria-endemic countries. Historically, malaria was considered by default the principal cause of fever, and presumptive antimalarial therapy was widespread policy and practice [1]. In 2010, the World Health Organization (WHO) amended the recommendation for acute fever case management from presumptive antimalarial treatment to parasite-based diagnosis for all populations and endemic areas [1]. While challenges remain for malaria RDT implementation, the availability of relatively simple, reliable, and accessible point-of-care tests means that it has become more straightforward to rule malaria out of the differential diagnosis.

The “test before treat” approach has demonstrated that misclassification of much febrile illness had previously led to an overestimation of malaria incidence [2]. Since the clinical presentations of febrile episodes are often non-specific, definitive diagnosis requires an array of laboratory tests, many unavailable at point of care [3]. Where tests are used, a large proportion of patients with fever still remain undiagnosed. In many African countries, diagnostic facilities are limited, and surveillance networks are often clustered around research institutions, leaving wide geographic swathes with no data on the aetiologies of febrile illnesses [4]. In the absence of reliable data, health care providers often resort to prescription of empiric antimicrobial therapies [5–7], potentially promoting the emergence and spread of antimicrobial resistance (AMR). This has catapulted the improvement of fever case management into the limelight as a global health priority, with a recent proliferation of articles describing aetiologies of non-malarial febrile illnesses (NMFI) in low- and middle-income countries [4, 6, 8–12].

Where reliable diagnostics are lacking, a knowledge of pathogen distributions may inform judicious empiric prescription of antimicrobials [13]. Currently, there is a paucity of information regarding pathogen distribution for many regions in Africa. Furthermore, there is currently no consensus on how to report NMFI aetiology results, making it difficult to view distribution across time and space. To begin to address this knowledge gap, a systematic review of published literature from 1980 to 2015 was conducted, and the results were used to generate an on-line, open-access, interactive map.

## Methods

### Literature search strategy

The systematic review followed the Preferred Reporting Items for Systematic Reviews and Meta-Analyses (PRISMA) guidelines [14], restricted to articles published from 1980 (estimated date of availability of modern diagnostic tests, including molecular testing, for infectious diseases) to 2015 in English and French languages in six databases: MEDLINE, EMBASE, Global Health (CABI) database, WHO Global Health Library, PASCAL, and Bulletin de la Société Française de Parasitologie (BDSP). Search terms were specific for pathogens and symptoms, combined with either “Africa” or individual country names (Additional file 1, Section 1). This review is registered with the PROSPERO (registration ID: CRD42016049281).

### Study selection and full-text review

Titles and abstracts, as well as full texts when the abstract did not provide sufficient information, were first screened for compliance with the inclusion and exclusion criteria (Table 1). One author (JE) independently applied these criteria to identified studies, and the screening was quality controlled by comparing results with a second author (PS) and with colleagues working on harmonised reviews of articles from other geographic regions [15].

**Table 1:** Inclusion and exclusion criteria

**Inclusion criteria**	Reporting on pathogens causing fever in human inpatients or outpatients
	Studies conducted in the targeted geographical areas
	Abstract and full text available in English or French
	Samples tested from normally sterile sites^1^
	Samples analysed in a laboratory setting
	Total number of individuals tested is clearly stated for population-based studies (case reports and case series were categorised separately and did not need to meet this criterion)
**Exclusion criteria**	Published before 1980
	Primary focus on malaria, HIV, or tuberculosis
	Non-clinical studies (descriptions of laboratory methods, modelling studies, economic evaluations, opinion pieces)
	Drug or vaccine trial
	Studies conducted in travellers
	Other studies of disease not including laboratory identification of pathogens causing fever

^1^The definition of a confirmed diagnosis was restricted to pathogens detected in or cultured from samples from normally sterile sites (e.g. bacterial or fungal isolates cultured from the blood, cerebrospinal fluid, arthrocentesis or paracentesis fluid, etc., or virus or parasite detection in the blood or cerebrospinal fluid) or serological evidence of current or past infection

### Data extraction

Data were extracted from selected articles for pre-defined variables on study design, study location, and pathogens (Additional file 1, Section 1). When the study tested for a specific pathogen and did not detect it, that pathogen was not included in the extracted data. Where studies identified fever-causing pathogens other than target pathogens, these were also extracted and included in the database.

### Case definitions

Case definitions were based on laboratory confirmation of infection; clinical criteria were not included. For this review, a confirmed diagnosis was defined as bacterial or fungal isolates detected in or cultured from samples from normally sterile sites (e.g. blood, cerebrospinal fluid, arthrocentesis or paracentesis fluid, or virus or parasite detection in blood or cerebrospinal fluid) or serological evidence of current or past infection. Where additional assays were done (PCR, etc.), these were noted in the database but not used in case definitions due to heterogeneous reporting standards.

### Study type

Studies were categorised into (i) *case series*, which included individual case reports or series of patients with the same infection; (ii) *fever series*, where a group of febrile patients was tested for a number of causative agents and where the total population tested (denominator) was reported; and (iii) *seroprevalence studies*, where serum samples were tested for one pathogen or a panel of pathogens simultaneously. Details regarding the timing of sample collection (e.g. analysis of paired sera) were not extracted for the seroprevalence studies.

### Geographical classification of countries

Countries were classified by sub-region according to United Nations designations [16]. To assess whether data may be biased toward sites near to urban areas, the distance between the study location and nearest major city was calculated using the Havernsine formula assuming the radius of the earth to be 6371 km available in *pracma* package [17] in R software version 3.2.4 (R Foundation for Statistical Computing, Vienna, Austria) [18]. The coordinates of cities in Africa were obtained from the *maps* package [19], and the nearest major city to a given study site was defined as a place with a population greater than 100,000 persons.

### Categorisation of infections

Infections were categorised as bacterial, fungal, parasitic, or viral and were sub-categorised using an epidemiological definition based on their principal mode of transmission as contact (direct, indirect, droplet, or droplet nuclei transmission), vector-borne, air-borne, and food- and/or water-borne. Infections caused by all serotypes of *Salmonella* except for Typhi, Paratyphi A, Paratyphi B, and Paratyphi C were defined as non-typhoidal *Salmonella* (NTS). Details regarding the categorisation of the infections are presented in Additional file 2.

### Study population

Study populations were grouped into four mutually exclusive categories: neonates (aged < 28 days), infants (1–12 months), children (1–12 years), and older (≥ 13 years). If a study reported any participants from each age group, then they were grouped as participants of “all ages”.

### Database and on-line interactive map

An on-line database enabled multiple users (JE, PS) in different locations to work on the review database simultaneously. Each study site location was geo-coded onto an on-line interactive map (surveyor), searchable by country, pathogen, year, and patient age group, and hosted by the Infectious Diseases Data Observatory (IDDO) [20].

### Statistical analyses and risk of bias assessment

The unit of analysis was “published article”. Articles reporting a given pathogen were categorised by geographic region, patient age group, pathogen category, and predominant epidemiologic mode of transmission. In fever series studies that reported the number of individuals tested for a given pathogen (the denominator), the median (range) proportion testing positive was presented by article. The heterogeneity of study design, pathogens sought, laboratory methods, reporting, and limitations in data extraction precluded meta-analysis or estimation of pathogen prevalence.

The currently available tools for assessing the quality and risk of bias were not applicable to our review design [21, 22]. We developed criteria specifically for quality assessment of the studies included in this review (Additional file 3). The risk of bias assessment was based on available information regarding study design and laboratory methods used for the identification of the pathogens. Case reports and series were considered to be at a high risk of bias because they may report atypical presentations and epidemiological outbreaks. Seroprevalence studies were considered to be at moderate risk of bias as the distinction of acute and past infections depends on sample timing. For fever series, studies using culture or PCR methods were considered to be at low risk of bias, those using serological methods at moderate risk, and those not clearly reporting the diagnostic methods used at unclear risk (Additional file 3).

## Results

### Search results

**Fig. 1:**
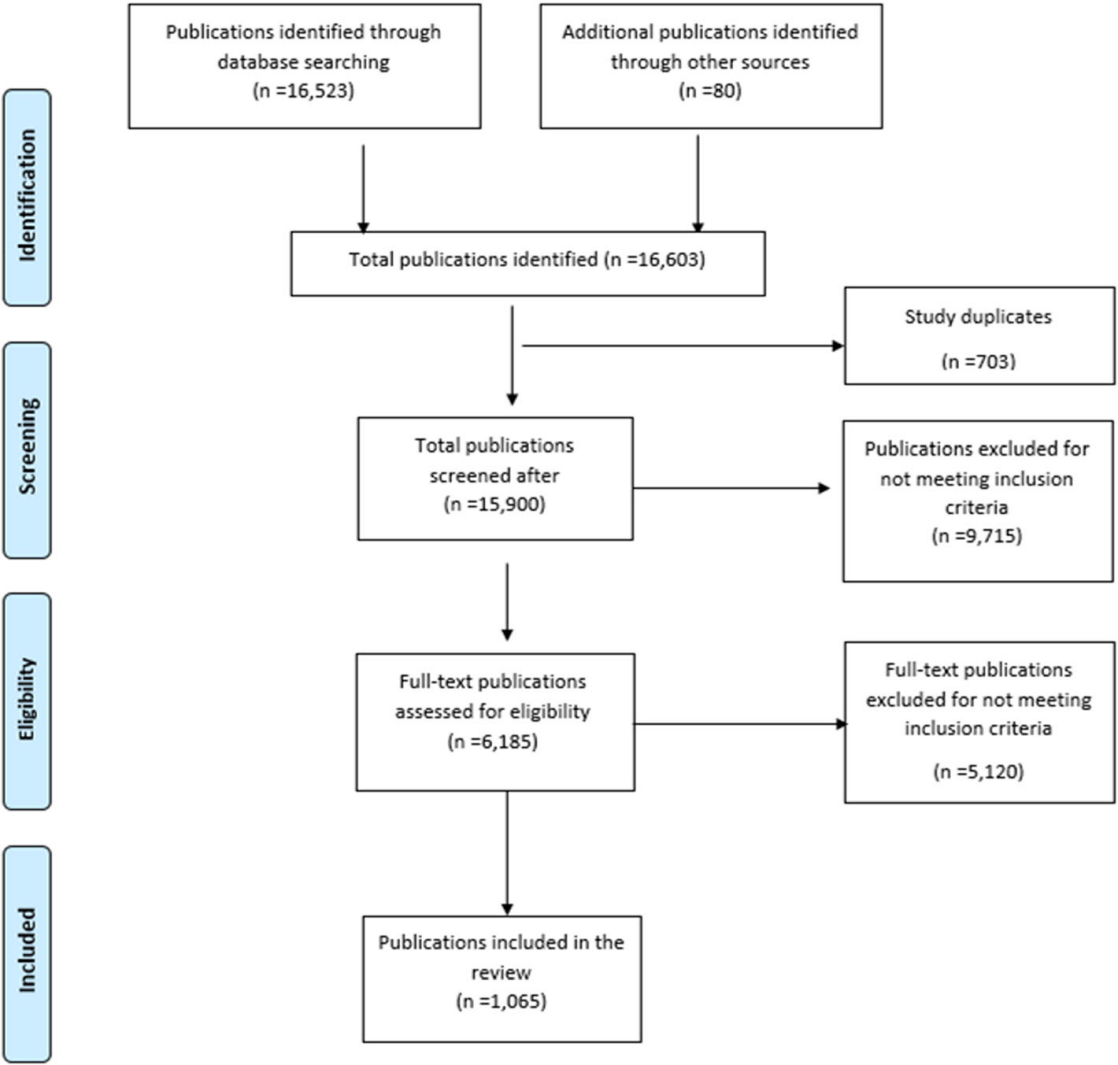
Preferred Reporting Items for Systematic Reviews and Meta-Analyses (PRISMA) flow diagram of publications screened in a systematic review of published aetiological studies and case reports from Africa, 1980–2015

The database search identified 16,523 records, of which 14,777 (89.4%) were in English and 1746 (10.6%) in French. Additional 80 records were identified from the bibliographies of other reports and through the personal knowledge of the co-authors, bringing the total number of articles identified to 16,603. Of the records resulting from the initial search, 703 (4.2%) were identified as duplicates and removed. Of the remaining 15,900 unique records, a further 9715 (61.1%) were excluded after title and abstract screening because they did not fit the selection criteria, leaving 6185 papers for full-text screening. Additional 5120 publications were excluded after full-text assessment bringing the total number of articles included in the review to 1065 (Fig. 1). Of the 1065 publications, 472 (44.3%) were fever series, 412 (38.7%) were case series, 160 (15.0%) were seroprevalence studies, and 21 (2.0%) were of mixed study designs (Additional file 2).

### Spatial distribution

**Fig. 2:**
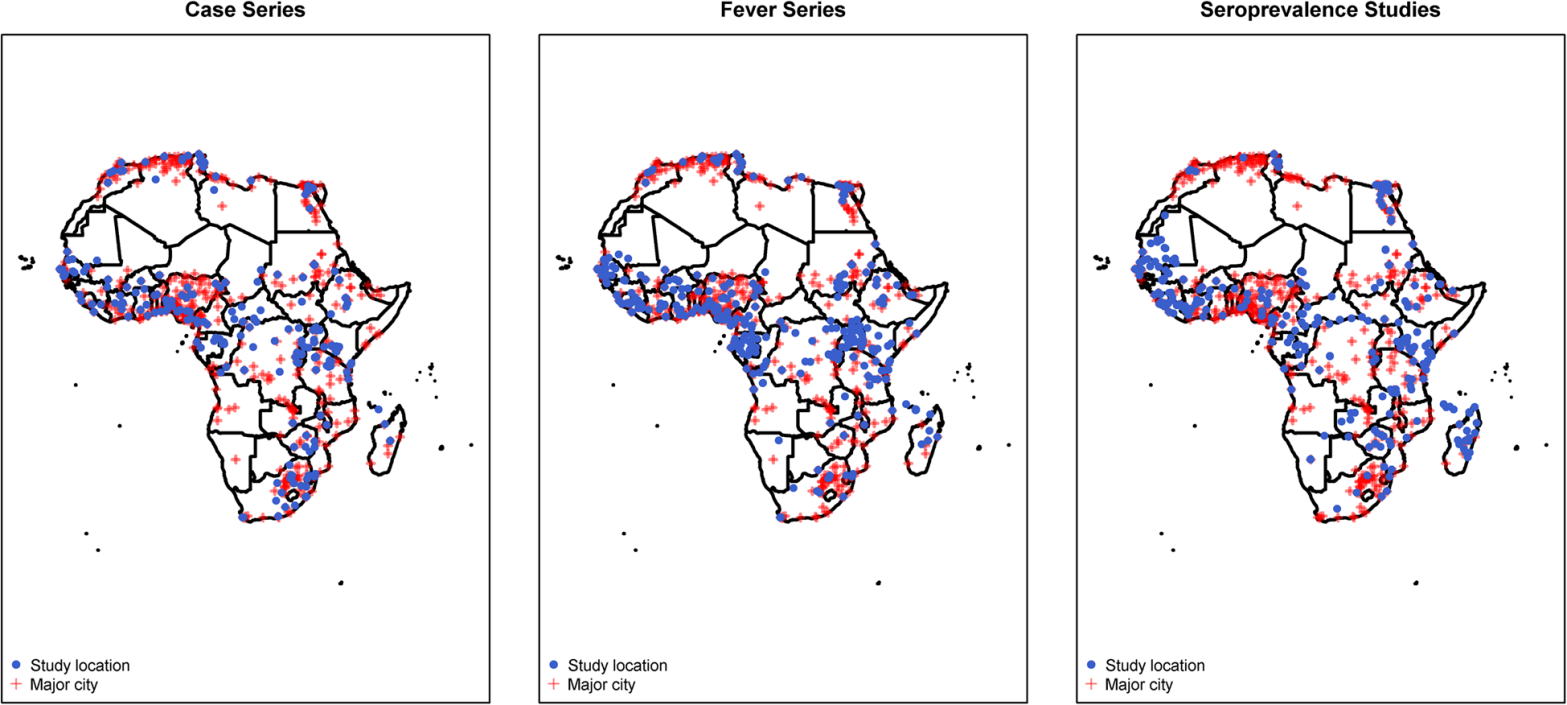
Location of study sites in a systematic review of published aetiological studies and case reports from Africa, 1980–2015. Legend: Location of study sites reported on in this review (in blue) augmented with major cities (in red). Data on major cities were obtained from “*maps*” package in R software, and for the purpose of this review, only cities with population greater than 100,000 are shown. Case series included individual case reports or series of patients with the same condition. Studies were classed as fever series if the total population denominator tested was reported. Seroprevalence studies were those where serum samples were tested for one pathogen or a panel of pathogens simultaneously

**Fig. 3:**
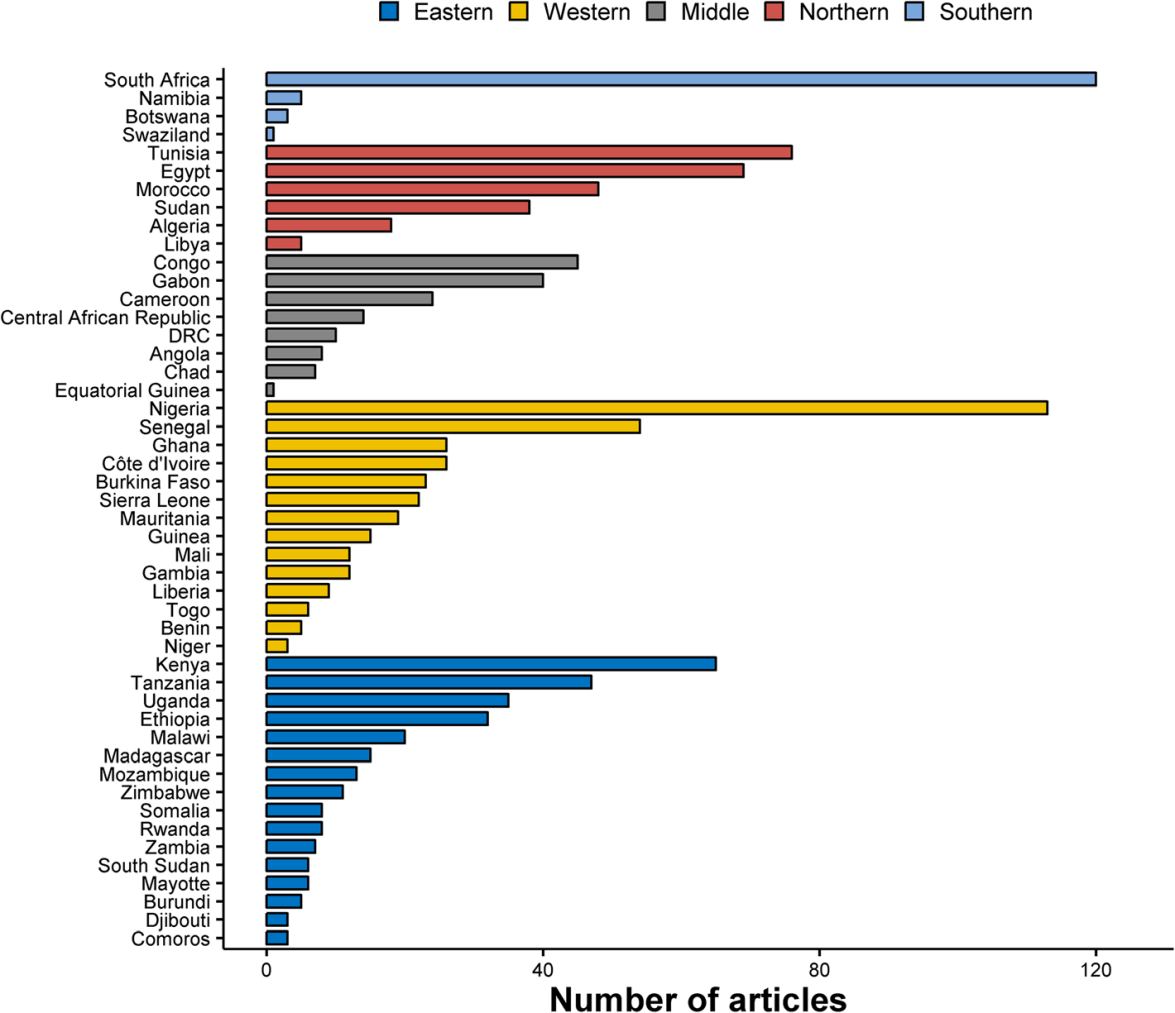
The number of publications by country, in a systematic review of published aetiological studies and case reports from Africa, 1980–2015. Legend: The total number of studies reported from each of the country over the review period from 1980 to 2015. Case series included individual case reports or series of patients with the same condition. Studies were classed as fever series if the total population denominator tested was reported. Seroprevalence studies were defined if serum samples were tested for one pathogen or a panel of pathogens simultaneously

The geographical distribution of the sites in the included papers is shown in Fig. 2; 262 reports (24.6%) were from Eastern Africa, 297 (27.9%) were from Western Africa, 238 (22.3%) were from Northern Africa, 125 (11.7%) from Southern Africa, 122 (11.5%) from Middle Africa, and 21 (2.0%) were multi-regional. South Africa (*n* = 120, 11.3%), Nigeria (*n* = 113, 10.6%), and Tunisia (*n* = 76, 7.1%) contributed the most reports. There were five or fewer articles from Benin, Burundi, Libya, Namibia, Botswana, Comoros, Djibouti, Niger, Equatorial Guinea, and Swaziland (Fig. 3), with no reports from Cabo Verde, Eritrea, Guinea-Bissau, Lesotho, Mauritius, Mauritania, Sao Tome and Principe, and Seychelles.

The median distance between the study sites and the nearest major city was 16.3 km (range 0.2–821.0 km), with 585 (72.5%) of 806 study sites being within a radius of 100 km and 392 (48.6%) of 806 sites being within a radius of 20 km of the nearest major city (Additional file 3).

### Study population

Neonates were reported on in 64 (6.0%) published articles, infants in 12 (1.1%), children aged 1 to < 13 years in 146 (13.7%), and older children and adults (≥ 13 years) in 224 (21.0%). Of all reports, 386 (36.2%) included all ages, while age was not specified in 233 (21.9%) reports. The age distribution for each geographical region is provided in Additional file 1 (Section 2).

### Samples collected and diagnostic methods

Blood was the main specimen analysed in 886 (83.2%) published reports, reflecting our selection criteria. Cerebrospinal fluid (CSF) samples were reported in 79 (7.4%) articles; a combination of CSF and blood culture in 40 (3.8%); bone marrow, joint, or liver aspirates in 13 (1.2%); multiple sample sources in 44 (4.1%); and the source was not stated in three (0.3%) articles (Additional file 1, Section 2).

Bacterial infections were detected using culture methods in 428 (75.9%) reports and serological assays in 111 (19.7%) reports. For viruses, 297 (79.4%) articles reported serological testing and 70 (18.7%) used PCR (Additional file 1, Section 2). Fungal infections were identified using culture methods in eight of nine reports, while parasites were detected using culture method in 20 (42.6%) and serological method in 19 (40.4%) reports.

Additional file 2 presents laboratory methods used over time to identify specific microorganism. For example, the detection of Ebola virus was based on serological tests in 40 of 47 earlier study reports (1983–2005) while 17 out of 23 more recent studies used PCR (207–2015).

### Aetiological findings

Bacterial infections were reported in 564 (53.0%) published articles, viral infections in 374 (35.1%), parasitic infections in 47 (4.4%), and fungal infections in nine (0.8%). Of the 71 (6.7%) articles reporting multiple groups, 46 (64.8%) reported bacteria and fungi; 21 (29.6%) reported bacteria and viruses; two (2.8%) reported bacteria, viruses, and parasites; and two (2.8%) reported bacteria, viruses, and fungi. The median (range) number of pathogens reported in a study was one (1–31) with 827 (77.7%) studies reporting four or fewer pathogens and 82 (7.8%) reporting ≥ 10 pathogens. A list of pathogens reported by country is presented in Additional file 2.

### Bacterial infections

**Fig. 4:**
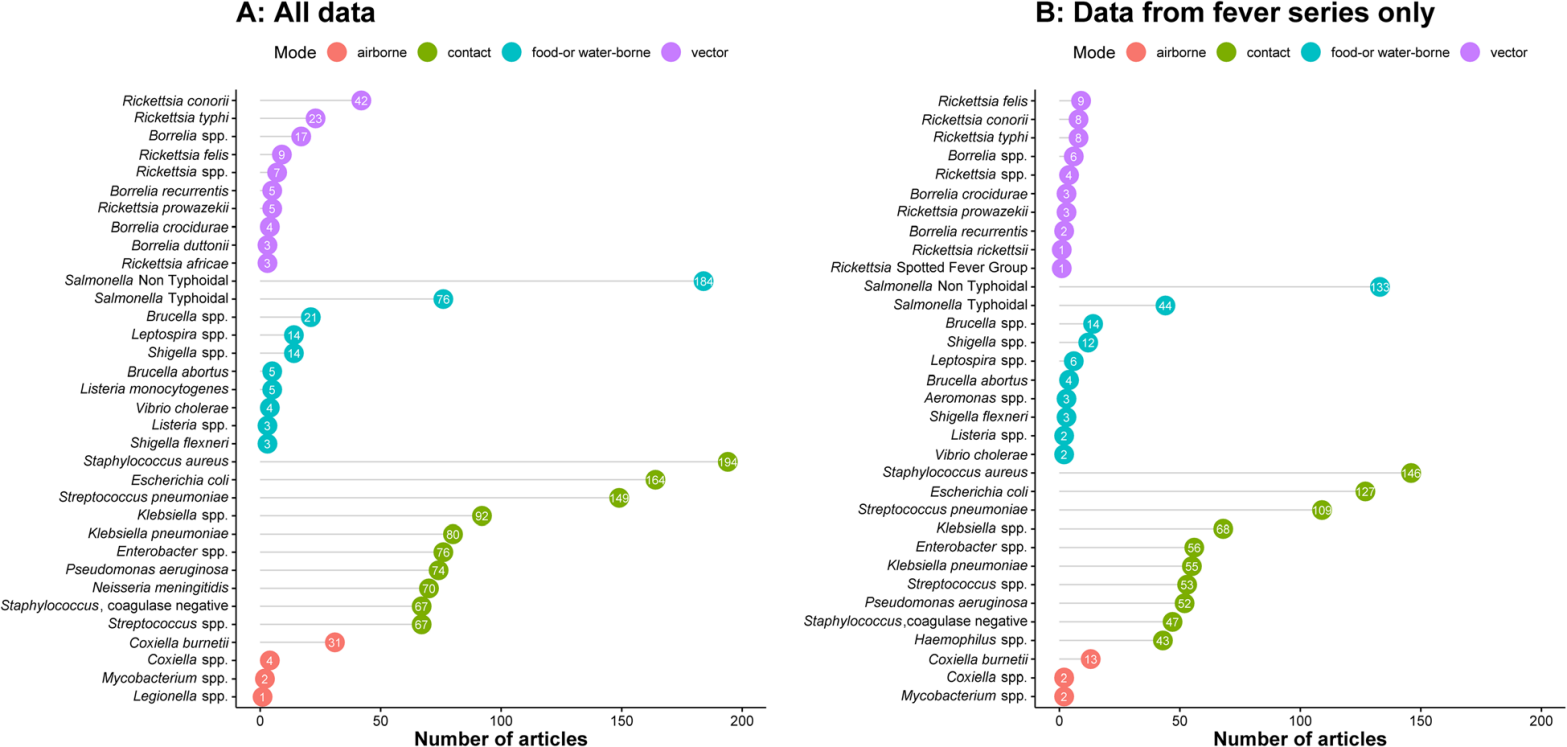
The most commonly reported bacterial infections by mode of transmission, in a systematic review of published aetiological studies and case reports from Africa, 1980–2015. Legend: The left-hand panel includes data from all study types (case series, fever series, and seroprevalence studies). The right-hand panel is restricted to fever series data. The graph presents the top 10 pathogens (based on the number of the published articles) by epidemiological mode of transmission. The numbers inside each dot represent the number of articles

Among 636 published reports of bacterial infections, the most commonly reported were due to *Staphylococcus aureus* (*n* = 194 reports), non-typhoidal *Salmonella* (NTS) (*n* = 184), *Escherichia coli* (*n* = 164), and *Streptococcus pneumoniae* (*n* = 149) (Fig. 4). Among children, *Streptococcus pneumoniae* (*n* = 50), NTS (*n* = 49), and *Staphylococcus aureus* (*n* = 46) were the most reported isolates. Among infants, non-typhoidal *Salmonella* (*n* = 7), *E. coli* (*n* = 7), *Streptococcus agalactiae* (*n* = 6), *Staphylococcus aureus* (*n* = 5), and *Streptococcus pneumoniae* (*n* = 5) were the five most commonly reported. Among neonates, *Klebsiella* spp*.* including *Klebsiella pneumoniae* (*n* = 60), *Escherichia coli* (*n* = 48), *Staphylococcus aureus* (*n* = 41), and *Streptococcus agalactiae* (*n* = 25) were most reported. The overall distribution of articles by patient age and predominant mode of transmission is presented in Additional file 1, section 3.

#### Vector-borne bacterial infections

Rickettsial infections (*n* = 69 published reports) and tick- and louse-borne relapsing fevers caused by *Borrelia* spp. (*n* = 32) were the most commonly reported vector-borne infections (Fig. 4 and Additional files 1 and 2). The predominant rickettsial species reported was *Rickettsia conorii* (*n* = 42), the cause of Mediterranean spotted fever, which is transmitted by the dog tick and appears to have a widespread distribution throughout the African continent. There were five reports of *Borrelia recurrentis*, which is associated with relapsing fever, all from Ethiopia. Among neonates, there was a single report of *Borrelia caucasica* from Rwanda. There were no reports of vector-borne infections among infants.

#### Food- and/or water-borne bacterial infections

Non-typhoidal *Salmonella* (*n* = 184 published reports) was the leading cause of food- and water-borne bacterial infections followed by typhoidal *Salmonella* (*n* = 76), *Brucella* spp. (*n* = 21), *Leptospira* spp. (*n* = 14), and *Shigella* spp. (*n* = 14). Infections due to *Listeria* spp., *Aeromonas* spp., *Cholera* spp., and *Campylobacter* spp. were infrequent and reported in fewer than 10 articles (Fig. 4, and also see Additional file 2). Four cases of *Burkholderia pseudomallei* were reported from Gabon, Madagascar, and Malawi (see Additional file 2).

#### Bacterial infections that spread through contact

*Staphylococcus aureus* (*n* = 194 published reports), *Streptococcus pneumoniae* (*n* = 149), *Klebsiella* spp. (*n* = 92), *Klebsiella pneumonia* (*n* = 80), and *Enterobacter* spp. (*n* = 76) were the most commonly reported bacterial infections in this category. *E. coli* (*n* = 48) was the most commonly reported bacterium among neonates (*n* = 48) and infants (*n* = 7), and *Streptococcus pneumoniae* (*n* = 50) among children aged 1 to < 13 years.

#### Air-borne bacterial infections

*Coxiella* spp*.* (*n* = 35 published reports) of which 31 were due to *Coxiella burnetii*, *Mycobacterium* spp. (*n* = 2), and *Legionella* spp. (*n* = 1) were the only predominantly air-borne bacterial infections reported.

### Viral infections

**Fig. 5:**
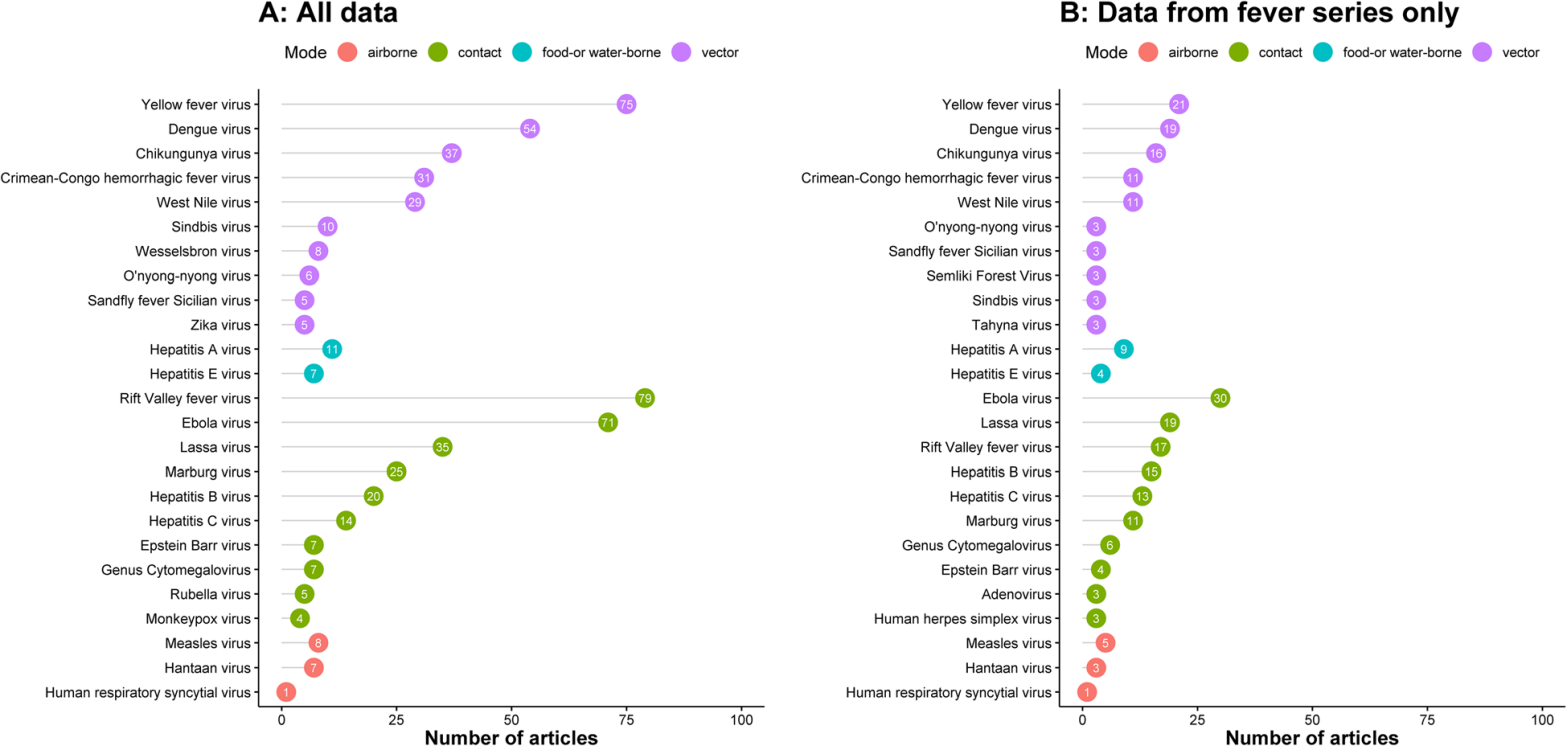
The most commonly reported viral infections by mode of transmission, in a systematic review of published aetiological studies and case reports from Africa, 1980–2015. Legend: The left panel includes data from all the study types (case series, fever series, and seroprevalence studies). The right panel is restricted to the fever series data. The graph presents the top 10 pathogens (based on the number of the published articles) by epidemiological mode of transmission. The numbers inside each dot represent the number of articles

**Fig. 6:**
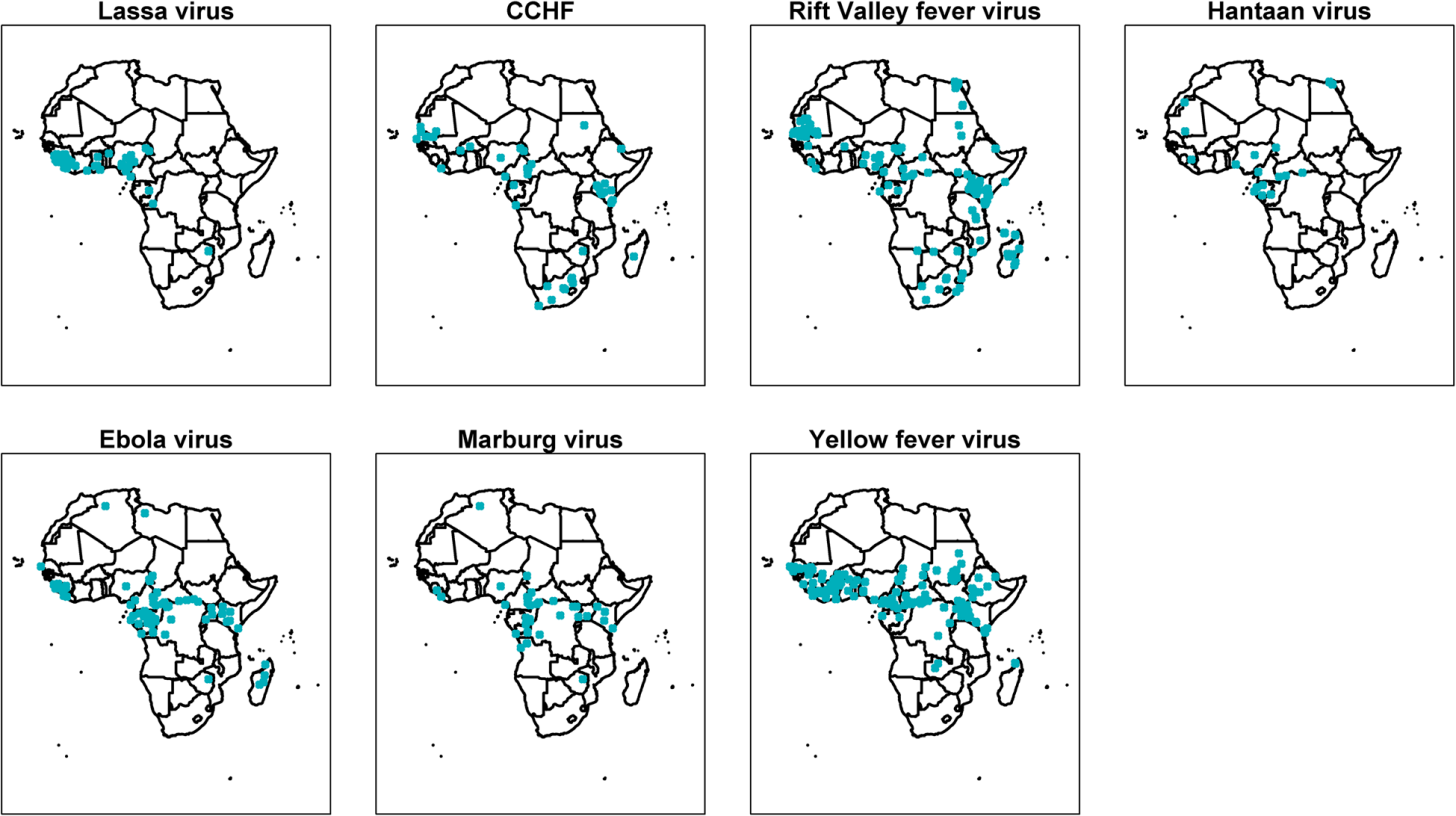
Viral haemorrhagic fever, in a systematic review of published aetiological studies and case reports from Africa, 1980–2015. Legend: CCHF Crimean-Congo haemorrhagic fever virus. The map shows the location of study sites reporting each pathogen. No distinction has been made between case series, fever series, and seroprevalence studies

Among 415 published reports of viral infections, there were none in infants, and only three in neonates attributed to cytomegalovirus, enterovirus, and herpes simplex virus once each (Additional file 2). The distribution of the most commonly reported viral infections is presented in Fig. 5 and of viral haemorrhagic fevers (VHFs) in Fig. 6.

#### Vector-borne viral infections

**Fig. 7:**
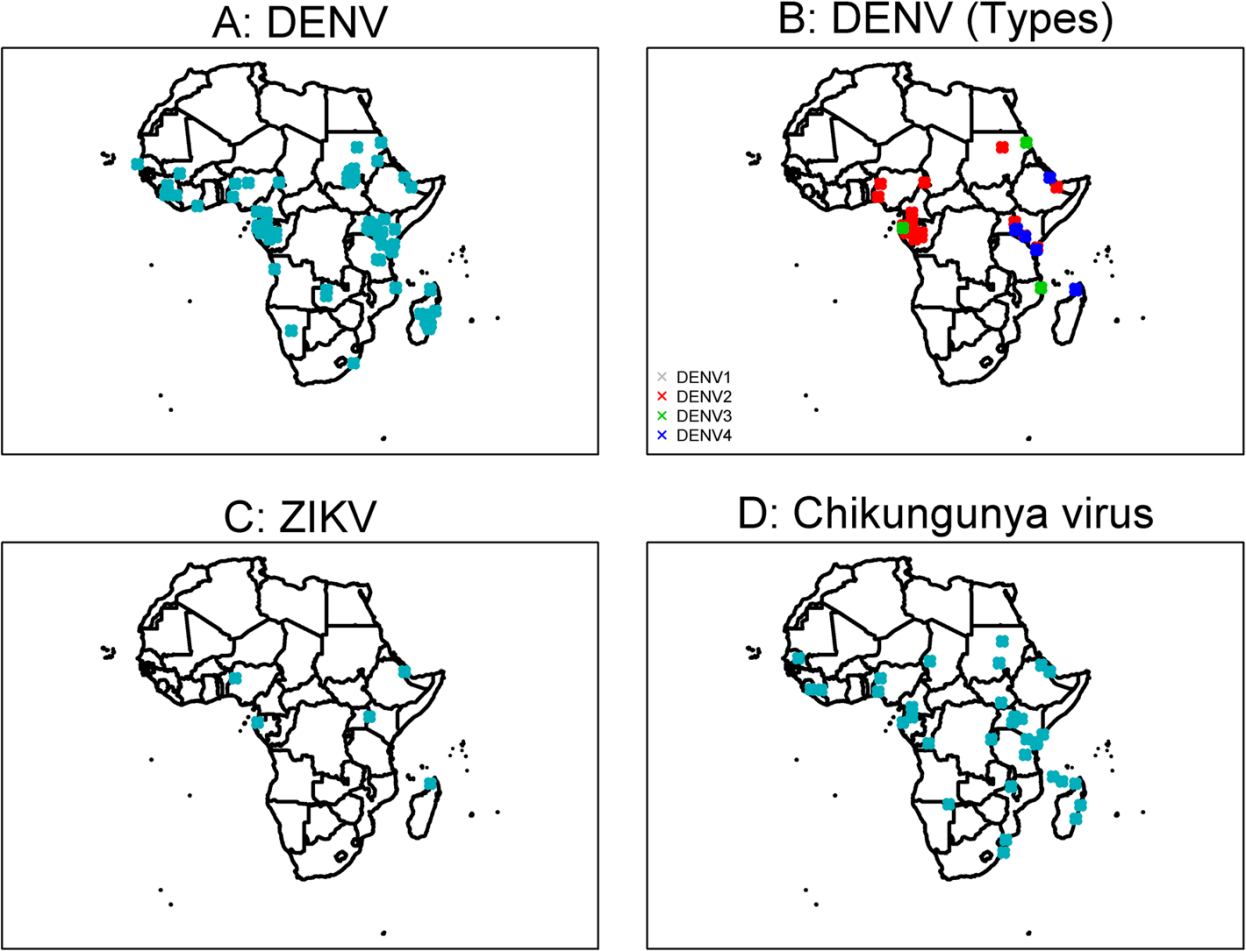
Reports of dengue, chikungunya, and Zika viral infections, a systematic review of published aetiological studies and case reports from Africa, 1980–2015. Legend: DENV dengue virus, ZIKV Zika virus. For DENV, serotypes were not reported in 35 publications. The map shows the location of study sites reporting each pathogen. No distinction has been made between case series, fever series, and seroprevalence studies

Yellow fever virus (*n* = 75 published reports) was the most commonly reported arbovirus. Dengue virus (DENV) serotypes 1 to 4 were reported in 54 articles from 20 countries (Figs. 5 and 6), the majority of which were published from 2010 through 2015 (61.1%, 33/54) with serotype 2 being the most reported (*n* = 17). Chikungunya virus was reported in 37 articles, the majority from studies conducted in Eastern Africa (62.1%, 23/37) (Fig. 7). Zika virus was reported in one article each from Nigeria in 1983, Madagascar in 1989, Uganda in 1989, Djibouti in 1996, and Gabon in 2014 (Additional file 2).

#### Food- and/or water-borne viral infections

Hepatitis A virus was reported in 11 studies from Egypt (*n* = 6), Ghana (*n* = 1), Morocco (*n* = 2), South Africa (*n* = 1), and Ivory Coast (*n* = 1). Hepatitis E was reported in Egypt in three studies and in one study each from Ethiopia, Ghana, Kenya, Somalia, and Sudan (Additional file 2). Enteroviruses were reported in an article each from Malawi, Morocco, Senegal, South Africa, and Tunisia. Enteroviruses among neonates were reported in the article from Morocco, among children from the articles in South Africa and Tunisia, and among participants of all ages from Malawi and Senegal (Additional file 2).

#### Air-borne viral infections

Measles virus was reported in eight articles in Nigeria (*n* = 3), Sudan (*n* = 3), Ghana (*n* = 1), and Somalia (*n* = 1). Hantaan virus was reported in seven articles from Cameroon, Central African Republic, Chad, Egypt, Guinea, Equatorial Guinea, Gabon, Mauritania, and Nigeria. Respiratory syncytial virus (RSV; *n* = 1) was reported in an article from Egypt. No other air-borne viruses were reported.

#### Viral infections spreading through contact

Rift Valley fever virus (*n* = 79 published reports) was the most common viral infection reported in this category. Ebola virus disease (*n* = 71 reports) was the second most common viral infection reported in this category, of which 18 (25.3%) were published during the 2014–2016 West African outbreak. The other most frequently reported viruses were Lassa virus (*n* = 35), Marburg virus (*n* = 25), hepatitis B virus (*n* = 20), and hepatitis C virus (*n* = 14) (Fig. 5).

### Fungal infections

A list of all reported fungal infections, from 57 published articles, is presented in Additional file 1 (Section 3). *Candida* spp. were the most commonly reported causes of fungal infections among neonates (*n* = 13 reports) and children (*n* = 7). *Cryptococcus* spp. (*n* = 7) were commonly reported among adults and were reported in studies from Botswana, Malawi, and South Africa; of these, two studies included some HIV-infected patients.

### Parasitic infections

Among the 49 published articles reporting non-malaria parasitic infections, leishmaniasis was the most commonly reported (*n* = 28 reports) among both children (*n* = 7) and adults (*n* = 8) (Additional file 1, Section 3). *Leishmania* spp. were reported from Cameroon, Chad, Ivory Coast, Ethiopia, Gambia, Morocco, Sierra Leone, Somalia, Sudan, and Tunisia with the majority of articles being from Tunisia (*n* = 11). *Trypanosoma* spp. (*n* = 4), *Mansonella perstans* (*n* = 2), *Wuchereria bancrofti* (*n* = 2), and *Babesia* spp. (*n* = 1) were the pathogens of the other vector-borne parasitic infections. *Fasciola hepatica*, the common liver fluke, was reported in three articles from Egypt, *Schistosoma* spp. in three articles from Egypt, *Taenia* spp. in an article each from Egypt and South Africa, and *Loa loa* in an article from Nigeria (Additional file 2). There were no reports of parasitic infections among neonates and infants.

### Spatial and temporal trends in infectious causes of fever

The most commonly reported fever-causing pathogens in each of the geographical regions over the period of this systematic review are presented in Tables 2, 3, 4, 5, and 6 (see Additional file 1, Section 4 for further details). Rift Valley fever (RVF) virus predominated in reports from Eastern Africa (Table 2), and yellow fever virus, RVF, and Lassa virus from Western Africa (Table 3). In Northern Africa, RVF was the most commonly reported virus until 2000, after which no reports were identified (Table 4). Viruses causing haemorrhagic fever predominated in Middle African reports (Table 6). *E. coli*, *Staphylococcus aureus*, typhoidal and non-typhoidal *Salmonella*, and *Streptococcus pneumoniae* were the most commonly reported bacterial causes of fever in Eastern and Western Africa throughout the study period (Tables 2 and 3).

**Table 2:** The top five most commonly reported pathogens in Eastern Africa, stratified by time period

	1980 to ≤ 1990	1991 to ≤ 2000	2001 to ≤ 2010	2011 to ≤ 2015
Bacteria	Typhoidal *Salmonella* (*n* = 5)	*Streptococcus pneumoniae* (*n* = 7)	Non-typhoidal *Salmonella* (*n* = 30)	*Staphylococcus aureus* (*n* = 29)
	Non-typhoidal *Salmonella* (*n* = 4)	*Staphylococcus aureus* (*n* = 7)	*Streptococcus pneumoniae* (*n* = 24)	Non-typhoidal *Salmonella* (*n* = 28)
	*Borrelia* spp. (*n* = 4)	*Escherichia coli* (*n* = 7)	*Staphylococcus aureus* (*n* = 22)	*Escherichia coli* (*n* = 23)
	*Staphylococcus aureus* (*n* = 3)	*Rickettsia typhi* (*n* = 5)	*Escherichia coli* (*n* = 22)	*Streptococcus pneumoniae* (*n* = 20)
	*Streptococcus pneumoniae* (*n* = 2)	*Haemophilus* spp. (*n* = 5)	*Haemophilus* spp*.* (*n* = 20)	*Klebsiella* spp. (*n* = 13)
Viruses	Rift Valley fever virus (*n* = 6)	Rift Valley fever virus (*n* = 9)	Ebola virus (*n* = 9)	Dengue virus (*n* = 13)
	Ebola virus (*n* = 5)	Yellow fever virus (*n* = 5)	Rift Valley fever virus (*n* = 7)	Chikungunya virus (*n* = 12)
	Chikungunya virus (*n* = 5)	O’nyong-nyong virus (*n* = 3)	Chikungunya virus (*n* = 4)	Rift Valley fever virus (*n* = 11)
	Wesselsbron virus (*n* = 4)	West Nile virus (*n* = 2)	Marburg virus (*n* = 2)	Yellow fever virus (*n* = 9)
	CCHF virus (*n* = 3)	CCHF virus (*n* = 2)	Dengue virus (*n* = 2)	West Nile virus (*n* = 4)
Parasites	Family Trypanosomatidae (*n* = 1)	*Toxocara* spp. (*n* = 1)	*Wuchereria bancrofti* (*n* = 1)	*Leishmania* spp. (*n* = 1)
	–	–	*Trypanosoma* spp. (*n* = 1)	*Babesia* spp. (*n* = 1)
	–	–	*Leishmania* spp. (*n* = 1)	–
Fungi	*Cryptococcus* spp. (*n* = 1)	*Candida albicans* (*n* = 2)	*Cryptococcus* spp. (*n* = 2)	*Cryptococcus* spp. (*n* = 3)
	–	*Candida* spp. (*n* = 1)	Fungus (*n* = 1)	*Candida* spp. (*n* = 2)
	–	–	*Candida* spp. (*n* = 1)	–

*CCHF* Crimean-Congo haemorrhagic fever virus. No distinction has been made between case series, fever series, and seroprevalence studies. Numbers in parenthesis indicate the number of publications reporting the given microorganism. The complete list of the microorganisms reported in a given region and time period is provided in Additional file 2

**Table 3:** The top five most commonly reported pathogens in Western Africa, stratified by time period

	1980 to ≤ 1990	1991 to ≤ 2000	2001 to ≤ 2010	2011 to ≤ 2015
Bacteria	*Staphylococcus aureus* (*n* = 7)	*Staphylococcus aureus* (*n* = 11)	*Staphylococcus aureus* (*n* = 20)	*Staphylococcus aureus* (*n* = 22)
	Typhoidal *Salmonella* (*n* = 5)	*Streptococcus pneumoniae* (*n* = 9)	*Streptococcus pneumoniae* (*n* = 16)	*Escherichia coli* (*n* = 21)
	*Klebsiella* spp. (*n* = 4)	Non-typhoidal *Salmonella* (*n* = 8)	*Escherichia coli* (*n* = 16)	*Streptococcus pneumoniae* (*n* = 16)
	*Citrobacter* spp. (*n* = 4)	*Neisseria meningitidis* (*n* = 7)	*Klebsiella* spp. (*n* = 11)	*Klebsiella* spp. (*n* = 13)
	Non-typhoidal *Salmonella* (*n* = 4)	*Escherichia coli* (*n* = 5)	*Streptococcus* spp. (*n* = 10)	*Klebsiella pneumoniae* (*n* = 12)
Viruses	Yellow fever virus (*n* = 14)	Yellow fever virus (*n* = 11)	Yellow fever virus (*n* = 11)	Ebola virus (*n* = 15)
	Lassa virus (*n* = 12)	Rift Valley fever virus (*n* = 3)	Lassa virus (*n* = 6)	Lassa virus (*n* = 11)
	Rift Valley fever virus (*n* = 8)	Lassa virus (*n* = 2)	Rift Valley fever virus (*n* = 4)	Rift Valley fever virus (*n* = 7)
	CCHF virus (*n* = 7)	Measles virus (*n* = 1)	CCHF virus (*n* = 2)	Dengue virus (*n* = 7)
	Chikungunya virus (*n* = 3)	CCHF virus (*n* = 1)	Dengue virus (*n* = 2)	Yellow fever virus (*n* = 5)
Parasites	Loa loa (*n* = 1)	*Leishmania* spp. (*n* = 1)	*Trypanosoma* spp. (*n* = 1)	*Wuchereria bancrofti* (*n* = 1)
	*Leishmania* spp. (*n* = 1)	–	*Leishmania* spp. (*n* = 1)	*Mansonella perstans* (*n* = 1)
Fungi	–	*Candida* spp. (*n* = 1)	–	*Candida* spp. (*n* = 2)
	–	*Candida albicans* (*n* = 1)	–	*Penicillium* spp. (*n* = 1)
	–	–	–	*Cryptococcus* spp. (*n* = 1)
	–	–	–	*Candida albicans* (*n* = 1)
	–	–	–	*Aspergillus niger* (*n* = 1)

CCHF Crimean-Congo haemorrhagic fever virus. No distinction has been made between case series, fever series, and seroprevalence studies. Numbers in parentheses indicate the number of publications reporting the given microorganism. The complete list of the microorganisms reported in a given region and time period is provided in Additional file 2

**Table 4:** The top five most commonly reported pathogens in Northern Africa, stratified by time period

	1980 to ≤ 1990	1991 to ≤ 2000	2001 to ≤ 2010	2011 to ≤ 2015
Bacteria	Non-typhoidal *Salmonella* (*n* = 3)	*Coxiella burnetii* (*n* = 7)	*Staphylococcus aureus* (*n* = 22)	*Escherichia coli* (*n* = 18)
	*Rickettsia conorii* (*n* = 3)	Non-typhoidal *Salmonella* (*n* = 5)	*Escherichia coli* (*n* = 14)	*Klebsiella pneumoniae* (*n* = 13)
	*Neisseria meningitidis* (*n* = 3)	*Rickettsia conorii* (*n* = 5)	*Rickettsia conorii* (*n* = 11)	*Staphylococcus aureus* (*n* = 10)
	*Streptococcus pneumoniae* (*n* = 2)	*Rickettsia typhi* (*n* = 4)	*Pseudomonas aeruginosa* (*n* = 11)	*Staphylococcus*, coagulase negative (*n* = 9)
	Typhoidal *Salmonella* (*n* = 2)	*Streptococcus pneumoniae* (*n* = 3)	*Streptococcus* spp. (*n* = 9)	*Streptococcus pneumoniae* (*n* = 7)
Viruses	Rift Valley fever virus (*n* = 3)	Rift Valley fever virus (*n* = 6)	Hepatitis C virus (*n* = 3)	Dengue virus (*n* = 8)
	Hepatitis B virus (*n* = 2)	West Nile virus (*n* = 3)	Hepatitis B virus (*n* = 3)	Yellow fever virus (*n* = 6)
	Hepatitis A virus (*n* = 2)	Sandfly fever Sicilian virus (*n* = 3)	Hepatitis A virus (*n* = 3)	Hepatitis C virus (*n* = 5)
	West Nile virus (*n* = 1)	Sandfly fever Naples virus (*n* = 3)	West Nile virus (*n* = 2)	West Nile virus (*n* = 3)
	Sandfly fever Naples virus (*n* = 1)	Hantaan virus (*n* = 2)	Dengue virus (*n* = 2)	Toscana virus (*n* = 3)
Parasites	–	*Leishmania* spp. (*n* = 8)	*Leishmania* spp. (*n* = 7)	*Leishmania* spp. (*n* = 6)
	–	*Fasciola hepatica* (*n* = 3)	–	*Schistosoma* spp. (*n* = 2)
	–	*Toxoplasma* spp. (*n* = 1)	–	–
	–	*Toxocara* spp. (*n* = 1)	–	–
	–	*Taenia* spp. (*n* = 1)	–	–
Fungi	–	*Aspergillus fumigatus* (*n* = 2)	*Candida albicans* (*n* = 4)	*Candida* spp. (*n* = 5)
	–	*Candida* spp. (*n* = 1)	*Candida* spp. (*n* = 3)	*Candida* albicans (*n* = 4)
	–	*Candida albicans* (*n* = 1)	*Penicillium marneffei* (*n* = 1)	*Candida parapsilosis* (*n* = 2)
	–	–	*Mucor* spp. (*n* = 1)	*Candida ciferrii* (*n* = 2)
	–	–	Histoplasma (*n* = 1)	*Blastoschizomyces pseudotrichosporon* (*n* = 2)

No distinction has been made between case series, fever series, and seroprevalence studies. Numbers in parenthesis indicate the number of publications reporting the given microorganism. The complete list of the microorganisms reported in a given region and time period is provided in Additional file 2

**Table 5:** The top five most commonly reported pathogens in Southern Africa, stratified by time period

	1980 to ≤ 1990	1991 to ≤ 2000	2001 to ≤ 2010	2011 to ≤ 2015
Bacteria	Non-typhoidal *Salmonella* (*n* = 8)	*Streptococcus pneumoniae* (*n* = 7)	*Streptococcus pneumoniae* (*n* = 4)	*Staphylococcus aureus* (*n* = 15)
	*Streptococcus pneumoniae* (*n* = 7)	*Staphylococcus aureus* (*n* = 5)	*Staphylococcus aureus* (*n* = 4)	*Escherichia coli* (*n* = 10)
	*Neisseria meningitidis* (*n* = 7)	*Staphylococcus*, coagulase negative (*n* = 3)	*Pseudomonas aeruginosa* (*n* = 3)	*Klebsiella pneumoniae* (*n* = 8)
	*Staphylococcus aureus* (*n* = 6)	*Klebsiella* spp. (*n* = 3)	*Escherichia coli* (*n* = 3)	*Enterobacter* spp. (*n* = 8)
	*Haemophilus* spp. (*n* = 4)	*Klebsiella pneumoniae* (*n* = 3)	*Streptococcus*, viridans group (*n* = 2)	*Acinetobacter baumannii* (*n* = 8)
Viruses	CCHF virus (*n* = 6)	CCHF virus (*n* = 3)	–	Rift Valley fever virus (*n* = 3)
	Rift Valley fever virus (*n* = 5)	Genus *Cytomegalovirus* (*n* = 1)	–	Wesselsbron virus (*n* = 1)
	West Nile virus (*n* = 2)	Adenovirus (*n* = 1)	–	Sindbis virus (*n* = 1)
	Wesselsbron virus (*n* = 2)	–	–	Human herpes simplex virus type 1 (*n* = 1)
	Sindbis virus *(n* = 2)	–	–	Human enterovirus (*n* = 1)
Parasites	*Taenia* spp. (*n* = 1)	–	–	–
	–			
Fungi	*Candida* spp. (*n* = 1)	*Candida albicans* (*n* = 1)	*Candida albicans* (*n* = 2)	*Candida* spp. (*n* = 5)
	–	–	*Candida* spp. (*n* = 1)	*Cryptococcus* spp. (*n* = 4)
	–	–	*Candida parapsilosis* (*n* = 1)	*Candida albicans* (*n* = 4)
	–	–	Fungus (*n* = 1)	*Candida parapsilosis* (*n* = 3)
	–	–	–	*Candida tropicalis* (*n* = 2)

CCHF Crimean-Congo haemorrhagic fever virus. No distinction has been made between case series, fever series, and seroprevalence studies. Numbers in parenthesis indicate the number of publications reporting the given microorganism

**Table 6:** The top five most commonly reported pathogens in Middle Africa, stratified by time period

	1980 to ≤ 1990	1991 to ≤ 2000	2001 to ≤ 2010	2011 to ≤ 2015
Bacteria	Non-typhoidal *Salmonella* (*n* = 5)	Non-typhoidal *Salmonella* (*n* = 5)	Non-typhoidal *Salmonella* (*n* = 5)	*Staphylococcus aureus* (*n* = 8)
	*Pseudomonas* spp. (*n* = 2)	*Streptococcus pneumoniae* (*n* = 3)	*Streptococcus pneumoniae* (*n* = 5)	Non-typhoidal *Salmonella* (*n* = 7)
	*Klebsiella pneumoniae* (*n* = 2)	*Rickettsia conorii* (*n* = 3)	*Neisseria meningitidis* (*n* = 4)	*Escherichia coli* (*n* = 6)
	*Escherichia coli* (*n* = 2)	*Neisseria meningitidis* (*n* = 3)	*Haemophilus* spp. (*n* = 3)	*Streptococcus pneumoniae* (*n* = 4)
	*Enterobacter* spp. (*n* = 2)	*Coxiella burnetii* (*n* = 2)	*Escherichia coli* (*n* = 3)	*Enterobacter* spp. (*n* = 4)
Viruses	Ebola virus (*n* = 6)	Ebola virus (*n* = 12)	Ebola virus (*n* = 9)	Yellow fever virus (*n* = 6)
	Rift Valley fever virus (*n* = 5)	Marburg virus (*n* = 6)	Marburg virus (*n* = 8)	Ebola virus (*n* = 4)
	Yellow fever virus (*n* = 3)	Hepatitis B virus (*n* = 2)	Yellow fever virus (*n* = 5)	Dengue virus (*n* = 3)
	Marburg virus (*n* = 2)	Saint-Floris virus (*n* = 1)	West Nile virus (*n* = 3)	Chikungunya virus (*n* = 2)
	Lassa virus (*n* = 2)	Rift Valley Fever virus (*n*-1)	Dengue virus (*n* = 3)	West Nile virus (*n* = 1)
Parasites	–	*Trypanosoma* spp. (*n* = 1)	*Entamoeba histolytica* (*n* = 1)	*Trypanosoma* spp. (*n* = 1)
	–	*Leishmania* spp. (*n* = 1)	–	*Mansonella perstans* (*n* = 1)
	–	–	–	*Leishmania* spp. (*n* = 1)

No distinction has been made between case series, fever series, and seroprevalence studies. Numbers in parenthesis indicate the number of publications reporting the given microorganism. There were no data on fungal infections in Middle Africa

### Vaccine-preventable infections

**Fig. 8:**
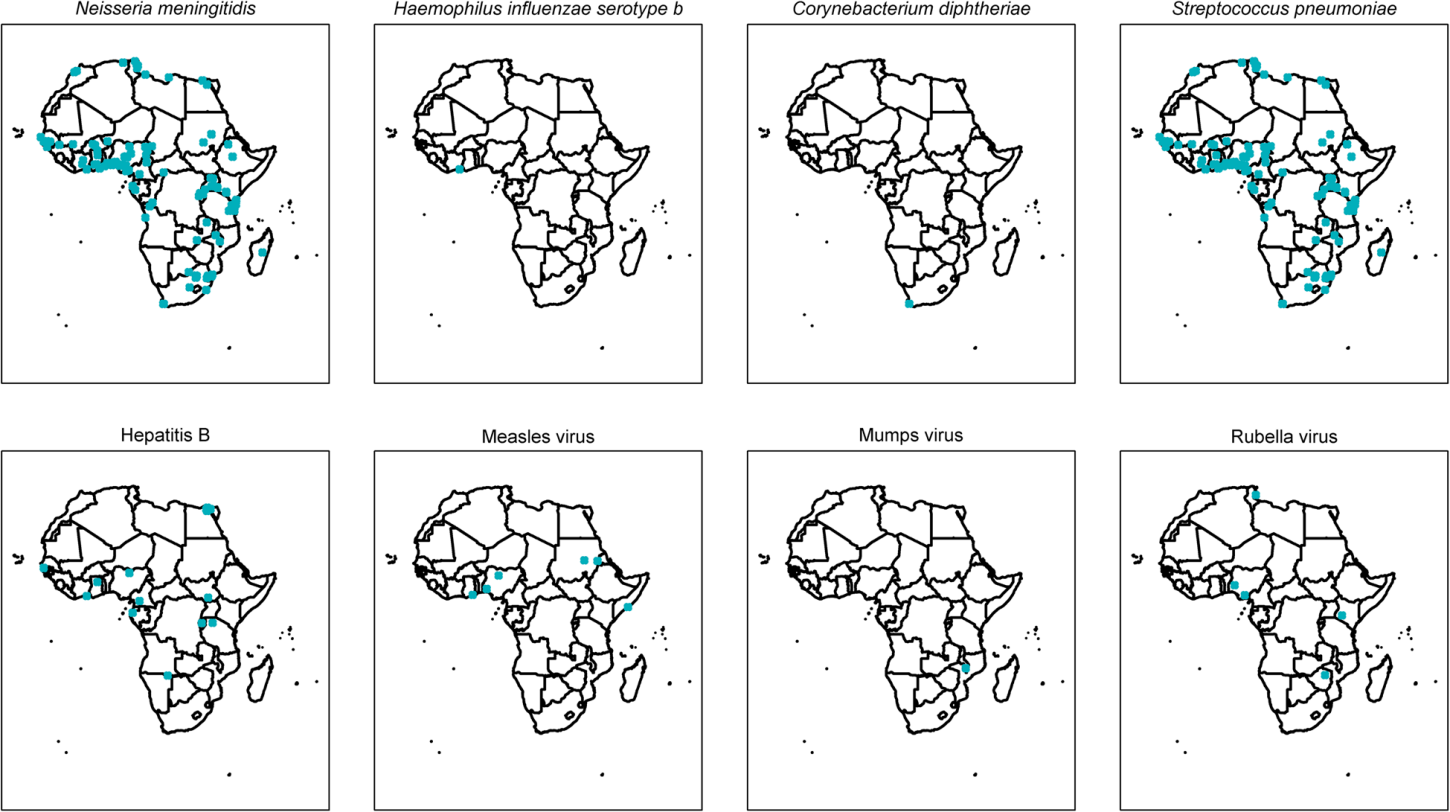
Studies reporting vaccine-preventable infections which are part of the WHO routine Expanded Programme on Immunisation, in a systematic review of published aetiological studies and case reports from Africa, 1980–2015. Legend: The map shows the location of study sites reporting each pathogen. No distinction has been made between case series, fever series, and seroprevalence studies

Figure 8 presents the sites of studies reporting some vaccine-preventable infections that are part of the WHO routine Expanded Programme on Immunisation (EPI) [23]. Infection with *Haemophilus influenzae* type b, hepatitis B, measles, mumps, rubella, yellow fever, and invasive infections caused by *Neisseria meningitidis* or *Streptococcus pneumoniae* are all reported, with an increasing number of publications on the latter two organisms over time. There was only one report of *Corynebacterium diphtheriae* from South Africa among adults, and there were no reports of pertussis or tetanus.

### Assessment of risk of bias

Among 1065 articles, the risk of bias was considered to be high in 426 articles (40.0%), moderate in 322 (30.2%), and low in 305 (28.6%). In the remaining 12 (1.1%) articles, the risk of bias was unclear (Additional file 3, Section 1). There was a reporting bias toward urban settings (Additional file 3, Section 2).

## Discussion

In Africa, the causes of non-malarial febrile illness and the distributions of associated pathogens are poorly understood due to the lack of aetiological testing [5, 10, 24]. As malaria is less prevalent than previously thought, and declining further in some areas, understanding the aetiology of fever is an increasingly important public health challenge [25, 26]. Management of non-malarial febrile illness in resource-constrained settings poses a conundrum to patients and health workers, who are often compelled to use antimicrobials empirically [27]. In addition to inappropriately treating individual patients, antimicrobial prescribing may be an important driver of antimicrobial drug resistance. To characterise the spectrum of causative pathogens for non-malarial febrile illnesses in Africa, a systematic review of published literature, to our knowledge the largest on this topic, was undertaken.

This review makes several observations that are highly relevant from a public health perspective. It demonstrates a broad diversity and heterogeneity of information reported about patient populations, laboratory methods, quality assurance of laboratory standards, and the wide spectrum of microorganisms detected and identified. This observation expands on similar findings in a narrative review of recent studies of NMFI in Africa [28]. This review also reveals heterogeneous and often poor reporting of study population ages, with 21.9% of articles not mentioning the age range of patients tested, despite this information being essential to interpret clinical and epidemiological studies, and to guide policy decisions. Over a third of the identified studies were published from 2010 to 2015, a time period after the introduction of the “test before treat” approach for the case management of malaria. This illustrates a recent heightened interest in identifying the causes of febrile illnesses. Just over 70% of the study sites were located in urban centres or within a radius of 100 km from the nearest city. While urbanisation is increasing globally, as of 2018, the United Nations estimated that Africa remains mostly rural with just 43% of the population living in urban areas [29]; so this reporting bias represents a substantial knowledge gap for causes of fever in rural environments.

Bacterial or viral infections were the most commonly reported pathogen groups, together constituting more than 80% of the articles included in this review. In children, *Streptococcus pneumoniae*, *Staphylococcus aureus*, and *Salmonella enterica* were commonly reported while *Klebsiella* spp*.*, *Escherichia coli*, *Staphylococcus aureus*, and *Streptococcus agalactiae* were the most reported isolates in the neonatal age group. Vaccine-preventable infections that are part of the routine EPI continue to be reported, although there was no report of pertussis, which is common in Africa [30]; the absence of reports in this review may be explained both by the exclusion of respiratory specimen testing and by the absence of accessible diagnostics.

Most of the bacterial pathogens reported are well known and common in countries at all income levels (Table 7). However, there were some exceptions. For example, there were only four reported cases of *Burkholderia pseudomallei*, from Madagascar, Malawi, and Gabon. *B. pseudomallei* is notoriously easy to miss in the clinical microbiology laboratory if the index of suspicion is low and it is not specifically sought. A recent modelling study has predicted the disease to be endemic and under-reported in much of sub-Saharan Africa [31], a result consistent with the environmental surveys [32, 33]. Taken together, they provide evidence that this serious infection is being under-diagnosed in Africa. The reports of *Klebsiella* spp. as a common causative agent of neonatal sepsis are noteworthy, given the intrinsic resistance of this pathogen to amoxicillin, the empiric treatment (with gentamicin) for neonatal sepsis in many LMICs. Other studies over the last decade have suggested that *Klebsiella* is being diagnosed more frequently than before in early onset neonatal sepsis [34, 35].

**Table 7:** Commonly reported bacterial pathogens among neonates, stratified by region and time period

	1980 to ≤ 1990	1991 to ≤ 2000	2001 to ≤ 2010	2011 to ≤ 2015
**Region**				
Eastern	*Borrelia caucasica* (*n* = 1)	*Staphylococcus aureus* (*n* = 3)	*Streptococcus agalactiae* (*n* = 6)	*Staphylococcus aureus* (*n* = 6)
	–	*Escherichia coli* (*n* = 3)	*Escherichia coli* (*n* = 6)	*Escherichia coli* (*n* = 6)
	–	*Streptococcus agalactiae* (*n* = 2)	*Staphylococcus aureus* (*n* = 5)	*Staphylococcus, coagulase negative* (*n* = 5)
	–	*Klebsiella* spp. (*n* = 2)	Non-typhoidal *Salmonella* (*n* = 5)	*Klebsiella* spp*.* (*n* = 5)
	–	*Enterobacter* spp. (*n* = 2)	*Streptococcus pneumoniae* (*n* = 4)	*Streptococcus* spp*.* (*n* = 4)
Western	*Citrobacter* spp. (*n* = 3)	*Streptococcus* spp. (*n* = 2)	*Staphylococcus aureus* (*n* = 7)	*Escherichia coli* (*n* = 7)
	*Streptococcus* spp. (*n* = 2)	*Staphylococcus aureus* (*n* = 2)	*Escherichia coli* (*n* = 5)	*Staphylococcus aureus* (*n* = 5)
	*Streptococcus pneumoniae* (*n* = 2)	*Proteus* spp. (*n* = 2)	*Streptococcus* spp*.* (*n* = 4)	*Pseudomonas aeruginosa* (*n* = 5)
	*Streptococcus agalactiae* (*n* = 2)	*Klebsiella* spp. (*n* = 2)	*Staphylococcus* spp*.* (*n* = 4)	Non-typhoidal *Salmonella* (*n* = 5)
	*Staphylococcus aureus* (*n* = 2)	*Escherichia coli* (*n* = 2)	*Pseudomonas* spp*.* (*n* = 4)	*Klebsiella pneumoniae* (*n* = 5)
Northern	–	Non-typhoidal *Salmonella* (*n* = 2)	*Staphylococcus, coagulase negative* (*n* = 2)	*Escherichia coli* (*n* = 12)
	–	*Staphylococcus aureus* (*n* = 1)	*Serratia liquefaciens* (*n* = 2)	*Klebsiella pneumoniae* (*n* = 7)
	–	*Serratia* spp. (*n* = 1)	*Staphylococcus aureus* (*n* = 1)	*Staphylococcus, coagulase negative* (*n* = 6)
	–	*Pseudomonas* spp. (*n* = 1)	*Serratia marcescens* (*n* = 1)	*Staphylococcus aureus* (*n* = 6)
	–	*Klebsiella* spp. (*n* = 1)	*Pseudomonas* spp*.* (*n* = 1)	*Klebsiella* spp*.* (*n* = 6)
Southern	*Vibrio cholerae* (*n* = 1)	Typhoidal *Salmonella* (*n* = 1)	*Streptococcus pneumoniae* (*n* = 1)	*Escherichia coli* (*n* = 4)
	*Acinetobacter calcoaceticus* (*n* = 1)	*Klebsiella* spp. (*n* = 1)	*Pasteurella multocida* (*n* = 1)	*Streptococcus agalactiae* (*n* = 3)
	–	–	*Klebsiella pneumoniae* (*n* = 1*)*	*Staphylococcus aureus* (*n* = 3)
	–	–	*Enterobacter cloacae* (*n* = 1)	*Klebsiella pneumoniae* (*n* = 3)
	–	–	*–*	*Streptococcus pneumoniae* (*n* = 2)
Middle	–	–	*Streptococcus pneumoniae* (*n* = 1)	*–*
	–	–	*Streptococcus agalactiae* (*n* = 1)	*–*
	–	–	*Klebsiella pneumoniae* (*n* = 1)	*–*

No distinction has been made between case series, fever series, and seroprevalence studies. Numbers in parenthesis indicate the number of publications reporting the given microorganism

The literature on viral infections was heavily biased toward the more lethal viral haemorrhagic fevers reflecting the attention that outbreaks of these diseases attract globally. The 2014–2016 West African outbreak was associated with a spike in the number of publications on Ebola virus disease, accounting for nearly a quarter of the Ebola virus disease reports published during the period of this review. There were only five reports of Enteroviruses, specifically coxsackie B3 virus, from the whole of Africa which contrasts with multiple descriptions of Enterovirus-71 outbreaks reported in Asia [31]. Dengue fever has not received much attention in Africa compared to malaria, despite the virus being present in most regions of the continent. Before the 1980s, there were no confirmed cases of dengue virus disease in Africa [31], and the number of reports identified in this review suggests that either the disease is increasingly endemic or the pathogen increasingly sought. Accurate point-of-care tests for dengue based on combined IgM antibody and NS1 antigen detection are now available, and they could be considered for incorporation into fever case management algorithms [24]. Viral hepatitis, whose burden has increased globally over the past 30 years [36], was reported from 18 countries with the majority of the articles being from Egypt.

This review has several limitations. First, as stated, the data presented here reflect neither overall incidence nor the prevalence of the microorganisms and should not be over-interpreted. That a pathogen was not reported from a country or region cannot be taken as definitive evidence of its absence, because this review did not capture records of an organism being sought but not found. Researchers are likely to look for pathogens that have been previously recognised or reported, or in order to answer a specific research question, such as on vaccine protective efficacy for a particular pathogen, or management of outbreaks (e.g. Ebola virus). It is worth noting that while malaria was reported across the entire African continent in the early 1980s, some northern and far southern regions have now eliminated the disease; this review did not exclude malaria-free countries. Reports on the presence of newsworthy pathogens, such as Ebola virus and Zika virus, are also more likely to be published, and thus, the clinical literature is susceptible to substantial reporting biases. Under-reporting is further compounded by the fact that some patients might have taken antimicrobials prior to hospital presentation, masking some bacterial pathogens. Nucleic acid amplification tests can also lack sensitivity for some infections, such as when timing of collection is after the bacteraemia or viremia has peaked. Routine data from clinical specimens tested in hospitals and private laboratories would provide a more representative picture of the leading causes of non-malaria febrile illness, but these are published infrequently. Increased electronic laboratory and clinical data capture has the potential to transform routine disease surveillance and information sharing in the future. Restricting the search to pathogens detected in blood and CSF meant that some important or common infections such as helminths and respiratory and sexually transmitted infections were omitted. However, restriction to organisms isolated from normally sterile sites limits the potential for misinterpretation of pathogen data in terms of distinguishing colonisation from true infection.

The studies included in this review exhibited large heterogeneity of study design, patient population, and diagnostic assays used. It was not feasible to comprehensively assess the heterogeneity of case definitions, or the quality of various study elements, because of inconsistent reporting and varied laboratory and diagnostic techniques. Just over two-third of the studies included in this review were considered to be at moderate to high risk of bias (Additional file 3). This heterogeneity precludes the possibility of any meaningful amalgamation of the data and meta-analysis. However, such analysis may be pursued in the future for subsets of studies identified through the database, which is publicly available on-line [20]. Information on the origin of the infections, including whether community- or hospital-acquired, was not systematically reported in the articles. Such information is particularly important in the assessment of the burden of antimicrobial resistance, since infections acquired in health care settings are more often drug resistant. Further limitation of this review is that information regarding whether the data originated from the referral facilities—where sicker patients are treated—or from the primary care level—where most people in SSA seek care was not available. There is a need for standardisation of the design and reporting of studies of the epidemiology of febrile illness, to aid comparison through time and space.

Certain pathogens are more likely to be hospital-acquired, such as *Candida* spp. and some Gram-negative bacteria such as *Acinetobacter baumannii*. The latter was reported from all regions except Western Africa; of note, this predominantly nosocomial infection has been associated with a high case fatality risk of 41% in hospitalised patients in Thailand [37]. The Global Antimicrobial Resistance Surveillance System (GLASS) has standardised the definition of nosocomial infections, and infection source should be clearly identified in future reports [38]. This review did not systematically capture whether the seroprevalence studies reported evidence of current or very recent infection (IgM, or change in antibody titre) or past infection (IgG). Finally, information regarding the underlying antimicrobial susceptibilities of pathogens was not reported in this review. A subset of the articles included in this review provided antimicrobial resistance data, which is currently being extracted and will be reported in the future.

## Conclusions

This review provides a comprehensive summary of published reports on potential causes of non-malarial febrile illnesses in Africa. Pathogens and diseases previously under-recognised or not thought to be endemic on the continent are clearly present, including dengue virus and melioidosis. Testing and reporting of febrile disease aetiology in Africa is patchy, with probably substantial under-reporting of and under-consideration of important pathogens in many regions. Reporting of microbiology data needs to improve, including classifying bacterial infections as community- or hospital-acquired. This concern has been noted by other authors and is an impediment to current assessments of the burden of drug-resistant infections in LMICs [39]. Guidelines for reporting fever aetiology studies are vital to improve the public health value of such research. More emphasis is needed in rural areas that remain beyond the reach of many research and surveillance efforts. A mechanism such as the recently inaugurated African Centers for Disease Control and Prevention may be able to reform this area. As the threat of antimicrobial resistance looms large, knowledge of the distribution of pathogens causing febrile illness should facilitate priority setting in the development of new diagnostic tools and improved antimicrobial stewardship.

## Supplementary information

**Additional file 1.** Further results

**Additional file 2.** List of studies included

**Additional file 3.** Assessment of risk of bias

## Data Availability

All data generated and analysed in this review are openly available from the IDDO webpage as a downloadable resource: https://www.iddo.org/surveyor/NMFI/#0.

## Abbreviations

AMR: Antimicrobial resistance
BDSP: Banque de Données Santé Publique
CSF: Cerebrospinal fluid
NMFI: Non-malarial febrile illness
NTS: Non-typhoidal *Salmonella*
PCR: Polymerase chain reaction
PRISMA: Preferred Reporting Items for Systematic Reviews and Meta-Analyses
RDT: Rapid diagnostic test
RVF: Rift Valley fever
VHF: Viral haemorrhagic fevers
WHO: World Health Organization
