# Supplementary material for: Non-malarial febrile illness: a systematic review of published aetiological studies and case reports from Africa, 1980–2015
Source: BMC Med. 2020 Sep 21;18:279. doi: 10.1186/s12916-020-01744-1 (PMC7504660; doi:10.1186/s12916-020-01744-1)
Supplement: Supplementary file 1 — Additional file 1. Further results [file 12916_2020_1744_MOESM1_ESM.docx]

**Additional file 1:**

**Non-malarial febrile illness: A systematic review of published aetiological studies and case reports from Africa, 1980-2015**

Table of Contents

[Section 1: Further details on the systematic review and data extraction 4](#_Toc29302848)

[1.1 Search terminology used for the systematic review 4](#_Toc29302849)

[1.2 List of variables extracted from each of the study articles eligible for inclusion 5](#_Toc29302850)

[Section 2: data description and additional results 6](#_Toc29302851)

[2.1 Number of articles over time 6](#_Toc29302852)

[2.2 Number of articles over time by countries 7](#_Toc29302853)

[2.3 Age-distribution of the patients reported in the articles by region 8](#_Toc29302854)

[2.4 Sample source analysed 8](#_Toc29302855)

[2.5 Number of articles by diagnostic methods and pathogens 9](#_Toc29302856)

[2.6 The use of diagnostic tests over time for each pathogen group 10](#_Toc29302857)

[Section 3: aetiological findings 11](#_Toc29302858)

[3.1 Number of articles describing top ten most common bacterial infections by mode of transmission 11](#_Toc29302859)

[3.2 Number of articles describing top ten most common bacterial infections by age-group 12](#_Toc29302860)

[3.3 Number of articles describing viral infections by epidemiological mode of transmission 13](#_Toc29302861)

[3.4 Number of articles describing top ten viral infections by age-group 14](#_Toc29302862)

[3.5 Number of articles describing fungal infections by epidemiological mode of transmission 15](#_Toc29302863)

[3.6 Fungal infections by age category and epidemiological mode of transmission 16](#_Toc29302864)

[3.7 Number of articles describing parasitic infections by epidemiological mode of transmission 17](#_Toc29302865)

[3.8 Number of articles describing parasitic infections by age-group and epidemiological mode of transmission 18](#_Toc29302866)

[3.9 The proportion of the participants testing positive in fever series for bacterial infections in studies where the pathogens were detected 19](#_Toc29302867)

[3.10 The proportion of the participants testing positive in fever series for viral infections in only the studies where the pathogens were detected 20](#_Toc29302868)

[Section 4: spatio-temporal reports 21](#_Toc29302869)

[4.1 Top 10 common pathogens in **East Africa** for each pathogen group over time 21](#_Toc29302870)

[4.2 Top 10 common pathogens in **West Africa** for each pathogen group over time 22](#_Toc29302871)

[4.3 Top 10 common pathogens in **North Africa** for each pathogen group over time 23](#_Toc29302872)

[4.4 Top 10 common pathogens in **Southern Africa** for each pathogen group over time 24](#_Toc29302873)

[4.5 Top 10 common pathogens in **Central Africa** for each pathogen group over time 25](#_Toc29302874)

[Section 5: country profiles: all reported pathogens 26](#_Toc29302875)

[5.1 Algeria 26](#_Toc29302876)

[5.2 Angola 27](#_Toc29302877)

[5.3 Benin 28](#_Toc29302878)

[5.4 Botswana 29](#_Toc29302879)

[5.5 Burkina Faso 30](#_Toc29302880)

[5.6 Burundi 31](#_Toc29302881)

[5.7 Cameroon 32](#_Toc29302882)

[5.8 Central African Republic 33](#_Toc29302883)

[5.9 Chad 34](#_Toc29302884)

[5.10 Comoros 35](#_Toc29302885)

[5.11 Congo 36](#_Toc29302886)

[5.12 Cote d’ Ivore 37](#_Toc29302887)

[5.13 Djibouti 38](#_Toc29302888)

[5.14 DRC 39](#_Toc29302889)

[5.15 Egypt 40](#_Toc29302890)

[5.16 Equatorial Guinea 41](#_Toc29302891)

[5.17 Ethiopia 42](#_Toc29302892)

[5.18 Gabon 43](#_Toc29302893)

[5.19 The Gambia 44](#_Toc29302894)

[5.20 Ghana 45](#_Toc29302895)

[5.21 Guinea 46](#_Toc29302896)

[5.22 Kenya 47](#_Toc29302897)

[5.23 Liberia 48](#_Toc29302898)

[5.24 Libya 49](#_Toc29302899)

[5.25 Madagascar 50](#_Toc29302900)

[5.26 Malawi 51](#_Toc29302901)

[5.27 Mali 52](#_Toc29302902)

[5.28 Mauritania 53](#_Toc29302903)

[5.29 Mayotte 54](#_Toc29302904)

[5.30 Morocco 55](#_Toc29302905)

[5.31 Mozambique 56](#_Toc29302906)

[5.32 Namibia 57](#_Toc29302907)

[5.33 Niger 58](#_Toc29302908)

[5.34 Nigeria 59](#_Toc29302909)

[5.35 Rwanda 60](#_Toc29302910)

[5.36 Senegal 61](#_Toc29302911)

[5.37 Sierra Leone 62](#_Toc29302912)

[5.38 Somalia 63](#_Toc29302913)

[5.39 South Africa 64](#_Toc29302914)

[5.40 South Sudan 65](#_Toc29302915)

[5.41 Sudan 66](#_Toc29302916)

[5.42 Swaziland 67](#_Toc29302917)

[5.43 Tanzania 68](#_Toc29302918)

[5.44 Togo 69](#_Toc29302919)

[5.45 Tunisia 70](#_Toc29302920)

[5.46 Uganda 71](#_Toc29302921)

[5.47 Zambia 72](#_Toc29302922)

[5.48 Zimbabwe 73](#_Toc29302923)

## **Section 1: Further details on the systematic review and data extraction**

### 1.1 Search terminology used for the systematic review

| **Category** | **English (Medline) search terms** | **French (Pascal and BDSP) search terms** |
| --- | --- | --- |
| Pathogens and diseases | Babesia microti, Trypanosoma, Trypanosomiasis, Leishmania, Leishmaniasis, Influenza A Virus, H1N1 Subtype, Influenza A virus, “Influenza virus A”, encephalitis virus, Japanese, Chikungunya virus, Epstein-Barr Virus Infections, Dengue, Respiratory Syncytial Virus, Measles, Hantavirus, Hantaan Virus, Puumala Virus, Seoul Virus, Sin Nombre Virus, Hantavirus Pulmonary Syndrome, Hemorrhagic Fever with Renal Syndrome, Nipah virus, Coxsackievirus Infections, Cytomegalovirus Infections, Hepatitis, Hepatitis (a, b, c, e), Hepacivirus, Coxiella burnetii, Salmonella typhi, Salmonella paratyphi, Burkholderia pseudomallei, Pseudomonas pseudomallei, Brucella, Escherichia coli, E coli, Citrobacter freundii, Listeria monocytogenes, Leptospira (kmetyi or interogans or weilii or parva), Rickettsia, Orientia tsutsugamushi, mycobacterium tuberculosis, H1N1, Human Herpesvirus 4 or HHV-4, Ehrlichia, Anaplasma, Bartonella, Borrelia, Neorickettsia, Sennetsu, Blastomyces, Cryptococcus, or Coccidioides, Histoplasma, Penicillium marneffii, Talaromyces, Yellow Fever virus, Yersinia pestis, Francisella tularensis, tularemia, Klebsiella pneumoniae, Mycoplasma, melioidosis, brucellosis, leptospirosis, typhus abdominal, “la peste”, “yersinia pestis”, ehrlichia, “cat scratch disease”, “cat scratch fever” “trench fever”, typhus, blastomycosis, “valley fever”, coccidioides, coccidioidomycosis, histoplasma, histoplasmosis, “penicillium marneffei”, penicilliosis, talaromyces, “yellow fever virus”, “yellow fever”, tularemia | Babésiose, babésiellos, leishmania, trypanosome, chagas, piroplasmose, “maladie de chagas”, “maladie du sommeil, “Encéphalite Japonaise”, “encéphalite japonaise de type B”, virus respiratoire syncytial or VRS, rougeole, fièvre hémorragique virale”, “fièvre hémorragique, “virus Nipah“, “virus Coxsackie”, Coxsackievirus, hépatite (a or b or c or e), mélioidose, “bacille de Whitmore”, listeriose, rickettsia, “virus de l’herpes 4”, “virus de l’ebstein-barr”, bartonellose, borrélie, cryptococcose, coccidioimycose, coccidioimycose, fièvre jaune, “fièvre Q”, salmonellose, listeria, leptospirose, rickettsia, fièvre typhoïde, fièvre paratyphoïde, ‘maladie des griffes du chat”, “lymphoréticulose bénigne d’inoculation”, ‘lymphogranulome bénin”, anaplasmose, “fièvre des tranchées”, “fièvre de wolhynie”, “fièvre quintane”, “carrion disease”, “maladie de carrion”, borreliose, bartonellose, “fièvre récurrente mondiale” or FRM, blastomycose, crytococcose, coccidioidomycose, “fièvre de la vallée de San Joaquin”, fièvre de la vallée de Californie”, “fièvre du desert”, coccidioimycose, histoplasmose, penicilliose, “virus de la fièvre jaune”, “bactérie à gram positif”, “gram positif”, “bactéries à Gram négatif”, “gram négatif”, “infection `a gram négatif”, “infection à gram positif” |
| Countries | Africa, Burundi, Comoros, Djibouti, Eritrea, Ethiopia, Kenya, Madagascar, Malawi, Mozambique, Rwanda, Somalia, Tanzania, Uganda, Zambia, Zimbabwe, Seychelles, Angola, Cameroon, “Central African Republic”, Chad, Congo, “Equatorial Africa”, “Sao Tomé-et-Principe”, Principe, Gabon, Algeria, Egypt, Morocco, “South Sudan”, Sudan, Tunisia, “Western Sahara”, “Sahara Occidental”, Botswana, Lesotho, Namibia, “South Africa”, Swaziland, Benin, “Burkina Faso” or “Cape Verde”, “Ivory Coast”, Gambia, Ghana or Guinea, “Guinea-Bissau”, Liberia, Mali, Mauritania, Nigeria, Niger, Senegal, “Sierra Leone”, Togo | Afrique, Burundi, Comoros, Djibouti, Erythrée, Ethiopie, Kenya, Madagascar, Malawi, Mozambique, Rwanda, Somalie, Tanzanie, Uganda, Zambie, Zimbabwe, Seychelles, Angola, Cameroun, “République centrafricaine”, Chad, Congo, “Afrique Équatoriale”, “Sao Tomé-et-Principe”, Principe, Gabon, Algeria, Egypt, Maroc, “Soudan du Sud”, Soudan, Tunisie, “Western Sahara”, “Sahara Occidental”, Botswana, Lesotho, Namibie, “South Africa”, “Afrique du Sud”, Swaziland, Bénin, “Burkina Faso”, “Cap-Vert”, “Côte d’Ivoire”, Gambie, Ghana, Guinée, “Guinée-Bissau”, Liberia, Mali, Mauritanie, Nigeria, Niger, Senegal, “Sierra Leone”, Togo |
| Other | Anti-bacterial agents. bacteremia, bacterial infections, blood-borne pathogens, sepsis, fever, cerebrospinal fluid | Bactériémie, septicemia, “intoxication du sang”, “infection du sang” , “infection hématogène”, “pathogène sanguine”, septicémie sanguine”, “infection csanguine”, fébrile or fièvre or fièvres or pyrexie or pyrétique or pyrexies or hyperthermia, hyperthermique, temperatures élevées, “liquide cérébro-spinal” or LCS, “liquide céphalo-rachidien” or LCR |

### 1.2 List of variables extracted from each of the study articles eligible for inclusion

| **Study details** |
| --- |
| - First and second authors |
| - Title |
| - Year |
| - Abstract |
| - Uniform resource locator (URL) link to the publication |
| - PubMed ID |
| - Journal |
| **Study design** |
| - Study start and end years |
| - Total patients tested |
| - Total patients positive |
| - Patient age range (in years and months) |
| - Age group |
| - Study group |
| - Study type* |
| - Sample source |
| - Laboratory method |
| **Study site** |
| - Site name |
| - Province |
| - Country |
| - Latitude |
| - Longitude |
| **Organism incidence** |
| - Total samples tested |
| - Total samples positive for each organism |
| - Organism name |
| - Clinical disorder |

* Studies were categorised separately as: **(i) case series,** where only the positive results were reported with no information on the underlying denominator; **(ii) fever series**, where the total population denominator who were tested were given, and **(iii) seroprevalence study**, where serum samples were tested against a panel of viruses or bacteria simultaneously.

## **Section 2: data description and additional results**

### 2.1 Number of articles over time


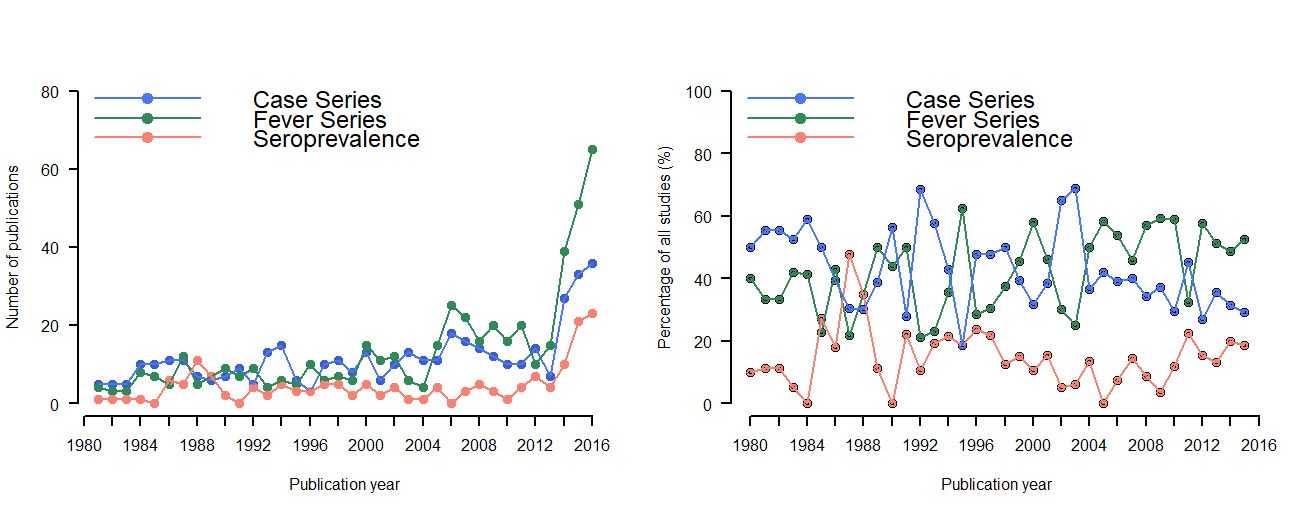


Legend: Number of articles published over time, a systematic review of published aetiological studies and case reports from Africa, 1980-2015

### 2.2 Number of articles over time by countries


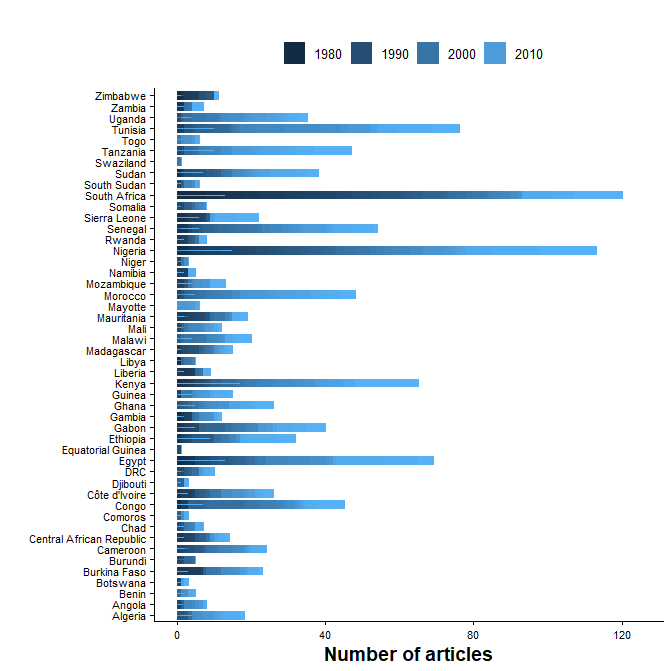


Legend: Number of articles published over time, a systematic review of published aetiological studies and case reports from Africa, 1980-2015

### 2.3 Age-distribution of the patients reported in the articles by region

| Age group | East Africa ^a^ | West Africa ^a^ | North Africa ^a^ | Southern Africa ^a^ | Central ^a^ | Multiple ^a^ | Total |
| --- | --- | --- | --- | --- | --- | --- | --- |
| Neonates | 15 (5.7%) | 20 (6.7%) | 19 (8%) | 10 (8%) | (0%) | (0%) | 64 (6%) |
| Infants ^b^ | 4 (1.5%) | 2 (0.7%) | 3 (1.3%) | 3 (2.4%) | (0%) | (0%) | 12 (1.1%) |
| Children | 39 (14.9%) | 37 (12.5%) | 35 (14.7%) | 22 (17.6%) | 11 (9%) | 2 (9.5%) | 146 (13.7%) |
| Adults | 48 (18.3%) | 34 (11.4%) | 74 (31.1%) | 42 (33.6%) | 23 (18.9%) | 3 (14.3%) | 224 (21%) |
| All ages ^c^ | 104 (39.7%) | 126 (42.4%) | 71 (29.8%) | 34 (27.2%) | 45 (36.9%) | 6 (28.6%) | 386 (36.2%) |
| Age unspecified | 52 (19.8%) | 78 (26.3%) | 36 (15.1%) | 14 (11.2%) | 43 (35.2%) | 10 (47.6%) | 233 (21.9%) |
| Total | 262 | 297 | 238 | 125 | 122 | 21 | 1065 |

^a^ Number of articles are shown in the cells with values in parenthesis representing column percentages

^b^ One study described population aged < 3 months (127 patients) were classified as Infants as it couldn’t be ascertained if any neonates were enrolled

^c^ Studies which described patients aged <18 years old were included in this category

### 2.4 Sample source analysed

| Sample used for detecting pathogens | Number of articles | Percentage |
| --- | --- | --- |
| Blood | 886 | 83.2% |
| CSF | 79 | 7.4% |
| CSF/Culture | 40 | 3.8% |
| Bone Marrow | 11 | 1.0% |
| Joint | 2 | 0.2% |
| Liver | 0 | 0.0% |
| Other | 3 | 0.3% |
| Multiple | 44 | 4.1% |

### 2.5 Number of articles by diagnostic methods and pathogens

| Diagnostic method | Bacteria | Viruses | Fungi | Parasites | Poly pathogenic |
| --- | --- | --- | --- | --- | --- |
| Culture | 428 (75.9%) | 6 (1.6%) | 8 (88.9%) | 20 (42.6%) | 54 (76.1%) |
| Microscopy/staining | 9 (1.6%) | (0%) | (0%) | 3 (6.4%) | (0%) |
| PCR | 16 (2.8%) | 70 (18.7%) | (0%) | 5 (10.6%) | 2 (2.8%) |
| Serological | 111 (19.7%) | 297 (79.4%) | 1 (11.1%) | 19 (40.4%) | 15 (21.1%) |
| Serological or PCR | 0 (0%) | 1 (0.3%) | (0%) | (0%) | (0%) |
| Total number of articles | 564 | 374 | 9 | 47 | 71 |

Percentages in parenthesis are column percentages

### 2.6 The use of diagnostic tests over time for each pathogen group


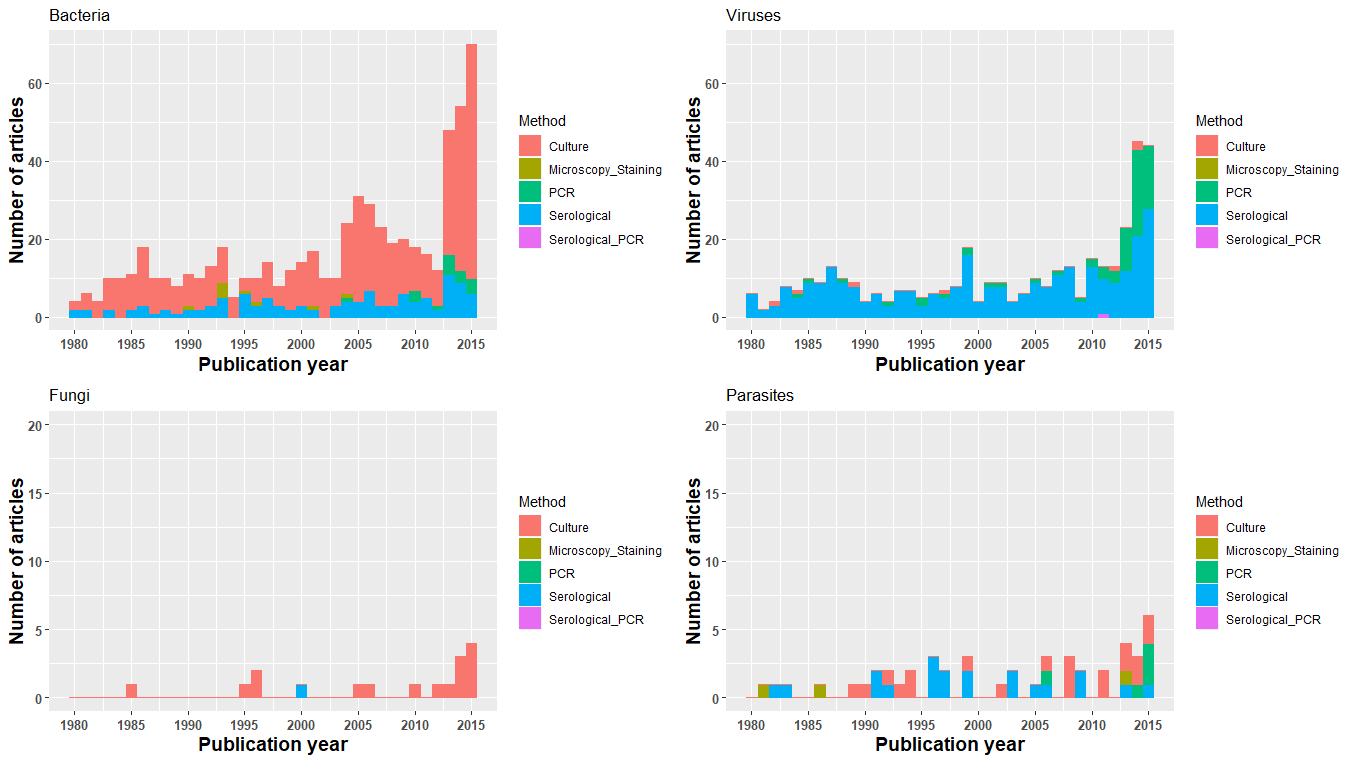


Legend: Number of articles over time by diagnostic methods, a systematic review of published aetiological studies and case reports from Africa, 1980-2015

## Section 3: aetiological findings

### 3.1 Number of articles describing top ten most common bacterial infections by mode of transmission

**
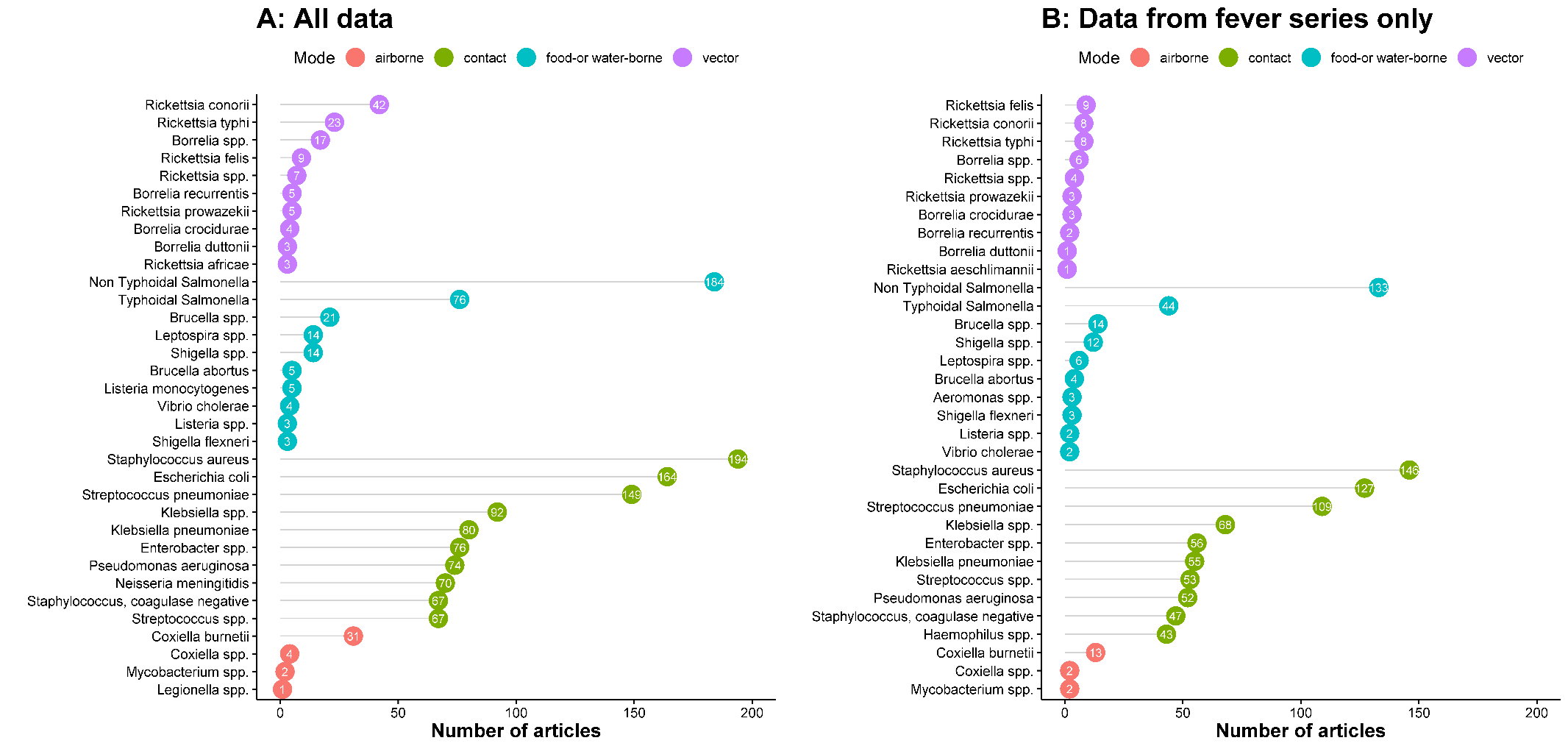
**

**Legend**: Most commonly reported bacterial infections by mode of transmission, a systematic review of published aetiological studies and case reports from Africa, 1980-2015. The left panel includes data from all the study types (case series, fever series and seroprevalence studies). The right panel is restricted to the fever series data. The graph presents the top 10 pathogens (based on the number of the published articles) by epidemiological mode of transmission.

### 3.2 Number of articles describing top ten most common bacterial infections by age-group


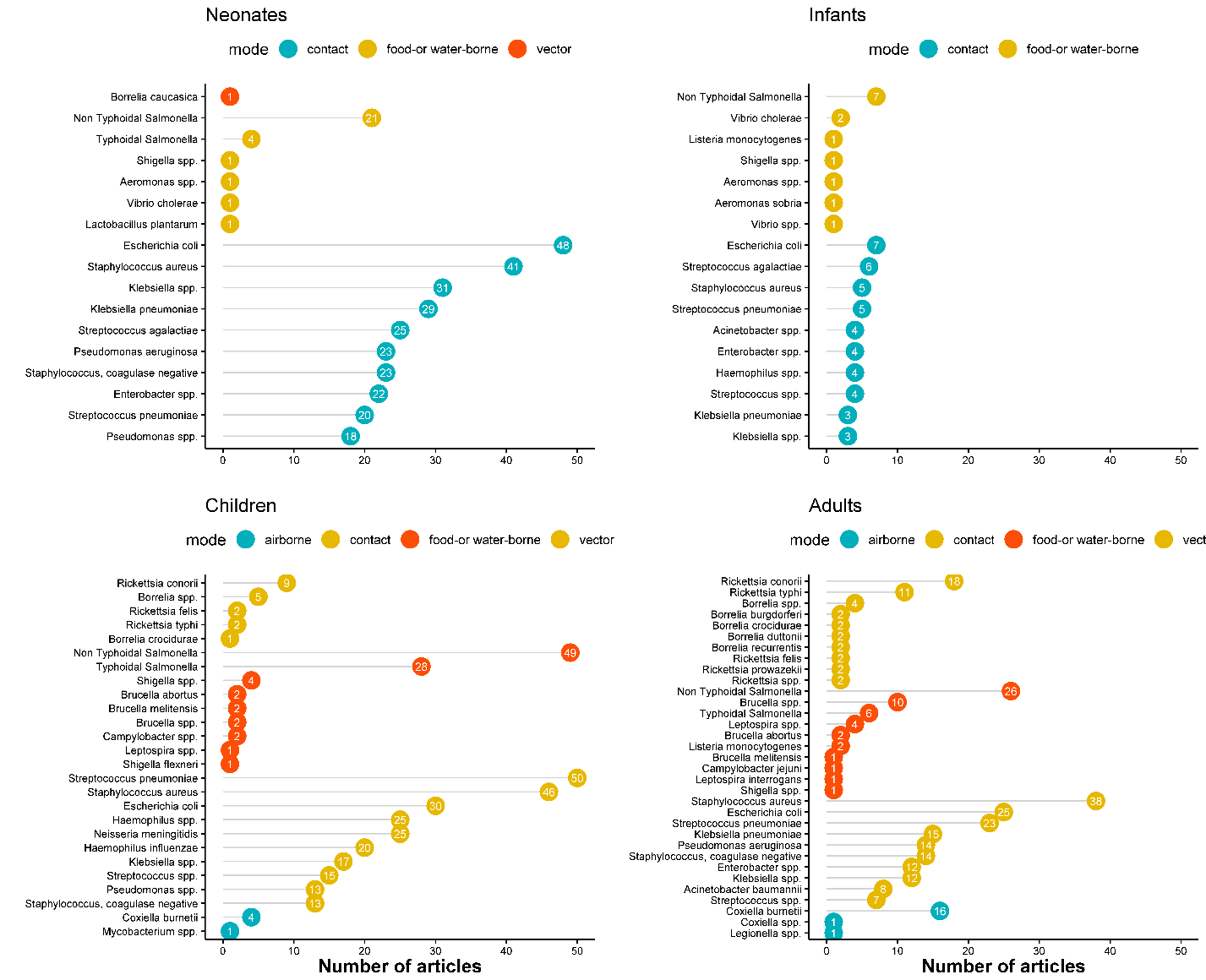


**Legend**: Most commonly reported bacterial infections by mode of transmission, a systematic review of published aetiological studies and case reports from Africa, 1980-2015. No distinction has been made between case series, fever series or seroprevalence studies. The number inside the dot plot shows the number of articles.

### 3.3 Number of articles describing viral infections by epidemiological mode of transmission

**
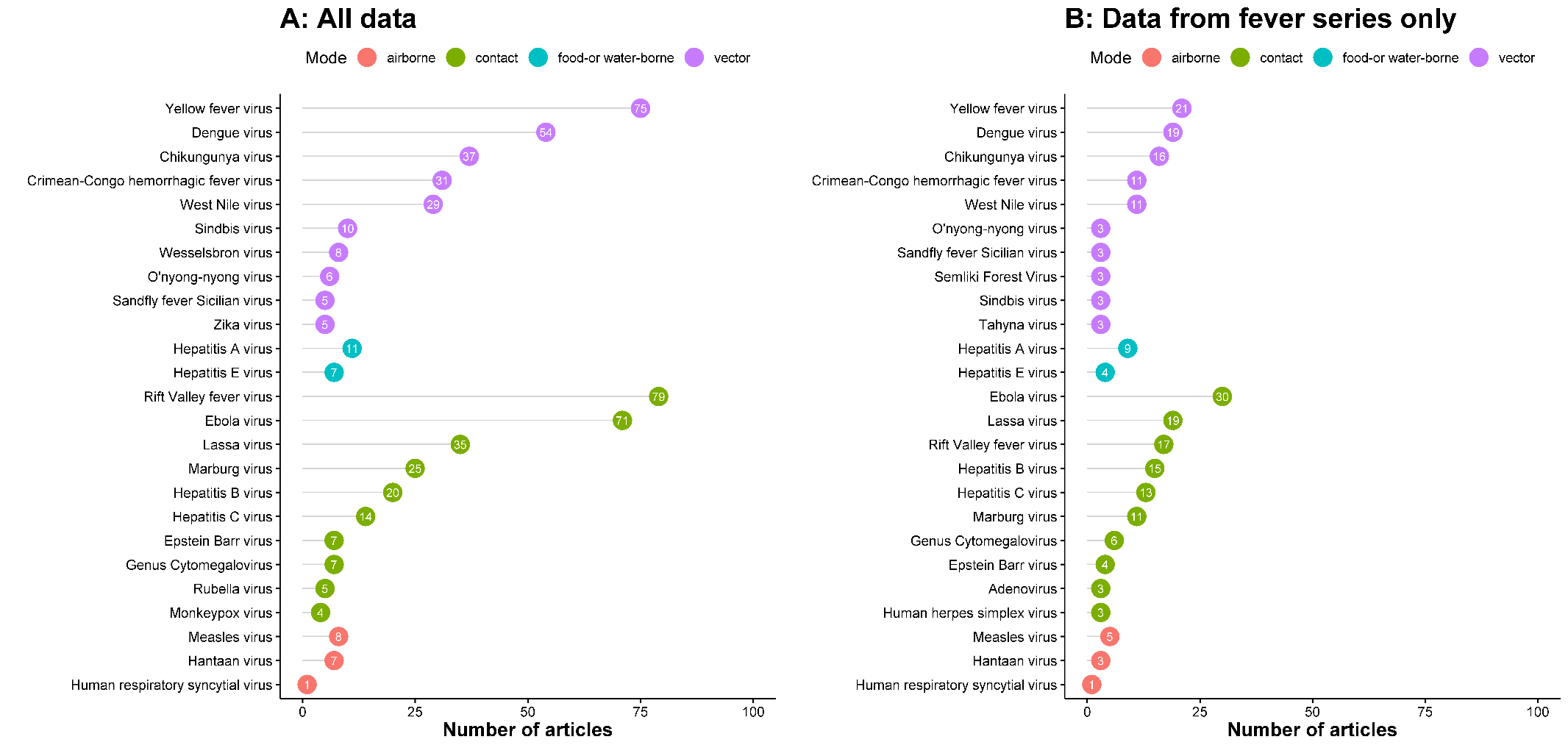
**

**Legend**: Most commonly reported viral infections by mode of transmission, a systematic review of published aetiological studies and case reports from Africa, 1980-2015. The left panel includes data from all the study types (case series, fever series and seroprevalence studies). The right panel is restricted to the fever series data. The graph presents the top 10 pathogens (based on the number of the published articles) by epidemiological mode of transmission.

### 3.4 Number of articles describing top ten viral infections by age-group


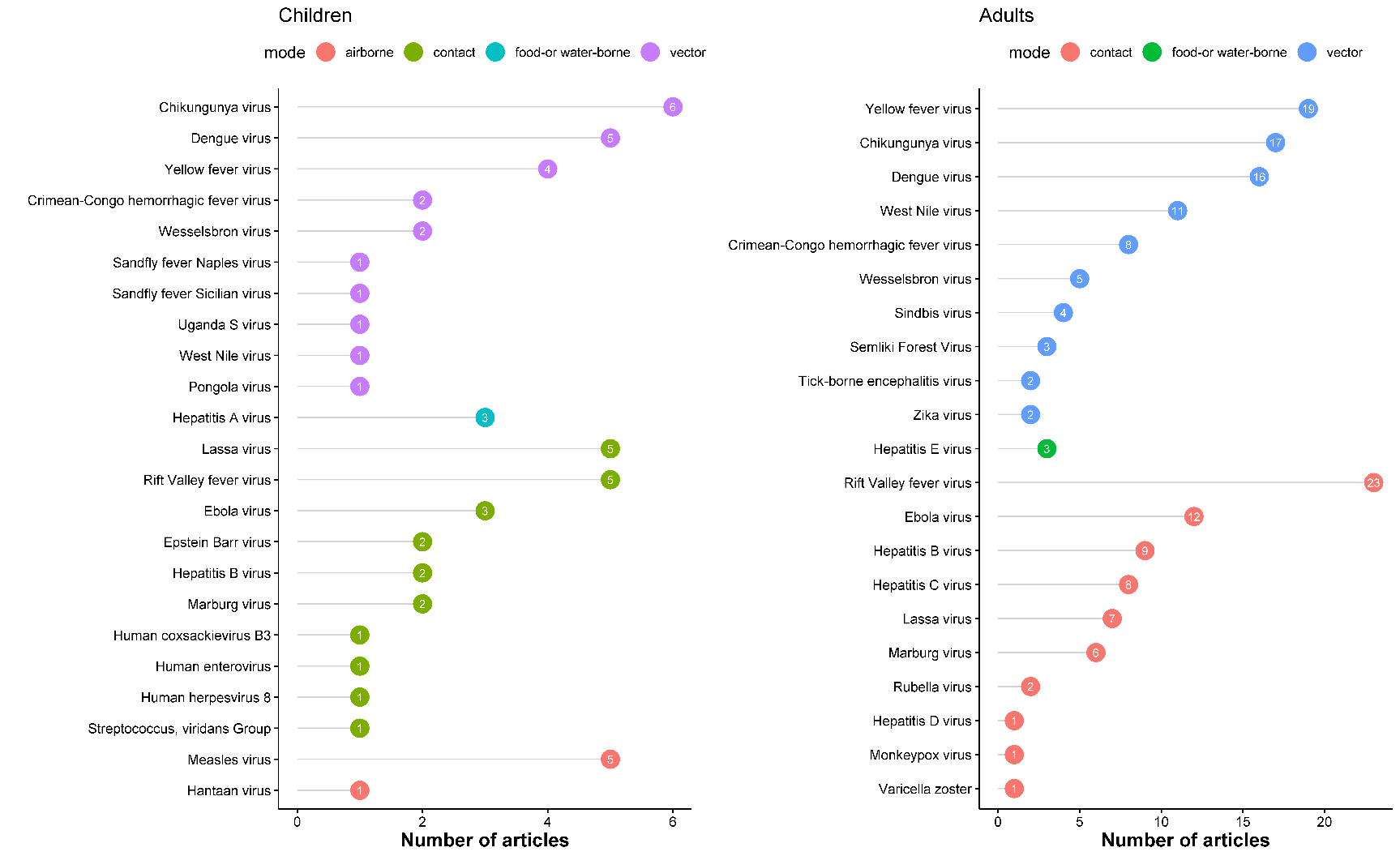


**Legend**: Most commonly reported viral infections by mode of transmission, a systematic review of published aetiological studies and case reports from Africa, 1980-2015. No distinction has been made between case series, fever series or seroprevalence studies. The number inside the dot plot shows the number of articles. There were no reports of viral infections on infants while only three articles reported viral infections among neonates (cytomegalovirus, enterovirus, and human herpes simplex virus).

### 3.5 Number of articles describing fungal infections by epidemiological mode of transmission


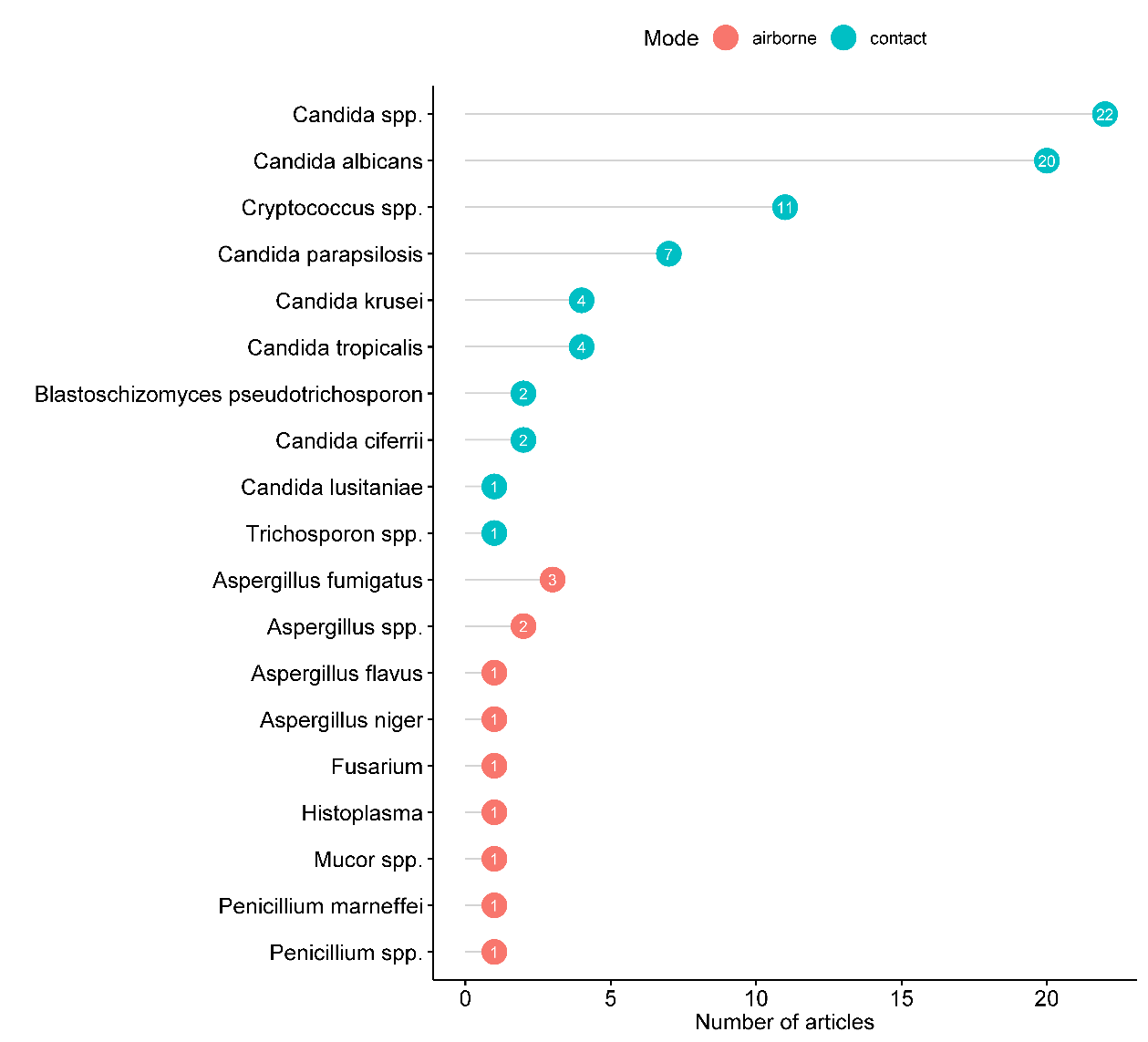


**Legend**: All reported fungal infections by mode of transmission, a systematic review of published aetiological studies and case reports from Africa, 1980-2015. No distinction has been made between case series, fever series or seroprevalence studies. The number inside the dot plot shows the number of articles.

### 3.6 Fungal infections by age category and epidemiological mode of transmission


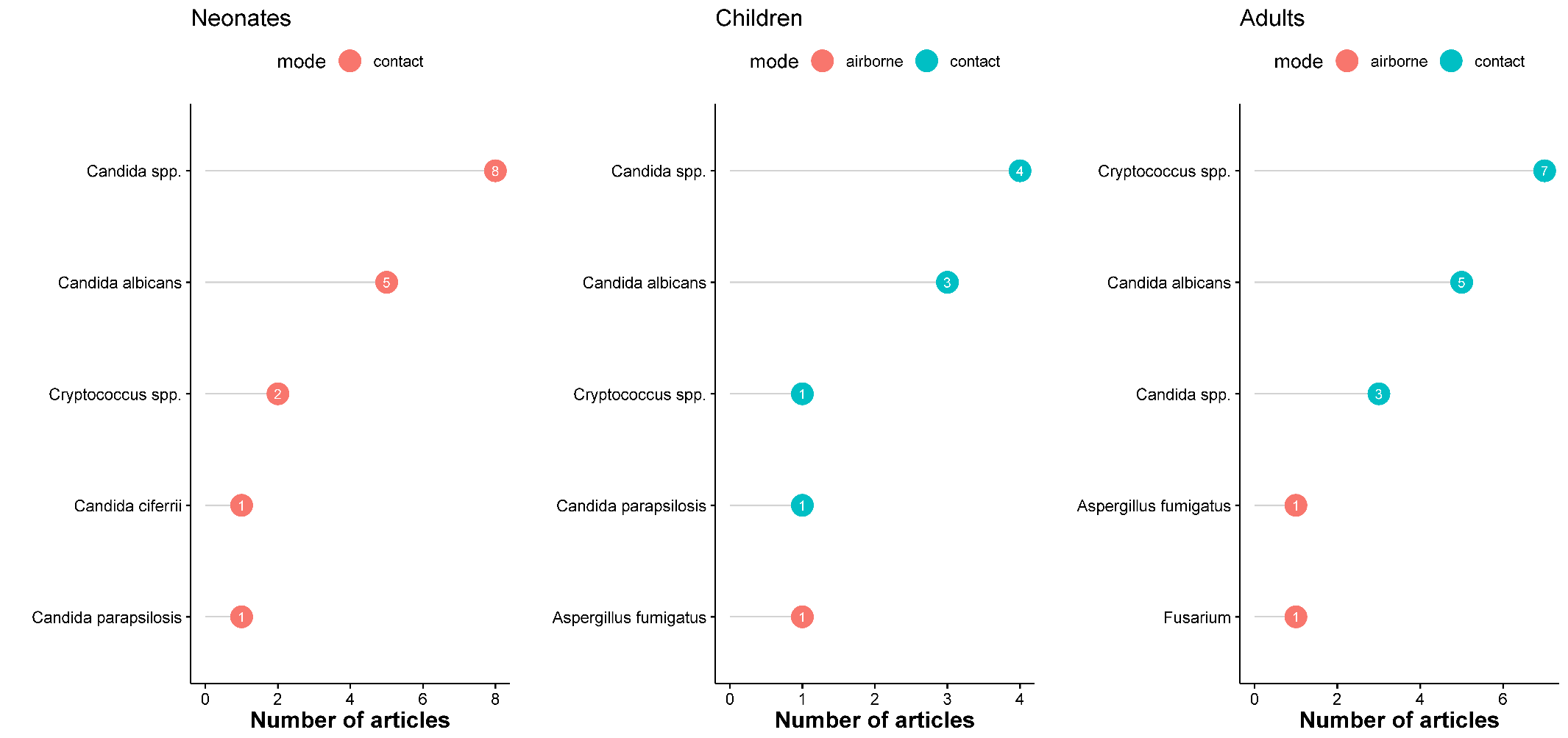


**Legend**: All reported fungal infections by mode of transmission, a systematic review of published aetiological studies and case reports from Africa, 1980-2015. No distinction has been made between case series, fever series or seroprevalence studies. The number inside the dot plot shows the number of articles.

### 3.7 Number of articles describing parasitic infections by epidemiological mode of transmission


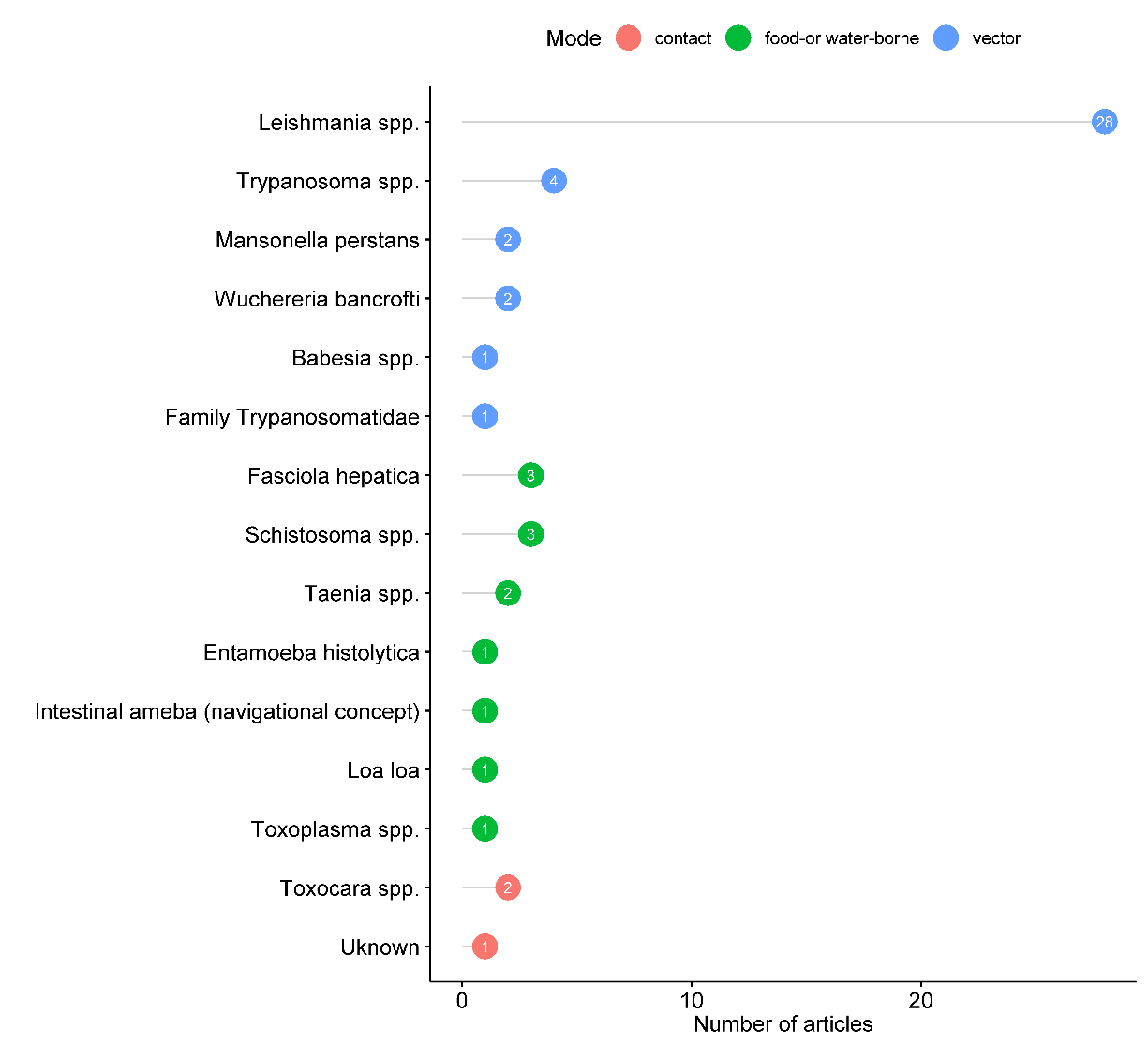


**Legend**: All reported parasitic infections by mode of transmission, a systematic review of published aetiological studies and case reports from Africa, 1980-2015. No distinction has been made between case series, fever series or seroprevalence studies. The number inside the dot plot shows the number of articles.

### 3.8 Number of articles describing parasitic infections by age-group and epidemiological mode of transmission


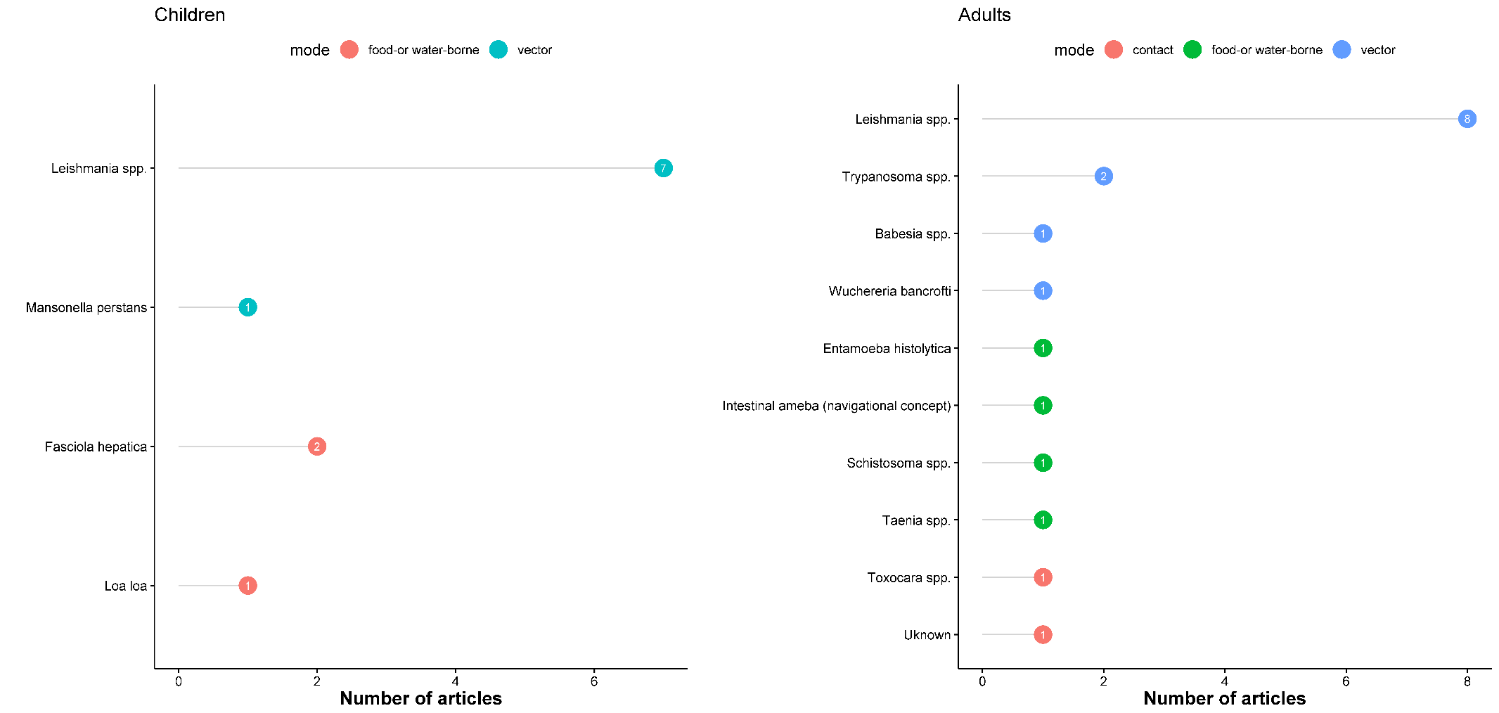


**Legend**: All reported parasitic infections by mode of transmission, a systematic review of published aetiological studies and case reports from Africa, 1980-2015. No distinction has been made between case series, fever series or seroprevalence studies. The number inside the dot plot shows the number of articles.

### 3.9 The proportion of the participants testing positive in fever series for bacterial infections in studies where the pathogens were detected


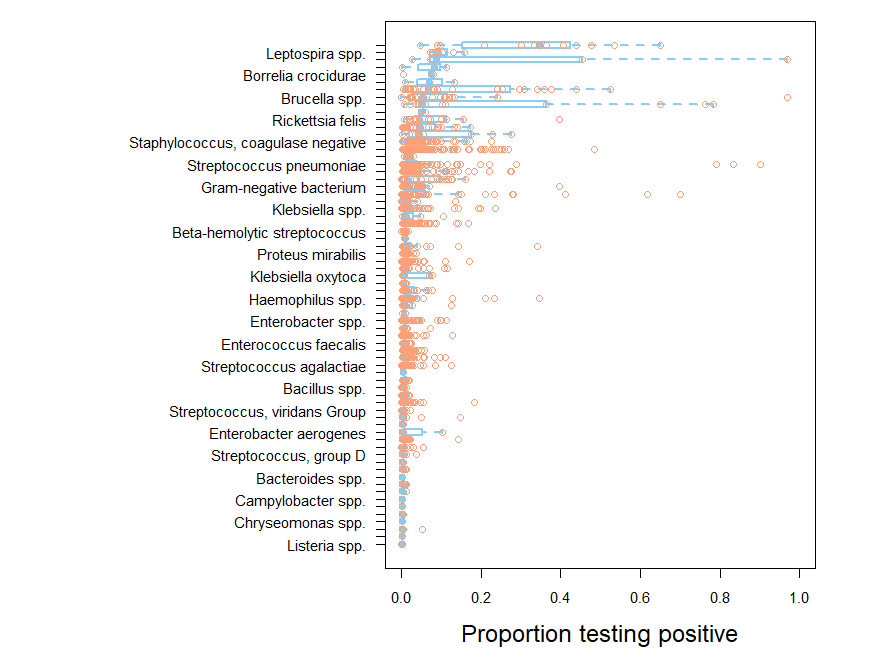


**Legend**: The boxplot shows the distribution of patients testing positive for the given bacterium, which were reported, in at least 5 articles AND with sample size ≥ 50 participants tested. **The median or any statistic should not be interpreted as incidence/prevalence of the bacterium as information regarding the number of pathogens that were tested but were negative were not captured.**

### 3.10 The proportion of the participants testing positive in fever series for viral infections in only the studies where the pathogens were detected


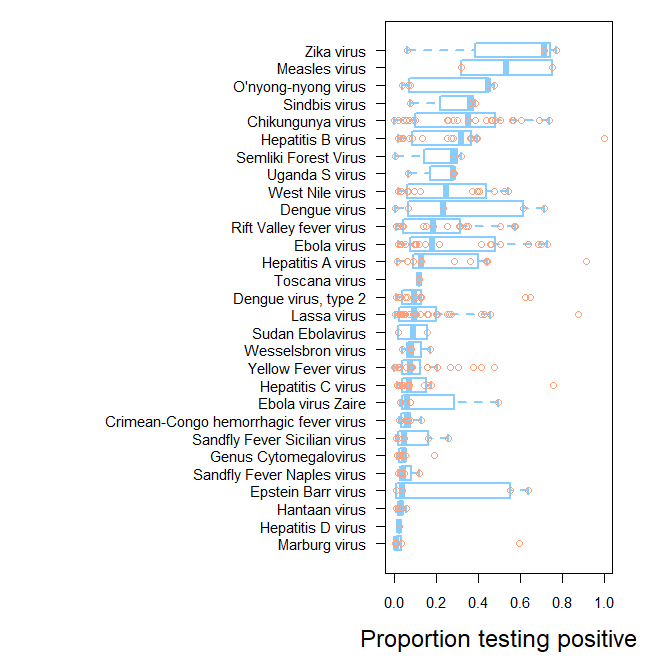


**Legend**: The boxplot shows the distribution of patients testing positive for the given viruses, which were reported, in at least 5 articles AND with sample size ≥ 50 participants tested. **The median or any statistic should not be interpreted as incidence/prevalence of the viruses as information regarding the number of pathogens that were tested but were negative were not captured.**

## **Section 4: spatio-temporal reports**

### 4.1 Top 10 common pathogens in **East Africa** for each pathogen group over time


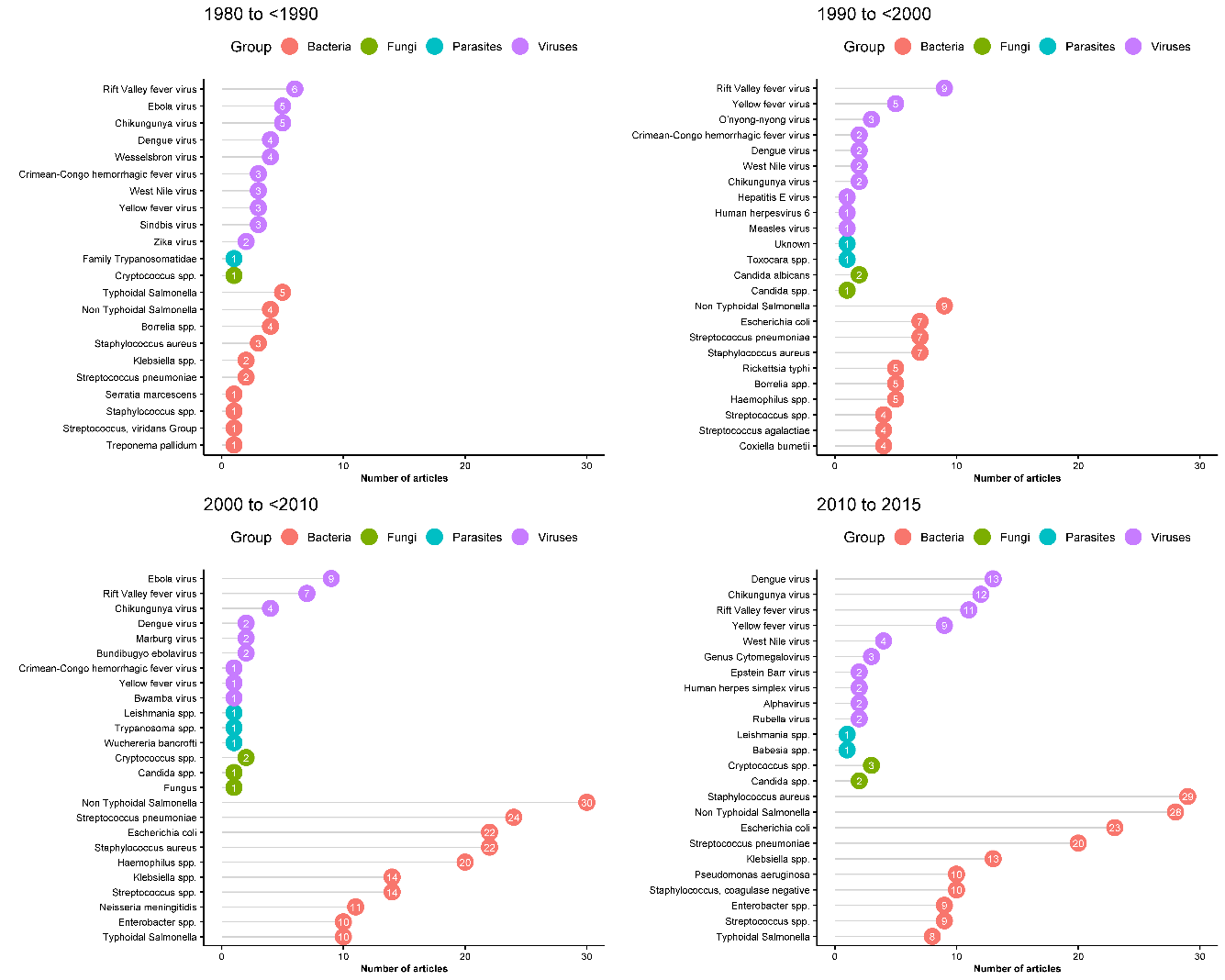


**Legend**: Most commonly reported pathogens by mode of transmission in East Africa, a systematic review of published aetiological studies and case reports from Africa, 1980-2015. No distinction has been made between case series, fever series or seroprevalence studies. The number inside the dot plot shows the number of articles.

### 4.2 Top 10 common pathogens in **West Africa** for each pathogen group over time


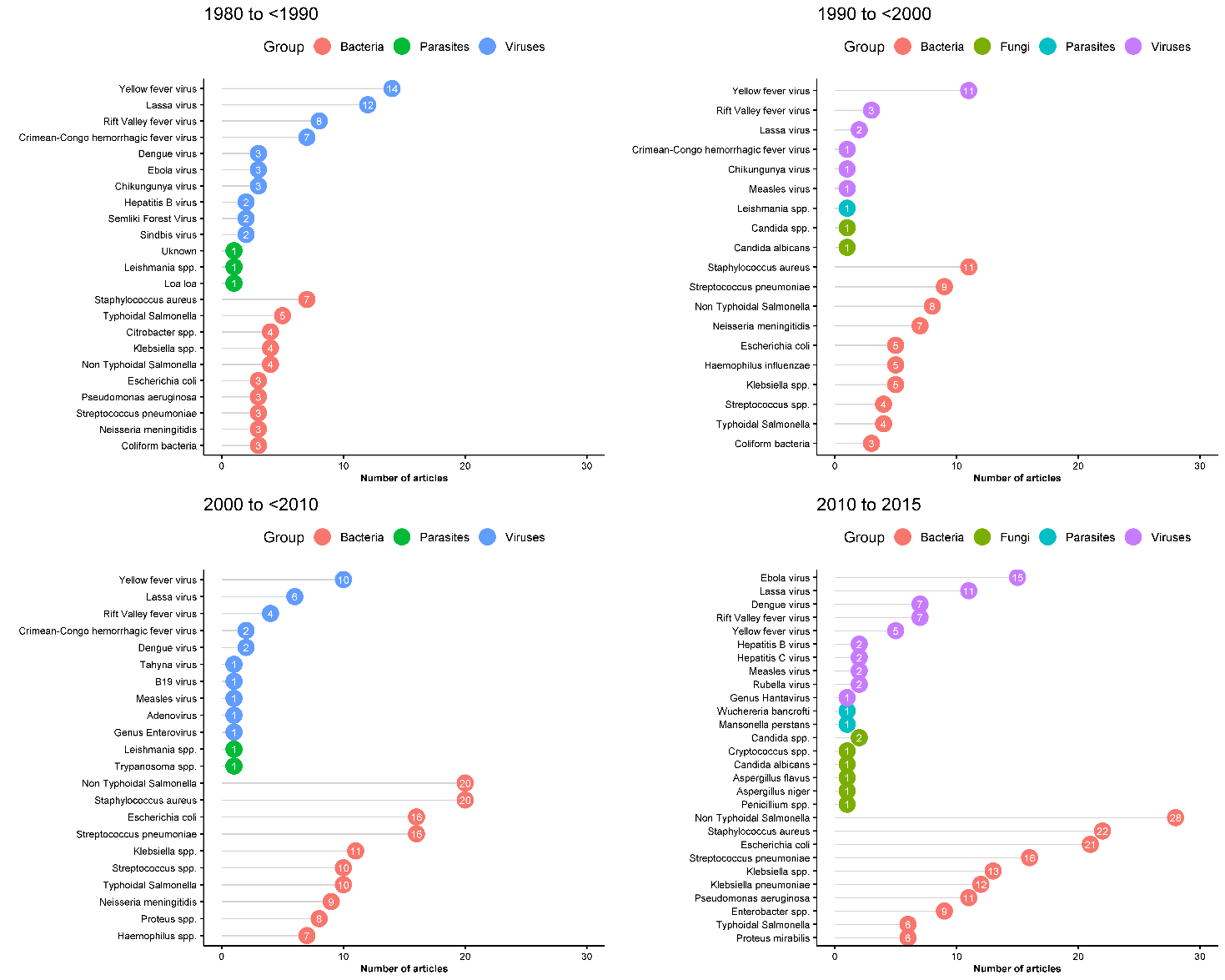


**Legend**: Most commonly reported pathogens by mode of transmission in West Africa, a systematic review of published aetiological studies and case reports from Africa, 1980-2015. No distinction has been made between case series, fever series or seroprevalence studies. The number inside the dot plot shows the number of articles.

### 4.3 Top 10 common pathogens in **North Africa** for each pathogen group over time


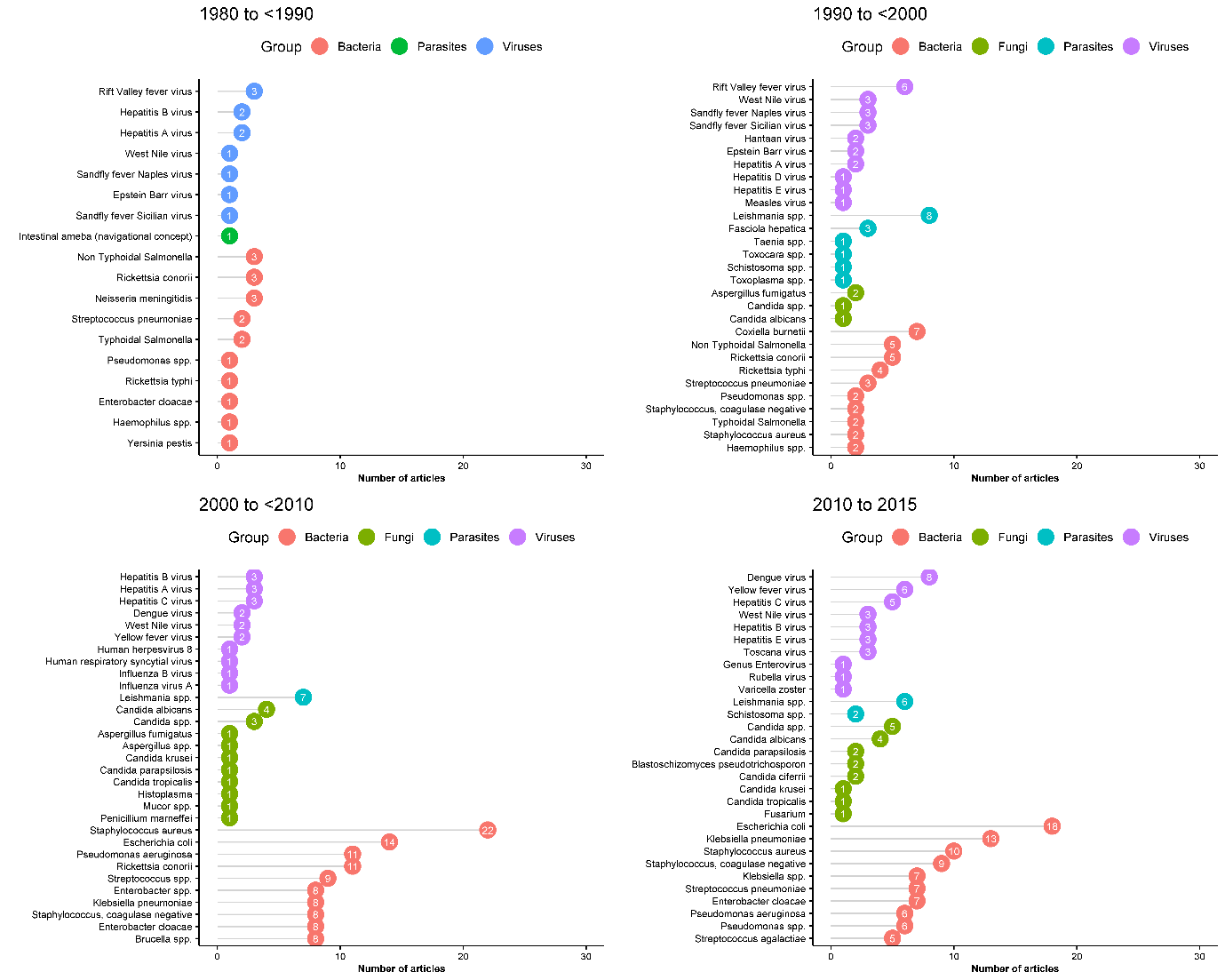


**Legend**: Most commonly reported pathogens by mode of transmission in Northern Africa, a systematic review of published aetiological studies and case reports from Africa, 1980-2015. No distinction has been made between case series, fever series or seroprevalence studies. The number inside the dot plot shows the number of articles.

### 4.4 Top 10 common pathogens in **Southern Africa** for each pathogen group over time


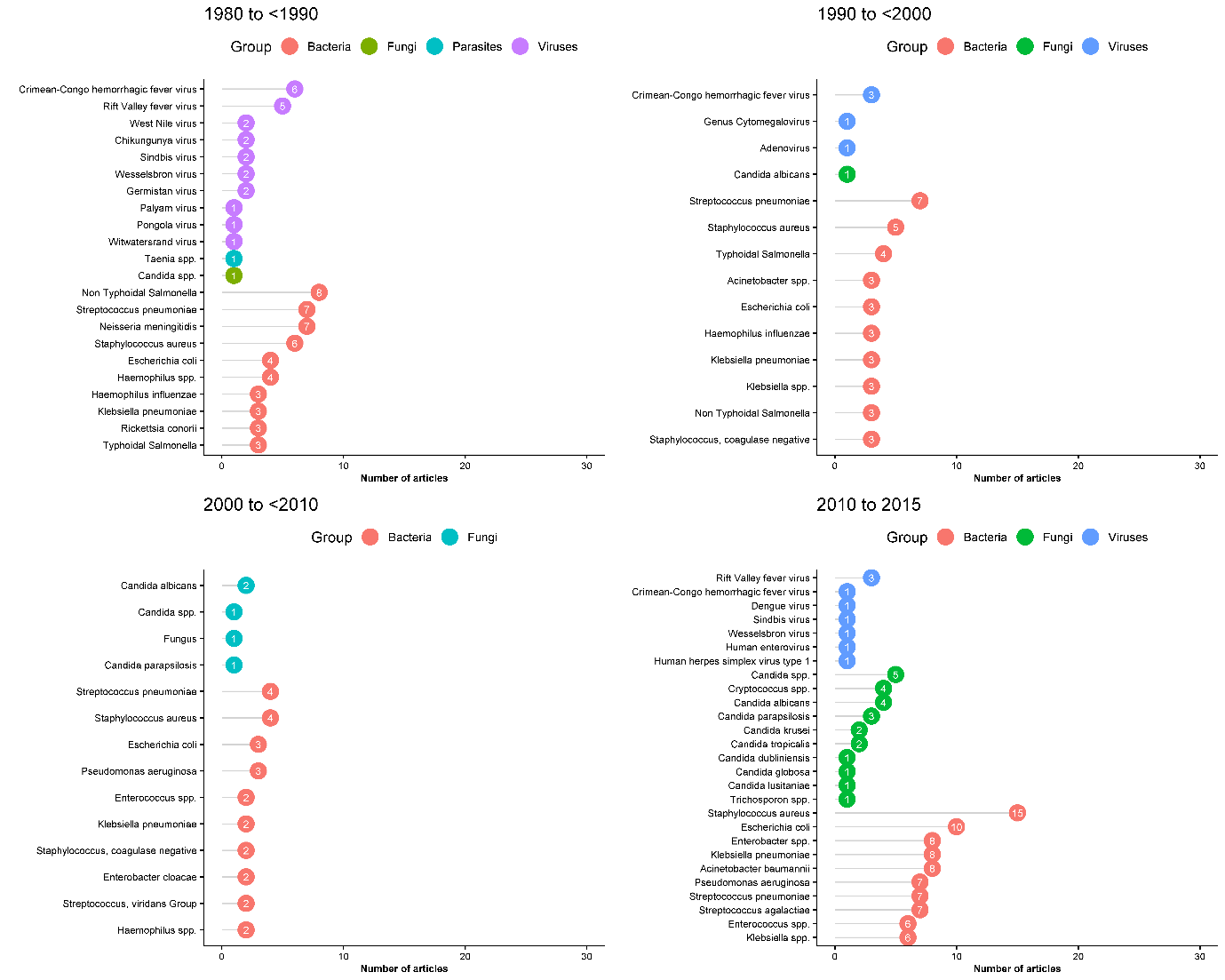


**Legend**: Most commonly reported pathogens by mode of transmission in Southern Africa, a systematic review of published aetiological studies and case reports from Africa, 1980-2015. No distinction has been made between case series, fever series or seroprevalence studies. The number inside the dot plot shows the number of articles.

### 4.5 Top 10 common pathogens in **Central Africa** for each pathogen group over time


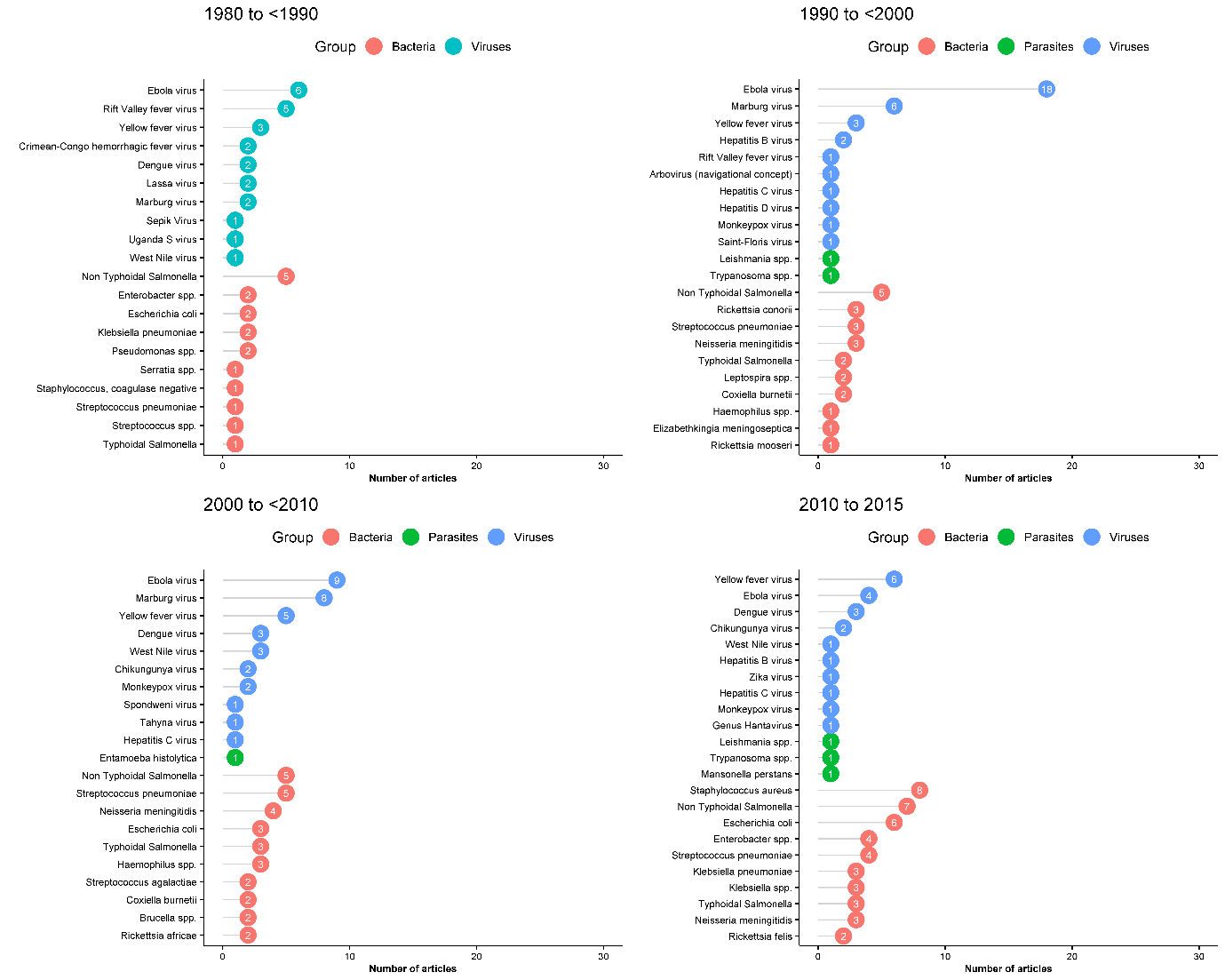


**Legend**: Most commonly reported pathogens by mode of transmission in Central Africa, a systematic review of published aetiological studies and case reports from Africa, 1980-2015. No distinction has been made between case series, fever series or seroprevalence studies. The number inside the dot plot shows the number of articles.

## **Section 5: country profiles: all reported pathogens**

### 5.1 Algeria


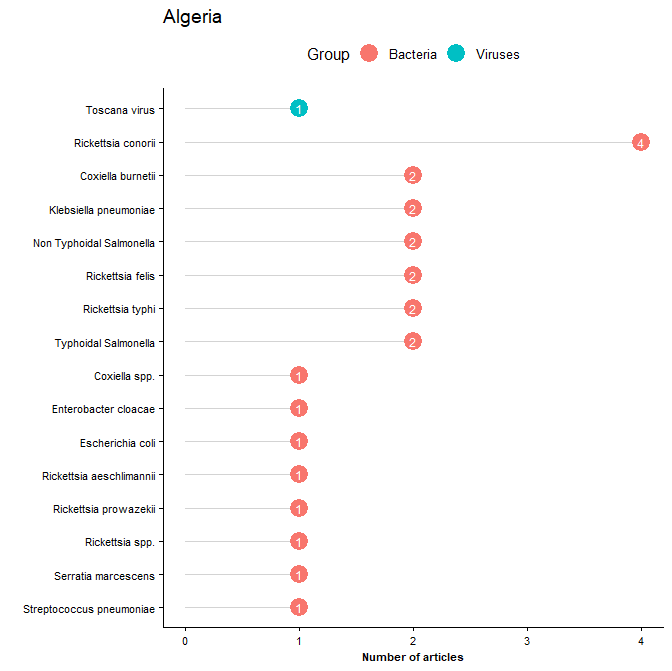


Legend: All reported pathogens by mode of transmission in Algeria, a systematic review of published aetiological studies and case reports from Africa, 1980-2015. No distinction has been made between case series, fever series or seroprevalence studies. The number inside the dot plot shows the number of articles.

### 5.2 Angola


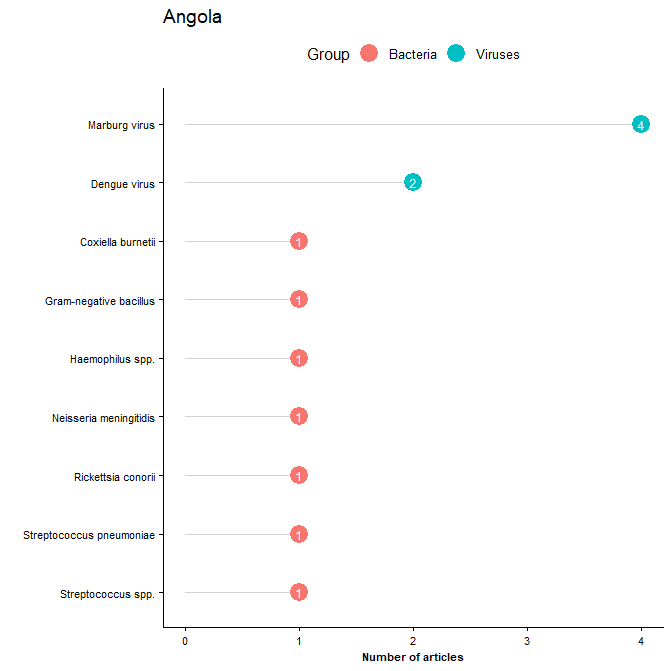


Legend: All reported pathogens by mode of transmission in Angola, a systematic review of published aetiological studies and case reports from Africa, 1980-2015. No distinction has been made between case series, fever series or seroprevalence studies. The number inside the dot plot shows the number of articles.

### 5.3 Benin


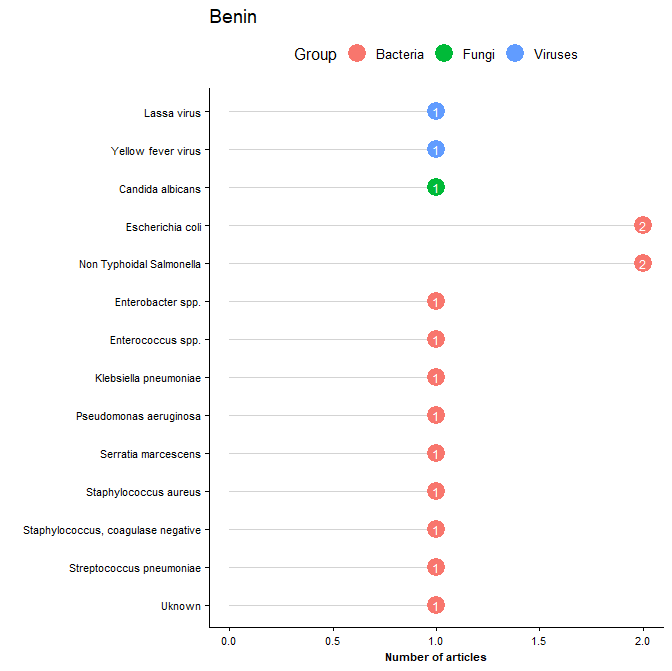


Legend: All reported pathogens by mode of transmission, a systematic review of published aetiological studies and case reports from Africa, 1980-2015. No distinction has been made between case series, fever series or seroprevalence studies. The number inside the dot plot shows the number of articles.

### 5.4 Botswana


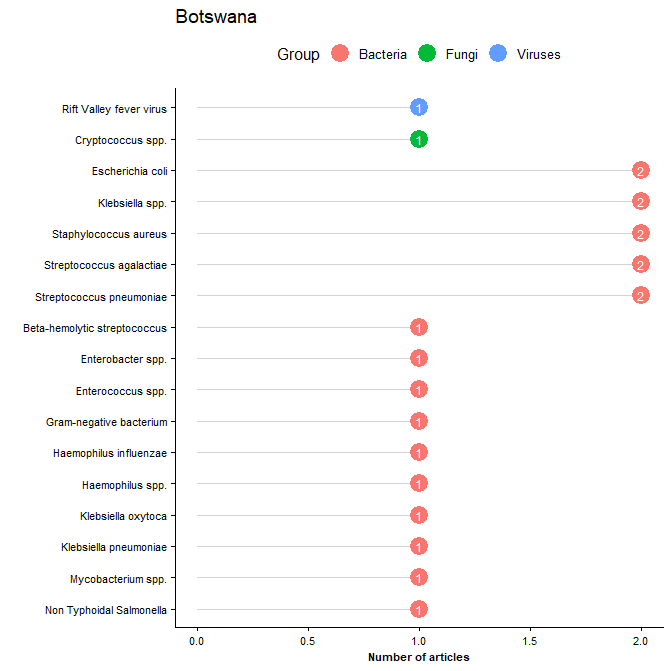


Legend: All reported pathogens by mode of transmission, a systematic review of published aetiological studies and case reports from Africa, 1980-2015. No distinction has been made between case series, fever series or seroprevalence studies. The number inside the dot plot shows the number of articles.

### 5.5 Burkina Faso


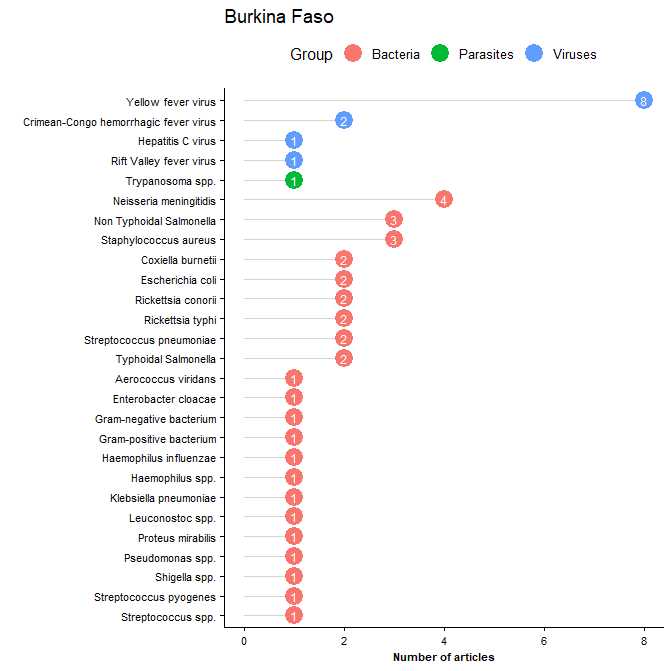


Legend: All reported pathogens by mode of transmission, a systematic review of published aetiological studies and case reports from Africa, 1980-2015. No distinction has been made between case series, fever series or seroprevalence studies. The number inside the dot plot shows the number of articles.

### 5.6 Burundi


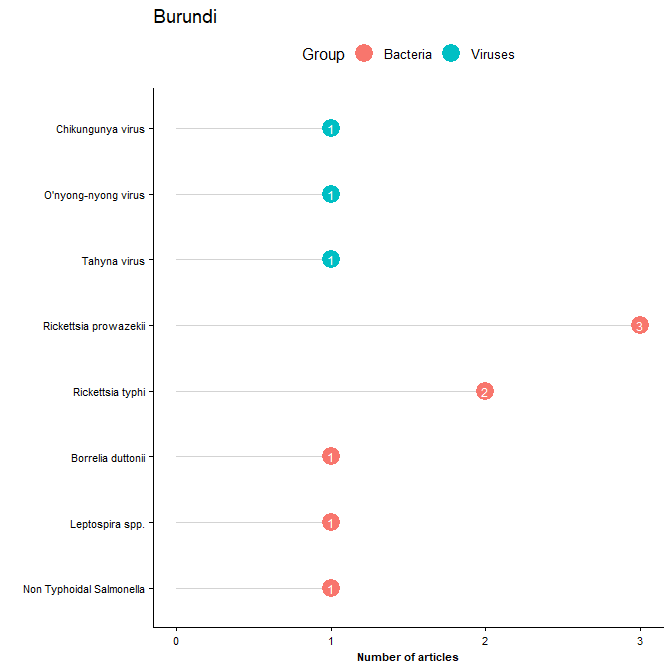


Legend: All reported pathogens by mode of transmission, a systematic review of published aetiological studies and case reports from Africa, 1980-2015. No distinction has been made between case series, fever series or seroprevalence studies. The number inside the dot plot shows the number of articles.

### 5.7 Cameroon


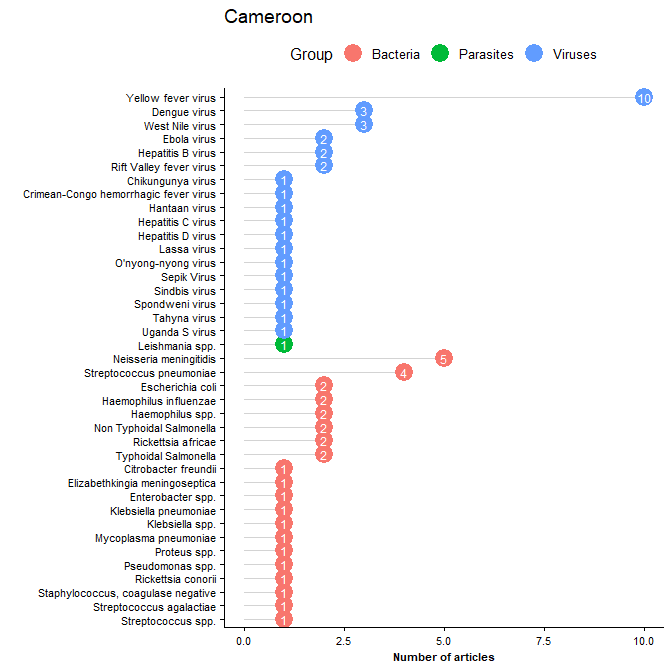


Legend: All reported pathogens by mode of transmission, a systematic review of published aetiological studies and case reports from Africa, 1980-2015. No distinction has been made between case series, fever series or seroprevalence studies. The number inside the dot plot shows the number of articles.

### 5.8 Central African Republic


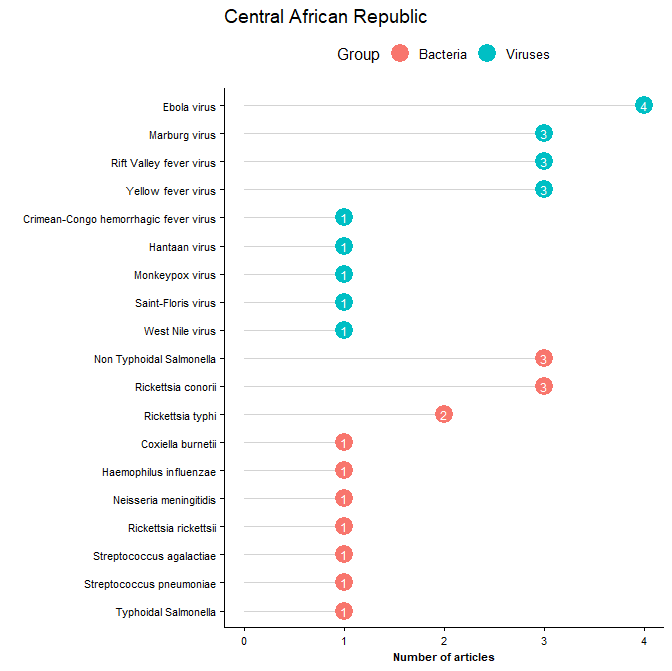


Legend: All reported pathogens by mode of transmission, a systematic review of published aetiological studies and case reports from Africa, 1980-2015. No distinction has been made between case series, fever series or seroprevalence studies. The number inside the dot plot shows the number of articles.

### 5.9 Chad


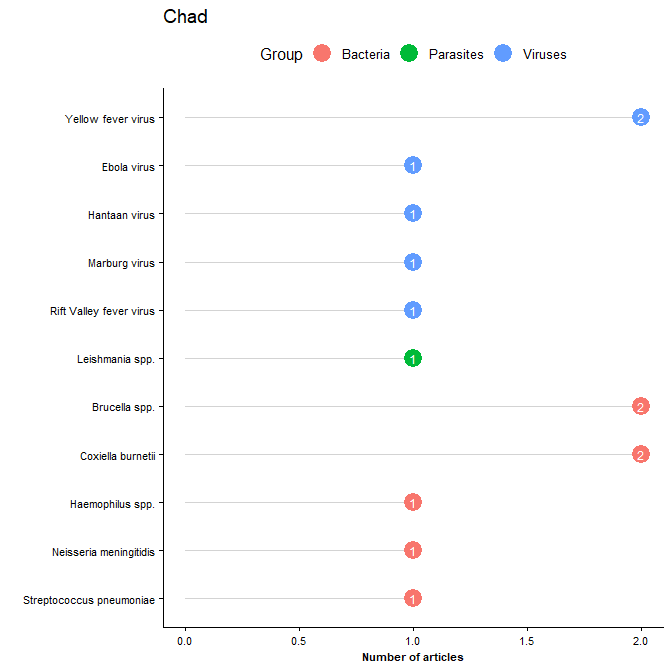


Legend: All reported pathogens by mode of transmission, a systematic review of published aetiological studies and case reports from Africa, 1980-2015. No distinction has been made between case series, fever series or seroprevalence studies. The number inside the dot plot shows the number of articles.

### 5.10 Comoros


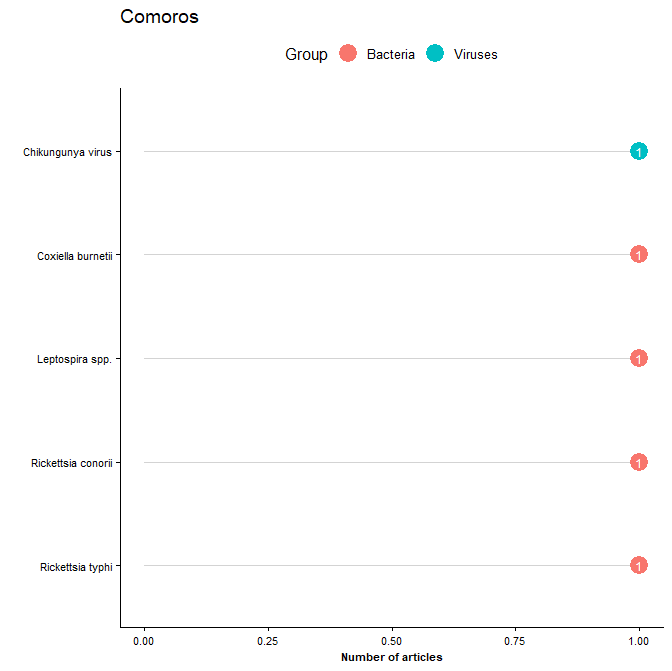


Legend: All reported pathogens by mode of transmission, a systematic review of published aetiological studies and case reports from Africa, 1980-2015. No distinction has been made between case series, fever series or seroprevalence studies. The number inside the dot plot shows the number of articles.

### 5.11 Congo


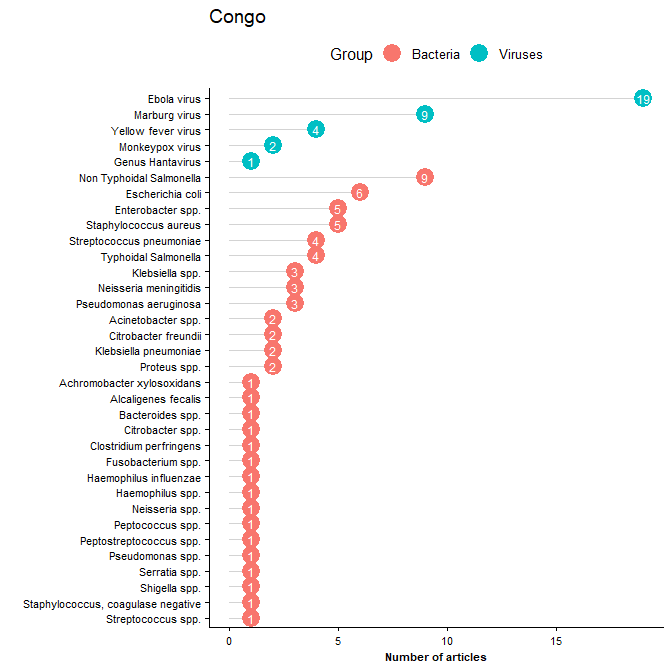


Legend: All reported pathogens by mode of transmission, a systematic review of published aetiological studies and case reports from Africa, 1980-2015. No distinction has been made between case series, fever series or seroprevalence studies. The number inside the dot plot shows the number of articles.

### 5.12 Cote d’ Ivore


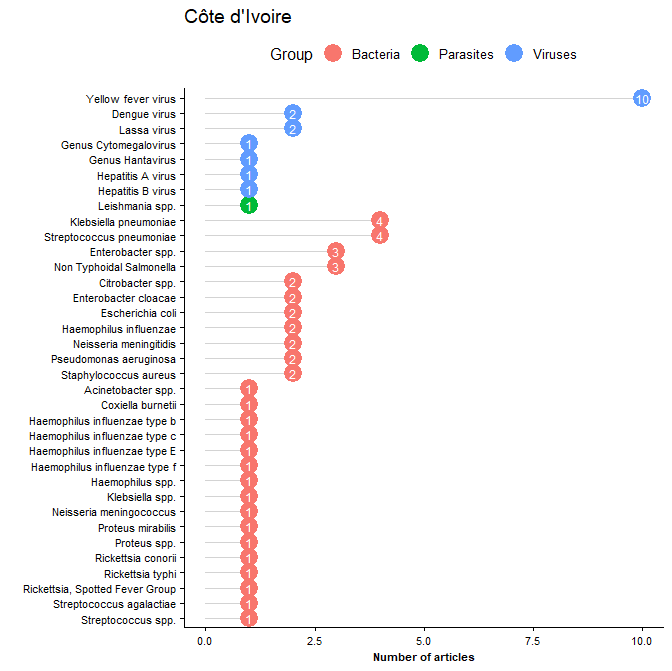


Legend: All reported pathogens by mode of transmission, a systematic review of published aetiological studies and case reports from Africa, 1980-2015. No distinction has been made between case series, fever series or seroprevalence studies. The number inside the dot plot shows the number of articles.

### 5.13 Djibouti


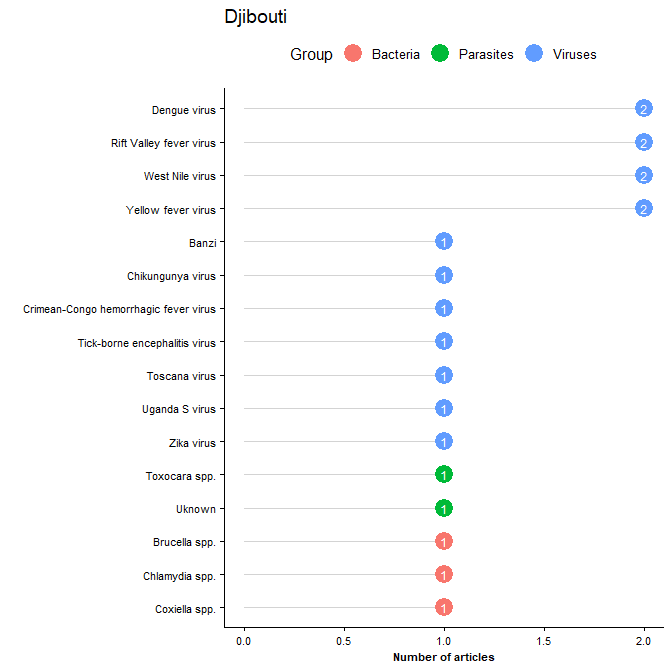


Legend: All reported pathogens by mode of transmission, a systematic review of published aetiological studies and case reports from Africa, 1980-2015. No distinction has been made between case series, fever series or seroprevalence studies. The number inside the dot plot shows the number of articles.

### 5.14 DRC


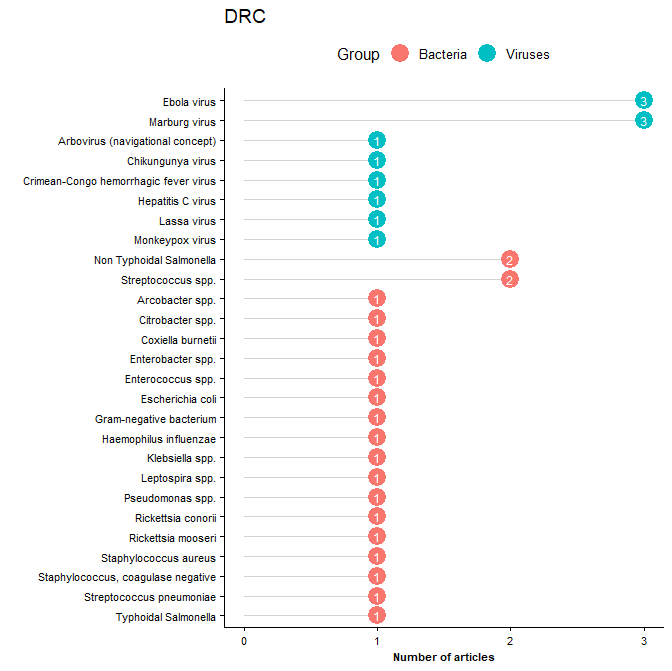


Legend: All reported pathogens by mode of transmission, a systematic review of published aetiological studies and case reports from Africa, 1980-2015. No distinction has been made between case series, fever series or seroprevalence studies. The number inside the dot plot shows the number of articles.

### 5.15 Egypt


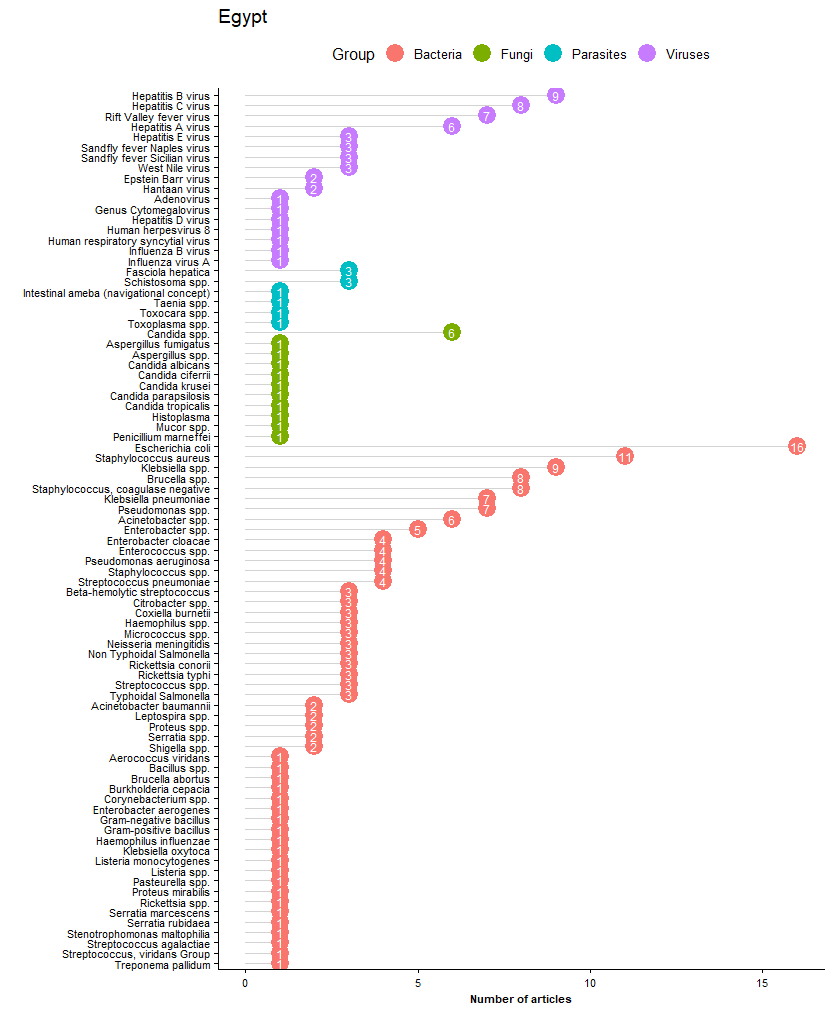


Legend: All reported pathogens by mode of transmission, a systematic review of published aetiological studies and case reports from Africa, 1980-2015. No distinction has been made between case series, fever series or seroprevalence studies. The number inside the dot plot shows the number of articles.

### 5.16 Equatorial Guinea


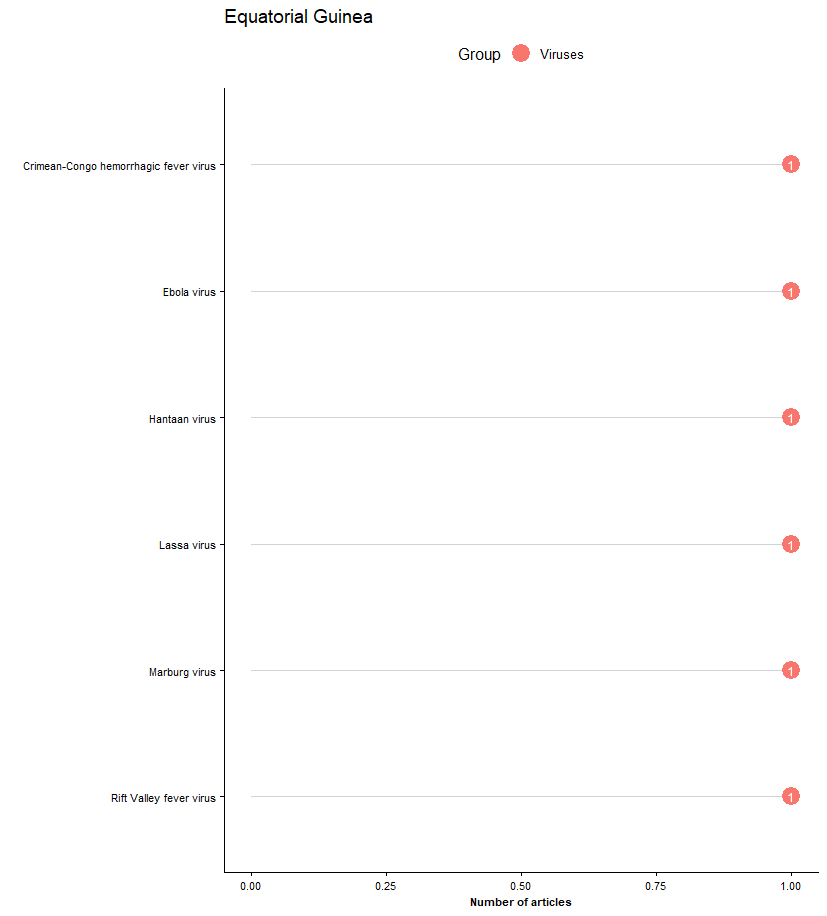


Legend: All reported pathogens by mode of transmission, a systematic review of published aetiological studies and case reports from Africa, 1980-2015. No distinction has been made between case series, fever series or seroprevalence studies. The number inside the dot plot shows the number of articles.

### 5.17 Ethiopia


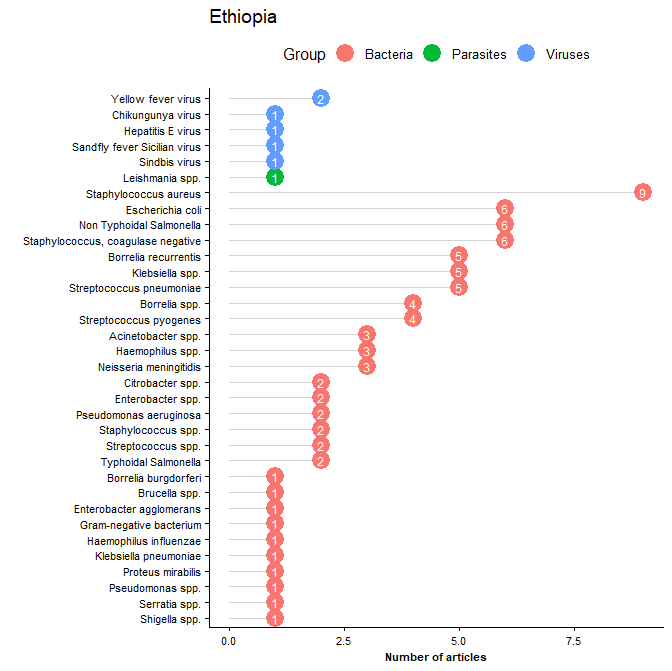


Legend: All reported pathogens by mode of transmission, a systematic review of published aetiological studies and case reports from Africa, 1980-2015. No distinction has been made between case series, fever series or seroprevalence studies. The number inside the dot plot shows the number of articles.

### 5.18 Gabon


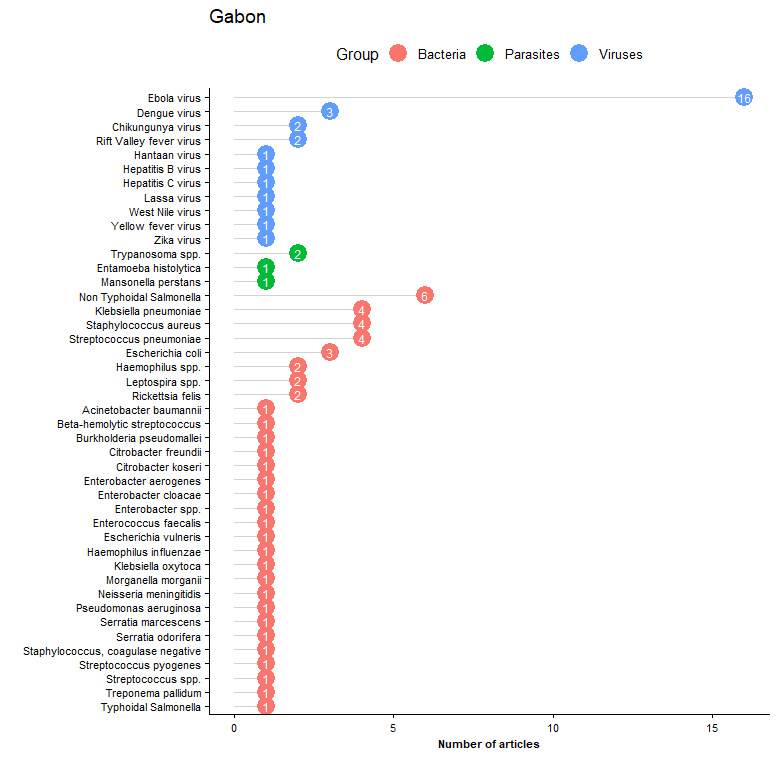


Legend: All reported pathogens by mode of transmission, a systematic review of published aetiological studies and case reports from Africa, 1980-2015. No distinction has been made between case series, fever series or seroprevalence studies. The number inside the dot plot shows the number of articles.

### 5.19 The Gambia


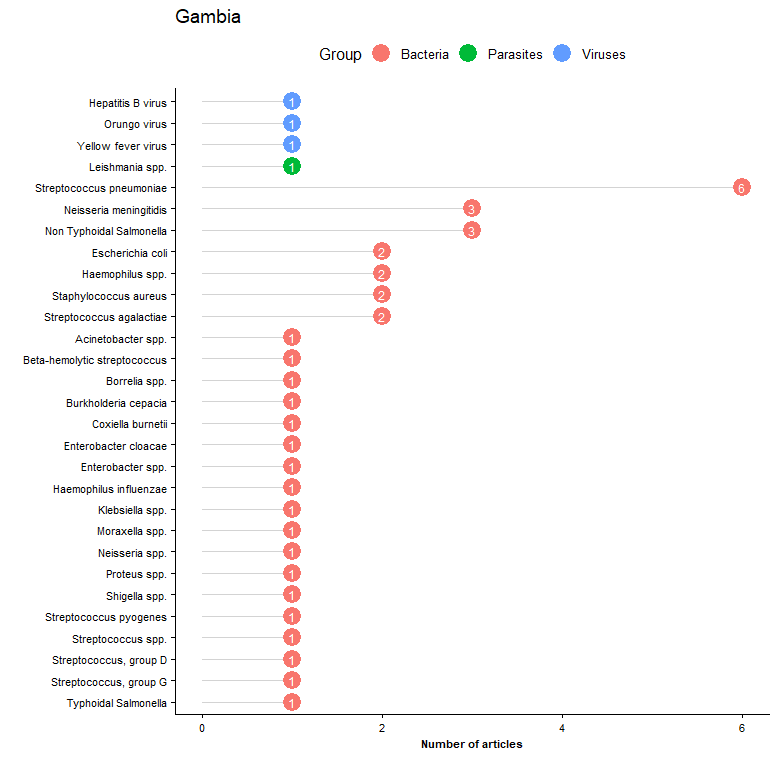


Legend: All reported pathogens by mode of transmission, a systematic review of published aetiological studies and case reports from Africa, 1980-2015. No distinction has been made between case series, fever series or seroprevalence studies. The number inside the dot plot shows the number of articles.

### 5.20 Ghana


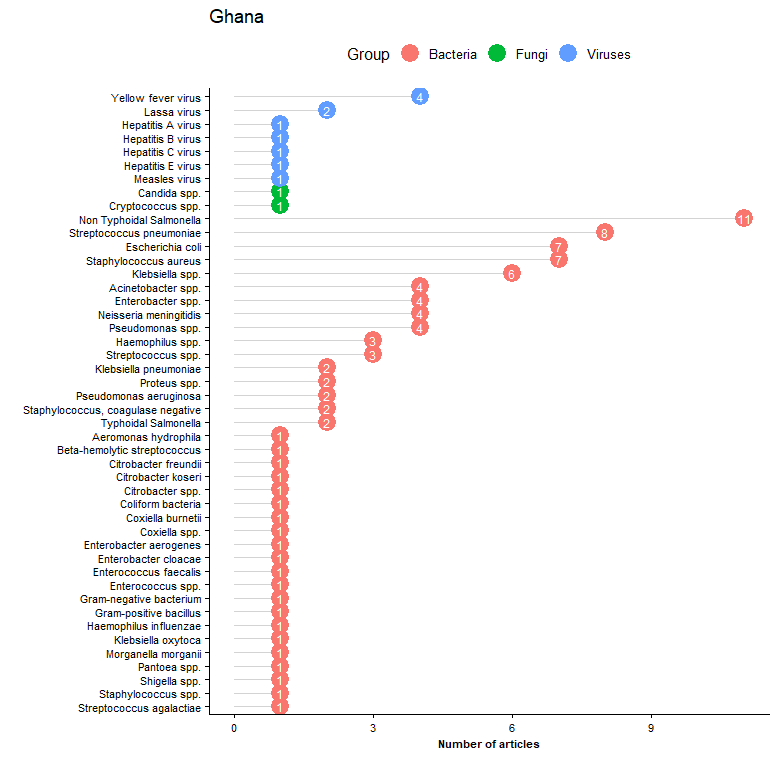


Legend: All reported pathogens by mode of transmission, a systematic review of published aetiological studies and case reports from Africa, 1980-2015. No distinction has been made between case series, fever series or seroprevalence studies. The number inside the dot plot shows the number of articles.

### 5.21 Guinea


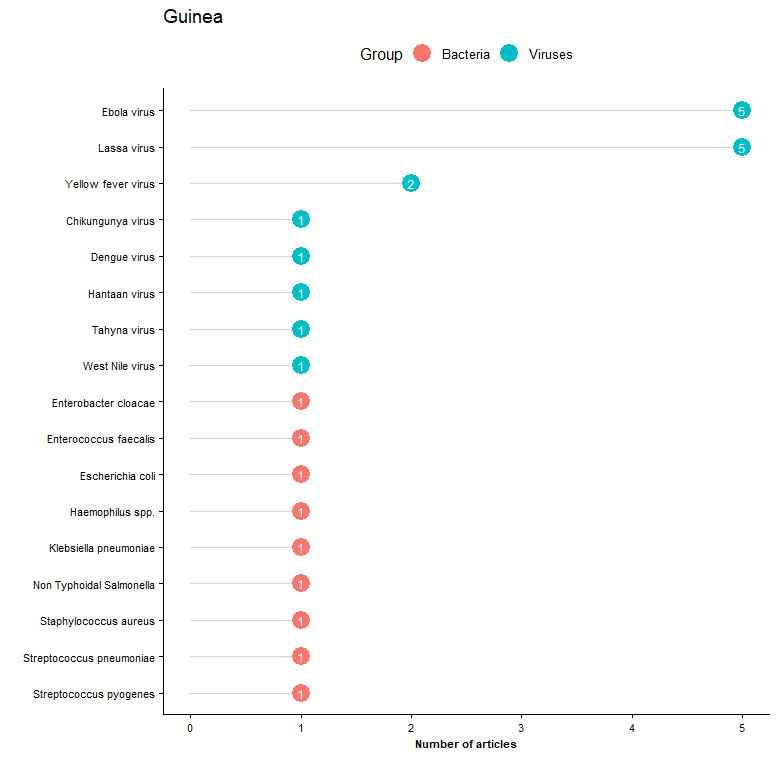


Legend: All reported pathogens by mode of transmission, a systematic review of published aetiological studies and case reports from Africa, 1980-2015. No distinction has been made between case series, fever series or seroprevalence studies. The number inside the dot plot shows the number of articles.

### 5.22 Kenya


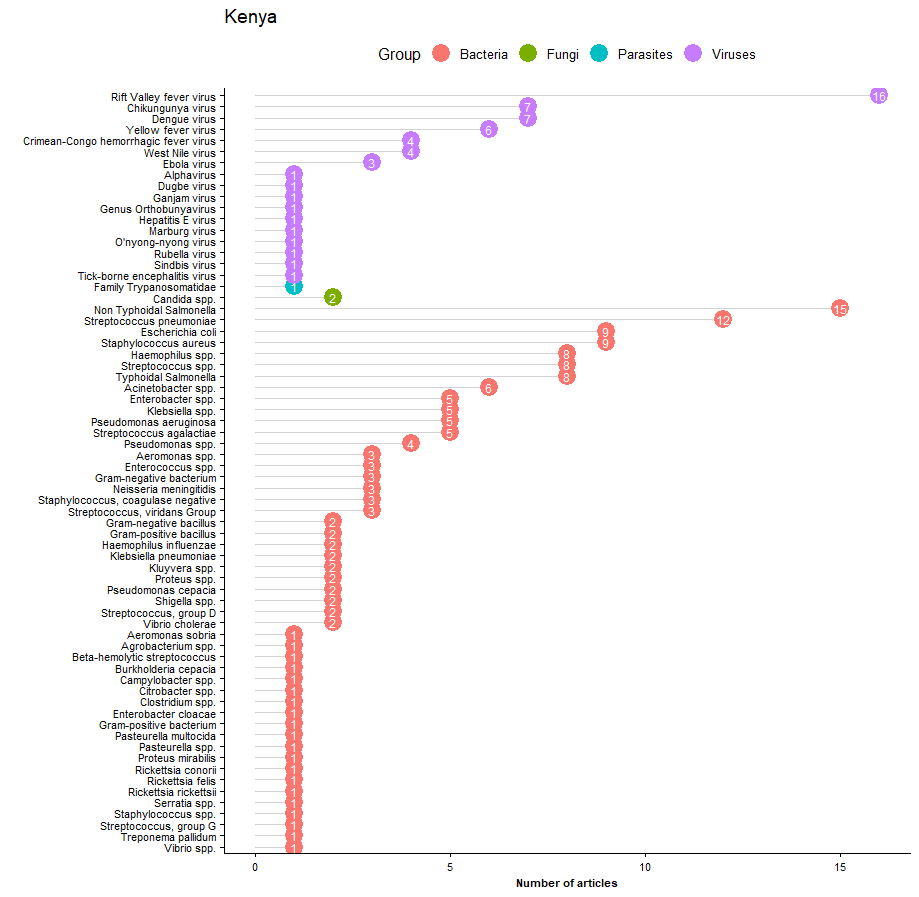


Legend: All reported pathogens by mode of transmission, a systematic review of published aetiological studies and case reports from Africa, 1980-2015. No distinction has been made between case series, fever series or seroprevalence studies. The number inside the dot plot shows the number of articles.

### 5.23 Liberia


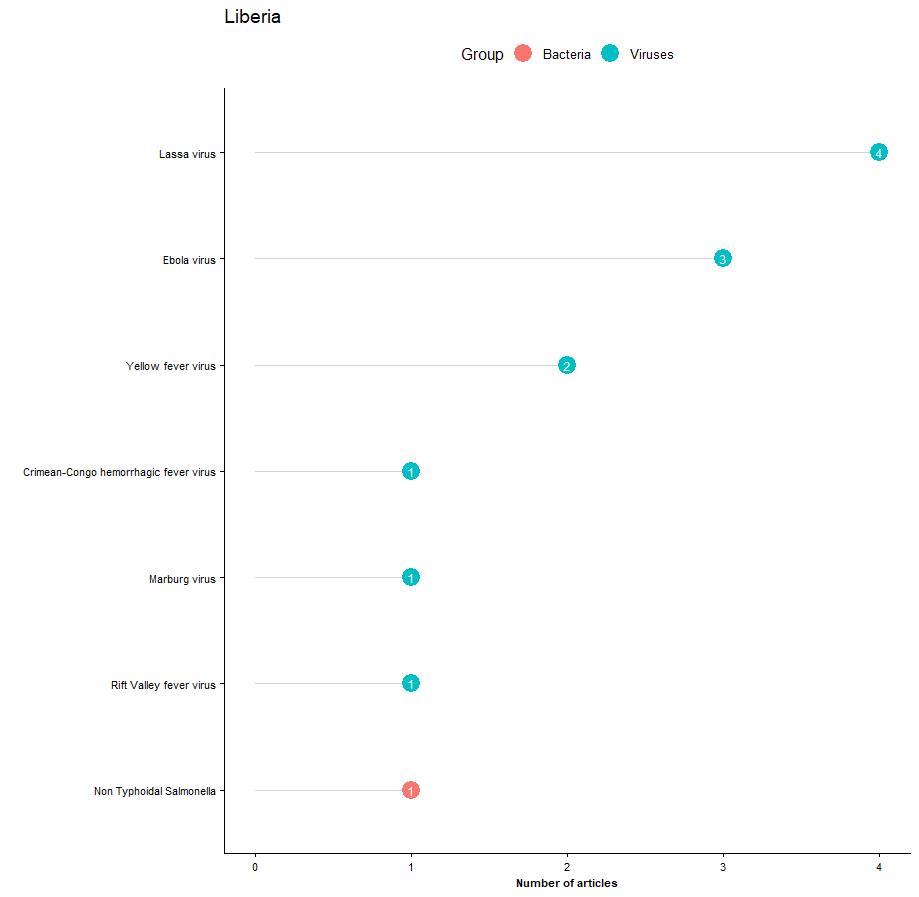


Legend: All reported pathogens by mode of transmission, a systematic review of published aetiological studies and case reports from Africa, 1980-2015. No distinction has been made between case series, fever series or seroprevalence studies. The number inside the dot plot shows the number of articles.

### 5.24 Libya


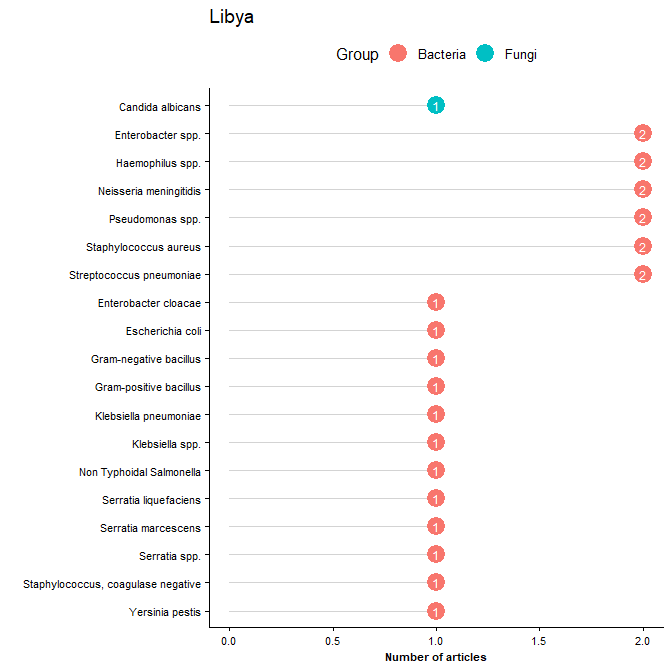


Legend: All reported pathogens by mode of transmission, a systematic review of published aetiological studies and case reports from Africa, 1980-2015. No distinction has been made between case series, fever series or seroprevalence studies. The number inside the dot plot shows the number of articles.

### 5.25 Madagascar


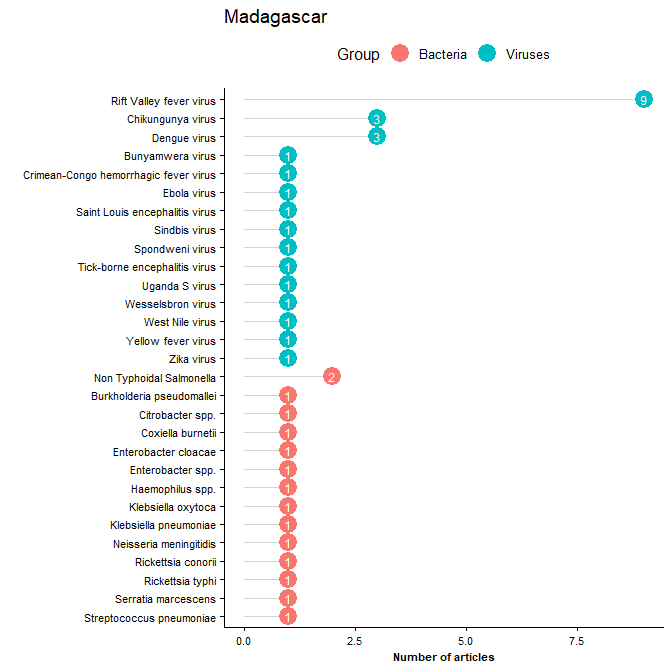


Legend: All reported pathogens by mode of transmission, a systematic review of published aetiological studies and case reports from Africa, 1980-2015. No distinction has been made between case series, fever series or seroprevalence studies. The number inside the dot plot shows the number of articles.

### 5.26 Malawi


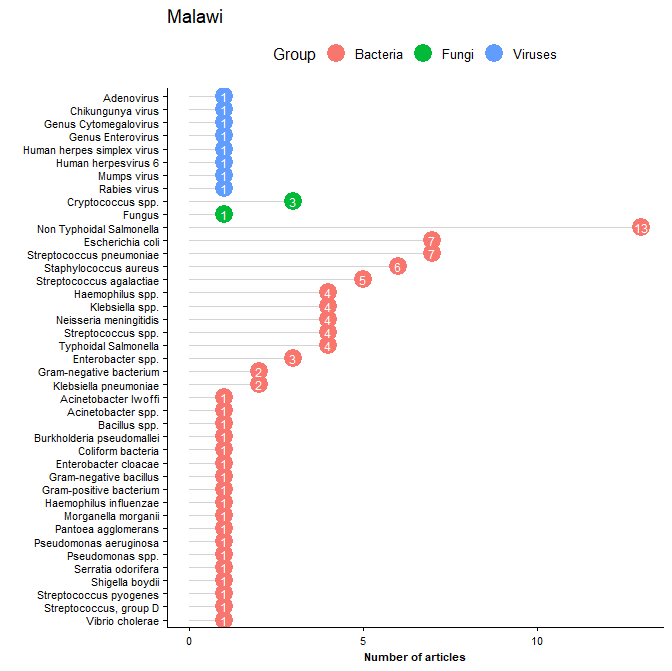


Legend: All reported pathogens by mode of transmission, a systematic review of published aetiological studies and case reports from Africa, 1980-2015. No distinction has been made between case series, fever series or seroprevalence studies. The number inside the dot plot shows the number of articles.

### 5.27 Mali


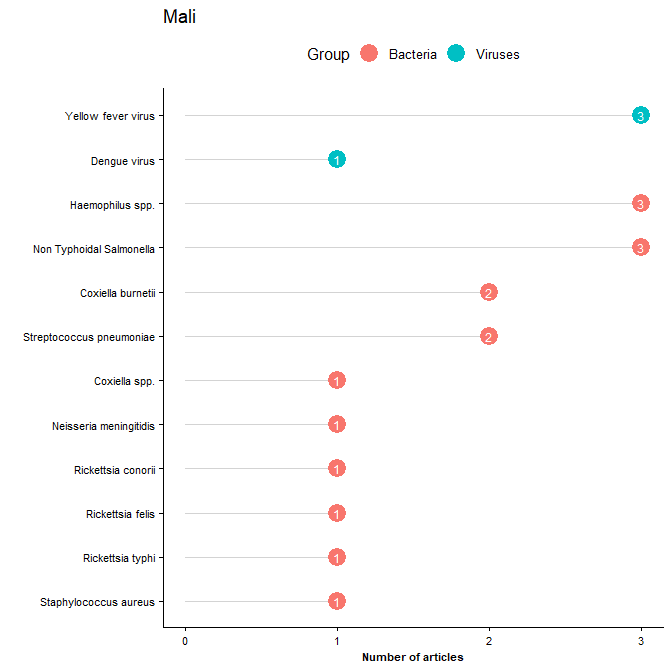


Legend: All reported pathogens by mode of transmission, a systematic review of published aetiological studies and case reports from Africa, 1980-2015. No distinction has been made between case series, fever series or seroprevalence studies. The number inside the dot plot shows the number of articles.

### 5.28 Mauritania


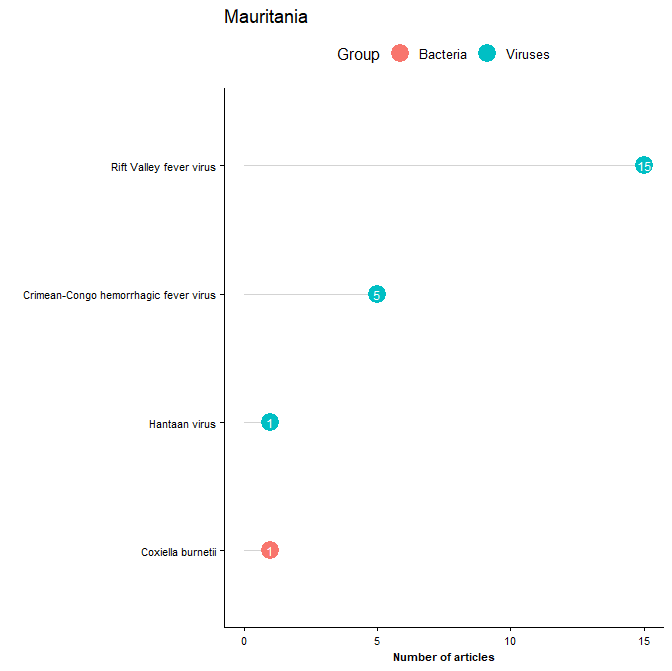


Legend: All reported pathogens by mode of transmission, a systematic review of published aetiological studies and case reports from Africa, 1980-2015. No distinction has been made between case series, fever series or seroprevalence studies. The number inside the dot plot shows the number of articles.

### 5.29 Mayotte


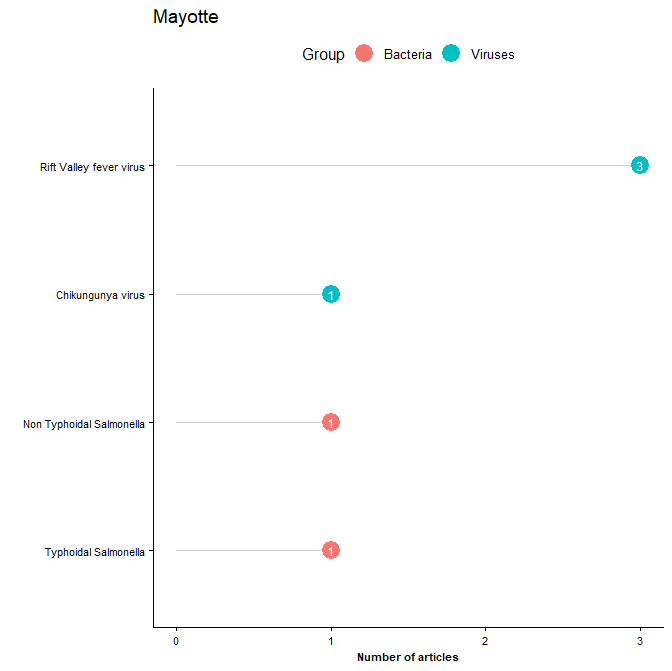


Legend: All reported pathogens by mode of transmission, a systematic review of published aetiological studies and case reports from Africa, 1980-2015. No distinction has been made between case series, fever series or seroprevalence studies. The number inside the dot plot shows the number of articles.

### 5.30 Morocco


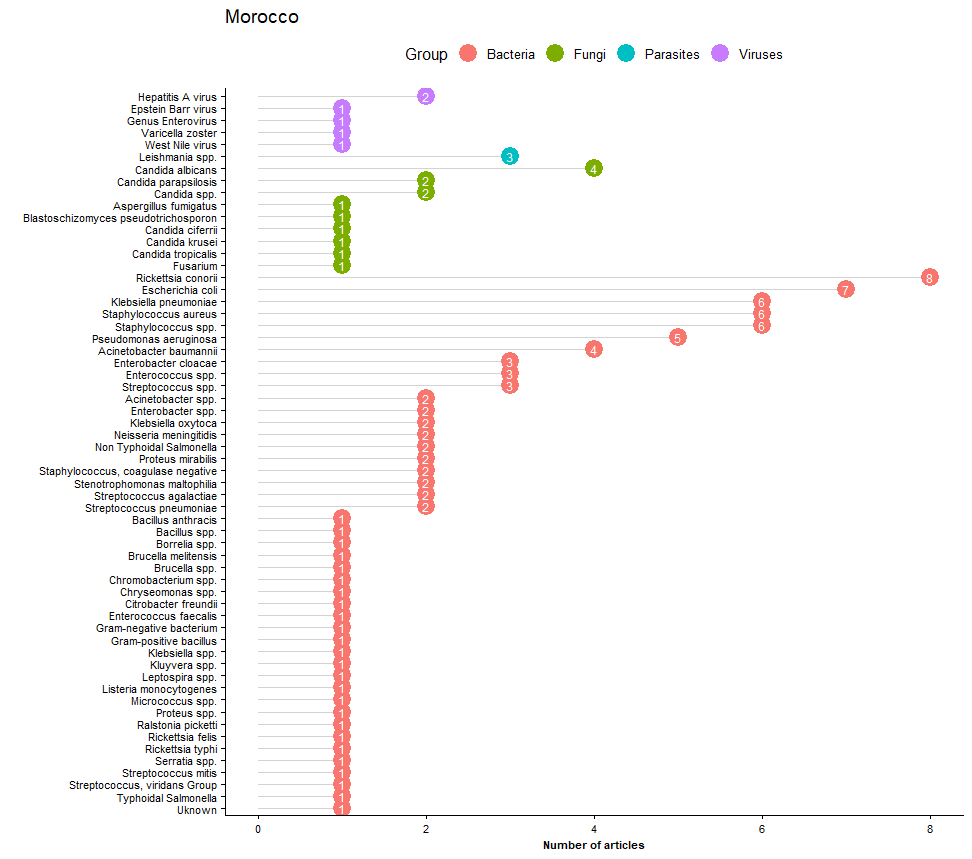


Legend: All reported pathogens by mode of transmission, a systematic review of published aetiological studies and case reports from Africa, 1980-2015. No distinction has been made between case series, fever series or seroprevalence studies. The number inside the dot plot shows the number of articles.

### 5.31 Mozambique


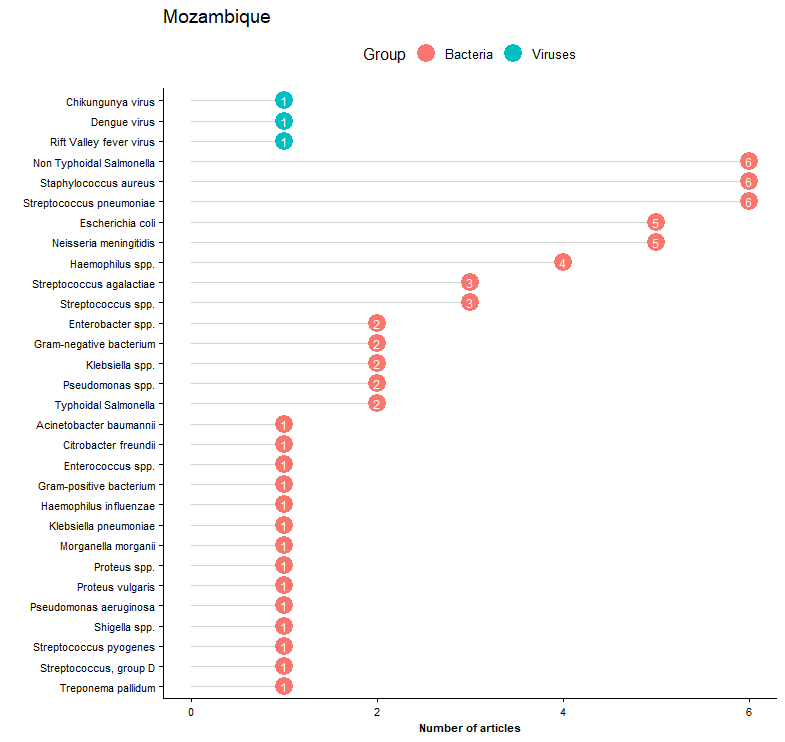


Legend: All reported pathogens by mode of transmission, a systematic review of published aetiological studies and case reports from Africa, 1980-2015. No distinction has been made between case series, fever series or seroprevalence studies. The number inside the dot plot shows the number of articles.

### 5.32 Namibia


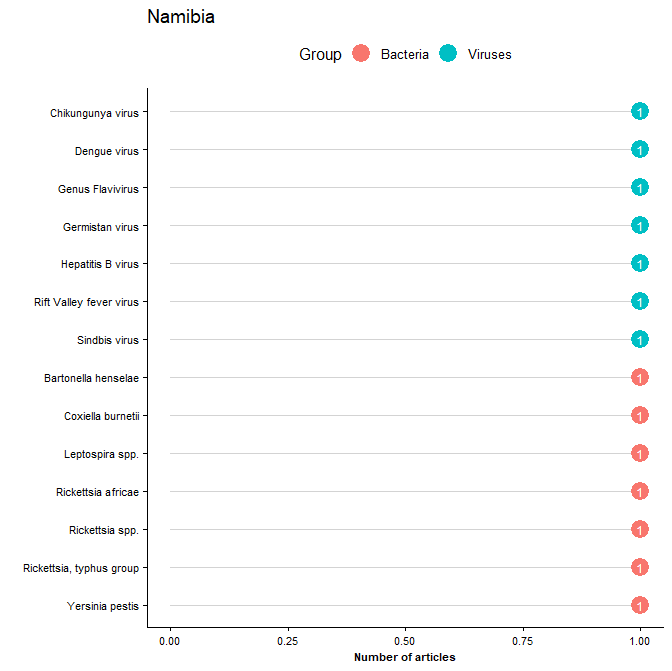


Legend: All reported pathogens by mode of transmission, a systematic review of published aetiological studies and case reports from Africa, 1980-2015. No distinction has been made between case series, fever series or seroprevalence studies. The number inside the dot plot shows the number of articles.

### 5.33 Niger


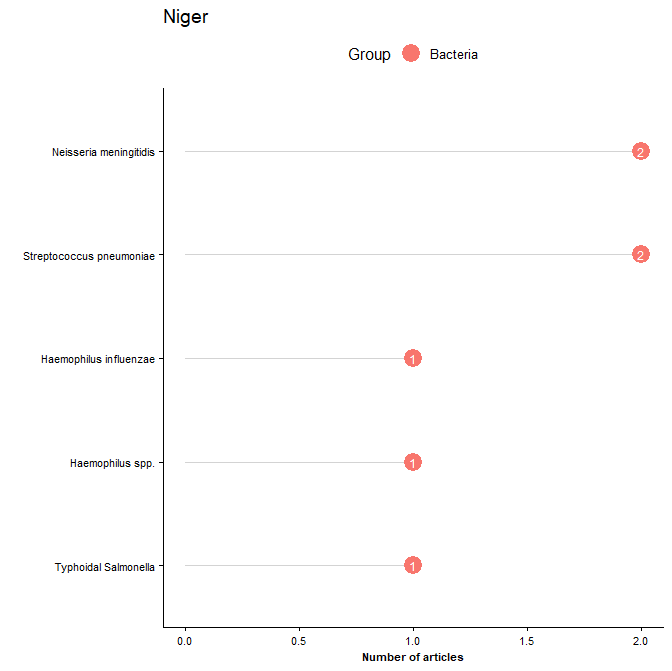


Legend: All reported pathogens by mode of transmission, a systematic review of published aetiological studies and case reports from Africa, 1980-2015. No distinction has been made between case series, fever series or seroprevalence studies. The number inside the dot plot shows the number of articles.

### 5.34 Nigeria


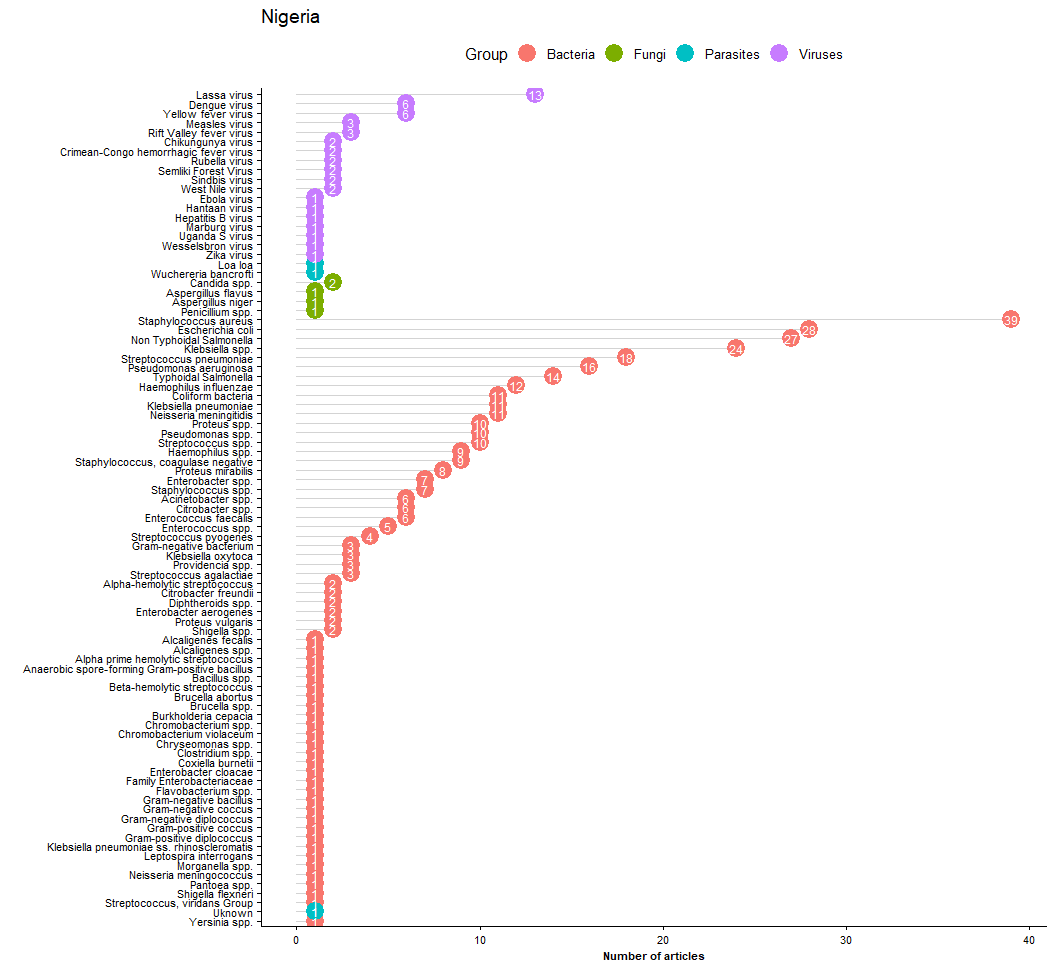


Legend: All reported pathogens by mode of transmission, a systematic review of published aetiological studies and case reports from Africa, 1980-2015. No distinction has been made between case series, fever series or seroprevalence studies. The number inside the dot plot shows the number of articles.

### 5.35 Rwanda


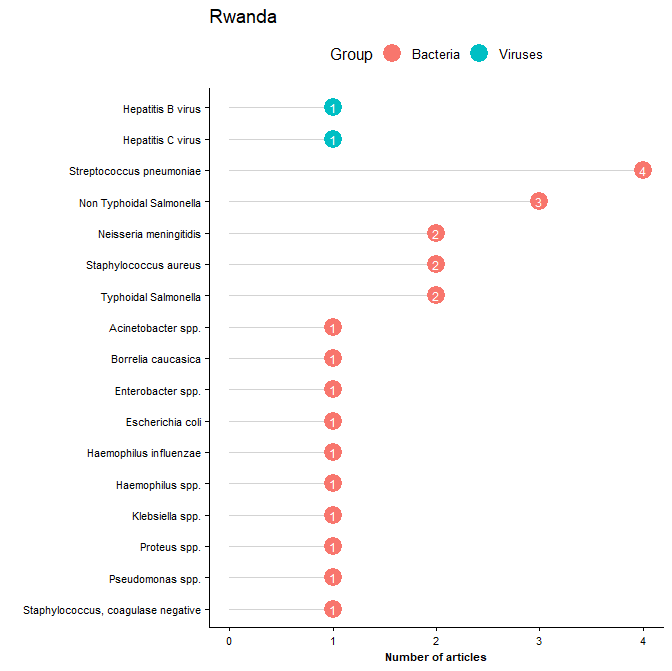


Legend: All reported pathogens by mode of transmission, a systematic review of published aetiological studies and case reports from Africa, 1980-2015. No distinction has been made between case series, fever series or seroprevalence studies. The number inside the dot plot shows the number of articles.

### 5.36 Senegal


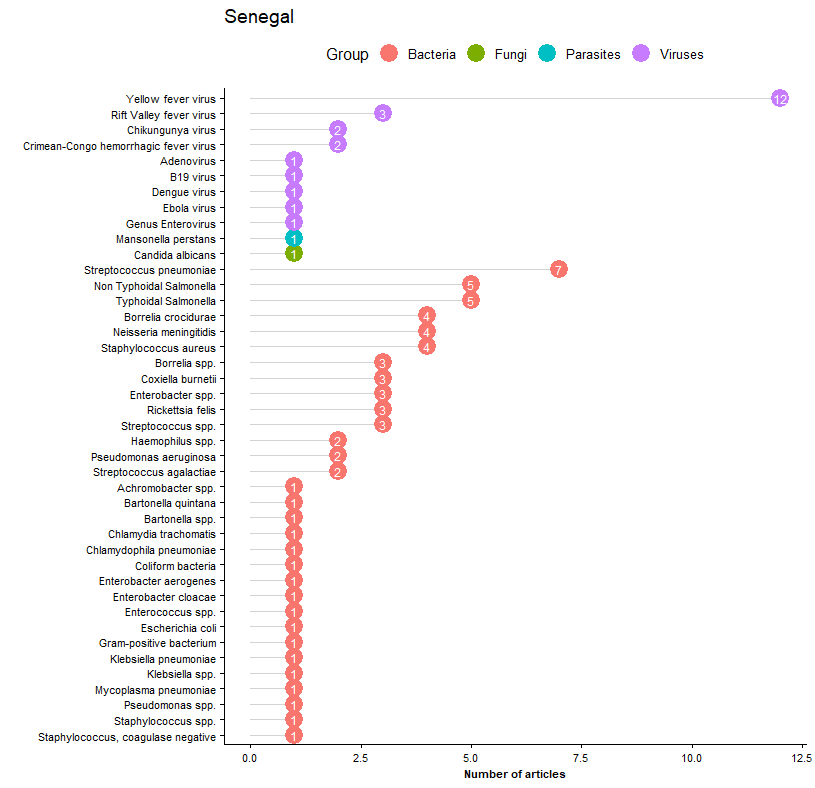


Legend: All reported pathogens by mode of transmission, a systematic review of published aetiological studies and case reports from Africa, 1980-2015. No distinction has been made between case series, fever series or seroprevalence studies. The number inside the dot plot shows the number of articles.

### 5.37 Sierra Leone


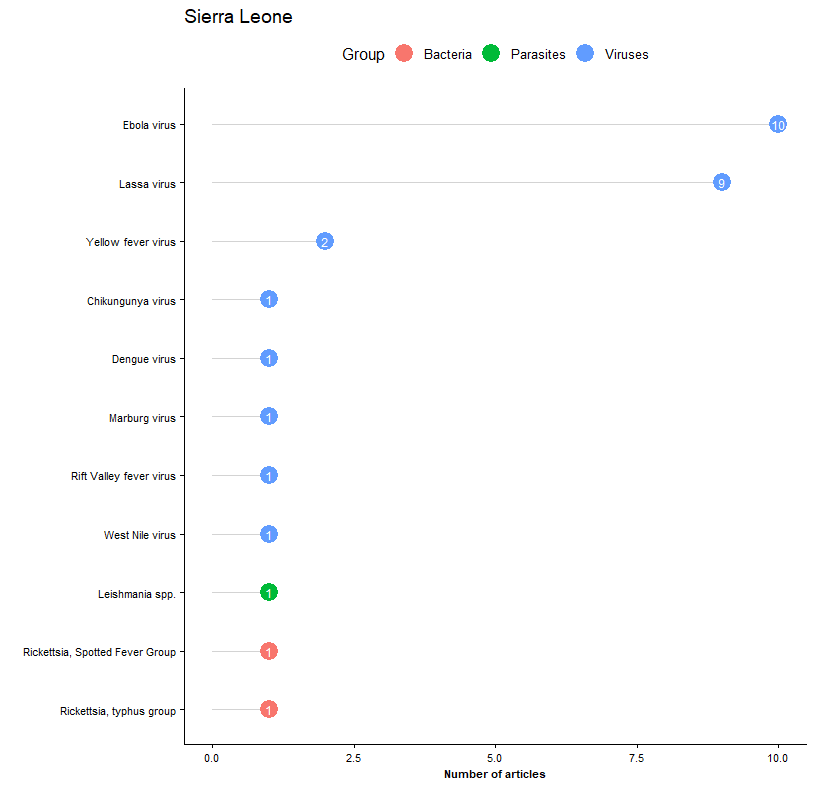


Legend: All reported pathogens by mode of transmission, a systematic review of published aetiological studies and case reports from Africa, 1980-2015. No distinction has been made between case series, fever series or seroprevalence studies. The number inside the dot plot shows the number of articles.

### 5.38 Somalia


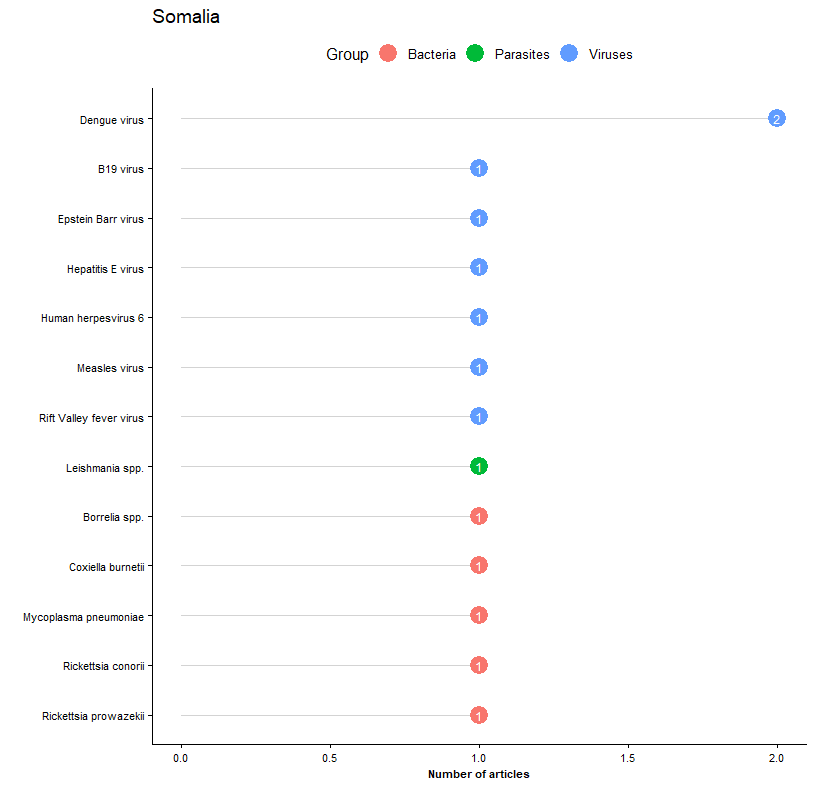
Legend: All reported pathogens by mode of transmission, a systematic review of published aetiological studies and case reports from Africa, 1980-2015. No distinction has been made between case series, fever series or seroprevalence studies. The number inside the dot plot shows the number of articles.

### 5.39 South Africa


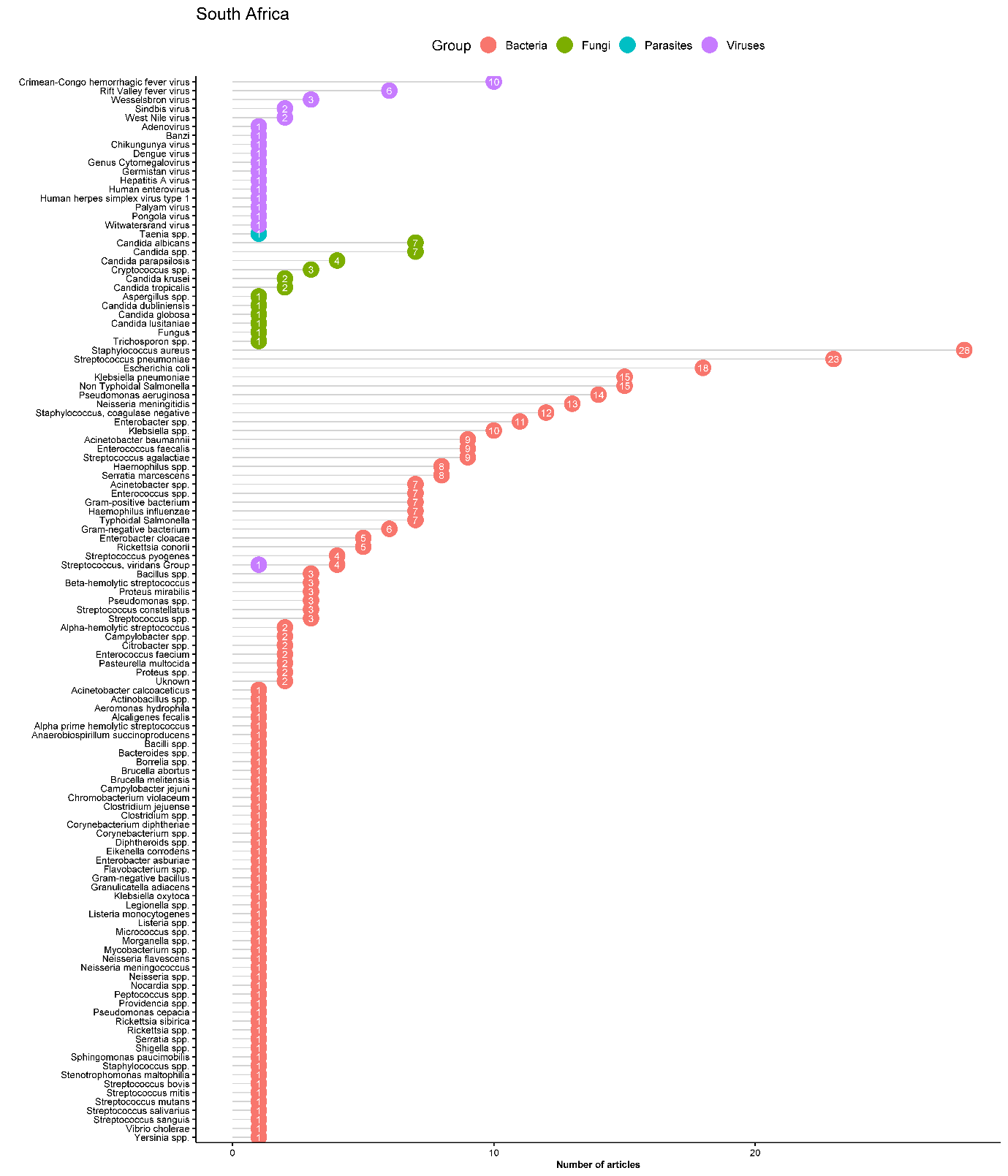


Legend: All reported pathogens by mode of transmission, a systematic review of published aetiological studies and case reports from Africa, 1980-2015. No distinction has been made between case series, fever series or seroprevalence studies. The number inside the dot plot shows the number of articles.

### 5.40 South Sudan


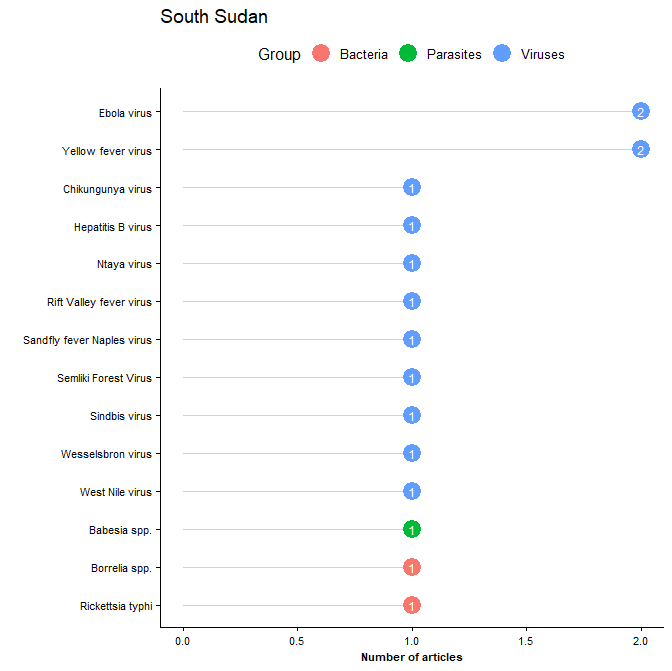


Legend: All reported pathogens by mode of transmission, a systematic review of published aetiological studies and case reports from Africa, 1980-2015. No distinction has been made between case series, fever series or seroprevalence studies. The number inside the dot plot shows the number of articles.

### 5.41 Sudan


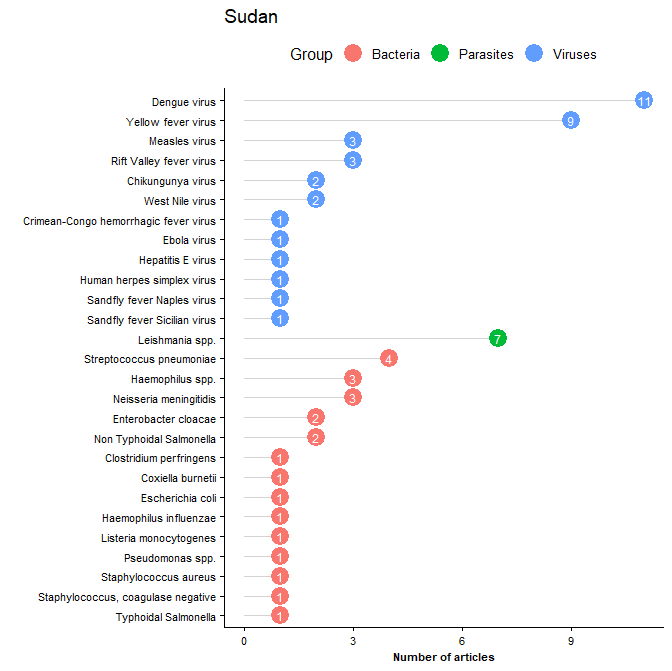


Legend: All reported pathogens by mode of transmission, a systematic review of published aetiological studies and case reports from Africa, 1980-2015. No distinction has been made between case series, fever series or seroprevalence studies. The number inside the dot plot shows the number of articles.

### 5.42 Swaziland


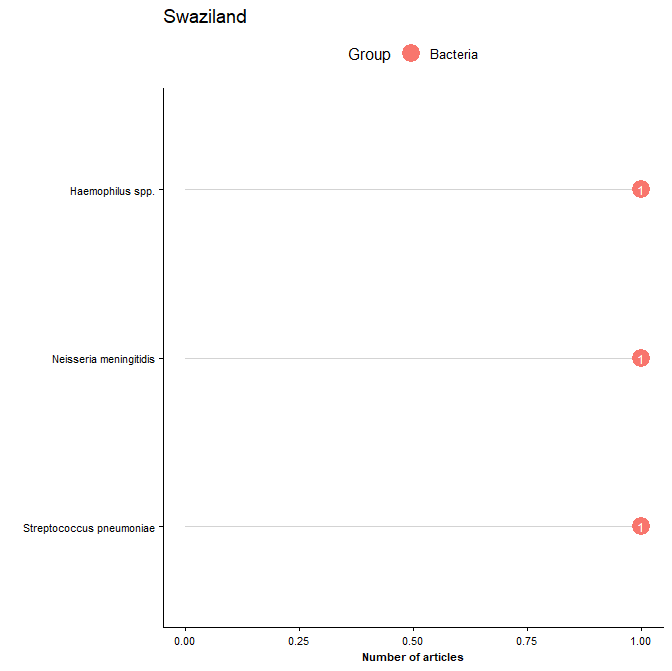


Legend: All reported pathogens by mode of transmission, a systematic review of published aetiological studies and case reports from Africa, 1980-2015. No distinction has been made between case series, fever series or seroprevalence studies. The number inside the dot plot shows the number of articles.

### 5.43 Tanzania


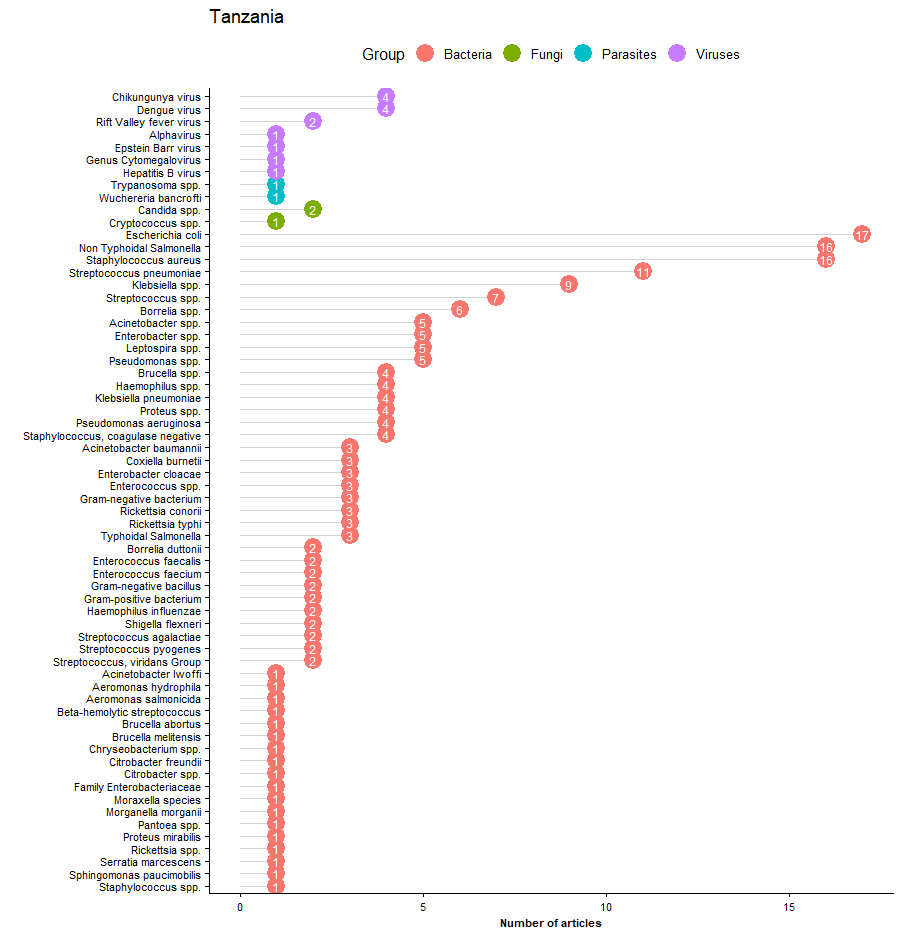


Legend: All reported pathogens by mode of transmission, a systematic review of published aetiological studies and case reports from Africa, 1980-2015. No distinction has been made between case series, fever series or seroprevalence studies. The number inside the dot plot shows the number of articles.

### 5.44 Togo


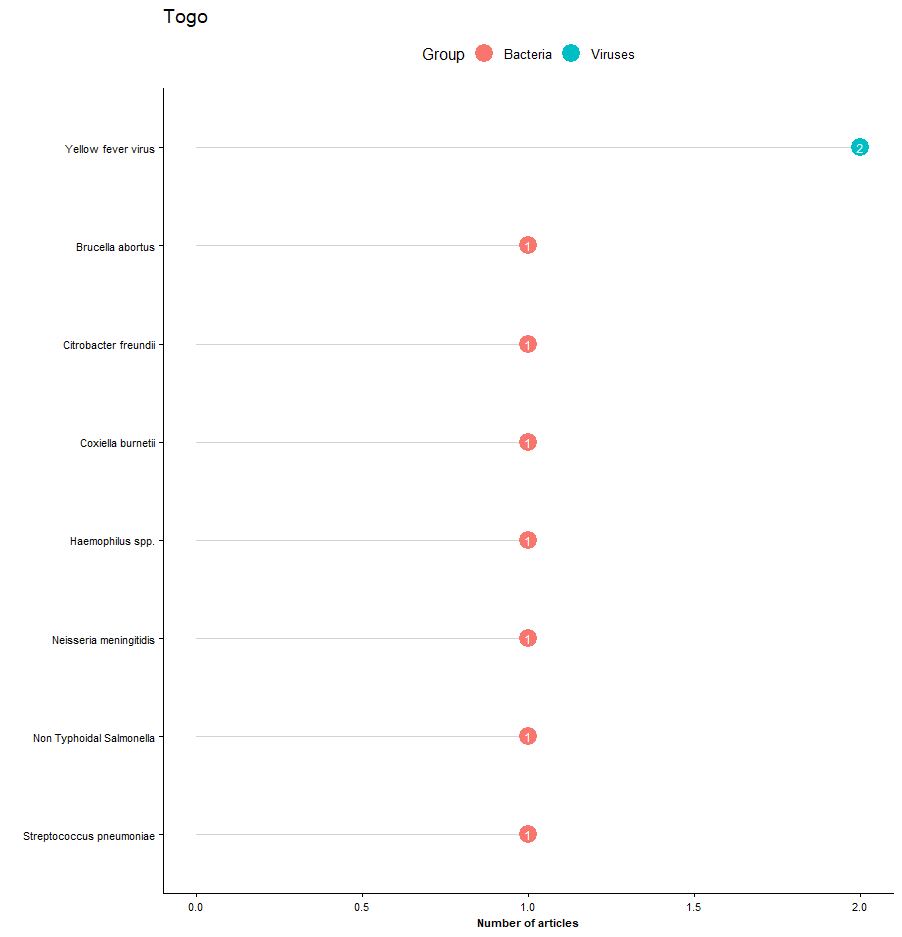
Legend: All reported pathogens by mode of transmission, a systematic review of published aetiological studies and case reports from Africa, 1980-2015. No distinction has been made between case series, fever series or seroprevalence studies. The number inside the dot plot shows the number of articles.

### 5.45 Tunisia


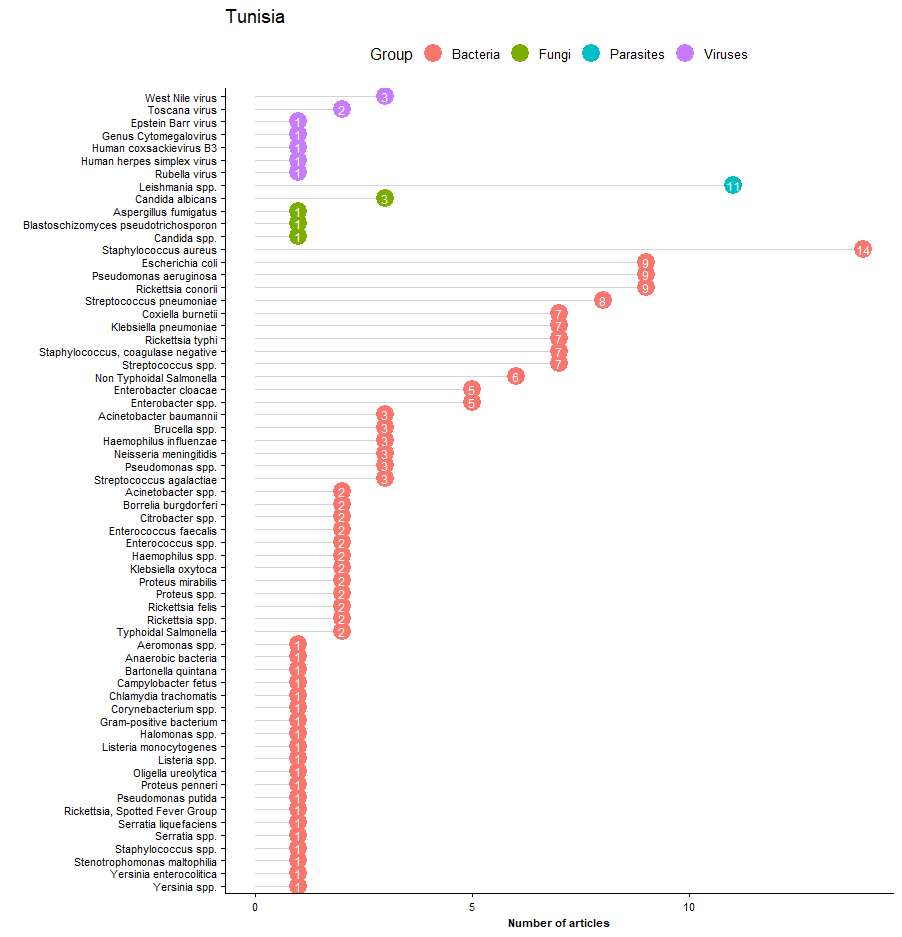


Legend: All reported pathogens by mode of transmission, a systematic review of published aetiological studies and case reports from Africa, 1980-2015. No distinction has been made between case series, fever series or seroprevalence studies. The number inside the dot plot shows the number of articles.

### 5.46 Uganda


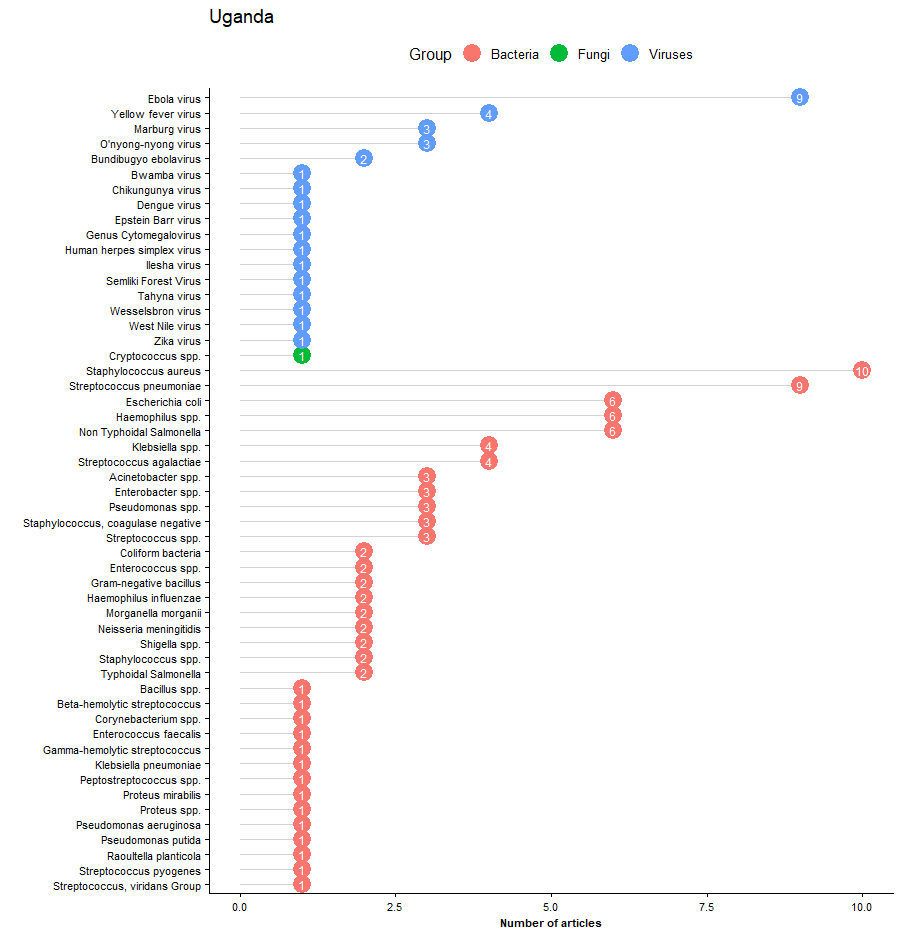


Legend: All reported pathogens by mode of transmission, a systematic review of published aetiological studies and case reports from Africa, 1980-2015. No distinction has been made between case series, fever series or seroprevalence studies. The number inside the dot plot shows the number of articles.

### 5.47 Zambia


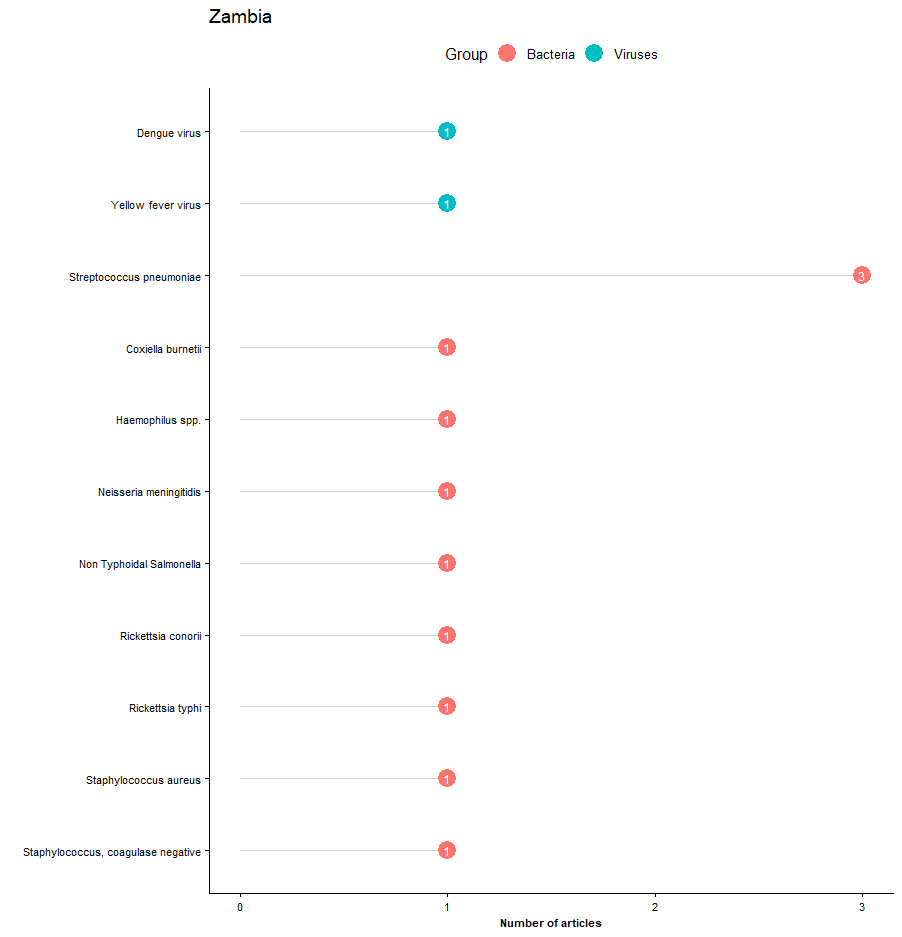


Legend: All reported pathogens by mode of transmission, a systematic review of published aetiological studies and case reports from Africa, 1980-2015. No distinction has been made between case series, fever series or seroprevalence studies. The number inside the dot plot shows the number of articles.

### 5.48 Zimbabwe


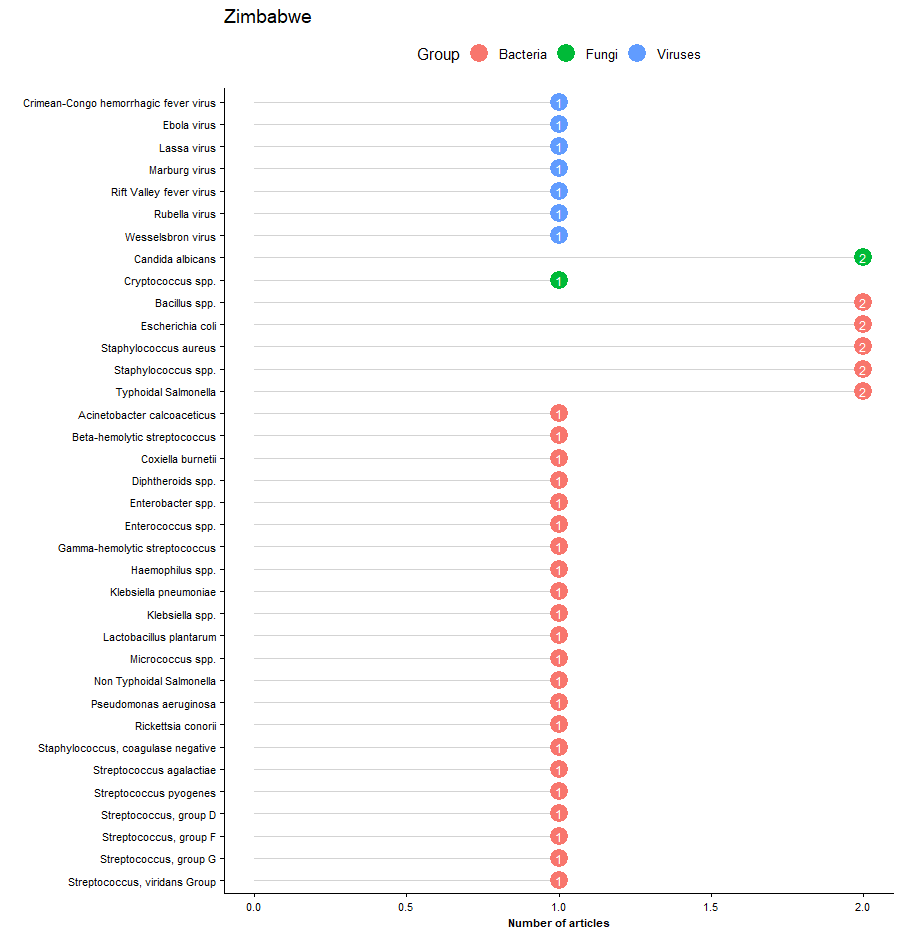


Legend: All reported pathogens by mode of transmission, a systematic review of published aetiological studies and case reports from Africa, 1980-2015. No distinction has been made between case series, fever series or seroprevalence studies. The number inside the dot plot shows the number of articles.
