# Supplementary material for: Non-malarial febrile illness: a systematic review of published aetiological studies and case reports from Africa, 1980–2015
Source: BMC Med. 2020 Sep 21;18:279. doi: 10.1186/s12916-020-01744-1 (PMC7504660; doi:10.1186/s12916-020-01744-1)
Supplement: Supplementary file 3 — Additional file 3. Assessment of risk of bias [file 12916_2020_1744_MOESM3_ESM.docx]

**Additional file 3: Assessment of risk of bias**

Non-malarial febrile illness: A systematic review of published aetiological studies and case reports from Africa, 1980-2015

Table of Contents

[Section 1: Methodology for the assessment of risk of bias in included studies 2](#_Toc29321448)

[1.1 Study meta-data and assessment of potential risk of bias in 1,065 included studies 3](#_Toc29321449)

[Section 2: Methodology for the assessment of risk of reporting bias in included studies toward urban settings 46](#_Toc29321450)

[2.1 Assessment of geographical bias toward reporting of data from urban settings in studies included in the systematic review 47](#_Toc29321451)

## **Section 1: Methodology for the assessment of risk of bias in included studies**

The usual risk of bias assessment which explicitly seeks to evaluate a study based on the selection, performance, attrition and reporting domains isn’t applicable for this review as it was designed to extract information on the causative pathogens of fever. The detection of a pathogen is affected by the laboratory method used, the study design, and selective reporting of the results.

We resort to using the terminology “potential risk of bias” rather than “risk of bias” as we have not extracted information on the records of an organism being sought but not found; a key metric required for comprehensive understanding of the aetiological agents of fever. The potential risk of bias in studies included in this review was thus carried out based on the on the study design (case series, fever series, seroprevalence studies), laboratory method used for pathogen detection and the selective reporting status.

The following rules were used:

- Case reports/series were considered to be at a high risk of bias because they report atypical presentations and are susceptible to reporting epidemiological outbreaks
- Seroprevalence studies were considered as moderate risk of bias as the distinction of the acute and past infection is dependent on the time at which samples are taken and doesn’t accurately represent prevalence/incidence
- For fever series, studies using culture or PCR methods were considered to be at low risk of bias, those using serological methods at moderate risk, and those not clearly reporting the diagnostic methods used at unclear risk

The study meta-data and the assessment of risk of bias is presented in Table 1.

### **Study meta-data and assessment of potential risk of bias in 1,065 included studies**

| IDDO id | Publication | Study Title | Study period | Study design | Diagnostic method(s) | Pathogen reported | number of +ve  microorganisms | Overall classification of "Potential" risk of bias |
| --- | --- | --- | --- | --- | --- | --- | --- | --- |
| 1 | Jennings–2007 | Brucellosis as a cause of acute febrile illness in Egypt | 2003—2003 | Fever Series | Serological | Bacteria/Viruses | 2 | Moderate |
| 13 | Formenty–2003 | Outbreak of Ebola hemorrhagic fever in the Republic of the Congo, 2003: a new strategy? | 2003—2003 | Case Series | Serological | Viruses | 1 | High |
| 22 | Bahri–2011 | Sero-epidemiological study of West Nile virus circulation in human in Tunisia | 2007—2007 | Seroprevalance study | Serological | Viruses | 1 | Moderate |
| 30 | Nkoghe–2011 | A limited outbreak of Ebola haemorrhagic fever in Etoumbi, Republic of Congo, 2005 | 2005—2005 | Case Series | Not described | Viruses | 1 | High |
| 34 | Jentes–2010 | Acute arboviral infections in Guinea, West Africa, 2006 | 2006—2007 | Fever Series | Serological | Viruses | 5 | Moderate |
| 41 | Cantaloube–2010 | Analysis of hepatitis C virus strains circulating in Republic of the Congo | 2005—2005 | Fever Series | PCR/Serological | Viruses | 1 | Low |
| 43 | Sarih–2009 | Borrelia hispanica relapsing fever, Morocco | 2005—2005 | Fever Series | Culture/PCR | Bacteria | 1 | Low |
| 44 | Mansour–2009 | Brucella meningitis: first reported case in Egypt | 2010—2010 | Case Series | Culture | Bacteria | 1 | High |
| 45 | Bouley–2012 | Brucellosis among hospitalized febrile patients in northern Tanzania | 2007—2008 | Fever Series | Serological | Bacteria | 1 | Moderate |
| 47 | Hertz–2012 | Chikungunya and dengue fever among hospitalized febrile patients in northern Tanzania | 2007—2008 | Seroprevalance study | Serological | Viruses | 2 | Moderate |
| 48 | Leroy–2009 | Concurrent chikungunya and dengue virus infections during simultaneous outbreaks, Gabon, 2007 | 2007—2007 | Fever Series | PCR | Viruses | 2 | Low |
| 51 | Malik–2011 | Dengue hemorrhagic fever outbreak in children in Port Sudan | 2004—2005 | Fever Series | Not described | Viruses | 2 | Unclear |
| 54 | Phoutrides–2011 | Dengue virus seroprevalence among febrile patients in Bamako, Mali: results of a 2006 surveillance study | 2006—2006 | Seroprevalance study | Serological | Viruses | 1 | Moderate |
| 56 | Jezek–1999 | Ebola between outbreaks: intensified Ebola hemorrhagic fever surveillance in the Democratic Republic of the Congo, 1981-1985 | 1981—1985 | Case Series | Serological | Viruses | 1 | High |
| 57 | Mupere–2001 | Ebola haemorrhagic fever among hospitalised children and adolescents in northern Uganda: epidemiologic and clinical observations | Not stated | Fever Series | PCR | Viruses | 1 | Low |
| 58 | Muyembe–1995 | Ebola haemorrhagic fever in Kikwit, Zaire. International Scientific and Technical Committee and WHO Collaborating Centre for Haemorrhagic Fevers. | 1995—1995 | Fever Series | PCR | Viruses | 1 | Low |
| 59 | Borchert–2011 | Ebola haemorrhagic fever outbreak in Masindi District, Uganda: outbreak description and lessons learned | Not stated | Case Series | Not described | Viruses | 1 | High |
| 61 | Wamala–2010 | Ebola hemorrhagic fever associated with novel virus strain, Uganda, 2007-2008 | 2007—2008 | Fever Series | Serological | Viruses | 1 | Moderate |
| 66 | Yalley-Ogunro–1984 | Endemic Lassa fever in Liberia. VI. Village serological surveys for evidence of Lassa virus activity in Lofa County, Liberia | 1980—1980 | Fever Series | Serological | Viruses | 1 | Moderate |
| 70 | Feki–2005 | Epidemic West Nile virus encephalitis in Tunisia | 1997—1997 | Fever Series | Serological | Viruses | 1 | Moderate |
| 73 | Wamala–2012 | Epidemiological and laboratory characterization of a yellow fever outbreak in northern Uganda, October 2010-January 2011 | 2010—2010 | Fever Series | Serological | Viruses | 1 | Moderate |
| 74 | Delarocque-Astagneau–2012 | Epidemiological and virological characteristics of symptomatic acute hepatitis E in Greater Cairo, Egypt | 2002—2007 | Fever Series | Serological | Viruses | 3 | Moderate |
| 75 | Abdallah–2012 | Epidemiology of dengue infections in Kassala, Eastern Sudan | 2010—2010 | Fever Series | Serological | Viruses | 1 | Moderate |
| 76 | Roddy–2010 | Factors associated with Marburg hemorrhagic fever: analysis of patient data from Uige, Angola | 2005—2005 | Fever Series | PCR | Viruses | 1 | Low |
| 77 | Bonney–2012 | Fatal hepatitis E viral infection in pregnant women in Ghana: a case series | 2010—2010 | Case Series | PCR | Viruses | 1 | High |
| 80 | Sfar–2009 | First molecular detection of R. conorii subsp. conorii 99 years after the Conor description of Mediterranean spotted fever, in Tunisia | 2007—2007 | Fever Series | Serological | Bacteria | 2 | Moderate |
| 81 | Sanders–1998 | First recorded outbreak of yellow fever in Kenya, 1992-1993. I. Epidemiologic investigations | Not stated | Case Series | Not described | Viruses | 1 | High |
| 83 | Pourrut–2010 | First serological evidence of West Nile virus in human rural populations of Gabon | 2009—2009 | Seroprevalance study | Serological | Viruses | 1 | Moderate |
| 84 | Becquart–2010 | High prevalence of both humoral and cellular immunity to Zaire ebolavirus among rural populations in Gabon | 2007—2007 | Seroprevalance study | Serological | Viruses | 1 | Moderate |
| 86 | Ehichioya–2012 | Hospital-based surveillance for Lassa fever in Edo State, Nigeria, 2005-2008 | 2007—2008 | Fever Series | Culture/PCR | Viruses | 1 | Low |
| 87 | Farnon–2010 | Household-based sero-epidemiologic survey after a yellow fever epidemic, Sudan, 2005 | 2005—2005 | Seroprevalance study | Serological | Viruses | 1 | Moderate |
| 88 | Richards–2010 | Human Infection with Rickettsia felis, Kenya | 2006—2008 | Fever Series | PCR | Bacteria | 1 | Low |
| 89 | Mokrani–2012 | Human rickettsioses in the Batna area, eastern Algeria | 2000—2006 | Fever Series | Serological | Bacteria | 5 | Moderate |
| 93 | Raoult–1997 | Jail fever (epidemic typhus) outbreak in Burundi | 1995—1996 | Fever Series | Not described | Bacteria | 2 | Unclear |
| 96 | Webb–1986 | Lassa fever in children in Sierra Leone, West Africa | 1977—1981 | Fever Series | Serological | Viruses | 1 | Moderate |
| 97 | Ehichioya–2010 | Lassa fever, Nigeria, 2005-2008 | 2005—2008 | Case Series | PCR | Viruses | 1 | High |
| 98 | Randall–1980 | Legionnaires' disease in Port Elizabeth | 1978—1979 | Case Series | Serological | Bacteria | 1 | High |
| 99 | Biggs–2011 | Leptospirosis among hospitalized febrile patients in northern Tanzania | 2007—2008 | Fever Series | Serological | Bacteria | 1 | Moderate |
| 100 | Bertherat–1999 | Leptospirosis and Ebola virus infection in five gold-panning villages in northeastern Gabon | 1995—1995 | Fever Series | Serological | Bacteria/Viruses | 2 | Moderate |
| 104 | Heffernan–2005 | Low seroprevalence of IgG antibodies to Ebola virus in an epidemic zone: Ogooue-Ivindo region, Northeastern Gabon, 1997 | 1997—1997 | Fever Series/Seroprevalence | Serological | Viruses | 2 | Moderate |
| 106 | Colebunders–2007 | Marburg hemorrhagic fever in Durba and Watsa, Democratic Republic of the Congo: clinical documentation, features of illness, and treatment | 1998—2000 | Case Series | Serological | Viruses | 1 | High |
| 107 | Mouffok–2009 | Mediterranean spotted fever in Algerian children | 2004—2005 | Fever Series | Serological | Bacteria | 1 | Moderate |
| 108 | Mouffok–2009 | Mediterranean spotted fever in Algeria--new trends | 2004—2005 | Fever Series | Serological | Bacteria | 1 | Moderate |
| 109 | Romdhane–2009 | Mediterranean spotted fever: a report of 200 cases in Tunisia | 1987—2006 | Case Series | Serological | Bacteria | 1 | High |
| 110 | Lepage–1986 | Neonatal relapsing fever in Rwanda | 1986—1986 | Case Series | Culture | Bacteria | 1 | High |
| 112 | Feldman–1988 | Pneumococcal bacteraemia in adults in a low socio-economic urban population | 1984—1985 | Case Series | Culture | Bacteria | 1 | High |
| 113 | Feldman–1991 | Comparison of bacteraemic community-acquired lobar pneumonia due to Streptococcus pneumoniae and Klebsiella pneumoniae in an intensive care unit | 1982—1985 | Case Series | Culture | Bacteria | 2 | High |
| 114 | World Health Organisation–2011 | Outbreak news. Yellow fever, Cote d'Ivoire | 2011—2011 | Seroprevalance study | Serological | Viruses | 1 | Moderate |
| 115 | Obaro–2011 | Community aquired bacteria bacteremia in young children from central Nigeria--a pilot study | 2008—2009 | Fever Series | Culture | Bacteria/Fungi | 18 | Low |
| 116 | Thriemer–2012 | The burden of invasive bacterial infections in Pemba, Zanzibar. | 2009—2010 | Fever Series | Culture | Bacteria | 5 | Low |
| 117 | Nadjm–2010 | WHO guidelines for antimicrobial treatment in children admitted to hospital to an area of intense Plasmodium falciparum transmission: prospective study. | 2006—2007 | Fever Series | PCR | Bacteria | 8 | Low |
| 120 | Berkley–2005 | Bacteremia among children admitted to a rural hospital in Kenya. | 1998—2002 | Fever Series | Culture | Bacteria | 12 | Low |
| 121 | SigaÃºque B–2009 | Community-acquired bacteremia among children admitted to a rural hospital in Mozambique | 2001—2006 | Fever Series | Culture | Bacteria | 14 | Low |
| 124 | World Health Organization–2009 | Outbreak news. Yellow fever, Republic of the Congo | 2009—2009 | Case Series | Not described | Viruses | 1 | High |
| 125 | World Health Organization–2011 | Outbreak news. Yellow fever, Sierra Leone | 2011—2011 | Case Series | Serological | Viruses | 1 | High |
| 126 | World Health Organization–2012 | Outbreak news. Yellow fever, Sudan | 2012—2012 | Case Series | Serological | Viruses | 1 | High |
| 127 | World Health Organization–2012 | Outbreak news. Yellow fever, Sudan - update | 2012—2012 | Case Series | Serological | Viruses | 1 | High |
| 128 | World Health Organization–2007 | Outbreak news. Yellow fever, Togo--update | 2007—2007 | Case Series | Serological | Viruses | 1 | High |
| 129 | World Health Organization–2011 | Outbreak news. Yellow fever, Uganda | 2010—2010 | Case Series | Not described | Viruses | 1 | High |
| 130 | Ratsitorahina–2008 | Outbreak of dengue and Chikungunya fevers, Toamasina, Madagascar, 2006 | 2006—2006 | Fever Series | Serological | Viruses | 2 | Moderate |
| 131 | World Health Organization–1996 | Outbreak of Ebola haemorrhagic fever in Gabon | 1996—1996 | Case Series | Not described | Viruses | 1 | High |
| 132 | World Health Organization–2005 | Outbreak of Ebola haemorrhagic fever in Yambio, south Sudan, April - June 2004 | 2004—2004 | Case Series/Fever Series | Not described | Viruses | 2 | High |
| 134 | Raoult–1998 | Outbreak of epidemic typhus associated with trench fever in Burundi | 1997—1997 | Case Series | Serological | Bacteria | 2 | High |
| 135 | Hutin–2001 | Outbreak of human monkeypox, Democratic Republic of Congo, 1996 to 1997 | 1996—1997 | Fever Series | Serological | Viruses | 1 | Moderate |
| 137 | Adjemian–2011 | Outbreak of Marburg hemorrhagic fever among miners in Kamwenge and Ibanda Districts, Uganda, 2007 | Not stated | Case Series | Not described | Viruses | 1 | High |
| 140 | World Health Organization–2003 | Outbreak(s) of Ebola haemorrhagic fever in the Republic of the Congo, January-April 2003 | 2002—2003 | Case Series | Serological | Viruses | 1 | High |
| 142 | KernÃ©is–2009 | Prevalence and risk factors of Lassa seropositivity in inhabitants of the forest region of Guinea: a cross-sectional study | 2000—2000 | Seroprevalance study | Serological | Viruses | 1 | Moderate |
| 143 | Busico–1999 | Prevalence of IgG antibodies to Ebola virus in individuals during an Ebola outbreak, Democratic Republic of the Congo, 1995 | 1995—1995 | Fever Series | Serological | Viruses | 1 | Moderate |
| 145 | MacNeil–2010 | Proportion of deaths and clinical features in Bundibugyo Ebola virus infection, Uganda | 2007—2007 | Case Series | Not described | Viruses | 1 | High |
| 147 | Blondeau–1990 | Q fever in Sokoto, Nigeria | 1953—1954 | Fever Series | Serological | Bacteria | 1 | Moderate |
| 148 | Kobbe–2008 | Q fever in young children, Ghana | 2007—2007 | Case Series/Fever Series | Serological | Bacteria | 2 | High |
| 150 | Prabhu–2011 | Q fever, spotted fever group, and typhus group rickettsioses among hospitalized febrile patients in northern Tanzania | 2007—2008 | Seroprevalance study | Serological | Bacteria | 3 | Moderate |
| 151 | John–2010 | Quantifying risk factors for human brucellosis in rural northern Tanzania | 2002—2003 | Case Series | Serological | Bacteria | 1 | High |
| 153 | Caron–2012 | Recent introduction and rapid dissemination of Chikungunya virus and Dengue virus serotype 2 associated with human and mosquito coinfections in Gabon, central Africa | 2010—2010 | Fever Series | PCR | Viruses | 2 | Low |
| 154 | Thonnon–1998 | Re-emergence of yellow fever in Senegal in 1995 | 1995—1995 | Case Series | Serological | Viruses | 1 | High |
| 155 | Shoemaker–2012 | Reemerging Sudan Ebola virus disease in Uganda, 2011 | 2011—2011 | Case Series | PCR | Viruses | 1 | High |
| 156 | Rosenthal–1982 | Relapsing fever in Cape Town. A case report | 1982—1982 | Case Series | Culture | Bacteria | 1 | High |
| 157 | Ismail–2006 | Retrospective serosurvey of leptospirosis among patients with acute febrile illness and hepatitis in Egypt | 1999—2003 | Seroprevalance study | Serological | Bacteria | 1 | Moderate |
| 158 | Maina–2012 | Rickettsia felis infection in febrile patients, western Kenya, 2007-2010 | 2007—2008 | Fever Series | PCR | Bacteria | 1 | Low |
| 159 | Socolovschi–2010 | Rickettsia felis-associated uneruptive fever, Senegal | 2008—2009 | Fever Series | PCR | Bacteria | 1 | Low |
| 160 | Kaabia–2009 | Rickettsial infection in hospitalised patients in central Tunisia: report of 119 cases | 2000—2007 | Fever Series | Serological | Bacteria | 4 | Moderate |
| 162 | Sutherland–2011 | Serologic evidence of arboviral infections among humans in Kenya | 2000—2003 | Seroprevalance study | Serological | Viruses | 8 | Moderate |
| 163 | El Rhaffouli–2012 | Serologic evidence of West Nile virus infection among humans, Morocco | 2011—2011 | Seroprevalance study | Serological | Viruses | 1 | Moderate |
| 164 | Kaabia–2006 | Serologic study of rickettsioses among acute febrile patients in central Tunisia | 2004—2004 | Fever Series | Serological | Bacteria | 5 | Moderate |
| 165 | Tomori–1999 | Serologic survey among hospital and health center workers during the Ebola hemorrhagic fever outbreak in Kikwit, Democratic Republic of the Congo, 1995 | 1995—1995 | Fever Series | Serological | Viruses | 1 | Moderate |
| 168 | Klempa–2010 | Serological evidence of human hantavirus infections in Guinea, West Africa | 2005—2005 | Fever Series | Serological | Viruses | 1 | Moderate |
| 171 | Mease–2011 | Seroprevalence and distribution of arboviral infections among rural Kenyan adults: a cross-sectional study | 2004—2004 | Seroprevalance study | Serological | Viruses | 5 | Moderate |
| 172 | Kuniholm–2006 | Seroprevalence and distribution of Flaviviridae, Togaviridae, and Bunyaviridae arboviral infections in rural Cameroonian adults | 2000—2003 | Fever Series | Serological | Viruses | 7 | Moderate |
| 173 | Schwarz–2012 | Seroprevalence of antibodies against Chikungunya, Dengue, and Rift Valley fever viruses after febrile illness outbreak, Madagascar | 2010—2010 | Seroprevalance study | Serological | Viruses | 3 | Moderate |
| 175 | Vairo–2012 | Seroprevalence of dengue infection: a cross-sectional survey in mainland Tanzania and on Pemba Island, Zanzibar | 2007—2007 | Seroprevalance study | Serological | Viruses | 1 | Moderate |
| 176 | Lacheheb–2009 | Seroprevalence of Q-fever in Algeria | 1995—1996 | Fever Series | Serological | Bacteria | 1 | Moderate |
| 177 | Steinmann–2005 | Seroprevalence of Q-fever in febrile individuals in Mali | 2002—2003 | Fever Series | Serological | Bacteria | 2 | Moderate |
| 178 | Corwin–1992 | The prevalence of arboviral, rickettsial, and Hantaan-like viral antibody among schoolchildren in the Nile river delta of Egypt | 1989—1989 | Seroprevalance study | Serological | Bacteria/Viruses | 8 | Moderate |
| 179 | Blaylock–2011 | The seroprevalence and seroincidence of dengue virus infection in western Kenya | 2010—2010 | Seroprevalance study | Serological | Viruses | 1 | Moderate |
| 185 | Thonnon–1998 | Yellow fever outbreak in Kaffrine, Senegal 1996: epidemiological and entomological findings | 1996—1996 | Fever Series | Serological | Viruses | 1 | Moderate |
| 186 | Onyango–2004 | Yellow fever outbreak, Imatong, southern Sudan | 2003—2003 | Fever Series | Serological | Viruses | 1 | Moderate |
| 187 | World Health Organization–2010 | Yellow fever, Senegal | 2010—2010 | Case Series | Serological | Viruses | 1 | High |
| 192 | Adejuyigbe–2001 | Septicaemia in high risk neonates at a teaching hospital in ile-lfe nigeria | 1994—1995 | Fever Series | Not described | Bacteria | 7 | Unclear |
| 193 | BA–2010 | Hospital surveillance of childhood bacterial meningits in senegal and the introduction of haemophilus influenzae type b conjugate vaccine | 2002—2008 | Fever Series | Culture | Bacteria | 3 | Low |
| 194 | Cisse–2001 | Neonatal bacterial infections at the CUH of dakar | 1997—1998 | Fever Series | Culture | Bacteria | 7 | Low |
| 195 | Fonkoua–2001 | Bacterial meningitis in yaounde (cameroon) in 1999-2000 | 1999—2000 | Fever Series | Culture | Bacteria | 9 | Low |
| 196 | Hill–2007 | Bacteraemia in patients admitted to an urban hospital in west africa | 2003—2005 | Fever Series | Not described | Bacteria | 15 | Unclear |
| 197 | Neema Kayange–2010 | Predictors of positive blood culture and deaths amoung neaonates with suspected neonatal sepsis in a tertiary hospital mwanza-tanzania | 2009—2009 | Fever Series | Culture | Bacteria | 6 | Low |
| 198 | Kisakye–2009 | Surveillance for streptococcus pneumoniae meningitis in children aged <5years: implications for immunization in uganda | 2001—2006 | Fever Series | Culture | Bacteria | 4 | Low |
| 205 | Okabayashi–1999 | Short report: prevalence of antibodies against spotted fever, murine typhus, and Q fever rickettsiae in humans living in Zambia | 1989—1996 | Seroprevalance study | Serological | Bacteria | 3 | Moderate |
| 207 | M. Traore-Lamizana–1996 | Surveillance for yellow fever virus in eastern Senegal during 1993 | 1993—1993 | Seroprevalance study | Serological | Viruses | 1 | Moderate |
| 210 | Owa–1988 | Neonatal bacteraemia in Wesley Guild Hospital, Ilesha, Nigeria. | 1986—1986 | Fever Series | Not described | Bacteria | 10 | Unclear |
| 211 | Owusu–2012 | Aetiological agents of cerebrospinal meningits: a retrospective study from teaching hospital in Ghana | 2008—2010 | Fever Series | Not described | Bacteria/Fungi | 13 | Unclear |
| 212 | RAYNER–1988 | Community aquired bacteria bacteremia: a prospective survey of 239 cases. | 1986—1987 | Fever Series | Culture | Bacteria | 13 | Low |
| 213 | Roca–2009 | Surveillance of acute bacterial meningitis among children admitted to a district hospital in rural Mozambique. | 2006—2007 | Fever Series | Culture | Bacteria | 10 | Low |
| 214 | Onipede–2009 | Invasive bacteria isolates from children with severe infections in a Nigeria hospital | 2005—2006 | Fever Series | Culture | Bacteria | 10 | Low |
| 215 | Samuel–2006 | Bacterial isolates of blood cultures in patients with suspected septicaemia in Ilorin, Nigeria. | 2003—2003 | Fever Series | Not described | Bacteria | 10 | Unclear |
| 216 | Schwarz–2010 | Systemic bacteraemiain children presenting with clinical pneumonia and the impact of non-typhoid salmonella (NTS). | 2007—2009 | Fever Series | Culture | Bacteria | 12 | Low |
| 217 | SigaÃºque–2008 | Acute bacterial meningitis among children, in Manhica, a rural area in Southern Mozambique. | 1998—2003 | Fever Series | Not described | Bacteria | 9 | Unclear |
| 218 | Molyneux–1998 | Acute bacterial meningitis in children admitted to the Queen Elizabeth Central Hospital, Blantyre Malawi in 1996-97. | 1996—1997 | Fever Series | Not described | Bacteria | 5 | Unclear |
| 219 | Muhe L–1999 | Etiology of pneumonia, sepsis and meningitis in infants younger than three months of age in Ethiopia. | 1991—1993 | Fever Series | Culture | Bacteria | 8 | Low |
| 220 | Mullan–2011 | Etiology of meningitis among patients among patients admitted to a tertiary referral hospital in Botswana. | 2000—2008 | Fever Series | Culture | Bacteria/Fungi | 10 | Low |
| 221 | Mwaniki–2011 | Clinical indicators of bacterial meningitis among neonates and young infants in rural Kenya. | 2001—2005 | Fever Series | Culture | Bacteria | 23 | Low |
| 223 | Ayoola–2002 | Predictors of bacteraemia among febrile infants in Ibadan, Nigeria. | 1998—1998 | Fever Series | Culture | Bacteria | 7 | Low |
| 224 | Bahwere–2001 | Community-acquired bacteremia among hospitalized children in rural central Africa. | 1989—1990 | Fever Series | Culture | Bacteria | 8 | Low |
| 225 | Talbert–2010 | Invasive bacterial infections in neonates and young infants born outside hospital admitted to a rural hospital in Kenya. | 2001—2009 | Fever Series | Culture | Bacteria | 26 | Low |
| 226 | Walsh–2000 | Bacteremia in febrile Malawian children: clinical and microbiologic features. | 1996—1997 | Fever Series | Culture | Bacteria | 7 | Low |
| 227 | Kohli-Kochhar–2011 | A ten-year review of neonatal bloodstreem infections in tertiary private hospital in Kenya. | 2000—2009 | Fever Series | Culture | Bacteria/Fungi | 18 | Low |
| 228 | Lepage–1987 | Community-acquired bacteraemia in African children. | 1984—1985 | Fever Series | Culture | Bacteria | 4 | Low |
| 229 | Maalej–2006 | Bacteriology of community acquired meningitis in Sfax, Tunisia (1993-2001) | 1993—2001 | Fever Series | Culture | Bacteria | 9 | Low |
| 230 | Meremikwu–2005 | Bacterial isolates of blood cultures of children with suspected septicaemia in Calabar, Nigeria. | 1996—2002 | Fever Series | Culture | Bacteria | 11 | Low |
| 231 | Mhada–2012 | Neonatal sepsis at Muhimbili National Hospital, Dar es Salaam, Tanzania; aetiology, antimicrobial sensitivity pattern and clinical outcome. | 2009—2010 | Fever Series | Culture | Bacteria | 7 | Low |
| 232 | Nadjm–2012 | Severe febrile illness in adult hospital admissions in Tanzania: a prospective study in an area of high malaria transmission. | 2007—2007 | Fever Series | Culture | Bacteria | 7 | Low |
| 233 | Nielsen–2012 | Incidence and characteristics of bacteremia among children in rural Ghana. | 2007—2009 | Fever Series | Culture | Bacteria | 13 | Low |
| 234 | Moyo–2010 | Bacteria isolated from bloodstream infections at a tertiary hospital in Dar es Salaam, Tanzania - antimicrobial resistance of isolates | 2005—2009 | Fever Series | Culture | Bacteria | 10 | Low |
| 235 | Mtove–2010 | Invasive salmonellosis among children admitted to a rural Tanzania hospital and comparison with previous studies. | 2008—2009 | Fever Series | Culture | Bacteria | 8 | Low |
| 236 | Nwadioha–2011 | Bacterial isolates in blood cultures of children with suspected septicaemia in Kano: a two year study. | 2006—2008 | Fever Series | Not described | Bacteria | 10 | Unclear |
| 241 | Brent–2006 | Incidence of clinically significant bacteraemia in children who present to hospital in Kenya: community-based observational study. | 2003—2003 | Fever Series | Culture | Bacteria | 9 | Low |
| 244 | Falade–2009 | Invasive pneumococcal disease in children aged <5 years admitted to 3 urban hospitals in Ibadan, Nigeria. | 2005—2007 | Fever Series | Culture | Bacteria | 8 | Low |
| 245 | Gordon–2001 | Bacteraemia and mortality among adult medical Hospital admissions in Malawi--predominance of non-typhi salmonellae and Streptococcus pneumoniae. | 1997—1998 | Fever Series | Culture | Bacteria/Fungi | 7 | Low |
| 249 | Petit–1995 | Bacteraemia in patients presenting with fever. | 1987—1992 | Fever Series | Not described | Bacteria/Fungi | 9 | Unclear |
| 365 | Abdelkefi–2006 | Detection of catheter-related bloodstream infections by the Gram stain-acridine orange leukocyte cytospin test in hematopoietic stem cell transplant recipients | 2002—2004 | Fever Series | Culture | Bacteria/Fungi | 9 | Low |
| 366 | Lhuillier–1985 | Rural epidemic of yellow fever with interhuman transmission in the Ivory Coast in 1982 | 1982—1982 | Fever Series | Serological | Viruses | 1 | Moderate |
| 367 | Dunster–2002 | First documentation of human Crimean-Congo hemorrhagic fever, Kenya | 2000—2000 | Case Series | Serological | Viruses | 1 | High |
| 368 | Ebong–1986 | Acute osteomyelitis in Nigerians with sickle cell disease | 1976—1981 | Case Series | Culture | Bacteria | 4 | High |
| 369 | Adejuyigbe–2004 | Bacterial isolates in the sick young infant in Ile-Ife, Nigeria | 2001—2001 | Fever Series | Culture | Bacteria | 8 | Low |
| 370 | Adekolu-John–1983 | Arthropod-borne virus antibodies in sera of residents of Kainji Lake Basin, Nigeria 1980 | 1980—1980 | Fever Series | Serological | Viruses | 6 | Moderate |
| 371 | Frame–1984 | Endemic Lassa fever in Liberia. II. Serological and virological findings in hospital patients | 1980—1982 | Fever Series | Serological | Viruses | 1 | Moderate |
| 372 | Fisher-Hoch–1992 | Risk of human infections with Crimean-Congo hemorrhagic fever virus in a South African rural community | 1984—1986 | Fever Series | Serological | Viruses | 1 | Moderate |
| 373 | Ghaffar–1991 | The impact of endemic schistosomiasis on acute viral hepatitis | 1983—1985 | Fever Series | Serological | Viruses | 2 | Moderate |
| 374 | Goel–1999 | Primary staphylococcal pneumonia in young children: a review of 100 cases | 1989—1995 | Fever Series | Culture | Bacteria | 1 | Low |
| 375 | Iregbu–2006 | Bacteriological profile of neonatal septicaemia in a tertiary hospital in Nigeria | 2002—2002 | Fever Series | Culture | Bacteria | 10 | Low |
| 376 | Kiwanuka–2001 | Childhood bacterial meningitis in Mbarara Hospital, Uganda: antimicrobial susceptibility and outcome of treatment | 1999—1999 | Fever Series | Culture | Bacteria/Fungi | 9 | Low |
| 377 | Kiwanuka–1999 | O'nyong-nyong fever in south-central Uganda, 1996-1997: clinical features and validation of a clinical case definition for surveillance purposes | 2010—2010 | Fever Series | Culture/Serological | Bacteria/Viruses | 6 | Low |
| 378 | AILAL–2004 | Forty-one pediatric cases of non-typhoidal salmonellosis (English) | 1994—2002 | Case Series | Culture | Bacteria | 1 | High |
| 379 | ALIOUA–2003 | Manifestations neurologiques de la fiÃ¨vre boutonneuse mÃ©diterranÃ©enne: Ã  propos de quatre observations | 2000—2000 | Case Series | Serological | Bacteria | 1 | High |
| 380 | AMENZOUY–2010 | Manifestations neurologiques de la fiÃ¨vre boutonneuse mÃ©diterranÃ©enne: Ã  propos de deux observations pÃ©diatriques | 2009—2009 | Case Series | Serological | Bacteria | 1 | High |
| 381 | World health Organization–2007 | Outbreak of Marburg haemorrhagic fever: Uganda, June-August 2007 | 2007—2007 | Case Series | Serological | Viruses | 1 | High |
| 382 | Brink–2007 | Antimicrobial susceptibility profile of selected bacteraemic pathogens from private institutions in South Africa | 2006—2006 | Case Series | Culture | Bacteria | 8 | High |
| 383 | Woodruff–1988 | A study of viral and rickettsial exposure and causes of fever in Juba, southern Sudan | 1986—1986 | Seroprevalance study | Serological | Bacteria/Viruses | 11 | Moderate |
| 384 | Bajani–1997 | A survey for antibodies to Lassa virus among health workers in Nigeria | 1992—1993 | Seroprevalance study | Serological | Viruses | 1 | Moderate |
| 385 | Olaleye–1990 | A survey for haemagglutination-inhibiting antibody to West Nile virus in human and animal sera in Nigeria | 1989—1989 | Fever Series | Serological | Viruses | 1 | Moderate |
| 386 | Schelling–2003 | Brucellosis and Q-fever seroprevalences of nomadic pastoralists and their livestock in Chad | 1999—2000 | Seroprevalance study | Serological | Bacteria | 2 | Moderate |
| 387 | Okware–2002 | An outbreak of Ebola in Uganda | 2000—2001 | Fever Series | Serological | Viruses | 1 | Moderate |
| 388 | Cotton–1992 | Bacteraemia in children in the south western cape a hospital based survey | 1989—1989 | Case Series | Culture | Bacteria | 6 | High |
| 389 | Misallati–2000 | Blood-culture-proven neonatal septicaemia: a review of 36 cases. | 1997—1998 | Case Series | Culture | Bacteria | 7 | High |
| 390 | Afifi–2005 | Hospital-based surveillance for acute febrile illness in Egypt: a focus on community-acquired bloodstream infections. | 1999—2003 | Fever Series | Serological | Bacteria | 3 | Moderate |
| 391 | Organisation Mondiale de la SantÃ©–2000 | FiÃ¨vre jaune, 1998-1999. | 1998—1999 | Case Series/Fever Series | Serological | Viruses | 1 | High |
| 392 | Organisation Mondiale de la SantÃ©–2001 | FlambÃ©e de fiÃ¨vre hÃ©morragique Ã  virus Ebola, Ouganda, aoÃ»t 2000-janvier 2001 | 2000—2001 | Case Series | Serological | Viruses | 1 | High |
| 393 | Adam–2008 | Case report: Rift Valley Fever with vertical transmission in a pregnant Sudanese woman | 2007—2007 | Case Series | Serological | Viruses | 1 | High |
| 394 | Ahmed–2002 | Pasteurella gallinarum neonatal meningitis | 2001—2001 | Case Series | Culture | Bacteria | 1 | High |
| 395 | World Health Organization–2008 | Yellow fever in Africa and South America, 2006 | 2006—2007 | Case Series/Fever Series | Serological | Viruses | 1 | High |
| 396 | Ashour–2007 | Microbial spectrum and antibiotic susceptibility profile of gram-positive aerobic bacteria isolated from cancer patients | 2007—2007 | Case Series | Culture | Bacteria | 5 | High |
| 397 | ATTOH-TOURE–2010 | RÃ©surgence des Ã©pidÃ©mies de fiÃ¨vre jaune en CÃ´te-d'Ivoire | 2001—2007 | Case Series | Serological | Viruses | 1 | High |
| 398 | Baudon–1986 | [The 1983 yellow fever epidemic in Burkina Faso] | 1983—1983 | Case Series | Serological | Viruses | 1 | High |
| 399 | Bausch–2003 | Risk factors for Marburg hemorrhagic fever, Democratic Republic of the Congo | 1998—2000 | Case Series | Serological | Viruses | 1 | High |
| 400 | Bellaoui–2009 | BactÃ©riÃ©mies Ã  Kluyvera : Ã  propos d'une Ã©pidÃ©mie dans un CHU au Maroc | 2005—2005 | Case Series | Culture | Bacteria | 2 | High |
| 401 | Ben Brahim–2014 | [Pericardial tamponade revealing systemic lupus erythematosus during the course of atypical visceral leishmaniasis] | 2003—2003 | Case Series | Serological | Bacteria | 1 | High |
| 402 | BEN SALEM–2003 | Une insuffisance rÃ©nale aiguÃ« obstructive rÃ©vÃ©lant une leishmaniose viscÃ©rale chez un diabÃ©tique | 2001—2001 | Case Series | Serological | Parasites | 1 | High |
| 403 | Borchert–2002 | Short communication: a cluster of Marburg virus disease involving an infant | 1999—1999 | Case Series/Fever Series | Serological | Viruses | 1 | High |
| 404 | Bosch–2008 | Successful treatment of Chromobacterium violaceum sepsis in South Africa | 2007—2007 | Case Series | Culture/Serological | Bacteria | 3 | High |
| 405 | Bouallegue-Godet–2005 | Nosocomial outbreak caused by Salmonella enterica serotype Livingstone producing CTX-M-27 extended-spectrum beta-lactamase in a neonatal unit in Sousse, Tunisia | 2002—2002 | Case Series | Culture | Bacteria | 1 | High |
| 406 | BOUANANI–2013 | Fusariose dissÃ©minÃ©e au cours d'une autogreffe de moelle osseuse | 2010—2010 | Case Series | Culture | Fungi | 1 | High |
| 407 | BOUAYED–2012 | Polyarthrite aiguÃ« fÃ©brile rÃ©vÃ©lant une mÃ©ningococcÃ©mie: Ã  propos d'un cas | 2011—2011 | Case Series | Culture | Bacteria | 1 | High |
| 408 | BOUMANDOUKI–2007 | Orthopoxvirose simienne (ou variole du singe) : Ã©tude de 8 cas observÃ©s Ã  l'hÃ´pital d'lmpfondo de la RÃ©publique du Congo | 2003—2003 | Case Series | Serological | Viruses | 1 | High |
| 409 | BOUMANDOUKI–2005 | Prise en charge des malades et des dÃ©funts lors de l'Ã©pidÃ©mie de fiÃ¨vre hÃ©morragique due au virus Ebola d'octobre Ã  dÃ©cembre 2003 au Congo. | 2003—2003 | Case Series | Serological | Viruses | 1 | High |
| 410 | BOUZENOUNE–2011 | SensibilitÃ© aux antibiotiques des Salmonella enterica sÃ©rotype Typhi isolÃ©es des hÃ©mocultures Ã  l'hÃ´pital d'Ain M'lila (AlgÃ©rie), entre 2005 et 2008 | 2005—2008 | Case Series | Culture | Bacteria | 1 | High |
| 411 | Burdge–1985 | Serious Pasteurella multocida infections from lion and tiger bites | 1985—1985 | Case Series | Culture | Bacteria | 1 | High |
| 412 | Cardinale–2001 | Dual emergence in food and humans of a novel multiresistant serotype of Salmonella in Senegal: Salmonella enterica subsp. enterica serotype 35:c:1,2 | 2000—2000 | Case Series | Culture | Bacteria | 1 | High |
| 413 | CENAC–2008 | DYSFONCTION VENTRICULAIRE AIGUÃ‹ FÃ‰BRILE Ã€ DAKAR DONNÃ‰ES CLINIQUES ET ENQUÃŠTE SÃ‰ROLOGIQUE | 2004—2005 | Case Series | Serological | Bacteria/Viruses | 6 | High |
| 414 | Centers for Disease Control (CDC).–1984 | Congo-Crimean hemorrhagic fever--Republic of South Africa. | 1983—1984 | Case Series | Serological | Viruses | 1 | High |
| 415 | Chekir–1996 | La pyomyosite chez l'adulte en Tunisie centrale. A propos de dix cas. | 1988—1994 | Case Series | Culture | Bacteria | 1 | High |
| 416 | Chihab–2004 | Chryseomonas luteola identified as the source of serious infections in a Moroccan University Hospital | 2003—2003 | Case Series | Culture | Bacteria | 1 | High |
| 417 | Chouchane–2004 | Les fievres prolongees de l'enfant. Etude retrospective de 67 cas. | 1988—1998 | Case Series | Culture | Bacteria | 2 | High |
| 418 | Cohen–1996 | Tick bite fever in black South Africans--a rare disease? | Not stated | Case Series | Serological | Bacteria | 1 | High |
| 419 | Conteh–1983 | Leishmaniasis in The Gambia. I. A case of cutaneous leishmaniasis and a case of visceral leishmaniasis | 1980—1980 | Case Series | Serological | Parasites | 1 | High |
| 420 | Coovadia–1983 | Vibrio cholerae bacteraemia in a newborn infant. A case report | 1982—1982 | Case Series | Culture | Bacteria | 1 | High |
| 421 | Coovadia–1986 | Meningitis due to beta-lactamase producing, chloramphenicol-resistant Haemophilus influenzae type b, in South Africa | 1985—1985 | Case Series | Culture | Bacteria | 1 | High |
| 422 | Coovadia–1992 | Multiresistant Klebsiella pneumoniae in a neonatal nursery: the importance of maintenance of infection control policies and procedures in the prevention of outbreaks | 1989—1989 | Case Series | Culture | Bacteria | 1 | High |
| 423 | Cutler–1999 | Successful in vitro cultivation of Borrelia duttonii and its comparison with Borrelia recurrentis | 1998—1998 | Fever Series | Serological | Bacteria | 1 | Moderate |
| 424 | DOUMBE–2003 | FiÃ¨vre typhoparatyphoÃ¯de chez l'enfant au cameroun: aspects cliniques, biologiques et Ã©volutifs d'une sÃ©rie de 65 cas Ã  YaoundÃ© | 1987—1990 | Case Series | Serological | Bacteria | 2 | High |
| 425 | Dube–1983 | Candida septicaemia in a newborn complicating bacterial bronchopneumonia | 1983—1983 | Case Series | Culture | Bacteria | 1 | High |
| 426 | EL AMERI–2011 | Tableau de pyÃ©lonÃ©phrite chez une femme enceinte: penser Ã  la listÃ©riose | 2010—2010 | Case Series | Not described | Bacteria | 1 | High |
| 427 | ELBELDI–2011 | Les infections Ã  Listeria monocytogenes Ã  Tunis: Ã  propos de sept cas | 2000—2008 | Case Series | Culture | Bacteria | 1 | High |
| 428 | ELMAATAOUI–2009 | Ã‰pidÃ©miologie des isolats d'hÃ©mocultures : expÃ©rience d'un service d'hÃ©matologie clinique | 2006—2008 | Case Series | Culture | Bacteria/Fungi | 12 | High |
| 429 | World Health Organization–1996 | La fiÃ¨vre jaune en 1994 et 1995. | 1994—1995 | Case Series/Fever Series | Serological | Viruses | 1 | High |
| 430 | Organisation Mondiale de la SantÃ©.–1998 | Une flambÃ©e de fiÃ¨vre de la vallÃ©e du Rift en Afrique orientale, 1997-1998 | 1997—1998 | Fever Series | Serological | Viruses | 1 | Moderate |
| 431 | Abdel-Aziz–1980 | Rift Valley fever as a possible cause of human abortions | 1977—1978 | Fever Series | Serological | Viruses | 1 | Moderate |
| 432 | Adegbola–2006 | Serotype and antimicrobial susceptibility patterns of isolates of Streptococcus pneumoniae causing invasive disease in The Gambia 1996-2003 | 1997—2003 | Fever Series | Culture | Bacteria | 1 | Low |
| 433 | AGBENU–2010 | Evaluation of the Widal-Felix serodiagnostic test in Togo (English) | 2005—2006 | Fever Series | Serological | Bacteria | 1 | Moderate |
| 434 | AISSAOUI–2010 | Central venous catheter-related bacteraemia: Prospective study in a Moroccan medical intensive care unit (English)Central venous catheter-related bacteraemia: Prospective study in a Moroccan medical intensive care unit (English) | 2009—2009 | Fever Series | Culture | Bacteria/Fungi | 8 | Low |
| 435 | AKOUA-KOFFI–2006 | DÃ©tection des anticorps anti-virus de lassa dans l'ouest forestier de la CÃ´te d'Ivoire | 2000—2000 | Fever Series | Serological | Viruses | 1 | Moderate |
| 436 | Allen–1984 | Lobar pneumonia in Northern Zambia: clinical study of 502 adult patients | 1981—1983 | Fever Series | Culture | Bacteria | 1 | Low |
| 437 | AMONKOU–1996 | PrÃ©lÃ¨vement bronchique protÃ©gÃ© systÃ©matique et hÃ©moculture : l'apport de chacun au cours des pneumopathies nosocomiales | 1995—1995 | Fever Series | Culture | Bacteria/Fungi | 5 | Low |
| 438 | World Health Organization–2007 | Outbreaks of Rift Valley fever in Kenya, Somalia and United Republic of Tanzania, December 2006-April 2007 | 2006—2007 | Fever Series | Serological | Viruses | 1 | Moderate |
| 439 | World Health Organization–2006 | Yellow fever situation in Africa and South America, 2005 | 2005—2005 | Seroprevalance study | Serological | Viruses | 1 | Moderate |
| 440 | World Health Organization–2005 | Marburg haemorrhagic fever, Angola--update | 2005—2005 | Fever Series | Serological | Viruses | 1 | Moderate |
| 441 | BEN HAMOUDA–2013 | Aspects cliniques et Ã©volutifs des mÃ©ningites bactÃ©riennes nÃ©onatales | 1996—2010 | Fever Series | Culture | Bacteria | 6 | Low |
| 442 | BEN JEMAA–2004 | Profil bactÃ©riologique des bactÃ©riÃ©mies et sensibilitÃ© aux antibiotiques des bactÃ©ries en cause dans la rÃ©gion de Sfax (1993-1998) | 1993—1998 | Fever Series | Culture | Bacteria | 23 | Low |
| 443 | Johnson–1996 | Acute bronchiolitis in tropical Africa: a hospital-based perspective in Ibadan, Nigeria | 1985—1987 | Fever Series | Culture | Bacteria | 4 | Low |
| 444 | Sharp–1982 | Lassa fever in children | 1982—1982 | Fever Series | Serological | Viruses | 1 | Moderate |
| 445 | BENKORTBI–1992 | Les fiÃ¨vres thyphoÃ¯des compliquÃ©es de l'enfant. Approche Ã©pidÃ©miologique, clinique et thÃ©rapeutique | 1975—1990 | Fever Series | Culture | Bacteria | 1 | Low |
| 446 | BENKORTBI–1999 | La fiÃ¨vre typhoÃ¯de Ã  l'hÃ´pital de MÃ©dÃ©a (AlgÃ©rie, 1994-1996). A propos de 111 cas | 1994—1996 | Fever Series | Culture | Bacteria | 1 | Low |
| 447 | Berkley–2004 | Indicators of acute bacterial meningitis in children at a rural Kenyan district hospital | 2001—2001 | Fever Series | Culture | Bacteria | 4 | Low |
| 448 | Blomberg–2005 | High rate of fatal cases of pediatric septicemia caused by gram-negative bacteria with extended-spectrum beta-lactamases in Dar es Salaam, Tanzania | 2001—2002 | Fever Series | Culture | Bacteria | 7 | Low |
| 449 | Blomberg–2007 | Antimicrobial resistance predicts death in Tanzanian children with bloodstream infections: a prospective cohort study | Not stated | Fever Series | Culture | Bacteria/Fungi | 31 | Low |
| 450 | Blomberg–2004 | Surveillance of antimicrobial resistance at a tertiary hospital in Tanzania | 1998—1999 | Case Series | Culture | Bacteria | 14 | High |
| 451 | Borchert–2006 | Serosurvey on household contacts of Marburg hemorrhagic fever patients | 1998—2000 | Fever Series | Serological | Viruses | 1 | Moderate |
| 452 | Ikumapayi–2007 | Molecular epidemiology of community-acquired invasive non-typhoidal Salmonella among children aged 2 29 months in rural Gambia and discovery of a new serovar, Salmonella enterica Dingiri | 2000—2004 | Case Series | Culture | Bacteria | 8 | High |
| 453 | Mouffok–2008 | Murine typhus, Algeria | 2004—2005 | Fever Series | Serological | Bacteria | 1 | Moderate |
| 454 | SISSOKO–2009 | Ã‰mergence chez l'homme de la fiÃ¨vre de la vallÃ©e du Rift Ã  Mayotte, 2007-2008 | 2007—2008 | Fever Series | Serological | Viruses | 1 | Moderate |
| 455 | Moore–2005 | Neonatal sepsis in Egypt associated with bacterial contamination of glucose-containing intravenous fluids | 2001—2001 | Fever Series | Culture | Bacteria | 4 | Low |
| 456 | Emmerich–2008 | Strain-specific antibody response to Lassa virus in the local population of west Africa | 2007—2007 | Fever Series | Serological | Viruses | 1 | Moderate |
| 457 | Monson–1984 | Endemic Lassa fever in Liberia. I. Clinical and epidemiological aspects at Curran Lutheran Hospital, Zorzor, Liberia | 1980—1982 | Case Series | Serological | Viruses | 1 | High |
| 458 | Salih–1990 | Endemic bacterial meningitis in Sudanese children: aetiology, clinical findings, treatment and short-term outcome | 1985—1986 | Fever Series | Culture | Bacteria | 7 | Low |
| 459 | Centers for Disease Control and Prevention–1998 | An outbreak of Rift Valley Fever, eastern Africa, 1997-1998 | 1997—1998 | Case Series/Fever Series | Serological | Viruses | 1 | High |
| 460 | REDJAH–1992 | EpidÃ©mie de fiÃ¨vre thphoÃ¯de de Dergana (banlieue d'Alger). Aspects cliniques et Ã©volutifs de la maladie chez l'enfant | 1990—1991 | Fever Series | Culture | Bacteria | 1 | Low |
| 461 | Itah–2004 | Correlation studies on Widal agglutination reaction and diagnosis of typhoid fever | 2003—2003 | Fever Series | Serological | Bacteria | 1 | Moderate |
| 462 | Laving–2003 | Neonatal bacterial meningitis at the newborn unit of Kenyatta National Hospital | 1999—1999 | Fever Series | Culture | Bacteria | 6 | Low |
| 463 | BOURROUS–2010 | SÃ©roprÃ©valence de l'hÃ©patite virale A dans les ictÃ¨res fÃ©briles chez les enfants de la rÃ©gion de Marrakech, Maroc | 2006—2008 | Fever Series | Serological | Viruses | 1 | Moderate |
| 464 | BOUSKRAOUI–2000 | Les complications de la fiÃ¨vre typhoÃ¯de chez l'enfant | 1981—1997 | Case Series/Fever Series | Culture | Bacteria | 2 | High |
| 465 | Brahim–2005 | Identifying relapsing fever Borrelia, Senegal | 1999—2003 | Fever Series | Culture | Bacteria | 2 | Low |
| 466 | Brahmi–2007 | Epidemiology and risk factors for colonization and infection by Acinetobacter baumannii in an ICU in Tunisia, where this pathogen is endemic | 2004—2005 | Fever Series | Culture | Bacteria | 1 | Low |
| 467 | Brown–1988 | Clinical presentation of louse-borne relapsing fever among Ethiopian refugees in northern Somalia | 1986—1986 | Fever Series | Serological | Bacteria | 2 | Moderate |
| 468 | Burans–1994 | Threat of hepatitis E virus infection in Somalia during Operation Restore Hope | 1992—1993 | Fever Series | Serological | Viruses | 1 | Moderate |
| 469 | Burt–1994 | Serodiagnosis of Crimean-Congo haemorrhagic fever | 1980—1992 | Fever Series | Serological | Viruses | 1 | Moderate |
| 470 | Burt–1996 | Investigation of tick-borne viruses as pathogens of humans in South Africa and evidence of Dugbe virus infection in a patient with prolonged thrombocytopenia | 1980—1993 | Case Series | Serological | Viruses | 1 | High |
| 471 | Jouan–1989 | Analytical study of a Rift Valley fever epidemic | 1987—1987 | Fever Series | Serological | Viruses | 1 | Moderate |
| 472 | Sissoko–2008 | Outbreak of Chikungunya fever in Mayotte, Comoros archipelago, 2005-2006 | 2005—2005 | Fever Series | Serological | Viruses | 1 | Moderate |
| 473 | Sanders–1999 | O'nyong-nyong fever in south-central Uganda, 1996-1997: description of the epidemic and results of a household-based seroprevalence survey | 1997—1997 | Fever Series | Serological | Viruses | 1 | Moderate |
| 474 | Campbell–2004 | Invasive pneumococcal infections among hospitalized children in Bamako, Mali | 2002—2003 | Fever Series | Culture | Bacteria | 4 | Low |
| 475 | CETRE-SOSSAH–2009 | FiÃ¨vre de la VallÃ©e du Rift: aspects vÃ©tÃ©rinaires et impacts sur la santÃ© humaine | 2007—2008 | Fever Series | Serological | Viruses | 1 | Moderate |
| 476 | Chambers–1980 | Rift valley fever in abattoir workers | 1978—1978 | Seroprevalance study | Serological | Viruses | 1 | Moderate |
| 477 | Chapman–1991 | Risk factors for Crimean-Congo hemorrhagic fever in rural northern Senegal | 1989—1989 | Fever Series | Serological | Viruses | 1 | Moderate |
| 478 | CHEMLI–2006 | Les fiÃ¨vres prolongÃ©es de l'enfant : Ã  propos de 110 cas | 1993—2005 | Case Series/Fever Series | Culture/Serological | Bacteria/viruses/parasites | 6 | High |
| 479 | CHEMSI–2013 | La fiÃ¨vre du nouveau-nÃ©. Ã‰tude prospective | 2010—2010 | Fever Series | Culture | Bacteria/Viruses | 5 | Low |
| 480 | Cobey–2001 | Short report: Detection of borrelia (relapsing fever) in rural Ethiopia by means of the quantitative buffy coat technique | 1998—1998 | Fever Series | Serological | Bacteria | 1 | Moderate |
| 481 | Corwin–1993 | Community-based prevalence profile of arboviral, rickettsial, and Hantaan-like viral antibody in the Nile River Delta of Egypt | 1991—1991 | Fever Series | Serological | Bacteria/Viruses | 8 | Moderate |
| 482 | Darwish–1987 | Arboviral causes of non-specific fever and myalgia in a fever hospital patient population in Cairo, Egypt | 1984—1984 | Fever Series | Serological | Viruses | 3 | Moderate |
| 483 | de Beer–1991 | A killing disease epidemic among displaced Sudanese population identified as visceral leishmaniasis | 1988—1988 | Fever Series | Serological | Parasites | 1 | Moderate |
| 484 | DE CARSALADE–2010 | La typhoÃ¬de Ã  Mayotte en 2007-2008 | 2007—2008 | Fever Series | Culture | Bacteria | 1 | Low |
| 485 | Demby–1994 | Early diagnosis of Lassa fever by reverse transcription-PCR | 1991—1991 | Fever Series | Culture | Parasites | 1 | Low |
| 486 | DIOP–1997 | Profil infectieux chez le drÃ©panocytaire | 1994—1997 | Fever Series | Culture | Bacteria | 1 | Low |
| 487 | Divizia–1999 | HAV and HEV infection in hospitalised hepatitis patients in Alexandria, Egypt | 1993—1993 | Seroprevalance study | Serological | Viruses | 2 | Moderate |
| 488 | Dubroca–2001 | EnquÃªtes sur les foyers anciens et actuels de fiÃ¨vre de la VallÃ©e du Rift au SÃ©nÃ©gal auprÃ¨s des services vÃ©tÃ©rinaires et dans la bibliographie | 1987—1987 | Fever Series | Serological | Viruses | 1 | Moderate |
| 489 | Dupont–1995 | Prevalence of antibodies to Coxiella burnetti, Rickettsia conorii, and Rickettsia typhi in seven African countries | 1992—1992 | Fever Series | Serological | Bacteria | 3 | Moderate |
| 490 | El Gaafary–2005 | Surveillance of acute hepatitis C in Cairo, Egypt | 2002—2002 | Fever Series | Serological | Viruses | 3 | Moderate |
| 491 | el Mubarak–2000 | Serological and virological characterization of clinically diagnosed cases of measles in suburban Khartoum | 1997—1997 | Fever Series | Serological | Viruses | 1 | Moderate |
| 492 | El-Gilany–2006 | Bloodborne infections among student voluntary blood donors in Mansoura University, Egypt | 2002—2003 | Fever Series | Serological | Bacteria/Viruses | 3 | Moderate |
| 493 | el-Karaksy–1999 | Human fascioliasis in Egyptian children: successful treatment with triclabendazole | 1999—1999 | Fever Series | Serological | Parasites | 1 | Moderate |
| 494 | Ellabib–2004 | Changing pattern of neonatal bacteremia. Microbiology and antibiotic resistance | 1997—2001 | Fever Series | Culture | Bacteria/Fungi | 11 | Low |
| 495 | El-Mahallawy–2005 | Clinical and microbiologic determinants of serious bloodstream infections in Egyptian pediatric cancer patients: a one-year study | 1999—1999 | Fever Series | Culture | Bacteria/Fungi | 21 | Low |
| 496 | El-Mahallawy–2005 | Community respiratory viruses as a cause of lower respiratory tract infections following suppressive chemotherapy in cancer patients | 2003—2004 | Fever Series | Serological | Viruses | 4 | Moderate |
| 497 | El-Mahallawy–2006 | Evaluation of pan-fungal PCR assay and Aspergillus antigen detection in the diagnosis of invasive fungal infections in high risk paediatric cancer patients | 2003—2004 | Fever Series | Culture | Fungi | 5 | Low |
| 498 | Enweronu-Laryea–2007 | Changing pattern of bacterial isolates and antimicrobial susceptibility in neonatal infections in Korle Bu Teaching Hospital, Ghana | 1990—1991 | Case Series | Culture | Bacteria | 13 | High |
| 499 | Ibrahim–2002 | Measles in suburban Khartoum: an epidemiological and clinical study | 1997—1999 | Fever Series | Serological | Viruses | 1 | Moderate |
| 500 | LaBeaud–2008 | Interepidemic Rift Valley fever virus seropositivity, northeastern Kenya | 2006—2006 | Seroprevalance study | Serological | Viruses | 1 | Moderate |
| 501 | Nur–1999 | IgM antibodies in hospitalized children with febrile illness during an inter-epidemic period of measles, in Somalia | 1995—1995 | Seroprevalance study | Serological | Bacteria/Viruses | 6 | Moderate |
| 502 | Rodhain–1989 | Arbovirus infections and viral haemorrhagic fevers in Uganda: a serological survey in Karamoja district, 1984 | 1984—1984 | Fever Series | Serological | Viruses | 9 | Moderate |
| 503 | Srikantiah–2006 | Population-based surveillance of typhoid fever in Egypt | 2002—2002 | Fever Series | Culture | Bacteria | 6 | Low |
| 504 | Swanepoel–1983 | Crimean-congo hemorrhagic fever in South Africa | 1981—1981 | Fever Series | Serological | Viruses | 1 | Moderate |
| 505 | TALANI–1999 | PrÃ©valence des anticorps anti-fiÃ¨vres hÃ©morragiques d'origine virale dans la rÃ©gion du Pool (Congo-Brazzaville) | 1981—1981 | Fever Series | Serological | Viruses | 3 | Moderate |
| 506 | van den Bosch–2000 | Chikungunya fever as a risk factor for endemic Burkitt's lymphoma in Malawi | 1987—1989 | Fever Series | Serological | Viruses | 1 | Moderate |
| 507 | Vial–2006 | Incidence of tick-borne relapsing fever in west Africa: longitudinal study | 1990—2005 | Case Series | Culture | Bacteria | 1 | High |
| 508 | Watts–1994 | Arthropod-borne viral infections associated with a fever outbreak in the northern province of Sudan | 1989—1990 | Seroprevalance study | Serological | Viruses | 8 | Moderate |
| 509 | El-Nawawy–2006 | One year study of bacterial and fungal nosocomial infections among patients in pediatric intensive care unit (PICU) in Alexandria | 2003—2004 | Fever Series | Culture | Bacteria | 5 | Low |
| 510 | ELOUENNASS–2008 | Ã‰pidÃ©miologie et profil de sensibilitÃ© des isolats d'hÃ©moculture dans un service de rÃ©animation (2002-2005) | 2004—2005 | Fever Series | Culture | Bacteria/Fungi | 16 | Low |
| 511 | el-Shabrawi–1997 | Human fascioliasis: clinical features and diagnostic difficulties in Egyptian children | 1989—1995 | Fever Series | Serological | Parasites | 1 | Moderate |
| 512 | Fadeel–2006 | Rapid enzyme-linked immunosorbent assay for the diagnosis of human brucellosis in surveillance and clinical settings in Egypt | 1999—2003 | Fever Series | Culture | Bacteria | 1 | Low |
| 513 | Fagbami–1988 | Heterologous flavivirus infection-enhancing antibodies in sera of Nigerians | 2003—2004 | Seroprevalance study | Serological | Viruses | 2 | Moderate |
| 514 | FAYE–2007 | Approche intÃ©grÃ©e de la surveillance de la fiÃ¨vre jaune : Ã©tude pilote au SÃ©nÃ©gal en 2003-2004 | 2003—2004 | Seroprevalance study | Serological | Viruses | 1 | Moderate |
| 515 | Faye–2007 | Rift Valley fever outbreak with East-Central African virus lineage in Mauritania, 2003 | 2003—2003 | Fever Series | Serological | Viruses | 1 | Moderate |
| 516 | Feldman–1989 | Community-acquired pneumonia of diverse aetiology: prognostic features in patients admitted to an intensive care unit and a ""severity of illness"" core | 1982—1985 | Fever Series | Culture | Bacteria | 3 | Low |
| 517 | Fisher-Hoch–1995 | Review of cases of nosocomial Lassa fever in Nigeria: the high price of poor medical practice | 1989—1989 | Fever Series | Serological | Viruses | 1 | Moderate |
| 518 | Fisher-Hoch–1988 | Hematologic dysfunction in Lassa fever | 1985—1985 | Fever Series | Serological | Viruses | 1 | Moderate |
| 519 | FONTENILLE–1989 | Les arboviroses dans la rÃ©gion de Nosy-BÃ©, Madagascar. DonnÃ©es sÃ©rologiques et entomologiques | 1986—1986 | Seroprevalance study | Serological | Viruses | 16 | Moderate |
| 520 | Gbadero–1995 | Microbial inciters of acute asthma in urban Nigerian children | 1993—1995 | Fever Series | Culture | Bacteria | 2 | Low |
| 521 | Gbadoe–2008 | Les salmonelloses chez l'enfant au CHU Tokoin de Lome (Togo). | 1995—2004 | Case Series | Culture | Bacteria | 6 | High |
| 522 | Gonzalez–1989 | Antibody prevalence against haemorrhagic fever viruses in randomized representative Central African populations | 1985—1987 | Fever Series/Seroprevalence | Serological | Viruses | 6 | Moderate |
| 523 | Gonzalez–1990 | A fatal case of Crimean-Congo haemorrhagic fever in Mauritania: virological and serological evidence suggesting epidemic transmission | 1988—1988 | Fever Series | Serological | Viruses | 1 | Moderate |
| 524 | Gordon–2008 | Epidemics of invasive Salmonella enterica serovar enteritidis and S. enterica Serovar typhimurium infection associated with multidrug resistance among adults and children in Malawi | 1999—1999 | Fever Series | Culture | Bacteria | 1 | Low |
| 525 | Gould–2008 | An outbreak of yellow fever with concurrent chikungunya virus transmission in South Kordofan, Sudan, 2005 | 2005—2005 | Fever Series | Serological | Viruses | 4 | Moderate |
| 526 | Gould–1985 | Cryptococcosis in Zimbabwe | 1968—1983 | Fever Series | Culture | Fungi | 1 | Low |
| 527 | Gray–2007 | Invasive group B streptococcal infection in infants, Malawi | 2004—2005 | Fever Series | Culture | Bacteria | 1 | Low |
| 528 | Gray–2006 | Identification and characterization of ceftriaxone resistance and extended-spectrum beta-lactamases in Malawian bacteraemic Enterobacteriaceae | 2004—2005 | Fever Series | Culture | Bacteria | 1 | Low |
| 529 | Gubler–1986 | Dengue 3 virus transmission in Africa | 1984—1985 | Fever Series | Serological | Viruses | 3 | Moderate |
| 530 | Gunther–2001 | Antibodies to Lassa virus Z protein and nucleoprotein co-occur in human sera from Lassa fever endemic regions | 2000—2000 | Fever Series | Serological | Viruses | 1 | Moderate |
| 531 | Hammami–1991 | Nosocomial outbreak of acute gastroenteritis in a neonatal intensive care unit in Tunisia caused by multiply drug resistant Salmonella wien producing SHV-2 beta-lactamase | 1988—1988 | Fever Series | Culture | Bacteria | 1 | Low |
| 532 | Hassan–1996 | Parasitic causes of hepatomegaly in children | 1995—1995 | Fever Series | Serological | Parasites | 5 | Moderate |
| 533 | Helmick–1986 | No evidence for increased risk of Lassa fever infection in hospital staff | 1972—1983 | Fever Series | Serological | Viruses | 1 | Moderate |
| 534 | Heyman–1998 | Meningococcal meningitis among Rwandan refugees: diagnosis, management, and outcome in a field hospital | 1994—1994 | Fever Series | Serological | Bacteria | 1 | Moderate |
| 535 | Holliman–2007 | Epidemiology of invasive pneumococcal disease in Kumasi, Ghana | 2002—2005 | Fever Series | Culture | Bacteria | 1 | Low |
| 536 | Hyams–1986 | Evaluation of febrile patients in Port Sudan, Sudan: isolation of dengue virus | 1984—1984 | Fever Series | Culture/Serological | Bacteria | 2 | Low |
| 537 | Ishiwada–2001 | Vaccine-modified measles in previously immunized children in Accra, Ghana: clinical, virological and serological parameters | 2000—2000 | Seroprevalance study | Serological | Viruses | 1 | Moderate |
| 538 | Itah–2005 | Bacteria isolated from blood, stool and urine of typhoid patients in a developing country | 2005—2005 | Fever Series | Culture/Serological | Bacteria | 3 | Low |
| 539 | Ivanoff–1982 | Haemorrhagic fever in Gabon. I. Incidence of Lassa, Ebola and Marburg viruses in Haut-Ogooue | 1980—1980 | Fever Series | Serological | Viruses | 2 | Moderate |
| 540 | JAÃDANE–2005 | EntÃ©roviroses neuromÃ©ningÃ©es en Tunisie : profil Ã©pidÃ©miologique, clinique et Ã©volutif. Ã€ propos de 26 cas pÃ©diatriques | 2001—2002 | Fever Series | Serological | Viruses | 1 | Moderate |
| 541 | Johnson–2008 | Etiologic agents and outcome determinants of community-acquired pneumonia in urban children: a hospital-based study | 2004—2007 | Fever Series | Culture | Bacteria | 5 | Low |
| 542 | Johnson–1983 | Antibodies against haemorrhagic fever viruses in Kenya populations | 1982—1982 | Fever Series/Seroprevalence | Serological | Viruses | 5 | Moderate |
| 543 | Johnson–1986 | Seasonal variation in antibodies against Ebola virus in Kenyan fever patients | 1984—1985 | Fever Series | Serological | Viruses | 1 | Moderate |
| 544 | Johnson–1993 | Haemorrhagic fever virus activity in equatorial Africa: distribution and prevalence of filovirus reactive antibody in the Central African Republic | 1992—1992 | Fever Series | Serological | Viruses | 2 | Moderate |
| 545 | Jongen–1997 | Tick-borne relapsing fever and pregnancy outcome in rural Tanzania | 1985—1995 | Case Series | Culture | Bacteria | 1 | High |
| 546 | Saluzzo–1985 | Crimean-Congo haemorrhagic fever and Rift Valley fever in south-eastern Mauritania | 1984—1984 | Seroprevalance study | Serological | Viruses | 1 | Moderate |
| 547 | Sarthou–1989 | Isolation of Rift Valley fever virus from human peripheral blood mononuclear cells: Mauritanian epidemic | 1987—1987 | Fever Series/Seroprevalence | Culture/Serological | Viruses | 1 | Moderate |
| 548 | ZELLER–1998 | La fiÃ¨vre de la vallÃ©e du Rift Ã  Madagascar : risques d'infection pour le personnel d'abattoir Ã  Antananarivo | 1995—1995 | Seroprevalance study | Serological | Viruses | 1 | Moderate |
| 549 | Onyango–2008 | Multidrug resistance of Salmonella enterica serovars Typhi and Typhimurium isolated from clinical samples at two rural hospitals in Western Kenya | 2004—2005 | Fever Series | Culture | Bacteria | 1 | Low |
| 550 | Thabet–2007 | Mortalite par meningites a pneumocoque chez l'enfant. Facteurs pronostiques a propos d'une serie de 73 observations. | 1995—2002 | Fever Series | Culture | Bacteria | 1 | Low |
| 551 | Mohammed–2000 | A severe epidemic of meningococcal meningitis in Nigeria, 1996 | 1996—1996 | Fever Series | Culture/Serological | Bacteria | 3 | Low |
| 552 | Mugalu–2006 | Aetiology, risk factors and immediate outcome of bacteriologically confirmed neonatal septicaemia in Mulago hospital, Uganda | 2002—2002 | Fever Series | Culture | Bacteria | 14 | Low |
| 553 | Scott–2005 | Progressive increase in antimicrobial resistance among invasive isolates of Haemophilus influenzae obtained from children admitted to a hospital in Kilifi, Kenya, from 1994 to 2002 | 1994—2002 | Case Series | Culture | Bacteria | 1 | High |
| 554 | Wiysonge–2008 | Yellow fever control in Cameroon: where are we now and where are we going? | 2006—2006 | Fever Series | Serological | Viruses | 1 | Moderate |
| 555 | Kaouech–2008 | Pediatric visceral leishmaniasis diagnosis in Tunisia: comparative study between optimised PCR assays and parasitological methods | 2003—2005 | Fever Series | Culture/Serological | Parasites | 1 | Low |
| 556 | Kariuki–2006 | Invasive multidrug-resistant non-typhoidal Salmonella infections in Africa: zoonotic or anthroponotic transmission? | 2002—2004 | Fever Series | Culture | Bacteria | 8 | Low |
| 557 | Keenlyside–1983 | Case-control study of Mastomys natalensis and humans in Lassa virus-infected households in Sierra Leone | 1982—1982 | Fever Series | Serological | Viruses | 1 | Moderate |
| 558 | Kisinza–2003 | A newly identified tick-borne Borrelia species and relapsing fever in Tanzania | 2002—2002 | Fever Series | Culture/Serological | Bacteria | 2 | Low |
| 559 | Kizito–2007 | Bacteraemia in homozygous sickle cell disease in Africa: is pneumococcal prophylaxis justified? | 2001—2002 | Fever Series | Culture | Bacteria | 7 | Low |
| 560 | KOKO–1997 | MÃ©ningites Ã  salmonelles de l'enfant Ã  Libreville. Etude rÃ©trospective de neuf observations | 1989—1993 | Case Series | Culture | Bacteria | 4 | High |
| 561 | Towner–2004 | Rapid diagnosis of Ebola hemorrhagic fever by reverse transcription-PCR in an outbreak setting and assessment of patient viral load as a predictor of outcome | 2000—2001 | Fever Series | Serological | Viruses | 1 | Moderate |
| 562 | Nwobu–1986 | Phage-types and resistance pattern of Staphylococcus aureus, isolated from clinical specimens, to penicillin and cloxacillin in a Lagos hospital | 1985—1985 | Fever Series | Culture | Bacteria | 1 | Low |
| 563 | NAKOUNNE–2000 | Veille microbiologique : les fiÃ¨vres hÃ©morragiques virales en RÃ©publique centrafricaine; donnÃ©es sÃ©rologiques actualisÃ©es chez l'homme | 1997—1997 | Seroprevalance study | Serological | Viruses | 6 | Moderate |
| 564 | Rao–1997 | In vitro antibiotic sensitivity pattern of common bacterial isolates from cases of acute bacterial meningitis with special reference to ceftriaxone | 1994—1995 | Fever Series | Culture | Bacteria | 3 | Low |
| 565 | Whistler–1989 | Characterization of Palyam serogroup orbiviruses isolated in South Africa and serologic evidence for their widespread distribution in the country | 1983—1987 | Fever Series | Serological | Viruses | 1 | Moderate |
| 566 | Adu–1997 | Measles outbreak in Ibadan: clinical, serological and virological identification of affected children in selected hospitals | 1997—1997 | Fever Series | Serological | Viruses | 1 | Moderate |
| 567 | LaBeaud–2007 | Spectrum of Rift Valley fever virus transmission in Kenya: insights from three distinct regions | 1997—1997 | Seroprevalance study | Serological | Viruses | 1 | Moderate |
| 568 | Ladhani–2004 | Bacteraemia due to Staphylococcus aureus | 1996—2001 | Fever Series | Culture | Bacteria | 1 | Low |
| 569 | Lasme Guillao–2011 | Infections a Klebsiella pneumonia et Enterobacter cloacae en nÃ©onatologie a Abidjan | 2011—2011 | Fever Series | Culture | Bacteria | 2 | Low |
| 570 | LEFEBVRE–2005 | Aspects clinique et biologique de la fiÃ¨vre typhoÃ¯de au SÃ©hÃ©gal : Ã‰tude de 70 cas | 1995—2002 | Fever Series | Culture | Bacteria | 1 | Low |
| 571 | LHUILLIER–1986 | Emergence endÃ©mique de la fiÃ¨vre jaune en CÃ´te d'Ivoire: place de la dÃ©tection des IgM antiamariles dans la stratÃ©gie de surveillance | 1982—1982 | Seroprevalance study | Serological | Viruses | 1 | Moderate |
| 572 | LHUILLIER–1983 | IntÃ©rÃªt des IgM antiamariles dans le diagnostic et la surveillance Ã©pidÃ©miologique de la fiÃ¨vre jaune | 1982—1982 | Fever Series | Serological | Viruses | 1 | Moderate |
| 573 | Logan–1992 | Rift Valley fever antibody in human sera collected after an outbreak in domestic animals in Kenya | 1992—1992 | Seroprevalance study | Serological | Viruses | 1 | Moderate |
| 574 | LOUSSAIEF–2005 | Les abcÃ¨s de la rate Ã  pyogÃ¨nes. Ã€ propos de 8 cas | 1993—2002 | Fever Series | Culture | Bacteria | 1 | Low |
| 575 | MAOULAININE–2012 | EpidÃ©miologie de l'infection nosocomiale bactÃ©rienne dans un service de rÃ©animation nÃ©onatale marocain | 2009—2010 | Fever Series | Culture | Bacteria | 11 | Low |
| 576 | Mathiot–1989 | Antibodies to haemorrhagic fever viruses in Madagascar populations | 1989—1989 | Fever Series/Seroprevalence | Serological | Viruses | 3 | Moderate |
| 577 | McCormick–1987 | A prospective study of the epidemiology and ecology of Lassa fever | 1985—1985 | Fever Series/Seroprevalence | Serological | Viruses | 1 | Moderate |
| 578 | MEUNIER–1987 | Surveillance sÃ©rologique des fiÃ¨vres hÃ©morragiques virales dans le Haut-Ogooue | 1985—1985 | Seroprevalance study | Serological | Viruses | 4 | Moderate |
| 579 | MILLELIRI–2004 | Les Ã©pidÃ©mies de fiÃ¨vre hÃ©morragique due au virus Ebola au Gabon (1994 - 2002) : Aspects Ã©pidÃ©miologiques et rÃ©flexions sur les mesures de contrÃ´le. | 2001—2002 | Fever Series | Serological | Viruses | 1 | Moderate |
| 580 | Morvan–1991 | Rift Valley fever on the east coast of Madagascar | 1990—1990 | Seroprevalance study | Serological | Viruses | 1 | Moderate |
| 581 | Mouffok–2006 | Reemergence of rickettsiosis in Oran, Algeria | 2004—2004 | Fever Series | Serological | Bacteria | 1 | Moderate |
| 582 | Mukonyora–1985 | Staphylococcal bacteraemia in Zimbabwe 1983 | 1983—1983 | Fever Series | Culture | Bacteria | 7 | Low |
| 583 | Musoke–2000 | Emergence of multidrug-resistant gram-negative organisms in a neonatal unit and the therapeutic implications | 1997—1998 | Fever Series | Culture | Bacteria | 8 | Low |
| 584 | Muyembe-Tamfum–1999 | Ebola outbreak in Kikwit, Democratic Republic of the Congo: discovery and control measures | 1995—1995 | Fever Series | Serological | Viruses | 1 | Moderate |
| 585 | Mwangi–2002 | Acute bacterial meningitis in children admitted to a rural Kenyan hospital: increasing antibiotic resistance and outcome | 1994—2000 | Fever Series | Culture | Bacteria | 5 | Low |
| 586 | Nabeth–2001 | Rift Valley fever outbreak, Mauritania, 1998: seroepidemiologic, virologic, entomologic, and zoologic investigations | 1998—1998 | Seroprevalance study | Serological | Viruses | 1 | Moderate |
| 587 | Nabeth–2004 | Crimean-Congo hemorrhagic fever, Mauritania | 2003—2003 | Seroprevalance study | Serological | Viruses | 1 | Moderate |
| 588 | Nantanda–2008 | Bacterial aetiology and outcome in children with severe pneumonia in Uganda | 2005—2006 | Fever Series | Culture | Bacteria | 5 | Low |
| 589 | Nathoo–1993 | Severe Klebsiella infection as a cause of mortality in neonates in Harare, Zimbabwe: evidence from postmortem blood cultures | 1991—1991 | Fever Series | Culture | Bacteria/Fungi | 16 | Low |
| 590 | NDIAYE–2011 | Aspects Ã©pidÃ©miologiques, cliniques et Ã©chographiques des endocardites infectieuses du coeur droit au SÃ©nÃ©gal : 6 observations | 2007—2010 | Fever Series | Culture | Bacteria | 1 | Low |
| 591 | Ndip–2004 | Acute spotted fever rickettsiosis among febrile patients, Cameroon | 2001—2001 | Seroprevalance study | Serological | Bacteria/Viruses | 6 | Moderate |
| 592 | Ndip–2004 | Detection of Rickettsia africae in patients and ticks along the coastal region of Cameroon | 2003—2003 | Fever Series | Serological | Bacteria | 1 | Moderate |
| 593 | Niklasson–1987 | Rift Valley fever: a sero-epidemiological survey among pregnant women in Mozambique | 1981—1983 | Seroprevalance study | Serological | Viruses | 1 | Moderate |
| 594 | Norton–2004 | Clinical predictors of bloodstream infections and mortality in hospitalized Malawian children | 1998—1998 | Fever Series | Culture | Bacteria/Fungi | 6 | Low |
| 595 | Nottidge–1985 | Haemophilus influenzae meningitis: a 5-year study in Ibadan, Nigeria | 1976—1980 | Case Series | Culture | Bacteria | 3 | High |
| 596 | Nsanze–1981 | Genital ulcers in Kenya. Clinical and laboratory study | 1980—1980 | Fever Series | Serological | Bacteria | 1 | Moderate |
| 597 | OMEZZINE-LETAÃEF–1997 | Etude sÃ©roÃ©pidÃ©miologique chez 300 malades fÃ©briles hospitalisÃ©s dans un service de mÃ©decine et maladies infectieuses | 1993—1994 | Seroprevalance study | Serological | Bacteria | 4 | Moderate |
| 598 | Omilabu–1990 | Yellow fever haemagglutination-inhibiting, neutralising and IgM antibodies in vaccinated and unvaccinated residents of Ibadan, Nigeria | 1990—1990 | Fever Series | Serological | Viruses | 3 | Moderate |
| 599 | Onyango–2007 | Laboratory diagnosis of Ebola hemorrhagic fever during an outbreak in Yambio, Sudan, 2004 | 2004—2004 | Seroprevalance study | Serological | Viruses | 1 | Moderate |
| 600 | Organisation Mondiale de la SantÃ©.–1998 | FiÃ¨vre jaune, 1996-1997. Partie 1 | 1997—1997 | Seroprevalance study | Serological | Viruses | 1 | Moderate |
| 601 | Ostroff–1996 | Resistance patterns of Streptococcus pneumoniae and Haemophilus influenzae isolates recovered in Egypt from children with pneumonia. The Antimicrobial Resistance Surveillance Study Group | 1991—1993 | Fever Series | Culture | Bacteria | 2 | Low |
| 602 | Ouedraogo–2011 | Aspects Ã©pidÃ©miologiques, microbiologiques et Ã©volutifs des bactÃ©riÃ©mies de l'enfant au Centre hospitalier universitaire pÃ©diatrique Charles de Gaulle (Burkina Faso) | 2001—2008 | Fever Series | Culture | Bacteria | 9 | Low |
| 603 | OULAI–1997 | La fiÃ¨vre du nouveau-nÃ© en milieu tropical. Aspects Ã©tiologiques | 1997—1997 | Fever Series | Culture | Bacteria | 9 | Low |
| 604 | Parker–2007 | Concurrent infections in acute febrile illness patients in Egypt | 2005—2006 | Fever Series | Serological | Bacteria | 4 | Moderate |
| 605 | Passeron–2010 | Suppurations intracrÃ¢niennes Ã  porte d'entrÃ©e otorhinolaryngologique chez l'enfant au SÃ©nÃ©gal | 1996—2004 | Fever Series | Culture | Bacteria | 1 | Low |
| 606 | RAKOTOARIVELO–2011 | Les formes graves de la fiÃ¨vre de la vallÃ©e de Rift pendant l'Ã©pidÃ©mie Ã  Madagascar | 2008—2008 | Case Series | PCR | Viruses | 1 | High |
| 607 | Roca–2006 | Invasive pneumococcal disease in children<5 years of age in rural Mozambique | 2001—2003 | Fever Series | Culture | Bacteria | 6 | Low |
| 608 | Rodier–1996 | Epidemic dengue 2 in the city of Djibouti 1991-1992 | 1991—1992 | Seroprevalance study | Serological | Viruses | 9 | Moderate |
| 609 | Rollin–1987 | Viral haemorrhagic fever seroepidemiology in Mauritania | 1976—1976 | Seroprevalance study | Serological | Viruses | 3 | Moderate |
| 610 | Rotimi–1987 | Occurrence and antibiotic susceptibility profiles of methicillin-resistant Staphylococcus aureus in Lagos University Teaching Hospital | 1985—1985 | Fever Series | Culture | Bacteria | 1 | Low |
| 611 | Saluzzo–1984 | Isolation of Crimean-Congo haemorrhagic fever and Rift Valley fever viruses in Upper Volta | 1984—1984 | Fever Series | Serological | Viruses | 2 | Moderate |
| 612 | Sanders–1996 | Sentinel surveillance for yellow fever in Kenya, 1993 to 1995 | 1993—1995 | Seroprevalance study | Serological | Viruses | 1 | Moderate |
| 613 | SANOU–2006 | MÃ©ningites Ã  mÃ©ningocoque du sÃ©rogroupe W135 : Ã‰tude de 148 cas observÃ©s en 2002 et en 2003 au CHU-YO de Ouagadougou, Burkina Faso | 2002—2003 | Case Series | Culture | Bacteria | 3 | High |
| 614 | Schelling–2004 | SÃ©roprÃ©valences des maladies zoonotiques chez les pasteurs nomades et leurs animaux dans le Chari-Baguirmi du Tchad : SpÃ©cial ""Nomades au Tchad"" | 1999—2000 | Seroprevalance study | Serological | Bacteria | 2 | Moderate |
| 615 | Sergon–2008 | Seroprevalence of Chikungunya virus (CHIKV) infection on Lamu Island, Kenya, October 2004 | 2004—2004 | Seroprevalance study | Serological | Viruses | 1 | Moderate |
| 616 | Sergon–2007 | Seroprevalence of Chikungunya virus infection on Grande Comore Island, union of the Comoros, 2005 | 2005—2005 | Seroprevalance study | Serological | Viruses | 1 | Moderate |
| 617 | Sharp–1987 | Anthropophilic mosquitoes at Richards Bay, Natal, and arbovirus antibodies in human residents | 1982—1982 | Seroprevalance study | Serological | Viruses | 10 | Moderate |
| 618 | Sibanda–1991 | Infection after Harare valve V-P shunt operations: a review of 92 cases | 1986—1988 | Case Series | Culture | Bacteria/Fungi | 6 | High |
| 619 | Sow–2005 | Burden of invasive disease caused by Haemophilus influenzae type b in Bamako, Mali: impetus for routine infant immunization with conjugate vaccine | 2002—2004 | Fever Series | Culture | Bacteria | 1 | Low |
| 620 | Talbert–1998 | Spraying tick-infested houses with lambda-cyhalothrin reduces the incidence of tick-borne relapsing fever in children under five years old | 1994—1995 | Fever Series | Culture | Bacteria | 1 | Low |
| 621 | THONNON–1999 | EpidÃ©mies Ã  virus Chikungunya en 1996 et 1997 au SÃ©nÃ©gal | 1996—1996 | Seroprevalance study | Serological | Viruses | 2 | Moderate |
| 622 | Thonnon–1999 | Rift valley fever surveillance in the lower Senegal river basin: update 10 years after the epidemic | 1995—1996 | Seroprevalance study | Serological | Viruses | 1 | Moderate |
| 623 | Tomori–2008 | Rift Valley fever virus infection in man in Nigeria | 1969—1978 | Seroprevalance study | Culture/Serological | Viruses | 1 | Moderate |
| 624 | Tomori–1988 | Viral hemorrhagic fever antibodies in Nigerian populations | 1986—1988 | Seroprevalance study | Serological | Viruses | 6 | Moderate |
| 625 | Tomori–1981 | Arbovirus infections among laboratory personnel in Ibadan, Nigeria | 1963—1977 | Fever Series | Serological | Viruses | 6 | Moderate |
| 626 | Towner–2006 | Marburgvirus genomics and association with a large hemorrhagic fever outbreak in Angola | 2005—2005 | Fever Series | Serological | Viruses | 1 | Moderate |
| 627 | Trape–1991 | Tick-borne borreliosis in west Africa | 1989—1990 | Case Series/Fever Series | Culture | Bacteria | 1 | High |
| 628 | Tsai–1987 | Investigation of a possible yellow fever epidemic and serosurvey for flavivirus infections in northern Cameroon, 1984 | 1984—1984 | Fever Series | Serological | Viruses | 5 | Moderate |
| 629 | Uys–2000 | Viral isolates during febrile neutropaenia in children with cancer | 1996—1997 | Fever Series | Culture | Bacteria/Viruses | 6 | Low |
| 630 | van de Wetering–2001 | Bacteraemia in a paediatric oncology unit in South Africa | 1991—1995 | Fever Series | Culture | Bacteria/Fungi | 21 | Low |
| 631 | YARO–2010 | Situation Ã©pidÃ©miologique de la fiÃ¨vre jaune au Burkina Faso de 2003 Ã  2008 | 2005—2005 | Seroprevalance study | Serological | Viruses | 1 | Moderate |
| 632 | Zakaria–1988 | The etiology of acute hepatitis in hospitalized children in Cairo Egypt | 1983—1983 | Fever Series | Serological | Viruses | 3 | Moderate |
| 633 | Swanepoel–1985 | Investigations following initial recognition of Crimean-Congo haemorrhagic fever in South Africa and the diagnosis of 2 further cases | 1980—1984 | Fever Series/Seroprevalence | Serological | Viruses | 2 | Moderate |
| 634 | Kalai Blagui–2007 | Nosocomial outbreak of OXA-18-producing Pseudomonas aeruginosa in Tunisia | 1998—2000 | Case Series | Culture | Bacteria | 1 | High |
| 635 | Kalongi–1999 | Isolated case of Ebola hemorrhagic fever with mucormycosis complications, Kinshasa, Democratic Republic of the Congo | 1995—1995 | Case Series | Serological | Viruses | 1 | High |
| 636 | Kariuki–2006 | Decreasing prevalence of antimicrobial resistance in non-typhoidal Salmonella isolated from children with bacteraemia in a rural district hospital, Kenya | 1994—1997 | Case Series | Culture | Bacteria | 2 | High |
| 637 | Peltola–2001 | Burden of meningitis and other severe bacterial infections of children in africa: implications for prevention | 1979—1991 | Case Series | Culture | Bacteria | 3 | High |
| 638 | Pieboji–2004 | Antimicrobial resistance of Gram-negative bacilli isolates from inpatients and outpatients at Yaounde Central Hospital, Cameroon | 1995—1998 | Case Series | Culture | Bacteria | 5 | High |
| 639 | Poirel–2001 | GES-2, a class A beta-lactamase from Pseudomonas aeruginosa with increased hydrolysis of imipenem | 2000—2000 | Case Series | Culture | Bacteria | 1 | High |
| 640 | Rwaguma–1997 | Emergence of epidemic O'nyong-nyong fever in southwestern Uganda, after an absence of 35 years | 1996—1996 | Case Series | Culture | Viruses | 1 | High |
| 641 | SAGHROUNI–2012 | Les fongÃ©mies mixtes. Ã€ propos de quatre cas tunisiens | 2010—2011 | Case Series | Culture | Fungi | 6 | High |
| 642 | Vaagland–2004 | Nosocomial outbreak of neonatal Salmonella enterica serotype Enteritidis meningitis in a rural hospital in northern Tanzania | 2000—2000 | Fever Series | Culture | Bacteria | 2 | Low |
| 643 | VICENS–1993 | L'Ã©pidÃ©mie de fiÃ¨vre jaune de l'ExtrÃªme Nord du Cameroun en 1990: premier isolement du virus amaril au Cameroun | 1990—1990 | Case Series | Culture/Serological | Viruses | 1 | High |
| 644 | Walker–1982 | Pathologic and virologic study of fatal Lassa fever in man | 1976—1980 | Fever Series | Culture | Viruses | 1 | Low |
| 645 | Wall–1986 | Meningococcal bacteraemia in febrile contacts of patients with meningococcal disease | 1986—1986 | Fever Series | Culture | Bacteria | 2 | Low |
| 646 | Williams–1986 | Application of enzyme immunoassays for the confirmation of clinically suspect plague in Namibia, 1982 | 1982—1982 | Fever Series | Culture | Bacteria | 1 | Low |
| 647 | Wilson–1994 | Rift Valley fever in rural northern Senegal: human risk factors and potential vectors | 1989—1989 | Seroprevalance study | Serological | Viruses | 1 | Moderate |
| 648 | Woods–2002 | An outbreak of Rift Valley fever in Northeastern Kenya, 1997-98 | 1997—1998 | Seroprevalance study | Serological | Viruses | 1 | Moderate |
| 649 | Zaki–2007 | Extended spectrum beta-lactamases among gram-negative bacteria from an Egyptian pediatric hospital: a two-year experience | 2005—2006 | Fever Series | Culture | Bacteria | 11 | Low |
| 650 | Zougaghi–2009 | Leishmaniose viscÃ©rale infantile : profil Ã©pidÃ©miologique, clinique et biologique. A propos de 93 cas | 1997—2001 | Fever Series | Serological | Parasites | 1 | Moderate |
| 651 | Centers for Disease Control and Prevention–1993 | Rift Valley fever--Egypt, 1993 | 1993—1993 | Seroprevalance study | Serological | Viruses | 1 | Moderate |
| 652 | Centers for Disease Control and Prevention–1995 | Outbreak of Ebola Viral Hemorrhagic Fever -- Zaire, 1995 | 1995—1995 | Fever Series | Serological | Viruses | 1 | Moderate |
| 653 | Centers for Disease Control and Prevention–2007 | Rift Valley fever outbreak--Kenya, November 2006-January 2007 | 2006—2007 | Seroprevalance study | Serological | Viruses | 1 | Moderate |
| 654 | Cheesbrough–1997 | Clinical definition for invasive Salmonella infection in African children | 1990—1992 | Fever Series | Culture | Bacteria | 9 | Low |
| 655 | Farag–2005 | Epidemiological, clinical and prognostic profile of acute bacterial meningitis among children in Alexandria, Egypt | 2002—2003 | Fever Series | Culture | Bacteria | 3 | Low |
| 656 | LE GUENNO–1997 | Le virus Ebola : donnÃ©es Ã©cologiques | 1996—1996 | Seroprevalance study | PCR | Viruses | 1 | Moderate |
| 657 | Mulholland–1999 | Etiology of serious infections in young Gambian infants | 1990—1991 | Fever Series | Culture | Bacteria | 11 | Low |
| 658 | Eltoum–1992 | Congenital kala-azar and leishmaniasis in the placenta | 1989—1989 | Case Series | Serological | Parasites | 1 | High |
| 659 | ENNIBI–2009 | Nodules hÃ©patiques fÃ©briles chez un marocain | 2008—2008 | Case Series | Culture | Bacteria | 1 | High |
| 660 | ESSABBAH AGUIR–2013 | La leishmaniose viscÃ©rale de l'adulte immunocompÃ©tent. Ã€ propos de six cas | 1998—2009 | Case Series | Not described | Parasites | 1 | High |
| 661 | FAUCHER–2009 | SurditÃ© dÃ©finitive au dÃ©cours d'une fiÃ¨vre typhoÃ¯de : une observation Ã  Dakar (SÃ©nÃ©gal) | 2007—2007 | Case Series | Culture | Bacteria | 1 | High |
| 662 | Feldman–1990 | Klebsiella pneumoniae bacteraemia at an urban general hospital | 1987—1988 | Case Series | Culture | Bacteria | 1 | High |
| 663 | Nesbitt–1989 | Salmonella septicaemias in Kenyan children | 1986—1986 | Case Series | Culture | Bacteria | 3 | High |
| 664 | Olowu–2002 | Klebsiella-induced purpura fulminans in a Nigerian child: case report and a review of literature | 2000—2000 | Case Series | Culture | Bacteria | 1 | High |
| 665 | Wasfy–2002 | Trends of multiple-drug resistance among Salmonella serotype Typhi isolates during a 14-year period in Egypt | 1987—2000 | Case Series | Culture | Bacteria | 1 | High |
| 666 | Perovic–1999 | Invasive group B streptococcal disease in nonpregnant adults | 1995—1997 | Case Series | Culture | Bacteria | 1 | High |
| 667 | Muhe–1999 | Pneumococcal and Haemophilus influenzae meningitis in a children's hospital in Ethiopia: serotypes and susceptibility patterns | 1993—1995 | Case Series | Culture | Bacteria | 2 | High |
| 668 | Siam–1980 | Ocular disease resulting from infection with Rift Valley fever virus | 1977—1977 | Case Series | Serological | Viruses | 1 | High |
| 669 | JOUHADI–2003 | FiÃ¨vre typhoÃ¯de chez le nourrisson : Ã  propos de trois observations | 2001—2002 | Case Series | Culture | Bacteria | 1 | High |
| 670 | Migliani–2002 | Les meningites bacteriennes non tuberculeuses de l'enfant a Antananarivo, Madagascar. | 1998—2000 | Case Series | Culture | Bacteria | 4 | High |
| 671 | Lepage–1984 | Severe multiresistant Salmonella typhimurium systemic infections in Central Africa--clinical features and treatment in a paediatric department | 1982—1983 | Case Series | Culture | Bacteria | 1 | High |
| 672 | IBRAHIM MONTASSER–2011 | FiÃ¨vre boutonneuse mÃ©diterranÃ©enne compliquÃ©e d'insuffisance rÃ©nale aiguÃ« | 2008—2009 | Case Series | Serological | Bacteria | 1 | High |
| 673 | SEYDI–2002 | Aspects cliniques, bacteriologiques et therapeutiques des meningites cerebro-spinales a Dakar en 1999 | 1999—1999 | Case Series | Culture | Bacteria | 3 | High |
| 674 | Sharaf Eldin–2008 | Symptomatic acute hepatitis C in Egypt: diagnosis, spontaneous viral clearance, and delayed treatment with 12 weeks of pegylated interferon alfa-2a | 2002—2006 | Fever Series | Serological | Viruses | 3 | Moderate |
| 675 | Maher–1994 | Bacteraemia in Blantyre | 1991—1992 | Fever Series | Culture | Bacteria | 9 | Low |
| 676 | Coovadia–1983 | Salmonella typhi meningitis. A case report | Not stated | Case Series | Culture | Bacteria | 1 | High |
| 678 | Bogaerts–1993 | Antimicrobial susceptibility and serotype distribution of Streptococcus pneumoniae from Rwanda, 1984-1990 | 1984—1990 | Case Series | Culture | Bacteria | 1 | High |
| 679 | MOSSORO-KPINDE–2012 | SensibilitÃ© aux antibiotiques des sÃ©rotypes Typhi, Paratyphi A, B et C de Salmonella Ã  Bangui | 2010—2010 | Case Series/Fever Series | Culture | Bacteria | 4 | High |
| 680 | MOUANODJI MBAISSOUROUM–1993 | Un diagnostic inhabituel d'une fiÃ¨vre prolongÃ©e chez un cardiaque: le kala-azar | 1992—1992 | Case Series | Culture | Parasites | 1 | High |
| 681 | NEBIE–2008 | ENDOCARDITE INFECTIEUSE : Etude de 32 cas au Centre Hospitalier Universitaire de Ouagadougou -Burkina Faso | 1997—2002 | Fever Series | Culture | Bacteria | 2 | Low |
| 682 | Peddie–1988 | Methicillin-resistant Staphylococcus aureus at Tygerberg Hospital | 1985—1985 | Case Series | Culture | Bacteria | 2 | High |
| 683 | Kariuki–2005 | Increasing prevalence of multidrug-resistant non-typhoidal salmonellae, Kenya, 1994-2003 | 1997—2000 | Case Series | Culture | Bacteria | 8 | High |
| 684 | Keddy–1996 | Incidence of high-level gentamicin resistance in enterococci at Johannesburg Hospital | 1994—1994 | Case Series | Culture | Bacteria | 1 | High |
| 685 | HATIM–2014 | Endocardite infectieuse compliquÃ©e d'un anÃ©vrysme de l'artÃ¨re mÃ©sentÃ©rique supÃ©rieure | 2010—2010 | Case Series | Culture | Bacteria | 1 | High |
| 686 | SKHIRI–2004 | Insuffisance rÃ©nale aiguÃ« au cours de la fiÃ¨vre boutonneuse mÃ©diterranÃ©enne : Description de trois observations | 2002—2003 | Case Series | Serological | Bacteria | 1 | High |
| 687 | Scott–1998 | Serotype distribution and prevalence of resistance to benzylpenicillin in three representative populations of Streptococcus pneumoniae isolates from the coast of Kenya | 1992—1996 | Case Series | Culture | Bacteria | 1 | High |
| 688 | Flegg–1986 | Unusual complications of typhoid | 1985—1985 | Case Series | Culture | Bacteria | 2 | High |
| 689 | Forgor–2005 | Emergence of W135 meningococcal meningitis in Ghana | 2003—2004 | Case Series | Culture | Bacteria | 1 | High |
| 690 | Friedland–1992 | Increased resistance to amikacin in a neonatal unit following intensive amikacin usage | 1990—1991 | Case Series | Culture | Bacteria | 3 | High |
| 691 | Gardee–1993 | The incidence of inducible macrolide-lincosamide-streptogramin B resistance in methicillin-resistant staphylococci in clinical isolates from the Eastern Cape area of South Africa | 1992—1992 | Case Series | Culture | Bacteria | 1 | High |
| 692 | Gendrel–1986 | Klebsiella pneumoniae septicemia and Schistosoma infestation | 1986—1986 | Case Series | Culture | Bacteria | 1 | High |
| 693 | Georges-Courbot–1997 | Isolation and partial molecular characterisation of a strain of Ebola virus during a recent epidemic of viral haemorrhagic fever in Gabon | 1996—1996 | Case Series | Serological | Viruses | 1 | High |
| 694 | Georges-Courbot–1997 | Isolation and phylogenetic characterization of Ebola viruses causing different outbreaks in Gabon | 1994—1996 | Case Series | Serological | Viruses | 1 | High |
| 695 | GHARBI–2008 | Les neutropÃ©nies fÃ©briles chimio-induites : Ã  propos de 200 Ã©pisodes Profil clinique, microbiologique et thÃ©rapeutique | Not stated | Fever Series | Culture | Bacteria | 3 | Low |
| 696 | Gordon–2001 | Three cases of bacteremia caused by Vibrio cholerae O1 in Blantyre, Malawi | 1998—1999 | Case Series | Culture | Bacteria | 1 | High |
| 697 | Hadfield–1985 | An outbreak of antibiotic-resistant Salmonella enteritidis in Liberia, West Africa | 1980—1982 | Case Series | Culture | Bacteria | 1 | High |
| 698 | Huebner–2000 | Trends in antimicrobial resistance and serotype distribution of blood and cerebrospinal fluid isolates of Streptococcus pneumoniae in South Africa, 1991-1998 | 1995—1998 | Case Series | Culture | Bacteria | 1 | High |
| 699 | Hussey–1994 | Serotypes and antimicrobial susceptibility of Haemophilus influenzae | 1991—1992 | Case Series | Culture | Bacteria | 1 | High |
| 700 | Johnson–1992 | Chloramphenicol-resistant Haemophilus influenzae meningitis in young urban Nigerian children | 1990—1991 | Case Series | Culture | Bacteria | 1 | High |
| 701 | Kelly–1991 | Tick-bite fever in Zimbabwe. Survey of antibodies to Rickettsia conorii in man and dogs, and of rickettsia-like organisms in dog ticks | 1989—1990 | Case Series | Serological | Bacteria | 1 | High |
| 702 | Ki-Zerbo–2000 | SÃ©roprÃ©valence des rickettsioses et de la fiÃ¨vre Q chez les patients fÃ©briles Ã  l'hÃ´pital de Bobo-Dioulasso (Burkina Faso) | 1995—1995 | Seroprevalance study | Serological | Bacteria | 3 | Moderate |
| 703 | KOKO–1997 | Trypanosomose humaine africaine chez l'enfant. ExpÃ©rience d'un service de pÃ©diatrie Ã  Libreville, Gabon | 1989—1994 | Case Series | Serological | Parasites | 1 | High |
| 704 | Koko–2002 | Sensibilite aux antibiotiques des germes responsables des meningites purulentes de l'enfant a Libreville (Gabon). | 1989—1994 | Case Series | Culture | Bacteria | 3 | High |
| 705 | Leroy–2002 | Re-emergence of ebola haemorrhagic fever in Gabon | 2001—2001 | Case Series | Serological | Viruses | 1 | High |
| 706 | Lutwama–2002 | Isolations of Bwamba virus from south central Uganda and north eastern Tanzania | 1994—1997 | Case Series | Serological | Viruses | 1 | High |
| 707 | Rowe–1999 | Clinical, virologic, and immunologic follow-up of convalescent Ebola hemorrhagic fever patients and their household contacts, Kikwit, Democratic Republic of the Congo. Commission de Lutte contre les Epidemies a Kikwit | 1995—1995 | Case Series | PCR | Viruses | 1 | High |
| 708 | Vasilakis–2008 | Sylvatic dengue virus type 2 activity in humans, Nigeria, 1966 | 1964—1968 | Case Series | Serological | Viruses | 1 | High |
| 709 | Pillay–1999 | An outbreak of neonatal infection with Acinetobacter linked to contaminated suction catheters | 1997—1997 | Case Series | Culture | Bacteria | 1 | High |
| 710 | Ogunlesi–2005 | Pyogenic meningitis in Ilesa, Nigeria | 1998—2003 | Case Series | Culture | Bacteria | 5 | High |
| 711 | RAOBIJAONA–2000 | FiÃ¨vre typhoide chez l'enfant Ã  Antananarivo | 1994—1994 | Case Series | Serological | Bacteria | 1 | High |
| 712 | NDIAYE–2005 | Un cas de mÃ©ningoencÃ©phalite Ã  Acahthamoeba sp. Ã  Dakar | 1999—1999 | Case Series | Culture | Bacteria | 1 | High |
| 713 | Olle-Goig–2006 | Multiorgan involvement due to Salmonella typhi: case report | 2006—2006 | Case Series | Culture | Bacteria | 1 | High |
| 714 | Nkemngu–2005 | Treatment failure in a typhoid patient infected with nalidixic acid resistant S. enterica serovar Typhi with reduced susceptibility to ciprofloxacin: a case report from Cameroon | 2004—2004 | Case Series | Serological | Bacteria | 1 | High |
| 715 | OMEZZINE LETAIEF–1997 | Aspects Ã©pidÃ©miologiques et Ã©volutifs des bactÃ©riÃ©mies dans un service de mÃ©decine interne : Ã  propos de 148 cas | 1984—1993 | Case Series | Culture | Bacteria | 11 | High |
| 716 | Seydi–2005 | Meningites au cours des bacteriemies a Escherichia coli a la clinique des maladies infectieuses Ibrahima-Diop-Mar du Centre hospitalier national de Fann a Dakar (Senegal). | 1996—2003 | Case Series | Culture | Bacteria | 1 | High |
| 717 | Sharief–2006 | Leishmania donovani: an in vitro study of antimony-resistant amphotericin B-sensitive isolates | 2004—2005 | Case Series | Serological | Parasites | 1 | High |
| 718 | MAGNE–2013 | La leptospirose Ã  Libreville (Gabon): Ã  propos de quatre cas | 2009—2011 | Case Series | Serological | Bacteria | 1 | High |
| 719 | SIMON–2009 | Une hÃ©patopathie multinodulaire fÃ©brile en zone tropicale | 2007—2007 | Case Series | Serological | Parasites | 1 | High |
| 720 | Seydi–2004 | Place des bacteriemies a Staphylococcus aureus au CHU de Fann a Dakar. | 1996—2002 | Case Series | Culture | Bacteria | 3 | High |
| 721 | SEKKACH–2006 | DÃ©tresse respiratoire aiguÃ« rÃ©vÃ©latrice d'une leptospirose pulmonaire sÃ©vÃ¨re | 2005—2005 | Case Series | Serological | Bacteria | 1 | High |
| 722 | Ruiz–2008 | Detection of Salmonella enterica serotype typhimurium DT104 in Mozambique | 2000—2001 | Case Series | Culture | Bacteria | 1 | High |
| 723 | RUNEL-BELLIARD–2010 | FiÃ¨vre typhoÃ¯de et syndrome d'activation macrophagique chez un enfant comorien | 2009—2009 | Case Series | Culture | Bacteria | 1 | High |
| 724 | RAFIK–2011 | PolyradiculonÃ©vrite aigÃ¼e et infection Ã  Rickettsia conorii | 2010—2010 | Case Series | Serological | Bacteria | 1 | High |
| 725 | McIntosh–1980 | Rift Valley fever in humans in South Africa | 1974—1975 | Case Series | Serological | Viruses | 1 | High |
| 726 | Sow–2000 | Molecular characteristics and susceptibility to antibiotics of serogroup A Neisseria meningitidis strains isolated in Senegal in 1999 | 1998—1999 | Case Series | Culture | Bacteria | 1 | High |
| 727 | Reed–1994 | Neonatal typhoid fever | 1985—1993 | Case Series | Culture | Bacteria | 1 | High |
| 728 | Marcus–1996 | First reported African case of Anaerobiospirillum succiniciproducens septicemia | 1995—1995 | Case Series | Culture | Bacteria | 1 | High |
| 729 | Mayegga–2005 | Absence of focal neurological involvement in tick-borne relapsing fever in northern Tanzania | 2003—2003 | Case Series | Culture | Bacteria | 1 | High |
| 730 | Melkert–1988 | Relapsing fever in pregnancy: analysis of high-risk factors | 1986—1987 | Case Series | Culture | Bacteria | 1 | High |
| 731 | Mills-Robertson–2003 | Antibiotic resistance and genotyping of clinical group B Salmonella isolated in Accra, Ghana | 2001—2002 | Case Series | Culture | Bacteria | 1 | High |
| 732 | Mohamed–1989 | Pulsed Doppler and two-dimensional echocardiographic findings in coronary arteriovenous fistula complicated by bacterial endocarditis. A case report | 1988—1988 | Case Series | Culture | Viruses | 1 | High |
| 733 | Musa–2003 | Purulent pericarditis complicating septicaemia: report of two cases | 2001—2002 | Case Series | Culture | Bacteria | 2 | High |
| 734 | NADRID–1996 | Leishmaniose viscÃ©rale infantile : un diagnostic difficile en cas d'hÃ©mophagocytose associÃ©e | 1995—1995 | Case Series | Serological | Parasites | 1 | High |
| 735 | Ngumi–2006 | Nosocomial infections at Kenyatta National Hospital Intensive-Care Unit in Nairobi, Kenya | 2005—2005 | Case Series | Culture | Bacteria | 3 | High |
| 736 | NKOGHE–2005 | Plusieurs Ã©pidÃ©mies de fiÃ¨vre hÃ©morragique due au virus Ebola au Gabon, d'octobre 2001 Ã  avril 2002 | 2001—2002 | Case Series | Serological | Viruses | 1 | High |
| 737 | NOURI–1997 | Rupture spontanÃ©e de la rate et mononuclÃ©ose infectieuse | 1996—1996 | Case Series | Serological | Viruses | 1 | High |
| 738 | NOURI-MERCHAOUI–2009 | FiÃ¨vre du nouveau-nÃ©: Ã©tude de 134 cas colligÃ©s au service de nÃ©onatologie de Sousse (Tunisie) | 2003—2004 | Fever Series | Culture | Bacteria | 4 | Low |
| 739 | Okesola–1999 | Prevalence and antibiotic sensitivity pattern of methicillin-resistant Staphylococcus aureus in Ibadan, Nigeria | 1994—1995 | Case Series | Culture | Bacteria | 1 | High |
| 740 | Omar–1980 | Pasteurella multocida septicaemia complicating Felty's syndrome: A case report | 1979—1979 | Case Series | Culture | Bacteria | 1 | High |
| 741 | Oppenheim–1986 | Antibiotic-resistant pneumococcal disease in children at Baragwanath Hospital, Johannesburg | 1983—1983 | Case Series | Culture | Bacteria | 1 | High |
| 742 | Organisation Mondiale de la SantÃ©.–1999 | FiÃ¨vre hÃ©morragique virale/Marburg, RÃ©publique dÃ©mocratique du Congo. DerniÃ¨res nouvelles du comitÃ© de coordination Ã  Durba | 1999—1999 | Case Series | Serological | Viruses | 1 | High |
| 743 | Perine–1983 | Antibiotic treatment of louse-borne relapsing fever in Ethiopia: a report of 377 cases | 1971—1976 | Case Series | Culture | Bacteria | 1 | High |
| 744 | Sandler–1989 | Meningococcaemia complicated by myocarditis. A report of 2 cases | 1987—1988 | Case Series | Culture | Bacteria | 1 | High |
| 745 | Sindato–2008 | Challenges in the diagnosis and management of sleeping sickness in Tanzania: a case report | 2007—2007 | Case Series | Culture | Parasites | 1 | High |
| 746 | Skoll–1998 | Aeromonas hydrophila in burn patients | 1993—1995 | Case Series | Culture | Bacteria | 1 | High |
| 747 | Slabbert–2005 | Endotoxic shock after gamete intrafallopian transfer | 2004—2004 | Case Series | Culture | Bacteria | 1 | High |
| 748 | Spencer–1988 | Disturbances of blood coagulation associated with Salmonella typhi infections | 1985—1986 | Fever Series | Culture | Bacteria | 1 | Low |
| 749 | Taylor–2005 | Macrofilaricidal activity after doxycycline treatment of Wuchereria bancrofti: a double-blind, randomised placebo-controlled trial | 2003—2004 | Case Series | Serological | Parasites | 1 | High |
| 750 | Tien-Nguyen–1993 | Endocardite a Coxiella burnetii sur bioprothese valvulaire: revue de la litterature a propos d'un cas. | 1992—1992 | Case Series | Serological | Bacteria | 1 | High |
| 751 | Topley–1986 | Mild typhoid fever | Not stated | Fever Series | Culture | Bacteria | 1 | Low |
| 752 | TOUMI–2006 | Aspects Ã©pidÃ©miologiques, cliniques et thÃ©rapeutiques de la leishmaniose viscÃ©rale de l'adulte Ã  l'hÃ´pital de la Rabta Ã  Tunis (Tunisie) entre 1983 et 2002 | 1983—2002 | Case Series | Culture/Serological | Parasites | 1 | High |
| 753 | TOUMI–2006 | MÃ©ningite rÃ©vÃ©lant une infection Ã  Rickettsia typhi | 2005—2005 | Case Series | Serological | Bacteria | 1 | High |
| 754 | TRAORE–2015 | Aspects hÃ©matologiques des fiÃ¨vres typhoÃ¯des dans le service de mÃ©decine interne Ã  l'HÃ´pital National du point-Â«GÂ» (Ã  propos de 59 cas) | 1984—1988 | Case Series | Culture | Bacteria | 1 | High |
| 755 | Widdowson–1998 | Emergence of the M phenotype of erythromycin-resistant pneumococci in South Africa | 1987—1991 | Case Series | Culture/Serological | Bacteria | 1 | High |
| 756 | Zerouali–2002 | Serogroups, serotypes, serosubtypes and antimicrobial susceptibility of Neisseria meningitidis isolates in Casablanca, Morocco | 1992—2000 | Case Series | Culture | Bacteria | 6 | High |
| 758 | Ageep–2006 | Clinical presentations and laboratory findings in suspected cases of dengue virus | 2005—2005 | Seroprevalance study | Serological | Viruses | 1 | Moderate |
| 759 | Ahoyo–2007 | Incidence of infections dues to Escherichia coli strains producing extended spectrum betalactamase, in the Zou/Collines Hospital Centre (CHDZ/C) in Benin (English) | 2005—2005 | Case Series | Culture | Bacteria | 1 | High |
| 760 | Airede–2008 | Neonatal bacterial meningitis and dexamethasone adjunctive usage in Nigeria | 1992—1995 | Case Series | Culture | Bacteria | 12 | High |
| 761 | Ait El Kadi–2006 | Prevalence of Acinetobacter baumannii and Pseudomonas aeruginosa isolates resistant to imipenem by production of metallo-beta-lactamase (English) | 2004—2005 | Case Series | Culture | Bacteria | 2 | High |
| 762 | Akakpo-Numado–2008 | Current bacterial causes of osteomyelitis in children with sickle cell disease (English) | 1998—2005 | Fever Series | Culture | Bacteria | 1 | Low |
| 763 | Akinyemi–2000 | Prevalence of multi-drug resistant Salmonella typhi among clinically diagnosed typhoid fever patients in Lagos, Nigeria | 1997—1998 | Fever Series | Culture | Bacteria | 3 | Low |
| 764 | Benabdellah–2007 | Mediterranean-spotted fever: clinical and laboratory characteristics of 34 children in Oran (Algeria) | 2002—2005 | Case Series | Serological | Bacteria | 1 | High |
| 765 | Ramos–2008 | Results of a 10-year survey of louse-borne relapsing fever in southern Ethiopia: a decline in endemicity | 1997—1998 | Case Series | Culture | Bacteria | 1 | High |
| 766 | World Health Organization–2006 | Outbreak news. Yellow fever, Cote d'Ivoire | 2006—2006 | Case Series | Serological | Viruses | 1 | High |
| 767 | Andreoni–2002 | Primary human herpesvirus 8 infection in immunocompetent children | 1999—2000 | Fever Series | PCR/Serological | Viruses | 1 | Low |
| 768 | CAMARA–2012 | Les ostÃ©omyÃ©lites aiguÃ«s Ã  Dakar | 1995—2005 | Fever Series | Culture | Bacteria | 5 | Low |
| 769 | DIGOUTTE–1985 | DonnÃ©es rÃ©centes sur les fiÃ¨vres hÃ©morragiques en Afrique de l'Ouest | 1983—1983 | Seroprevalance study | Serological | Viruses | 2 | Moderate |
| 770 | Akinyemi–2005 | Multidrug resistance in Salmonella enterica serovar typhi isolated from patients with typhoid fever complications in Lagos, Nigeria | 2000—2001 | Fever Series | Culture | Bacteria | 5 | Low |
| 771 | Akoh–1991 | Relative sensitivity of blood and bone marrow cultures in typhoid fever | 1989—1990 | Fever Series | Culture/Serological | Bacteria | 1 | Low |
| 772 | Akpede–1993 | Pattern of infections in children under-six years old presenting with convulsions associated with fever of acute onset in a children's emergency room in Benin City, Nigeria | 1988—1989 | Fever Series | Culture | Bacteria | 3 | Low |
| 773 | HIDA–1999 | Leishmaniose viscÃ©rale et malnutrition: Ã  propos d'une observation | 1996—1996 | Case Series | Culture | Parasites | 1 | High |
| 774 | Marlet–2003 | A neglected disease of humans: a new focus of visceral leishmaniasis in Bakool, Somalia | 2000—2001 | Fever Series | Serological | Parasites | 1 | Moderate |
| 775 | el-Safi–1991 | Studies on the leishmaniases in the Sudan. 2. Clinical and parasitological studies on cutaneous leishmaniasis | 1986—1986 | Fever Series | Serological | Parasites | 1 | Moderate |
| 776 | NAFIL–2011 | Association leucÃ©mie aiguÃ« lymphoblastique et leishmaniose viscÃ©rale | 2010—2010 | Case Series | Culture | Parasites | 1 | High |
| 777 | Eholie–2008 | Trois nouveaux cas de leishmaniose viscerale autochtone en Cote d'Ivoire. | 2004—2005 | Case Series | Culture | Parasites | 1 | High |
| 778 | Zijlstra–1992 | Clinical aspects of kala-azar in children from the Sudan: a comparison with the disease in adults | 1989—1990 | Case Series | Culture | Parasites | 1 | High |
| 779 | MBASSI AWA–2011 | Leishmaniose viscÃ©rale: une observation pÃ©diatrique en dehors des foyers traditionnels au Cameroun | 2010—2010 | Case Series | Culture | Parasites | 1 | High |
| 780 | Lastovica–1986 | Plasmid profiles of clinical isolates of Campylobacter with no or weak catalase activity | 1984—1985 | Case Series | Culture | Bacteria | 1 | High |
| 781 | Antia-Obong–1991 | Bacterial agents in neonatal septicaemia in Calabar, Nigeria: review of 100 cases | 1985—1987 | Fever Series | Culture | Bacteria | 10 | Low |
| 782 | Sprenger–1983 | Neonatal septicaemia due to a nutritionally deficient streptococcus. A case report | 1987—1988 | Case Series | Serological | Bacteria | 1 | High |
| 783 | World Health Organization–1999 | Viral haemorrhagic fever/Marburg, Democratic Republic of the Congo | 1999—1999 | Case Series | Serological | Viruses | 1 | High |
| 784 | Canada Communicable Disease Report–2003 | Outbreak(s) of Ebola hemorrhagic fever, Congo and Gabon, October 2001 to July 2002 | 2001—2002 | Case Series | Serological | Viruses | 1 | High |
| 785 | Akpede–1993 | Convulsions with fever of acute onset in school-age children in Benin City, Nigeria | 1988—1989 | Fever Series | Culture | Bacteria | 3 | Low |
| 786 | Akujobi–2005 | Antimicrobial susceptibility pattern of Klebsiella species from Ebonyi State University Teaching Hospital Abakaliki, Nigeria | 2003—2004 | Case Series | Culture | Bacteria | 1 | High |
| 787 | Amod–2005 | Ventriculitis due to a hetero strain of vancomycin intermediate Staphylococcus aureus (hVISA): successful treatment with linezolid in combination with intraventricular vancomycin | 2002—2002 | Case Series | Culture | Bacteria | 1 | High |
| 788 | Anah–2008 | Neonatal septicaemia in Calabar, Nigeria | 2002—2004 | Fever Series | Culture | Bacteria | 9 | Low |
| 789 | Hendricks–1995 | Brucellosis in childhood in the Western Cape | 1992—1993 | Fever Series | Serological | Bacteria | 2 | Moderate |
| 790 | Berger–2007 | An outbreak of Halomonas phocaeensis sp. nov. bacteraemia in a neonatal intensive care unit | 1999—2000 | Case Series | Culture/PCR | Bacteria | 2 | High |
| 791 | Seebaran–1990 | Typhoid fever in the adult and paediatric Indian population of Durban | 1981—1987 | Fever Series | Serological | Bacteria/Viruses | 2 | Moderate |
| 792 | Madhi–2003 | High burden of invasive Streptococcus agalactiae disease in South African infants | 1997—1999 | Case Series | Culture | Bacteria | 1 | High |
| 794 | Joubert–1990 | Cysticercal meningitis--a pernicious form of neurocysticercosis which responds poorly to praziquantel | 1988—1988 | Case Series | Culture | Parasites | 1 | High |
| 795 | Gear–1983 | Tick-bite fever in South Africa. The occurrence of severe cases on the Witwatersrand | 1979—1981 | Case Series | Serological | Bacteria | 1 | High |
| 796 | Angyo–1998 | Changing patterns of antibiotic sensitivity and resistance during an outbreak of meningococcal infection in Jos, Nigeria | 1996—1996 | Fever Series | Culture | Bacteria | 1 | Low |
| 798 | Anstey–1997 | Seroepidemiology of Rickettsia typhi, spotted fever group rickettsiae, and Coxiella burnetti infection in pregnant women from urban Tanzania | 1993—1993 | Seroprevalance study | Serological | Bacteria | 1 | Moderate |
| 800 | Ben Nejma–2006 | Molecular characterization of methicillin-resistant Staphylococcus aureus isolated in Tunisia | 2003—2004 | Case Series | Culture | Bacteria | 1 | High |
| 801 | Berkowitz–1984 | Bacteremia in hospitalized black South African children. A one-year study emphasizing nosocomial bacteremia and bacteremia in severely malnourished children | 1982—1982 | Case Series | Culture | Bacteria | 11 | High |
| 802 | Grobler–1997 | Bacterial meningitis in children at Kalafong Hospital, 1990-1995 | 1990—1995 | Case Series | Culture | Bacteria | 3 | High |
| 803 | Moodley–1990 | Benign intracranial hypertension in typhoid fever. A case report | 1989—1989 | Case Series | Culture | Bacteria | 1 | High |
| 804 | de Moor–1985 | Brain abscess in children due to Streptococcus milleri. A report of 2 cases | 1983—1984 | Case Series | Culture | Bacteria | 2 | High |
| 805 | Boisier–2007 | Case-fatality ratio of bacterial meningitis in the African meningitis belt: we can do better | 2003—2006 | Fever Series | Culture | Bacteria | 8 | Low |
| 806 | World Health Organization–1994 | La fiÃ¨vre jaune en 1992 et 1993 | 1992—1993 | Case Series | Serological | Viruses | 1 | High |
| 807 | Adam–1992 | Biological parameters of patients infected by the Rift Valley fever virus in Rosso (Mauritania) (English) | 1987—1987 | Case Series | PCR | Viruses | 1 | High |
| 808 | AUBRY–1983 | Les borrelioses Ã  tiques au SÃ©nÃ©gal: Ã  propos de 23 observations | 1981—1982 | Case Series | Culture | Bacteria | 1 | High |
| 809 | BARDI–1993 | AnÃ©vrysme mycotique de l'artÃ¨re iliaque rÃ©vÃ©lÃ© par une mass abdominale | 1992—1992 | Case Series | Culture | Bacteria | 1 | High |
| 810 | BEN OTHMAN–1996 | Aspergillose pulmonaire du sujet neutropenique. A propos de 4 cas | 1994—1994 | Case Series | Culture | Fungi | 1 | High |
| 811 | BENHAMMOU–1991 | FiÃ©vre boutonneuse mÃ©diterranÃ©enne rÃ©vÃ©lÃ©e par une atteinte neurologique grave | 1990—1990 | Case Series | Serological | Bacteria | 1 | High |
| 812 | BOIRO–1987 | ElÃ©ments de recherches clinico-Ã©pidÃ©miologiques et de laboratoire sur les fiÃ¨vres hÃ©morragiques en GuinÃ©e | 1982—1983 | Seroprevalance study | Serological | Viruses | 1 | Moderate |
| 813 | BONKOUNGOU–2007 | LES FIEVRES TYPHO-PARATYPHIQUES DANS LE SERVICE DE PEDIATRIE DU CENTRE HOSPITALIER UNIVERSITAIRE DE BOBO-DIOULASSO : ASPECTS EPIDEMIOLOGIQUES ET CIRCONSTANCES DE DIAGNOSTIC | 2004—2004 | Case Series/Fever Series | Culture | Bacteria | 1 | High |
| 814 | BOUDERKA–2002 | Aspect Ã©pidemiologique et pronostic des bactÃ©riemies nosocomiales en rÃ©animation | 1999—2001 | Fever Series | Culture | Bacteria/Fungi | 12 | Low |
| 815 | BOUSSAYMA KHEMAKHEM HAMMAMI–2007 | L'ABCES DU PSOAS DE L'ADULTE : Etude de 38 cas | 1990—2005 | Fever Series | Culture | Bacteria | 3 | Low |
| 816 | BUISSON–1983 | ActivitÃ©s du centre national sÃ©nÃ©galais des entÃ©robactÃ©ries en 1980, 1981 et 1982 | 1980—1982 | Case Series | Culture | Bacteria | 15 | High |
| 817 | FLOCH–1990 | FiÃ¨vre rÃ©currente au Burundi: premier cas observÃ© depuis 30 ans | 1990—1990 | Case Series | Culture | Bacteria | 1 | High |
| 818 | FRADI–2006 | Endocardite infectieuse a Coxiella Burnetii : A propos d'un cas | 2005—2005 | Case Series | Serological | Bacteria | 1 | High |
| 819 | GALLAIS–1993 | La fiÃ¨vre thyphoÃ¯de en Afrique Noire | 1976—1980 | Fever Series | Culture/Serological | Bacteria | 1 | Low |
| 820 | GENDREL–1984 | FiÃ¨vres typhoÃ¯des et drÃ©panocytose: une sensibilitÃ© particuliÃ¨re des sujets AS? | 1982—1983 | Case Series | Culture | Bacteria | 4 | High |
| 821 | GEORGES–1984 | Aspects bactÃ©riologiques et cliniques des infections Ã  Salmonella typhi en RÃ©publique Centrafricaine: bilan d'une Ã©tude de 3 annÃ©es Ã  Bangui | 1980—1983 | Fever Series | Culture | Bacteria | 1 | Low |
| 822 | GONZALEZ–1985 | Approche sÃ©rologique sur l'incidence des rickettsioses en RÃ©publique centrafricaine | 1983—1984 | Seroprevalance study | Serological | Bacteria | 3 | Moderate |
| 823 | HAMZA–2006 | Association arthrite idiopathique juvÃ©nile et maladie de lyme : A propos d'une observation | 2005—2005 | Case Series | Serological | Bacteria | 1 | High |
| 824 | HELA–2005 | Oligoarthrite septique compliquant une polyarthrite rhumatoide - : A propos d'un cas | 2000—2000 | Case Series | Culture | Bacteria | 1 | High |
| 825 | HIDA–1998 | HydrocholÃ©cyste rÃ©vÃ©lateur d'une hÃ©patite virale A chez l'enfant : A propos d'un cas | 1996—1996 | Case Series | Serological | Viruses | 1 | High |
| 826 | Hildenwall–2015 | Causes of non-malarial febrile illness in outpatients in Tanzania. | 2011—2012 | Fever Series | Culture | Bacteria | 4 | Low |
| 827 | JEMNI–1985 | La fiÃ¨vre boutonneuse mÃ©diterranÃ©enne: Ã  propos des cas observÃ©s Ã  Sousse | 1983—1985 | Case Series | Serological | Bacteria | 1 | High |
| 828 | JEMNI–1992 | FiÃ¨vre prolongÃ©e, hÃ©patite granulomateuse et infection Ã  Coxiella burnetti | 1990—1990 | Case Series | Serological | Bacteria | 1 | High |
| 829 | KANE–2000 | Les endocardites infectieuses Ã  Dakar: Ã  propos de 35 cas | 1998—1998 | Fever Series | Culture | Bacteria | 7 | Low |
| 830 | KENANE–2010 | DiarrhÃ©e fÃ©brile Â« jus de melon Â»: penser Ã  la mÃ©ningite Ã  pneumocoques ! | 2008—2009 | Case Series | Culture | Bacteria | 1 | High |
| 831 | KILANI–2005 | L'abcÃ¨s cÃ©rÃ©bral Ã  pyogÃ¨nes en milieu mÃ©dical un problÃ¨me toujours d'actualitÃ© | 1989—2000 | Fever Series | Culture | Bacteria | 5 | Low |
| 833 | LAMIA–2005 | Profil bacteriologique des bacteriemies chez les brules | 2001—2002 | Case Series | Culture | Bacteria | 10 | High |
| 834 | LENIAUD–1984 | Les fiÃ¨vres typhoÃ¯des en service de rÃ©animation | 1979—1982 | Fever Series | Culture/Serological | Bacteria | 6 | Low |
| 835 | MAHMAL–2004 | Les germes producteurs de beta lactamases Ã  spectre etendu: Epidemie dans un service d'hemato-oncologie | 2000—2000 | Case Series | Culture | Bacteria | 1 | High |
| 836 | MAZIGH M'RAD–2000 | Granulomatose septique chronique chez une fillette rÃ©vÃ©lÃ©e par une aspergilose pulmonaire invasive et une paraplÃ©gie flasque | 1998—1998 | Case Series | Serological | Fungi | 1 | High |
| 837 | MBULA–1995 | Le profil biologique de la fiÃ¨vre typhoide Ã  Kinshasa | 1979—1980 | Fever Series | Culture/Serological | Bacteria | 1 | Low |
| 838 | MEUNIER–1988 | La fiÃ¨vre de la vallÃ©e du Rift et les phlÃ©boviroses en RÃ©publique centrafricaine | 1983—1985 | Seroprevalance study | Serological | Viruses | 1 | Moderate |
| 839 | MEUNIER–1987 | DonnÃ©es sÃ©rologiques actuelles sur les fiÃ¨vres hÃ©morragiques virales en RÃ©publique Centrafricaine | 1985—1985 | Seroprevalance study | Serological | Viruses | 5 | Moderate |
| 840 | MILLOGO–2002 | La trypanosomose humaine africaine : Ã‰tude retrospective de 7 cas dans le service de medecine interne de Bobo-Dioulasso (1995-2000) | 1995—2000 | Case Series | Culture | Parasites | 1 | High |
| 841 | NKURIKIYINFURA–1985 | Evaluation des hÃ©mocultures aux Cliniques Universitaires de Kinshasa | 1979—1979 | Fever Series | Culture | Bacteria | 17 | Low |
| 842 | Gear–1990 | Severe tick-bite fever in young children. A report of 3 cases | 1987—1988 | Case Series | Serological | Bacteria | 1 | High |
| 843 | Gordon–1986 | Occult life-threatening streptococcal septicaemia in the elderly. A report of 2 cases | 1984—1984 | Case Series | Culture | Bacteria | 1 | High |
| 844 | Maritz–1981 | Pasteurella ureae septicaemia. A case report | 1980—1980 | Case Series | Culture | Bacteria | 1 | High |
| 845 | Moodley–2005 | Intravenous glucose preparation as the source of an outbreak of extended-spectrum beta-lactamase-producing Klebsiella pneumoniae infections in the neonatal unit of a regional hospital in KwaZulu-Natal | 2005—2005 | Case Series | Culture | Bacteria | 2 | High |
| 847 | Aamir–2015 | Prevalence of multidrug resistant bacteria causing late-onset neonatal sepsis | 2011—2012 | Case Series | Culture | Bacteria/Fungi | 9 | High |
| 848 | Abdel-Wahab–2013 | Nosocomial infection surveillance in an Egyptian neonatal intensive care unit | 2009—2010 | Case Series | Culture | Bacteria/Fungi | 5 | High |
| 849 | Acquah–2013 | Susceptibility of bacterial etiological agents to commonly-used antimicrobial agents in children with sepsis at the Tamale Teaching Hospital | 2011—2012 | Fever Series | Culture | Bacteria | 10 | Low |
| 850 | Ajayi–2015 | The diagnosis of Salmonella typhi co-infection from blood samples of clinically suspected typhoid fever patients at some hospitals in Ondo State, Nigeria | 2013—2013 | Fever Series | Culture | Bacteria/Fungi | 9 | Low |
| 851 | Egbe–2014 | Aetiologic Agents of Fevers of Unknown Origin among Patients in Benin City, Nigeria | 2013—2013 | Fever Series | Culture | Bacteria | 12 | Low |
| 852 | OBENGUI–2004 | Considerations actuelles de la fiÃ¨vre typhoide Ã  Brazzaville | 1998—2002 | Fever Series | Culture | Bacteria | 1 | Low |
| 853 | OKOME-NKOUMOU–1999 | Un cas de fiÃ¨vre hÃ©morragique Ã  virus Ebola Ã  Libreville (Gabon) responsable d'un dÃ©cÃ¨s aprÃ¨s Ã©vacuation en Afrique du Sud | 1996—1996 | Case Series | Serological | Viruses | 1 | High |
| 854 | OUATTARA–1986 | Etiologie virale des hÃ©patites ictÃ©rigÃ¨nes en CÃ´te-d'Ivoire | 1984—1985 | Fever Series | Serological | Viruses | 4 | Moderate |
| 855 | OUSSEINI–1987 | La bilharziose, cause d'Ã©chec du traitement des salmonelloses | 1986—1986 | Case Series | Culture | Bacteria | 1 | High |
| 856 | PAIX–1988 | Etude sÃ©rologique de virus responsables de fiÃ¨vres hÃ©morragiques dans une population urbaine du Cameroun | 1985—1985 | Seroprevalance study | Serological | Viruses | 3 | Moderate |
| 857 | PERRONE–1987 | SurditÃ© isolÃ©e rÃ©gressive au cours d'une fiÃ¨vre boutonneuse mÃ©diterranÃ©enne | 1986—1986 | Case Series | Serological | Bacteria | 1 | High |
| 858 | RODHAIN–1987 | Arboviroses humaines au Burundi: rÃ©sultats d'une enquÃªte sÃ©roÃ©pidÃ©miologique, 1980-1982 | 1980—1982 | Seroprevalance study | Serological | Viruses | 3 | Moderate |
| 859 | ROUX–1984 | L'Ã©pidÃ©mie de fiÃ¨vre jaune du Sud-Est de la Haute-Volta (octobre-dÃ©cembre 1983). Etude Ã©pidÃ©miologique. RÃ©sultats prÃ©liminaires | 1983—1983 | Case Series | Not described | Viruses | 1 | High |
| 860 | World Health Organization–1988 | FiÃ¨vre de la vallÃ©e du Rift: Mauritanie | 1987—1987 | Case Series | PCR | Viruses | 1 | High |
| 861 | World Health Organization–1994 | FiÃ¨vre de la VallÃ©e du Rift. Egypte | 1993—1993 | Case Series/Fever Series | Serological | Viruses | 1 | High |
| 862 | SALAUN-SARAUX–1995 | Les mÃ©ningites septiques de l'enfant au Rwanda de 1983 Ã  1990: Ã©tude rÃ©trospective au centre hospitalier de Kigali | 1983—1990 | Fever Series | Culture | Bacteria | 4 | Low |
| 863 | SALUZZO–1983 | Une poussÃ©e Ã©pidÃ©mique due au virus chikungunya dans l'ouest du SÃ©nÃ©gal, en 1982 | 1982—1982 | Fever Series | Serological | Viruses | 1 | Moderate |
| 864 | SANOU–1988 | La fiÃ¨vre thyphoÃ¯de chez l'adulte Ã  propos de 30 cas dans le service de mÃ©decine A | 1982—1985 | Fever Series | Culture/Serological | Bacteria | 1 | Low |
| 865 | SELMI–1997 | MononuclÃ©ose infectieuse compliquÃ©e d'une dÃ©tresse respiratoire, de troubles hÃ©matologiques et d'une septicÃ©mie chez un nourrisson | 1994—1994 | Case Series | Not described | Bacteria/Viruses | 2 | High |
| 866 | SEYDI–2010 | FiÃ¨vre jaune au SÃ©nÃ©gal en 2005 | 2005—2005 | Case Series | Serological | Viruses | 1 | High |
| 867 | SIDI ALY–2001 | Endocardite Ã  Coxiella Burnetti sur canal artÃ©riel persistant | 1997—1997 | Case Series | Serological | Bacteria | 1 | High |
| 868 | TALANI–1999 | PrÃ©valence des anticorps spÃ©cifiques du monkeypox au Congo - Brazzaville | 1981—1981 | Fever Series | Serological | Viruses | 1 | Moderate |
| 869 | THABET–1999 | Syndrome d'activation inapropriÃ©e des macrophages associÃ© Ã  une Leishmaniose viscÃ©rale infantile | 1997—1997 | Case Series | Serological | Parasites | 1 | High |
| 870 | TIENDREBEOGO–1985 | Facteurs Ã©pidÃ©miologiques et cliniques de pronostic des mÃ©ningites purulentes Ã  mÃ©ningocoques Ã  Ouagadougou | 1982—1983 | Fever Series | Culture | Bacteria | 1 | Low |
| 871 | TOGOLA–2010 | Perforation typhique de la vÃ©sicule biliaire | 2009—2009 | Case Series | Serological | Bacteria | 1 | High |
| 872 | TRABELSI–1986 | La fiÃ¨vre typhoÃ¯de chez l'enfant en zone rurale tunisienne. Etude Ã©pidÃ©miologique et clinique | 1979—1983 | Fever Series | Serological | Bacteria | 1 | Moderate |
| 873 | WERNER–1985 | PrÃ©valence des anticorps contre la fiÃ¨vre jaune dans le nord du ZaÃ¯re | 1983—1984 | Seroprevalance study | Serological | Viruses | 1 | Moderate |
| 874 | World Health Organization–1995 | FiÃ¨vre hÃ©morragique Ã  virus Ebola. ZaÃ¬re | 1995—1995 | Case Series | PCR | Viruses | 1 | High |
| 875 | World Health Organization–1995 | FiÃ¨vre jaune. EnquÃªte sur une Ã©pidÃ©mie dans l'Ã©tat d'Imo. NigÃ©ria | 1994—1994 | Fever Series | Serological | Viruses | 1 | Moderate |
| 876 | World Health Organization–2007 | Outbreak news. Marburg haemorrhagic fever, Uganda | 2007—2007 | Case Series | PCR | Viruses | 1 | High |
| 877 | Centers for Disease Control and Prevention–1985 | Leads from the MMWR. Congo-Crimean hemorrhagic fever--Republic of South Africa | 1984—1984 | Case Series | PCR | Viruses | 1 | High |
| 878 | Centers for Disease Control and Prevention–2001 | Outbreak of Ebola hemorrhagic fever, Uganda, August 2000-January 2001 | 2000—2001 | Case Series | Not described | Viruses | 1 | High |
| 879 | Bass–1990 | Pseudomonas septicaemia after endoscopic retrograde cholangiopancreatography--an unresolved problem | 1984—1985 | Case Series | Culture | Bacteria | 1 | High |
| 880 | Bausch–2001 | Lassa fever in Guinea: I. Epidemiology of human disease and clinical observations | 1996—1999 | Seroprevalance study | Serological | Viruses | 1 | Moderate |
| 881 | Berkowitz–1983 | Serratia marcescens endocarditis treated with ceftazidime. A case report | 1981—1981 | Case Series | Culture | Bacteria | 1 | High |
| 882 | Bise–1997 | Epidemic typhus in a prison in Burundi | 1996—1996 | Seroprevalance study | Serological | Bacteria | 3 | Moderate |
| 883 | Bonn–2005 | Marburg fever in Angola: still a mystery disease | 1985—1985 | Fever Series | Serological | Viruses | 2 | Moderate |
| 884 | Botros–1989 | Serological evidence of dengue fever among refugees, Hargeysa, Somalia | 1985—1985 | Fever Series | Serological | Viruses | 1 | Moderate |
| 885 | Bouallegue–2004 | Outbreak of Pseudomonas putida bacteraemia in a neonatal intensive care unit | 2001—2001 | Case Series | Culture | Bacteria | 1 | High |
| 886 | Coovadia–1984 | Neisseria flavescens septicaemia with meningitis. A case report | 1983—1983 | Case Series | Culture | Bacteria | 1 | High |
| 887 | Potter–1984 | Meningitis in Cape Town children | 1981—1981 | Fever Series | Culture | Bacteria | 5 | Low |
| 888 | Pretorius–2004 | Rickettsia mongolotimonae infection in South Africa | 2002—2002 | Case Series | PCR | Bacteria | 1 | High |
| 889 | Sandler–1982 | Corynebacterium diphtheriae endocarditis in an adult with congenital cyanotic heart disease. A case report | 1981—1981 | Case Series | Culture | Bacteria | 1 | High |
| 890 | Smith–1991 | Septic shock in the Intensive Care Unit, Hillbrow Hospital, Johannesburg | 1987—1989 | Fever Series | Culture | Bacteria/Fungi | 13 | Low |
| 891 | Stones–1981 | Salmonella meningitis. A report of 4 cases | 1980—1980 | Case Series | Culture | Bacteria | 2 | High |
| 893 | DHAHRI–1995 | Alerte aux septicÃ©mies Ã  levures en rÃ©animation polyvalente | 1987—1993 | Case Series | Culture | Fungi | 1 | High |
| 894 | ABABOU–1996 | Massive bleeding complicating a brucellosis (English) | 1993—1993 | Case Series | Microscopy/Staining | Bacteria | 1 | High |
| 895 | Maharaj–1985 | Typhoid fever and haemolytic anaemia in a black patient with glucose-6-phosphate dehydrogenase deficiency. A case report | 1983—1983 | Case Series | Culture | Bacteria | 1 | High |
| 896 | Donald–1981 | Meningococcal disease at Tygerberg Hospital | 1978—1979 | Fever Series | Culture | Bacteria | 1 | Low |
| 897 | Archer–2013 | Epidemiologic investigations into outbreaks of Rift Valley fever in humans, South Africa, 2008-2011 | 2008—2011 | Seroprevalance study | Serological | Viruses | 1 | Moderate |
| 898 | Awando–2013 | Seroprevalence of anti-Dengue virus 2 serocomplex antibodies in out-patients with fever visiting selected hospitals in rural parts of Western Kenya in 2010-2011: a cross sectional study | 2010—2011 | Seroprevalance study | Serological | Viruses | 1 | Moderate |
| 899 | Faye–2014 | Reemergence of Rift Valley fever, Mauritania, 2010 | 2010—2010 | Seroprevalance study | Serological | Viruses | 1 | Moderate |
| 900 | Faye–2014 | Urban epidemic of dengue virus serotype 3 infection, Senegal, 2009 | 2009—2010 | Seroprevalance study | PCR | Viruses | 1 | Moderate |
| 901 | Feikin–2013 | Viral and bacterial causes of severe acute respiratory illness among children aged less than 5 years in a high malaria prevalence area of western Kenya, 2007-2010 | 2007—2010 | Fever Series | Culture | Bacteria | 3 | Low |
| 902 | Heinrich–2015 | High seroprevalence for spotted fever group rickettsiae, is associated with higher temperatures and rural environment in Mbeya region, Southwestern Tanzania | 2007—2008 | Seroprevalance study | Serological | Bacteria | 1 | Moderate |
| 903 | Jansen van Vuren–2015 | Serum levels of inflammatory cytokines in Rift Valley fever patients are indicative of severe disease | 2013—2013 | Case Series | PCR | Viruses | 1 | High |
| 904 | Maltha–2014 | Frequency of severe malaria and invasive bacterial infections among children admitted to a rural hospital in Burkina Faso | 2012—2013 | Fever Series | Culture | Bacteria | 9 | Low |
| 905 | Kalonji–2015 | Invasive Salmonella Infections at Multiple Surveillance Sites in the Democratic Republic of the Congo, 2011-2014 | 2011—2014 | Fever Series | Culture | Bacteria | 5 | Low |
| 906 | Mediannikov–2013 | Common epidemiology of Rickettsia felis infection and malaria, Africa | 2010—2012 | Fever Series | PCR | Bacteria | 1 | Low |
| 907 | Mediannikov–2014 | Borrelia crocidurae infection in acutely febrile patients, Senegal | 2010—2012 | Fever Series | PCR | Bacteria | 2 | Low |
| 908 | Ochieng–2015 | Seroprevalence of infections with dengue, Rift Valley fever and chikungunya viruses in Kenya, 2007 | 2007—2007 | Seroprevalance study | Serological | Viruses | 3 | Moderate |
| 909 | EKALA–1995 | SÃ©roprÃ©valence des infections Ã  rickettsies du groupe des fiÃ¨vres boutonneuse ou apparentÃ©es Ã  Bangui (RÃ©publique Centrafricaine) | 1993—1993 | Seroprevalance study | Serological | Bacteria | 1 | Moderate |
| 910 | DEROUICHE–2009 | EMPHYSEMATOUS PYELITIS: epidemiological, therapeutic and evolutive features | 1995—2009 | Case Series | Culture | Bacteria | 1 | High |
| 911 | BERTHERAT–1999 | RÃ©publique dÃ©mocratique du Congo : Entre guerre civile et virus Marburg | 1998—1999 | Fever Series | PCR | Viruses | 1 | Low |
| 912 | BOUABDELLAH–2009 | OstÃ©omyÃ©lite du pubis chez un athlÃ¨te: Ã  propos d'un cas et revue de la littÃ©rature | 2008—2008 | Case Series | Culture | Bacteria | 1 | High |
| 913 | Ben Ameur–2014 | Neonatal renal candidiasis: a case report | 1996—1996 | Case Series | Serological | Bacteria | 1 | High |
| 914 | BOUGTEBA–2011 | MÃ©ningoencÃ©phalite Ã  Rickettsia conorii chez un nourrisson | 2009—2009 | Case Series | Serological | Bacteria | 1 | High |
| 915 | Salah–1988 | A negative human serosurvey of haemorrhagic fever viruses in Djibouti | 1987—1987 | Seroprevalance study | Serological | Viruses | 2 | Moderate |
| 916 | Nabeth–2004 | Human Crimean-Congo hemorrhagic fever, Senegal | 2006—2006 | Seroprevalance study | Serological | Viruses | 1 | Moderate |
| 917 | Akoua-Koffi–2015 | Epidemiology of community-onset bloodstream infections in Bouake, central Cote d'Ivoire | 2012—2014 | Fever Series | Culture | Bacteria | 7 | Low |
| 918 | Mahende–2015 | Bloodstream bacterial infection among outpatient children with acute febrile illness in north-eastern Tanzania | 2013—2013 | Fever Series | Culture | Bacteria | 5 | Low |
| 919 | Lundgren–2015 | Bacteremia and malaria in Tanzanian children hospitalized for acute febrile illness | 2006—2009 | Fever Series | PCR | Bacteria | 2 | Low |
| 920 | Nail–2013 | Visceral leishmaniasis: Clinical and demographic features in an African population | 2006—2010 | Case Series | Microscopy/Staining | Parasites | 1 | High |
| 921 | Aamodt–2015 | Genetic relatedness and risk factor analysis of ampicillin-resistant and high-level gentamicin-resistant enterococci causing bloodstream infections in Tanzanian children | 2001—2002 | Fever Series | Culture | Bacteria/Fungi | 8 | Low |
| 922 | Abdaldafi–2014 | Serofrequency of dengue virus among febrile patients attending portsudan teaching hospital | 2014—2014 | Seroprevalance study | Serological | Viruses | 1 | Moderate |
| 923 | Abdalla–2015 | Correlation of measles and dengue infection in Kassala, Eastern Sudan | 2012—2012 | Seroprevalance study | Serological | Viruses | 2 | Moderate |
| 924 | Abdelaziz–2013 | Cephalosporin resistant Escherichia coli from cancer patients in Cairo, Egypt | 2009—2010 | Case Series | Culture | Bacteria | 1 | High |
| 925 | Abo-Salem–2015 | A huge thrombosed pulmonary artery aneurysm without pulmonary hypertension in a patient with hepatosplenic schistosomiasis | 2007—2007 | Case Series | Serological | Parasites | 1 | High |
| 926 | Abou El Azm–2013 | Can brucellosis influence the course of chronic hepatitis C in dual infection? | 2008—2011 | Fever Series | Serological | Bacteria | 1 | Moderate |
| 927 | Adiku–2015 | Aetiology of Acute Lower Respiratory Infections among Children Under Five Years in Accra, Ghana | 2001—2001 | Fever Series | Culture | Bacteria | 5 | Low |
| 928 | O'Shea–2015 | Diagnosis of febrile illnesses other than Ebola virus disease at an Ebola treatment unit in Sierra Leone | 2014—2015 | Fever Series | PCR | Viruses | 1 | Low |
| 929 | Rachas–2014 | Timeliness of yellow fever surveillance, Central African Republic | 2007—2012 | Case Series | Serological | Viruses | 1 | High |
| 930 | Weyer–2015 | Ebola virus disease: history, epidemiology and outbreaks | 1996—1996 | Case Series | Not described | Viruses | 1 | High |
| 931 | Mohammed–2015 | Seroprevalence of HBsAg among patients with febrile illnessess in Murtala Muhammad Specialist Hospital, Kano, Nigeria | 2013—2013 | Fever Series | Serological | Viruses | 1 | Moderate |
| 932 | Ziadi–2014 | [Anthrax meningoencephalitis: a case following a cutaneous lesion in Morocco] | 2013—2013 | Case Series | Culture | Bacteria | 1 | High |
| 933 | Crump–2013 | Etiology of severe non-malaria febrile illness in Northern Tanzania: a prospective cohort study | 2007—2008 | Fever Series | Serological | Bacteria/Viruses | 5 | Moderate |
| 934 | Trabelsi–2015 | Geotrichum capitatum septicemia: case report and review of the literature | 2005—2013 | Case Series | Culture | Fungi | 1 | High |
| 935 | Chebbi–2014 | [Polymorphic erythema secondary to alpha hemolytic streptococcus infection in Behcet disease] | 2013—2013 | Case Series | Culture | Bacteria | 1 | High |
| 936 | Ahmed–2013 | Microbial profile in women with puerperal sepsis in Gadarif State, Eastern Sudan | 2011—2012 | Fever Series | Culture | Bacteria | 6 | Low |
| 937 | Ahoyo–2013 | Staphylococcus sciuri outbreak at tertiary hospital in Benin | 2008—2008 | Fever Series | Culture | Bacteria/Fungi | 12 | Low |
| 938 | Aissi–2015 | Epidemiological, clinical and biological features of infantile visceral leishmaniasis at Kairouan hospital (Tunisia): about 240 cases (English) | 2004—2013 | Case Series | PCR | Parasites | 1 | High |
| 939 | Ajayi–2014 | Lassa fever - full recovery without ribavarin treatment: a case report | 2013—2013 | Case Series | PCR | Viruses | 1 | High |
| 940 | Ajayi–2013 | Containing a Lassa fever epidemic in a resource-limited setting: outbreak description and lessons learned from Abakaliki, Nigeria (January-March 2012) | 2012—2012 | Case Series | PCR | Viruses | 1 | High |
| 941 | Akanbi–2013 | Occurrence of methicillin and vancomycin resistant Staphylococcus aureus in University of Abuja Teaching Hospital, Abuja, Nigeria | 2010—2011 | Case Series | Culture | Bacteria | 2 | High |
| 942 | Allam–2014 | Schistosomiasis does not affect the outcome of HCV infection in genotype 4-infected patients | 2012—2013 | Fever Series | PCR | Parasites | 1 | Low |
| 943 | Andayi–2014 | A sero-epidemiological study of arboviral fevers in Djibouti, horn of Africa | 2010—2011 | Seroprevalance study | Serological | Viruses | 7 | Moderate |
| 944 | Andrews–2014 | Simplified severe sepsis protocol: a randomized controlled trial of modified early goal-directed therapy in Zambia | 2012—2012 | Fever Series | Culture | Bacteria | 3 | Low |
| 945 | Andualem–2014 | A comparative study of Widal test with blood culture in the diagnosis of typhoid fever in febrile patients | 2010—2011 | Fever Series | Culture | Bacteria | 2 | Low |
| 946 | Angelakis–2014 | Coxiella burnetii-positive PCR in febrile patients in rural and urban Africa | 2010—2012 | Fever Series | PCR | Bacteria | 1 | Low |
| 947 | World Health Organization–2014 | Yellow fever in Africa and South America, 2013 | 2013—2013 | Case Series | Serological | Viruses | 1 | High |
| 948 | World Health Organization–2015 | Yellow fever in Africa and the Americas, 2014 | 2014—2014 | Case Series | Serological | Viruses | 1 | High |
| 949 | Aouam–2015 | Epidemiological, clinical and laboratory features of murine typhus in central Tunisia | 2006—2011 | Seroprevalance study | Serological | Bacteria | 1 | Moderate |
| 950 | Aoussi–2014 | Seven native cases of dengue in Abidjan, Ivory Coast | 2010—2010 | Seroprevalance study | Serological | Viruses | 1 | Moderate |
| 951 | Staples–2014 | Yellow fever risk assessment in the Central African Republic | 2009—2009 | Seroprevalance study | Serological | Viruses | 2 | Moderate |
| 952 | Araya Gebreyesus–2015 | Diagnosis and treatment of typhoid fever and associated prevailing drug resistance in Northern Ethiopia | 2014—2014 | Fever Series | Culture | Bacteria | 8 | Low |
| 953 | Bassene–2015 | High Prevalence of Mansonella perstans Filariasis in Rural Senegal | 2010—2010 | Fever Series | PCR | Parasites | 1 | Low |
| 954 | Klempa–2013 | Seroepidemiological study reveals regional co-occurrence of Lassa- and Hantavirus antibodies in Upper Guinea, West Africa | 2004—2004 | Seroprevalance study | Serological | Viruses | 1 | Moderate |
| 955 | Garin–2014 | Autochthonous melioidosis in humans, Madagascar, 2012 and 2013 | 2012—2013 | Case Series | Culture | Bacteria | 1 | High |
| 956 | Chaari–2014 | Childhood rubella encephalitis: diagnosis, management, and outcome | 2011—2011 | Seroprevalance study | Serological | Viruses | 1 | Moderate |
| 957 | Odhiambo–2015 | Orthobunyavirus antibodies among humans in selected parts of the Rift Valley and northeastern Kenya | 2009—2012 | Fever Series | Serological | Viruses | 1 | Moderate |
| 958 | Fezaa–2014 | Serological and molecular detection of Toscana and other phleboviruses in patients and sandflies in Tunisia | 2011—2012 | Fever Series | Serological | Viruses | 1 | Moderate |
| 959 | Yuill–2013 | Latest outbreak news from ProMED-mail. Yellow fever outbreak - Darfur Sudan and Chad. (Special Issue: Focus on virology.) | 2013—2013 | Case Series | PCR | Viruses | 1 | High |
| 960 | El-Azm–2013 | Can brucellosis influence the course of chronic hepatitis C in dual infection? | 2008—2011 | Fever Series | Serological | Bacteria | 1 | Moderate |
| 961 | Njeru–2015 | Rubella outbreak in a Rural Kenyan District, 2014: documenting the need for routine rubella immunization in Kenya | 2013—2013 | Case Series | Serological | Viruses | 1 | High |
| 962 | Awoke–2014 | Bacteriological profile of burn patients at Yekatit 12 Hospital Burn Center, Ethiopia: a longitudinal study | 2010—2011 | Fever Series | Culture | Bacteria | 2 | Low |
| 963 | Aworh–2013 | Human brucellosis: seroprevalence and associated exposure factors among abattoir workers in Abuja, Nigeria - 2011 | 2010—2011 | Fever Series | Serological | Bacteria | 1 | Moderate |
| 964 | Awosanya–2013 | Factors associated with probable cluster of leptospirosis among kennel workers in Abuja, Nigeria | 2009—2009 | Fever Series | Serological | Bacteria | 1 | Moderate |
| 965 | Aziz–2013 | Influence of multi drug resistance Gram negative bacteria in liver transplant recipient | 2012—2013 | Case Series | Culture | Bacteria | 3 | High |
| 966 | Babaniyi–2015 | Risk assessment for yellow Fever in Western and north-Western provinces of zambia | 2013—2013 | Seroprevalance study | Serological | Viruses | 1 | Moderate |
| 967 | Bah–2015 | Clinical presentation of patients with Ebola virus disease in Conakry, Guinea | 2014—2014 | Fever Series | PCR | Viruses | 1 | Low |
| 968 | Bahemia–2015 | Microbiology and antibiotic resistance in severe burns patients: a 5 year review in an adult burns unit | 2008—2012 | Fever Series | Culture | Bacteria/Fungi | 11 | Low |
| 969 | Baize–2014 | Emergence of Zaire Ebola virus disease in Guinea | 2014—2014 | Fever Series | PCR | Viruses | 1 | Low |
| 970 | Balbaa–2013 | Incidence of nosocomial blood stream infection (BSI) in intensive care units (ICUs) in Cairo and Beni-Suef University Hospitals | 2011—2012 | Fever Series | Culture | Bacteria/Fungi | 12 | Low |
| 971 | Ballen–2015 | First report of a Klebsiella pneumoniae ST466 strain causing neonatal sepsis harbouring the blaCTX-M-15 gene in Rabat, Morocco | 2013—2013 | Fever Series | Culture | Bacteria | 1 | Low |
| 972 | Barry–2014 | Ebola outbreak in Conakry, Guinea: epidemiological, clinical, and outcome features | 2014—2014 | Case Series | PCR | Viruses | 1 | High |
| 973 | Bar-Zeev–2015 | Minimum incidence of adult invasive pneumococcal disease in Blantyre, Malawi an urban african setting: a hospital based prospective cohort study | 2005—2006 | Fever Series | Culture | Bacteria/Fungi | 4 | Low |
| 974 | Bazira–2014 | Trends in antimicrobial resistance of Staphylococcus aureus isolated from clinical samples at Mbarara Regional Referral Hospital in rural Uganda | 2003—2012 | Case Series | Culture | Bacteria | 1 | High |
| 975 | Belbel–2014 | Outbreak of an armA methyltransferase-producing ST39 Klebsiella pneumoniae clone in a pediatric Algerian Hospital | 2010—2011 | Case Series | Culture | Bacteria | 1 | High |
| 976 | Biggs–2013 | Estimating leptospirosis incidence using hospital-based surveillance and a population-based health care utilization survey in Tanzania | 2011—2011 | Fever Series | PCR | Bacteria | 1 | Low |
| 977 | Birhanie–2014 | Malaria, Typhoid Fever, and Their Coinfection among Febrile Patients at a Rural Health Center in Northwest Ethiopia: A Cross-Sectional Study | 2013—2013 | Fever Series | Culture/Serological | Bacteria | 1 | Low |
| 978 | Bosman–2013 | Early onset meningococcal meningitis | 2012—2012 | Case Series | Culture | Bacteria | 1 | High |
| 979 | Bouchal–2015 | Bacterial meningoencephalitis vasculitis revealing a pituitary adenoma | 2014—2014 | Case Series | Culture | Bacteria | 1 | High |
| 980 | Bouchekoua–2014 | Visceral leishmaniasis after kidney transplantation: report of a new case and a review of the literature | 2013—2013 | Case Series | Culture | Parasites | 1 | High |
| 981 | Browne–2015 | Notes from the field: hepatitis E outbreak among refugees from South Sudan - Gambella, Ethiopia, April 2014-January 2015 | 2014—2014 | Fever Series | PCR | Viruses | 1 | Low |
| 982 | Centers for Disease Control and Prevention–2013 | Ongoing dengue epidemic - Angola, June 2013 | 2013—2013 | Fever Series | PCR | Viruses | 1 | Low |
| 983 | Chipwaza–2015 | Prevalence of bacterial febrile illnesses in children in Kilosa district, Tanzania | 2013—2013 | Fever Series | Serological | Bacteria | 3 | Moderate |
| 984 | Chouchene–2015 | Hematologic abnormalities in infantile visceral leishmaniasis | 2000—2013 | Case Series | Culture | Parasites | 1 | High |
| 985 | Christopher–2013 | Bacteremia and resistant gram-negative pathogens among under-fives in Tanzania | 2011—2012 | Fever Series | Culture | Bacteria | 8 | Low |
| 986 | Cutland–2015 | Increased risk for group B Streptococcus sepsis in young infants exposed to HIV, Soweto, South Africa, 2004-2008 | 2004—2008 | Case Series | Culture | Bacteria | 1 | High |
| 987 | Dagnew–2013 | Bacterial profile and antimicrobial susceptibility pattern in septicemia suspected patients attending Gondar University Hospital, Northwest Ethiopia | 2006—2012 | Fever Series | Culture | Bacteria | 10 | Low |
| 988 | Dahmane–2014 | Constraints in the diagnosis and treatment of Lassa Fever and the effect on mortality in hospitalized children and women with obstetric conditions in a rural district hospital in Sierra Leone | 2011—2012 | Fever Series | Serological | Viruses | 1 | Moderate |
| 989 | Dallatomasina–2015 | Ebola outbreak in rural West Africa: epidemiology, clinical features and outcomes | 2014—2014 | Fever Series | PCR | Viruses | 1 | Low |
| 990 | Dangor–2015 | Burden of invasive group B Streptococcus disease and early neurological sequelae in South African infants | 2012—2014 | Case Series | Culture | Bacteria | 1 | High |
| 991 | Dia–2013 | [Serotype and antimicrobial susceptibility patterns of Streptococcus pneumoniae isolates in Senegal between 1996 and 2010] | 1996—2010 | Case Series | Culture | Bacteria | 1 | High |
| 992 | Diallo–2013 | Yellow fever outbreak in central part of Senegal 2002: epidemiological findings | 2002—2002 | Seroprevalance study | PCR | Viruses | 1 | Moderate |
| 993 | Diatta–2014 | Prevalence of Bartonella quintana in patients with fever and head lice from rural areas of Sine-Saloum, Senegal | 2010—2011 | Fever Series | PCR | Bacteria | 1 | Low |
| 994 | Diro–2015 | Clinical aspects of paediatric visceral leishmaniasis in North-west Ethiopia | 2011—2012 | Case Series | Culture | Parasites | 1 | High |
| 995 | Dixon–2014 | Ebola viral disease outbreak - West Africa, 2014 | 2014—2014 | Case Series | PCR | Viruses | 1 | High |
| 996 | Dongo–2013 | Lassa fever presenting as acute abdomen: a case series | 2009—2012 | Case Series | PCR | Viruses | 1 | High |
| 997 | Dramowski–2015 | Trends in paediatric bloodstream infections at a South African referral hospital | 2008—2013 | Fever Series | Culture | Bacteria/Viruses | 27 | Low |
| 998 | Dubula–2015 | Spectrum of infections and outcome among hospitalized South Africans with systemic lupus erythematosus | 2003—2009 | Case Series | Culture | Bacteria | 6 | High |
| 999 | BEN AMEUR–1997 | L'endocardite infectieuse A Coxiella burnetti: A propos d'un cas | 2013—2013 | Case Series | Culture | Bacteria/Fungi | 2 | High |
| 1000 | BEN BRAHIM–2006 | Une fiÃ¨vre boutonneuse mÃ©diterranÃ©enne compliquÃ©e d'une embolie pulmonaire | 2013—2013 | Case Series | PCR | Parasites | 1 | High |
| 1001 | El-Amin–2013 | The underlying aetiologies of coma in febrile Sudanese children | 2011—2011 | Fever Series | Culture/PCR | Bacteria/Viruses | 3 | Low |
| 1002 | Elduma–2014 | Dengue and hepatitis E virus infection in pregnant women in Eastern Sudan, a challenge for diagnosis in an endemic area | 2012—2012 | Fever Series | PCR | Viruses | 2 | Low |
| 1003 | Ellis–2015 | A household serosurvey to estimate the magnitude of a dengue outbreak in Mombasa, Kenya, 2013 | 2013—2013 | Seroprevalance study | Serological | Viruses | 3 | Moderate |
| 1004 | El-Mahallawy–2014 | Source, pattern and antibiotic resistance of blood stream infections in hematopoietic stem cell transplant recipients | 2009—2009 | Fever Series | Culture | Bacteria | 11 | Low |
| 1005 | El-Tras–2013 | Seroprevalence of hepatitis E virus in humans and geographically matched food animals in Egypt | 2010—2011 | Seroprevalance study | Serological | Viruses | 1 | Moderate |
| 1006 | Faari–2015 | Prevalence of extended spectrum beta-lactamase-producing Klebsiella species at the University of Ilorin Teaching Hospital | 2009—2009 | Case Series | Culture | Bacteria | 2 | High |
| 1007 | Faneye–2015 | Measles Virus Infection Among Vaccinated and Unvaccinated Children in Nigeria | 2011—2012 | Fever Series | Serological | Viruses | 1 | Moderate |
| 1008 | Fortuin-de Smidt–2015 | Staphylococcus aureus bacteraemia in Gauteng academic hospitals, South Africa | 2012—2013 | Case Series | Culture | Bacteria | 2 | High |
| 1009 | Frickmann–2015 | 16S rRNA gene sequence-based identification of bacteria in automatically incubated blood culture materials from tropical sub-Saharan Africa | 2007—2009 | Fever Series | Culture/PCR | Bacteria | 14 | Low |
| 1010 | Pillai–2015 | Prevalence and patterns of infection in critically ill trauma patients admitted to the trauma ICU, South Africa | 2011—2012 | Case Series | Culture | Bacteria | 4 | High |
| 1011 | Heymann–1980 | Ebola hemorrhagic fever: Tandala, Zaire, 1977-1978 | 1977—1978 | Fever Series | Serological | Viruses | 1 | Moderate |
| 1012 | Milledge–2005 | Aetiology of neonatal sepsis in Blantyre, Malawi: 1996-2001 | 1996—2001 | Fever Series | Culture | Bacteria | 9 | Low |
| 1013 | Pelkonen–2008 | Acute childhood bacterial meningitis in Luanda, Angola | 2004—2004 | Fever Series | Culture | Bacteria | 5 | Low |
| 1014 | van den Ende–1986 | An analysis of blood culture isolates from 7 South African teaching hospital centres | 1978—1978 | Case Series | Culture | Bacteria/Fungi | 31 | High |
| 1015 | Froeschl–2015 | Pox-like lesions and haemorrhagic fever in two concurrent cases in the Central African Republic: case investigation and management in difficult circumstances | 2012—2012 | Case Series | PCR | Viruses | 1 | High |
| 1016 | Gadallah–2014 | Surveillance of health care-associated infections in a tertiary hospital neonatal intensive care unit in Egypt: 1-year follow-up | 2012—2012 | Fever Series | Culture | Bacteria/Fungi | 11 | Low |
| 1017 | Gnassingbe–2013 | Acute cholecystitis from typhic origin in children | 2006—2010 | Case Series | Serological | Bacteria | 1 | High |
| 1018 | Gomard–2014 | Serologic evidence of leptospirosis in humans, Union of the Comoros, 2011 | 2011—2011 | Seroprevalance study | Serological | Bacteria | 1 | Moderate |
| 1019 | Gudo–2015 | Serological Evidence of Chikungunya Virus among Acute Febrile Patients in Southern Mozambique | 2013—2013 | Fever Series | Serological | Viruses | 1 | Moderate |
| 1020 | Harchay–2013 | Epidemic diffusion of Klebsiella pneumoniae isolates producing extended-spectrum beta-lactamases in neonatal and pediatric wards in Rabta hospital of Tunisia | 2007—2008 | Case Series | Culture | Bacteria | 1 | High |
| 1021 | Mallewa–2013 | Viral CNS infections in children from a malaria-endemic area of Malawi: a prospective cohort study | 2002—2004 | Fever Series | PCR | Viruses | 7 | Low |
| 1022 | Mahende–2014 | Aetiology of acute febrile episodes in children attending Korogwe District Hospital in north-eastern Tanzania | 2013—2013 | Fever Series | Culture | Bacteria | 5 | Low |
| 1023 | Kamoun–2015 | Neonatal purulent meningitis in southern Tunisia: Epidemiology, bacteriology, risk factors and prognosis | 1990—2012 | Case Series | Culture | Bacteria | 10 | High |
| 1024 | Kasim–2014 | Nosocomial infections in a neonatal intensive care unit | 2012—2013 | Case Series | Culture | Bacteria | 6 | High |
| 1025 | Katangwe–2013 | Human melioidosis, Malawi, 2011 | 2011—2011 | Case Series | Culture | Bacteria | 1 | High |
| 1026 | Kateera–2015 | Hepatitis B and C seroprevalence among health care workers in a tertiary hospital in Rwanda | 2013—2013 | Fever Series | Serological | Viruses | 2 | Moderate |
| 1027 | Khattab–2013 | Ventilator associated pneumonia in a neonatal intensive care unit | 2012—2012 | Fever Series | Culture | Bacteria/Fungi | 5 | Low |
| 1028 | Kilmarx–2014 | Ebola virus disease in health care workers--Sierra Leone, 2014 | 2014—2014 | Case Series | PCR | Viruses | 1 | High |
| 1029 | Kishk–2014 | Pattern of blood stream infections within neonatal intensive care unit, Suez Canal University Hospital, Ismailia, Egypt | 2013—2013 | Fever Series | Culture/PCR | Bacteria/Viruses/Fungi | 13 | Low |
| 1030 | Kishk–2014 | Efflux pump genes and chlorhexidine resistance: clue for Klebsiella pneumoniae infections in intensive care units, Egypt | 2012—2013 | Case Series | Culture | Bacteria | 1 | High |
| 1031 | KOKO–2013 | Trypanosomose humaine africaine rÃ©vÃ©lÃ©e par une fiÃ¨vre prolongÃ©e : Ã  propos de trois cas pÃ©diatriques | 2011—2012 | Case Series | Serological | Parasites | 1 | High |
| 1032 | Koubaa–2014 | Spinal brucellosis in South of Tunisia: review of 32 cases | 1990—2010 | Fever Series | Culture/Serological | Bacteria | 1 | Low |
| 1033 | Kwallah–2015 | Seroprevalence of yellow fever virus in selected health facilities in Western Kenya from 2010 to 2012 | 2010—2012 | Seroprevalance study | Serological | Viruses | 1 | Moderate |
| 1034 | LaBeaud–2015 | High rates of o'nyong nyong and chikungunya virus transmission in coastal Kenya | 2009—2009 | Seroprevalance study | Serological | Viruses | 2 | Moderate |
| 1035 | L'Azou–2015 | Dengue: etiology of acute febrile illness in Abidjan, Cote d'Ivoire, in 2011-2012 | 2011—2012 | Fever Series | Serological | Viruses | 1 | Moderate |
| 1036 | Lernout–2013 | Rift valley fever in humans and animals in Mayotte, an endemic situation? | 2010—2011 | Seroprevalance study | Serological | Viruses | 1 | Moderate |
| 1037 | Lobna–2014 | Sero diagnosis of brucellosis by using simple and rapid field tests with emphasis on some possible risk factors in humans | 2012—2012 | Fever Series | Serological | Bacteria | 1 | Moderate |
| 1038 | Lu–2015 | Ebola Virus Outbreak Investigation, Sierra Leone, September 28-November 11, 2014 | 2014—2014 | Fever Series | PCR | Viruses | 1 | Low |
| 1039 | Lunguya–2013 | Antimicrobial resistance in invasive non-typhoid Salmonella from the Democratic Republic of the Congo: emergence of decreased fluoroquinolone susceptibility and extended-spectrum beta lactamases | 2007—2011 | Fever Series | Culture | Bacteria | 6 | Low |
| 1040 | Maganga–2014 | Ebola virus disease in the Democratic Republic of Congo | 2014—2014 | Case Series | PCR | Viruses | 1 | High |
| 1041 | Mandomando–2015 | Invasive Salmonella infections among children from rural Mozambique, 2001-2014. (Special Issue: Invasive Salmonella disease in Africa.) | 2001—2014 | Fever Series | Culture | Bacteria | 3 | Low |
| 1042 | Markoff–2013 | Yellow fever outbreak in Sudan | 2012—2012 | Seroprevalance study | PCR | Viruses | 1 | Moderate |
| 1043 | Massenet–2013 | Epidemiologic pattern of meningococcal meningitis in northern Cameroon in 2007-2010: contribution of PCR-enhanced surveillance | 2007—2010 | Fever Series | PCR | Bacteria | 4 | Low |
| 1044 | Mazaba-Liwewe–2014 | First sero-prevalence of dengue fever specific immunoglobulin G antibodies in Western and North-Western provinces of Zambia: a population based cross | 2010—2010 | Seroprevalance study | Serological | Viruses | 1 | Moderate |
| 1045 | McKay–2015 | Community- versus healthcare-acquired bloodstream infections at Groote Schuur Hospital, Cape Town, South Africa | 2011—2012 | Case Series | Culture | Bacteria/Fungi | 17 | High |
| 1046 | Mkony–2014 | Management of neonatal sepsis at Muhimbili National Hospital in Dar es Salaam: diagnostic accuracy of C-reactive protein and newborn scale of sepsis and antimicrobial resistance pattern of etiological bacteria | 2012—2013 | Fever Series | Culture | Bacteria | 5 | Low |
| 1047 | Mourembou–2015 | Mansonella, including a Potential New Species, as Common Parasites in Children in Gabon | 2012—2013 | Fever Series | PCR | Parasites | 1 | Low |
| 1048 | Mourembou–2015 | Possible Role of Rickettsia felis in Acute Febrile Illness among Children in Gabon | 2013—2014 | Fever Series | PCR | Bacteria | 1 | Low |
| 1049 | Moyen–2014 | First reported chikungunya fever outbreak in the republic of Congo, 2011 | 2011—2011 | Fever Series | PCR | Viruses | 1 | Low |
| 1050 | Moyen–2015 | Risk factors associated with Ebola and Marburg viruses seroprevalence in blood donors in the Republic of Congo | 2011—2011 | Seroprevalance study | Serological | Viruses | 2 | Moderate |
| 1051 | Mudau–2013 | Outbreak of multi-drug resistant Pseudomonas aeruginosa bloodstream infection in the haematology unit of a South African Academic Hospital | 2010—2011 | Case Series | Culture | Bacteria | 1 | High |
| 1052 | Mudzikati–2015 | Neonatal septicaemia: prevalence and antimicrobial susceptibility patterns of common pathogens at Princess Marina Hospital, Botswana | 2012—2012 | Fever Series | Culture | Bacteria | 11 | Low |
| 1053 | Mueller–2015 | Prevalence of hepatitis B virus infection among health care workers in a tertiary hospital in Tanzania | 2012—2012 | Fever Series | PCR | Viruses | 1 | Low |
| 1054 | Mwongula–2013 | Seroprevalence of Chikungunya infection in pyretic children seeking treatment in Alupe District Hospital, Busia County Kenya | 2010—2010 | Fever Series | Serological | Viruses | 1 | Moderate |
| 1055 | Naidoo–2013 | Epidemiology of Staphylococcus aureus bacteraemia at a tertiary children's hospital in Cape Town, South Africa | 2007—2011 | Case Series | Culture | Bacteria | 2 | High |
| 1056 | Ndlangisa–2014 | Population snapshot of Streptococcus pneumoniae causing invasive disease in South Africa prior to introduction of pneumococcal conjugate vaccines | 2007—2007 | Case Series | Culture | Bacteria | 1 | High |
| 1057 | Nyenswah–2014 | Ebola epidemic--Liberia, March-October 2014. | 2014—2014 | Fever Series | PCR | Viruses | 1 | Low |
| 1058 | Obaro–2015 | Salmonella bacteremia among children in central and northwest Nigeria, 2008-2015. | 2008—2015 | Fever Series | Culture | Bacteria | 2 | Low |
| 1059 | Obeng-Nkrumah–2013 | High levels of extended-spectrum beta-lactamases in a major teaching hospital in Ghana: the need for regular monitoring and evaluation of antibiotic resistance | 2008—2008 | Case Series | Culture | Bacteria | 7 | High |
| 1060 | Oluyege–2015 | Isolation and characterization of Salmonella typhi from widal positive patients attending Ekiti State University Teaching Hospital | 2011—2011 | Fever Series | Culture | Bacteria | 9 | Low |
| 1061 | Osuorah–2015 | Outbreak of serotype W135 Neisseria meningitidis in central river region of the Gambia between February and June 2012: a hospital-based review of paediatric cases | 2012—2012 | Fever Series | Culture | Bacteria | 2 | Low |
| 1062 | Oyero–2014 | High dengue NS1 antigenemia in febrile patients in Ibadan, Nigeria | 2013—2013 | Fever Series | PCR | Viruses | 1 | Low |
| 1063 | Peterside–2015 | Bacteriological profile and antibiotic susceptibility pattern of neonatal sepsis at a teaching hospital in Bayelsa State, Nigeria | 2011—2013 | Fever Series | Culture | Bacteria | 6 | Low |
| 1064 | Poirel–2013 | Occurrence of OXA-48 and VIM-1 carbapenemase-producing Enterobacteriaceae in Egypt | 2009—2010 | Case Series | Culture | Bacteria | 2 | High |
| 1065 | Preziosi–2015 | A prospective observational study of bacteraemia in adults admitted to an urban Mozambican hospital | 2011—2012 | Fever Series | Culture | Bacteria | 15 | Low |
| 1066 | Rafai–2015 | Meningoencephalitis by Streptococcus agalactiae in the immunocompetent adult | 2014—2014 | Case Series | Culture | Bacteria | 1 | High |
| 1067 | Ratmanov–2013 | The correlation of Q fever and Coxiella burnetii DNA in household environments in rural Senegal | 2008—2011 | Fever Series | PCR | Bacteria | 1 | Low |
| 1068 | Rerambiah–2014 | Antimicrobial profiles of bacterial clinical isolates from the Gabonese National Laboratory of Public Health: data from routine activity | 2010—2010 | Case Series | Culture | Bacteria | 19 | High |
| 1069 | Riabi–2014 | An outbreak of West Nile Virus infection in the region of Monastir, Tunisia, 2003 | 2003—2003 | Fever Series | PCR | Viruses | 1 | Low |
| 1070 | Rovira–2015 | The challenge of diagnosing and treating Staphylococcus aureus invasive infections in a resource-limited sub-Saharan Africa setting: a case report | 2014—2014 | Case Series | Culture | Bacteria | 1 | High |
| 1071 | Rubach–2015 | Etiologies of illness among patients meeting integrated management of adolescent and adult illness district clinician manual criteria for severe infections in northern Tanzania: implications for empiric antimicrobial therapy | 2007—2008 | Seroprevalance study | PCR | Bacteria/Fungi/viruses | 8 | Moderate |
| 1072 | Sakhria–2013 | Co-circulation of Toscana virus and Punique virus in northern Tunisia: a microneutralisation-based seroprevalence study | 2011—2011 | Seroprevalance study | Serological | Viruses | 1 | Moderate |
| 1073 | Savadogo–2015 | A haemorrhagic stroke revealing meningitis due to Neisseria meningitidis | 2014—2014 | Case Series | Culture | Bacteria | 1 | High |
| 1074 | Seboxa–2015 | High Mortality from Blood Stream Infection in Addis Ababa, Ethiopia, Is Due to Antimicrobial Resistance | 2012—2013 | Fever Series | Culture | Bacteria | 3 | Low |
| 1075 | Sini–2013 | Miller Fisher syndrome with negative anti-GQ1b antibodies in Mycoplasma pneumoniae pneumonia | 2012—2012 | Case Series | Serological | Bacteria | 1 | High |
| 1076 | Sogbanmu–2014 | Management of cryptococcal meningitis in adults at Mthatha Hospital Complex, Eastern Cape, South Africa | 2011—2012 | Case Series | Culture | Fungi | 1 | High |
| 1077 | Sokhna–2013 | Point-of-care laboratory of pathogen diagnosis in rural Senegal | 2011—2012 | Fever Series | PCR | Bacteria | 4 | Low |
| 1078 | Sothmann–2015 | Urbanicity and Paediatric Bacteraemia in Ghana-A Case-Control Study within a Rural-Urban Transition Zone | 2012—2012 | Fever Series | Culture | Bacteria | 3 | Low |
| 1079 | Sow–2014 | Rift Valley fever outbreak, southern Mauritania, 2012 | 2012—2012 | Seroprevalance study | PCR | Viruses | 1 | Moderate |
| 1080 | Bukbuk–2014 | Development and validation of serological assays for viral hemorrhagic fevers and determination of the prevalence of Rift Valley fever in Borno State, Nigeria | 2011—2012 | Fever Series | Serological | Viruses | 3 | Moderate |
| 1081 | Caron–2013 | First evidence of simultaneous circulation of three different dengue virus serotypes in Africa | 2007—2010 | Seroprevalance study | PCR | Viruses | 3 | Moderate |
| 1082 | Depasse–2013 | Infections in hospitalized children and young adults with acute leukemia in Morocco | 2011—2011 | Fever Series | Culture | Bacteria/Fungi | 6 | Low |
| 1083 | Dietz–2015 | Epidemiology and Risk Factors for Ebola Virus Disease in Sierra Leone-23 May 2014 to 31 January 2015 | 2014—2015 | Case Series | PCR | Viruses | 1 | High |
| 1084 | El Ekiaby–2015 | Viremia levels in hepatitis C infection among Egyptian blood donors and implications for transmission risk with different screening scenarios | 2007—2010 | Fever Series | PCR | Viruses | 1 | Low |
| 1085 | Hamer–2015 | Etiology of bacteremia in young infants in six countries | 2014—2014 | Case Series | Culture | Bacteria | 5 | High |
| 1086 | Hecini-Hannachi–2014 | Serotypes and antimicrobial resistance of invasive Streptococcus pneumoniae isolates from East Algeria (2005-2011) | 2005—2011 | Case Series | Culture | Bacteria | 1 | High |
| 1087 | Ikama–2013 | [Profile of infective endocarditis at Brazzaville University Hospital] | 2001—2009 | Fever Series | Culture | Bacteria | 3 | Low |
| 1088 | Irenge–2015 | Antimicrobial resistance of bacteria isolated from patients with bloodstream infections at a tertiary care hospital in the Democratic Republic of the Congo | 2013—2014 | Fever Series | Culture | Bacteria | 13 | Low |
| 1089 | Lochan–2013 | Blood cultures in sick children | 2012—2012 | Fever Series | Culture | Bacteria/Fungi | 15 | Low |
| 1090 | Makani–2015 | Bacteraemia in sickle cell anaemia is associated with low haemoglobin: a report of 890 admissions to a tertiary hospital in Tanzania | 2006—2008 | Fever Series | Culture | Bacteria | 11 | Low |
| 1091 | Phoba–2014 | Epidemic increase in Salmonella bloodstream infection in children, Bwamanda, the Democratic Republic of Congo | 2011—2012 | Fever Series | Culture | Bacteria | 7 | Low |
| 1092 | Sow–2014 | Rift Valley fever in Kedougou, southeastern Senegal, 2012 | 2012—2012 | Seroprevalance study | PCR | Viruses | 1 | Moderate |
| 1093 | Suleiman–2015 | Rubella antibody avidity among rubella seropositive women attending a tertiary care facility in Nigeria | 2012—2012 | Seroprevalance study | Serological | Viruses | 1 | Moderate |
| 1094 | Suliman–2013 | An outbreak of Babesiosis in the military area of Kapoita town | 1994—1994 | Fever Series | Culture | Parasites | 1 | Low |
| 1095 | Swann–2014 | Bacterial meningitis in Malawian infants <2 months of age: etiology and susceptibility to World Health Organization first-line antibiotics | 2002—2008 | Case Series | Culture | Bacteria | 22 | High |
| 1096 | Tantawy–2013 | Pathogenesis and prognosis of neutropenia in infants and children admitted in a university children hospital in Egypt | 2010—2010 | Fever Series | Serological | Viruses | 2 | Moderate |
| 1097 | Thairu–2014 | Antimicrobial susceptibility pattern of Haemophilus influenzae among under-five children presenting at the emergency paediatric unit (EPU) of two teaching hospitals in Jos, Nigeria | 2009—2010 | Fever Series | Culture | Bacteria | 7 | Low |
| 1098 | Trabelsi–2015 | Fungemia caused by Yarrowia lipolytica | 2012—2014 | Case Series | Culture | Bacteria | 1 | High |
| 1099 | van der Hoek–2013 | Short communication: prevalence of antibodies against Coxiella burnetii (Q fever) in children in The Gambia, West Africa | 2008—2008 | Fever Series | Serological | Bacteria | 1 | Moderate |
| 1100 | Venter–2014 | Macroarray assay for differential diagnosis of meningoencephalitis in southern Africa | 2013—2013 | Case Series | PCR | Bacteria/Viruses | 5 | High |
| 1101 | Verani–2015 | Burden of invasive nontyphoidal Salmonella disease in a rural and urban site in Kenya, 2009-2014. | 2009—2014 | Fever Series | Culture | Bacteria | 2 | Low |
| 1102 | Waitt–2015 | Sepsis carries a high mortality among hospitalised adults in Malawi in the era of antiretroviral therapy scale-up: a longitudinal cohort study | 2008—2009 | Fever Series | Culture | Bacteria/Fungi | 6 | Low |
| 1103 | Wasihun–2015 | Bacteriological profile and antimicrobial susceptibility patterns of blood culture isolates among febrile patients in Mekelle Hospital, Northern Ethiopia | 2014—2014 | Fever Series | Culture | Bacteria | 11 | Low |
| 1104 | Weller–2014 | Seroprevalence of alphavirus antibodies in a cross-sectional study in southwestern Tanzania suggests endemic circulation of chikungunya | 2007—2008 | Seroprevalance study | PCR | Viruses | 1 | Moderate |
| 1105 | Wiersinga–2015 | Clinical, environmental, and serologic surveillance studies of melioidosis in Gabon, 2012-2013 | 2012—2013 | Fever Series | Culture/Serological | Bacteria | 7 | Low |
| 1106 | Witkowski–2015 | Human seroprevalence indicating hantavirus infections in tropical rainforests of Cote d'Ivoire and Democratic Republic of Congo | 2007—2007 | Seroprevalance study | Serological | Viruses | 1 | Moderate |
| 1107 | Wolfaardt–2014 | Molecular characterisation of enteroviruses and clinical findings from a cluster of paediatric viral meningitis cases in Tshwane, South Africa 2010-2011 | 2010—2011 | Fever Series | Culture | Viruses | 1 | Low |
| 1108 | Woyessa–2014 | An outbreak of acute febrile illness caused by Sandfly Fever Sicilian Virus in the Afar region of Ethiopia, 2011 | 2011—2011 | Fever Series | PCR | Viruses | 1 | Low |
| 1109 | Yimer–2014 | Prevalence and risk factors of louse-borne relapsing fever in high risk populations in Bahir Dar city Northwest, Ethiopia | 2012—2012 | Fever Series | Culture | Bacteria | 1 | Low |
| 1110 | Yimer–2014 | Louse-borne relapsing fever profile at Felegehiwot referral hospital, Bahir Dar city, Ethiopia: a retrospective study | 2009—2012 | Fever Series | Culture | Bacteria | 1 | Low |
| 1111 | Zeba–2014 | Characterisation of hepatitis C virus genotype among blood donors at the regional blood transfusion centre of Ouagadougou, Burkina Faso | 2011—2011 | Fever Series | PCR | Viruses | 1 | Low |
| 1112 | Znazen–2013 | Murine typhus in Tunisia: a neglected cause of fever as a single symptom | 2006—2008 | Fever Series | Serological | Bacteria | 1 | Moderate |
| 1113 | Alkan–2015 | Virus isolation, genetic characterization and seroprevalence of Toscana virus in Algeria | 2013—2013 | Seroprevalance study | Serological | Viruses | 1 | Moderate |
| 1114 | Bisiwe–2015 | Haemodialysis catheter-related bloodstream infections at Universitas Academic Hospital, Bloemfontein: should we change our empiric antibiotics? | 2012—2012 | Fever Series | Culture | Bacteria/Fungi | 11 | Low |
| 1115 | Bonney–2013 | Hospital-based surveillance for viral hemorrhagic fevers and hepatitides in Ghana | 2009—2011 | Fever Series | PCR | Viruses | 3 | Low |
| 1116 | Bouba–2014 | Neisseria meningitidis susceptibility in North-Cameroon | 2012—2012 | Fever Series | Culture | Bacteria | 1 | Low |
| 1117 | Boushab–2015 | [Severe hemorrhagic form of Rift Valley Fever in Mauritania] | 2012—2012 | Case Series | PCR | Viruses | 1 | High |
| 1118 | Boyles–2015 | Blood cultures taken from patients attending emergency departments in South Africa are an important antibiotic stewardship tool, which directly influences patient management | 2013—2013 | Fever Series | Culture | Bacteria/Fungi | 14 | Low |
| 1119 | Chechet–2014 | Seroprevalence of measles IgM in children 5-12 years from selected primary schools in Giwa Local Government Area, Zaria, Kaduna state | 2012—2012 | Fever Series | Serological | Viruses | 1 | Moderate |
| 1120 | Chipwaza–2014 | Dengue and Chikungunya fever among viral diseases in outpatient febrile children in Kilosa district hospital, Tanzania | 2013—2013 | Seroprevalance study | Serological | Viruses | 2 | Moderate |
| 1121 | Christiana–2014 | Lymphatic filariasis and associated morbidities in rural communities of Ogun State, Southwestern Nigeria | 2013—2013 | Fever Series | Culture | Parasites | 1 | Low |
| 1122 | Chukwuemeka–2014 | Quality assurance in blood culture: a retrospective study of blood culture contamination rate in a tertiary hospital in Nigeria | 2010—2011 | Fever Series | Culture | Bacteria/Fungi | 10 | Low |
| 1123 | D'Acremont–2014 | Beyond malaria--causes of fever in outpatient Tanzanian children | 2008—2008 | Fever Series | Culture/PCR | Bacteria/Viruses | 13 | Low |
| 1124 | Dawang–2015 | Occurrence and antibiogram of Salmonella isolates from patients attending Bingham Hospital, Jos, Nigeria | 2011—2012 | Fever Series | Culture | Bacteria | 1 | Low |
| 1125 | Dean–2013 | Epidemiology of brucellosis and q Fever in linked human and animal populations in northern togo | 2012—2012 | Fever Series | Serological | Bacteria | 2 | Moderate |
| 1126 | El-Sahrigy–2015 | Nosocomial infection in an Egyptian neonatal intensive care unit | 2009—2010 | Fever Series | Culture | Bacteria/Fungi | 5 | Low |
| 1127 | Endris–2014 | Bacterial sepsis in patients with visceral leishmaniasis in Northwest Ethiopia | Not stated | Fever Series | Culture | Bacteria | 5 | Low |
| 1128 | Fattouh–2014 | Emergence of carbapenem-resistant Acinetobacter baumannii in the intensive care unit in Sohag University Hospital, Egypt | 2013—2014 | Case Series | Culture | Bacteria | 1 | High |
| 1129 | Feasey–2015 | Rapid emergence of multidrug resistant, H58-lineage Salmonella typhi in Blantyre, Malawi | 1998—1998 | Fever Series | Culture | Bacteria | 1 | Low |
| 1130 | Feasey–2015 | Three Epidemics of Invasive Multidrug-Resistant Salmonella Bloodstream Infection in Blantyre, Malawi, 1998-2014 | 1998—2014 | Fever Series | Culture | Bacteria | 4 | Low |
| 1131 | Gebewo–2014 | Sero-prevalence of bovine and human brucellosis in Adami Tulu, central Ethiopia | 2010—2011 | Fever Series | Serological | Bacteria | 1 | Moderate |
| 1132 | Gqunta–2015 | First report of an IMI-2 carbapenemase-producing Enterobacter asburiae clinical isolate in South Africa | 2012—2012 | Case Series | Culture | Bacteria | 2 | High |
| 1133 | Grard–2014 | Zika virus in Gabon (Central Africa)--2007: a new threat from Aedes albopictus? | 2007—2010 | Seroprevalance study | PCR | Viruses | 1 | Moderate |
| 1134 | Gray–2015 | Seroepidemiological Study of Interepidemic Rift Valley Fever Virus Infection Among Persons with Intense Ruminant Exposure in Madagascar and Kenya | 2010—2012 | Seroprevalance study | Serological | Viruses | 1 | Moderate |
| 1135 | Himatt–2015 | Sero-prevalence of dengue infections in the Kassala state in the eastern part of the Sudan in 2011 | 2011—2011 | Seroprevalance study | Serological | Viruses | 1 | Moderate |
| 1136 | Huson–2014 | Methicillin-resistant Staphylococcus aureus as a cause of invasive infections in Central Africa: a case report and review of the literature | 2013—2013 | Case Series | Culture | Bacteria | 1 | High |
| 1137 | Ibegbulam-Njoku–2014 | Prevalence of antibody titre in healthy individual and enteric fever patients in Owerri, Nigeria | 2013—2013 | Fever Series | Serological | Bacteria | 1 | Moderate |
| 1138 | Iroezindu–2015 | Lessons learnt from the management of a case of Lassa fever and follow-up of nosocomial primary contacts in Nigeria during Ebola virus disease outbreak in West Africa | 2014—2014 | Case Series | PCR | Viruses | 1 | High |
| 1139 | Iroha–2015 | Occurrence of extended spectrum beta lactamase producing Escherichia coli from human clinical and wild birds (pigeons, bats, parrots and ducks) samples from Ebonyi State, Nigeria | 2013—2013 | Fever Series | Culture | Bacteria | 1 | Low |
| 1140 | Isendahl–2014 | Prevalence of community-acquired bacteraemia in Guinea-Bissau: an observational study | 2010—2010 | Fever Series | Culture | Bacteria | 8 | Low |
| 1141 | Kariuki–2015 | Ceftriaxone-resistant Salmonella enterica serotype Typhimurium sequence type 313 from Kenyan patients is associated with the blaCTX-M-15 gene on a novel IncHI2 plasmid | 2009—2011 | Case Series | Culture | Bacteria | 1 | High |
| 1142 | Kooli–2014 | Bartonella quintana meningoencephalitis in an immunocompetent: rare case | 2012—2012 | Case Series | Serological | Bacteria | 1 | High |
| 1143 | Kyei–2015 | Imported Lassa fever: a report of 2 cases in Ghana | 2013—2013 | Case Series | PCR | Viruses | 1 | High |
| 1144 | LaBeaud–2015 | Factors associated with severe human Rift Valley fever in Sangailu, Garissa County, Kenya | 2011—2011 | Seroprevalance study | Serological | Viruses | 1 | Moderate |
| 1145 | Labi–2014 | Salmonella blood stream infections in a tertiary care setting in Ghana | 2010—2013 | Fever Series | Culture | Bacteria | 5 | Low |
| 1146 | Labid–2014 | High prevalence of extended spectrum beta-lactamase (ESBL) producers in fatal cases of pediatric septicemia among the Enterobacteriaceae in the pediatric hospital of Annaba, Algeria | 2010—2011 | Case Series | Culture | Bacteria | 4 | High |
| 1147 | Lado–2015 | Clinical features of patients isolated for suspected Ebola virus disease at Connaught Hospital, Freetown, Sierra Leone: a retrospective cohort study | 2014—2014 | Fever Series | PCR | Viruses | 1 | Low |
| 1148 | Mahfoudhi–2015 | Atypical form of Lyme disease | 2014—2014 | Case Series | Serological | Bacteria | 1 | High |
| 1149 | Mamoudou–2014 | Tetanus complicated sepsis on major haemoglobinopathies | 2013—2013 | Case Series | Culture | Bacteria | 1 | High |
| 1150 | Mamvura–2015 | Seroprevalence of rubella virus IgG in pregnant women in Harare, Zimbabwe | 2014—2014 | Seroprevalance study | Serological | Viruses | 1 | Moderate |
| 1151 | Mirkovic–2014 | Importation and containment of ebola virus disease - Senegal, August-September 2014 | 2014—2014 | Case Series | PCR | Viruses | 1 | High |
| 1152 | Morkel–2014 | Bloodstream infections and antimicrobial resistance patterns in a South African neonatal intensive care unit | 2008—2008 | Fever Series | Culture | Bacteria/Fungi | 12 | Low |
| 1153 | Munier–2013 | Frequent transient hepatitis C viremia without seroconversion among healthcare workers in Cairo, Egypt | 2008—2010 | Fever Series | Serological | Viruses | 1 | Moderate |
| 1154 | Mushagalusa–2015 | Bacterial meningitis in children in Lwiro (South-Kivu DRC) | 2012—2013 | Case Series | Culture | Bacteria | 3 | High |
| 1155 | Muthumbi–2015 | Invasive Salmonellosis in Kilifi, Kenya | 1998—2014 | Fever Series | Culture | Bacteria | 1 | Low |
| 1156 | Noden–2014 | Risk assessment of flavivirus transmission in Namibia | 2011—2012 | Seroprevalance study | Serological | Viruses | 1 | Moderate |
| 1157 | Noden–2014 | Exposure and risk factors to Coxiella burnetii, spotted fever group and typhus group rickettsiae, and Bartonella henselae among volunteer blood donors in Namibia | 2011—2012 | Seroprevalance study | Serological | Bacteria | 4 | Moderate |
| 1158 | Ntirenganya–2015 | High prevalence of antimicrobial resistance among common bacterial isolates in a tertiary healthcare facility in Rwanda | 2013—2013 | Case Series | Culture | Bacteria | 9 | High |
| 1159 | Okon–2014 | Retrospective analysis of gram-negative bacteria isolated at a tertiary hospital in Maiduguri, Nigeria | 2007—2011 | Case Series | Culture | Bacteria | 4 | High |
| 1160 | Oneko–2015 | Emergence of community-acquired, multidrug-resistant invasive nontyphoidal Salmonella disease in rural western Kenya, 2009-2013. | 2009—2013 | Fever Series | Culture | Bacteria | 5 | Low |
| 1161 | Onyedibe–2015 | Bacteriologic profile, antibiotic regimen and clinical outcome of neonatal sepsis in a University Teaching Hospital in North Central Nigeria | 2011—2011 | Fever Series | Culture | Bacteria | 11 | Low |
| 1162 | Opajobi–2014 | Antimicrobial susceptibility pattern of some recently isolated serovars of Salmonellae in Jos, Nigeria | 2006—2011 | Fever Series | Culture | Bacteria | 5 | Low |
| 1163 | Orem–2014 | Epstein-Barr virus viral load and serology in childhood non-Hodgkin's lymphoma and chronic inflammatory conditions in Uganda: implications for disease risk and characteristics | 2004—2008 | Fever Series | PCR | Viruses | 1 | Low |
| 1164 | Orth–2013 | Characterisation of Staphylococcus aureus bacteraemia at Tygerberg hospital | 2008—2009 | Case Series | Culture | Bacteria | 1 | High |
| 1165 | Scherbaum–2014 | Incidence, pathogens and resistance patterns of nosocomial infections at a rural hospital in Gabon | 2009—2009 | Fever Series | Culture | Bacteria | 4 | Low |
| 1166 | Schieffelin–2014 | Clinical illness and outcomes in patients with Ebola in Sierra Leone | 2014—2014 | Fever Series | PCR | Viruses | 1 | Low |
| 1167 | Schoepp–2014 | Undiagnosed acute viral febrile illnesses, Sierra Leone | 2006—2008 | Seroprevalance study | Serological | Viruses | 7 | Moderate |
| 1168 | Serghini–2014 | Severe pneumonia with bronchial involvement complicating varicella in an immunocompetent adult | 2013—2013 | Case Series | Serological | Viruses | 1 | High |
| 1169 | Shaffer–2014 | Lassa fever in post-conflict sierra leone | 2008—2012 | Seroprevalance study | Serological | Viruses | 1 | Moderate |
| 1170 | Siawaya–2015 | HIV, HBS, HCV, HTLV and syphilis prevalence among high school learners and university student in Libreville, Gabon | 2013—2014 | Fever Series | Serological | Bacteria/Viruses | 3 | Moderate |
| 1171 | Soghaier–2015 | Cross-sectional community-based study of the socio-demographic factors associated with the prevalence of dengue in the eastern part of Sudan in 2011 | 2011—2011 | Seroprevalance study | Serological | Viruses | 1 | Moderate |
| 1172 | Soghaier–2013 | Dengue fever in a border state between Sudan and Republic of South Sudan: epidemiological perspectives | 2012—2012 | Seroprevalance study | Serological | Viruses | 1 | Moderate |
| 1173 | Storm–2014 | Human cases of Sindbis fever in South Africa, 2006-2010 | 2006—2010 | Seroprevalance study | Serological | Viruses | 1 | Moderate |
| 1174 | Sumaye–2015 | Inter-epidemic acquisition of Rift Valley fever virus in humans in Tanzania | 2012—2012 | Seroprevalance study | Serological | Viruses | 1 | Moderate |
| 1175 | Sweswe-Han–2013 | First detected isolate of glycopeptide-intermediate resistant Staphylococcus aureus in a renal unit at a central academic hospital in KwaZulu-Natal | 2009—2009 | Case Series | Culture | Bacteria | 1 | High |
| 1176 | Tann–2014 | Prevalence of bloodstream pathogens is higher in neonatal encephalopathy cases vs. controls using a novel panel of real-time PCR assays | 2011—2012 | Fever Series | Culture | Bacteria/Viruses | 9 | Low |
| 1178 | Dramowski–2015 | Neonatal nosocomial bloodstream infections at a referral hospital in a middle-income country: burden, pathogens, antimicrobial resistance and mortality. (Special Issue: Neonatal care in low- and middle income countries.) | 2009—2013 | Fever Series | Culture | Bacteria/Fungi | 15 | Low |
| 1179 | Bercion–2008 | Acute bacterial meningitis at the 'Complexe Pediatrique' of Bangui, Central African Republic | 2004—2005 | Fever Series | Culture | Bacteria | 5 | Low |
| 1180 | Amsalu–2005 | Meningitis in children beyond the neonatal period in Gondar University hospital | 1998—2003 | Fever Series | Culture | Bacteria | 6 | Low |
| 1182 | Thiga–2015 | High seroprevalence of antibodies against spotted fever and scrub typhus bacteria in patients with febrile Illness, Kenya | 2014—2014 | Seroprevalance study | Serological | Bacteria | 1 | Moderate |
| 1183 | Tigoi–2015 | Seroepidemiology of selected arboviruses in febrile patients visiting selected health facilities in the lake/river basin areas of Lake Baringo, Lake Naivasha, and Tana River, Kenya | 2009—2012 | Seroprevalance study | Serological | Viruses | 4 | Moderate |
| 1184 | Tobin–2015 | Lassa fever in Nigeria: insights into seroprevalence and risk factors in rural Edo State: a pilot study | 2014—2014 | Fever Series | Serological | Viruses | 1 | Moderate |
| 1185 | Trifa–2014 | Thrombopenia and the nature of the microorganism in infected critically ill children | 2009—2009 | Fever Series | Culture | Bacteria/Fungi | 10 | Low |
| 1186 | Umeh–2014 | Case based rubella surveillance in Abia State, South East Nigeria, 2007-2011 | 2007—2007 | Seroprevalance study | Serological | Viruses | 1 | Moderate |
| 1187 | Weyer–2013 | Human cases of Wesselsbron disease, South Africa 2010-2011 | 2010—2011 | Case Series | PCR | Viruses | 1 | High |
| 1188 | Yan–2015 | Clinical characteristics of 154 patients suspected of having Ebola virus disease in the Ebola holding center of Jui Government Hospital in Sierra Leone during the 2014 Ebola outbreak | 2014—2014 | Fever Series | PCR | Viruses | 1 | Low |
| 1189 | Zaki–2013 | Clinicolaboratory study of mother-to-neonate transmission of hepatitis E virus in Egypt | 2012—2013 | Fever Series | Serological | Viruses | 3 | Moderate |
| 1190 | World Health Organization–2013 | Yellow fever in Africa and South America, 2011-2012 | 2011—2012 | Case Series | PCR/Serological | Viruses | 1 | High |
| 1191 | Abdullahi–2015 | Antibacterial resistance profile and PCR detection of antibiotic resistance genes in Salmonella serovars isolated from blood samples of hospitalized subjects in Kano, North-West, Nigeria | 2011—2013 | Fever Series | Culture | Bacteria | 5 | Low |
| 1192 | Guirguis–1983 | Bacterial meningitis in Egypt: analysis of CSF isolates from hospital patients in Cairo, 1977-78 | Not stated | Fever Series | Culture | Bacteria | 3 | Low |
| 1193 | Idoko–2014 | Serological survey of dengue virus immunoglobulin M among febrile patients in Kaduna metropolis, Nigeria | 2013—2013 | Seroprevalance study | Serological | Viruses | 1 | Moderate |
| 1194 | Kibuuka–2015 | Bacteremia Among Febrile Ugandan Children Treated with Antimalarials Despite a Negative Malaria Test | 2012—2012 | Fever Series | Culture | Bacteria | 4 | Low |
| 1195 | Muiruri–2015 | Cross-sectional survey of Rift Valley fever virus exposure in Bodhei village located in a transitional coastal forest habitat in Lamu county, Kenya | 2006—2006 | Seroprevalance study | Serological | Viruses | 1 | Moderate |
| 1196 | Nebhani–2015 | Tetraplegia revealing a serious pneumococcal meningomyelitis: about a case and literature review | 2014—2014 | Case Series | Culture | Bacteria | 1 | High |
| 1197 | Ochieng–2015 | Bacteria causing ventriculoperitoneal shunt infections in a Kenyan population | 2010—2012 | Fever Series | Culture | Bacteria | 4 | Low |
| 1198 | Rustenhoven-Spaan–2013 | Maternal mortality in a rural Tanzanian hospital: fatal Jarisch-Herxheimer reaction in a case of relapsing fever in pregnancy | 2015—2015 | Case Series | Culture | Bacteria | 1 | High |
| 1199 | Asse–2001 | [Bacterial meningitis in children at the University Hospital Center of Bouake in the Republic of the Ivory Coast] | 1999—2000 | Case Series | Culture | Bacteria | 3 | High |
| 1200 | Ayoola–2003 | Aetiological agents, clinical features and outcome of septicaemia in infants in Ibadan | 1998—1998 | Fever Series | Culture | Bacteria | 7 | Low |
| 1201 | Greco–2001 | [Epidemiological profile of methicillin resistant Staphylococcus epidermidis with diminished sensitivity to teicoplanin and isolated from neutropenic patients at the National Center of Bone Marrow Transplantation in Tunis] | 1998—1998 | Case Series | Culture | Bacteria | 1 | High |
| 1202 | Bitekyerezo–2002 | The outbreak and control of Ebola viral haemorrhagic fever in a Ugandan medical school | 2000—2000 | Case Series | PCR | Viruses | 1 | High |
| 1203 | Christie–1980 | Plague in camels and goats: their role in human epidemics | 1976—1976 | Fever Series | Serological | Bacteria | 1 | Moderate |
| 1204 | El-Shanshory–2013 | Prevalence of hepatitis C infection among children with beta-thalassaemia major in Mid Delta, Egypt: a single centre study | 2010—2011 | Fever Series | Serological | Viruses | 1 | Moderate |
| 1205 | Monath–1980 | Yellow fever in the Gambia, 1978--1979: epidemiologic aspects with observations on the occurrence of orungo virus infections | 1978—1979 | Fever Series | Serological | Viruses | 2 | Moderate |
| 1206 | Sangare–2007 | [Salmonella meningitis in Ouagadougou, Burkina Faso, from 2000 to 2004] | 2000—2004 | Fever Series | Culture | Bacteria | 6 | Low |
| 1207 | Joubert–1985 | Prevalence of hepatitis virus and some arbovirus infections in Kavango, northern SWA/Namibia | 1983—1983 | Seroprevalance study | Serological | Viruses | 6 | Moderate |
| 1208 | Kelly–1993 | Q fever in Zimbabwe. A review of the disease and the results of a serosurvey of humans, cattle, goats and dogs | 1992—1992 | Seroprevalance study | Serological | Bacteria | 1 | Moderate |
| 1209 | Lalloo–1992 | Listeria monocytogenes meningitis at King Edward VIII Hospital, Durban. A 10-year experience, 1981-1990 | 1981—1990 | Case Series | Culture | Bacteria | 1 | High |
| 1210 | Bamford–2007 | Streptococcus pneumoniae infections in neonates | 2005—2006 | Case Series | Culture | Bacteria | 1 | High |
| 1211 | Boushab–2015 | [Survey of investigation around cases of Rift Valley Fever at Tagant, Mauritania] | 2012—2012 | Seroprevalance study | PCR | Viruses | 1 | Moderate |
| 3808 | Hodes–1993 | Endocarditis in Ethiopia. Analysis of 51 cases from Addis Ababa. | 1977—1985 | Case Series | Culture | Bacteria | 5 | High |
| 3810 | Adeyemo–1993 | Klebsiella septicaemia, osteomyelitis and septic arthritis in neonates in Ibadan, Nigeria. | 1990—1990 | Case Series | Not described | Bacteria | 1 | High |
| 3811 | Agbonlahor–1986 | Multiple drug-resistant Citrobacter freundii infection mimicking typhoid fever. | 1984—1984 | Case Series | Culture | Bacteria | 1 | High |
| 3813 | Akpede–1999 | Response to antimicrobial therapy in childhood bacterial meningitis in tropical Africa: report of a bi-centre experience in Nigeria, 1993-1998. | 1993—1998 | Case Series | Culture | Bacteria | 10 | High |
| 3817 | Akpede–1994 | Pattern and antibiotic susceptibility of bacteria in pyogenic meningitis in a children's emergency room population in Maiduguri, Nigeria, 1988-1992. | 1988—1992 | Case Series | Culture | Bacteria | 8 | High |
| 3818 | Baudon–1984 | Epidemic yellow fever in Upper Volta. | 1983—1983 | Case Series | PCR | Viruses | 1 | High |
| 3823 | Allison–1984 | Pyogenic liver abscess caused by Streptococcus milleri. Case reports. | 1984—1984 | Case Series | Culture | Bacteria | 1 | High |
| 3839 | Almaviva–1993 | Louse-borne relapsing fever epidemic in Arssi Region, Ethiopia: a six months survey. | 1991—1991 | Case Series | Microscopy/Staining | Bacteria | 1 | High |
| 3840 | Andy–1981 | Loasis as a possible trigger of African endomyocardial fibrosis: a case report from Nigeria. | 1977—1977 | Case Series | Microscopy/Staining | Parasites | 1 | High |
| 3841 | Andy–1987 | Cardiovascular complications of tropical pyomyositis. | 1978—1978 | Case Series | Culture | Bacteria | 1 | High |
| 3842 | Atarraf–2014 | [Post infectious upper femoral epiphyseal detachment, report of two cases]. | 2012—2012 | Case Series | Culture | Bacteria | 1 | High |
| 3845 | Berkowitz–1982 | Acinetobacter meningitis - a diagnostic pitfall. A report of 3 cases. | 1980—1981 | Case Series | Culture | Bacteria | 1 | High |
| 3846 | Berkowitz–1987 | Streptococcus bovis bacteraemia associated with candidal lesions of the large bowel in a leukaemic child. A case report. | 1985—1985 | Case Series | Culture | Bacteria | 1 | High |
| 3847 | Borgnolo–1993 | Louse-borne relapsing fever. A clinical and an epidemiological study of 389 patients in Asella Hospital, Ethiopia. | 1991—1992 | Case Series | Microscopy/Staining | Bacteria | 1 | High |
| 3848 | Coovadia–1992 | An outbreak of multiresistant Salmonella typhi in South Africa. | 1991—1991 | Case Series | Culture | Bacteria | 1 | High |
| 3849 | Edoh–2001 | Aspects bacteriologiques des meningites dues a haemophilus influenzae chez l'enfant au CHU de Treichville. | 1996—1999 | Case Series | Culture | Bacteria | 7 | High |
| 3850 | Forman–1981 | Yersinia arthritis mimicking acute rheumatic fever. A case report. | 1980—1980 | Case Series | Serological | Bacteria | 1 | High |
| 3851 | Omanga–1989 | [Bacterial septicemias in children with homozygous sickle cell anemia. Analysis of 69 cases]. | 1964—1985 | Case Series | Culture | Bacteria | 12 | High |
| 3856 | Kokindombo–1991 | [Purulent meningitis due to flavobacterium meningosepticum in Cameroonian children]. | 1988—1989 | Case Series | Culture | Bacteria | 6 | High |
| 3857 | Salih–1990 | Features of a large epidemic of group A meningococcal meningitis in Khartoum, Sudan in 1988. | 1988—1988 | Case Series | Culture | Bacteria | 1 | High |
| 3858 | Green–1993 | Salmonella bacteraemia among young children at a rural hospital in western Zaire. | 1982—1986 | Case Series | Culture | Bacteria | 2 | High |
| 3859 | Mathur–1984 | Pseudomonas septicaemia following tribal tatoo marks. | 1983—1983 | Case Series | Culture | Bacteria | 1 | High |
| 3860 | Swanepoel–1985 | A common-source outbreak of Crimean-Congo haemorrhagic fever on a dairy farm. | 1984—1984 | Case Series | Serological | Viruses | 1 | High |
| 3861 | Coovadia–1987 | Chloramphenicol-resistant Salmonella typhi in Durban, South Africa. | 1985—1985 | Case Series | Culture | Bacteria | 1 | High |
| 3863 | Ryder–1987 | Group B meningococcal infection in children during an epidemic in Cape Town, South Africa. | 1980—1980 | Case Series | Culture | Bacteria | 2 | High |
| 3865 | Laditan–1981 | Problems in the clinical diagnosis of typhoid fever in children in the tropics. | 1978—1978 | Case Series | Culture | Bacteria | 2 | High |
| 3887 | Dare–1989 | Tetanus in pregnancy in a Nigerian woman--a case report. | 1987—1987 | Case Series | Culture | Bacteria | 1 | High |
| 3888 | Wellde–1989 | Presenting features of Rhodesian sleeping sickness patients in the Lambwe Valley, Kenya. | 1980—1984 | Case Series/Fever Series | Culture | Parasites | 1 | High |
| 3890 | Farid–1982 | Ameobic liver abscess presenting as fever of unknown origin (FUO). Serology, isotope scanning and metronidazole therapy in diagnosis and treatment. | 1977—1980 | Case Series | Serological | Parasites | 1 | High |
| 3891 | Mackie–1992 | A study of bacterial meningitis in Kumasi, Ghana. | 1989—1990 | Case Series | Culture | Bacteria | 3 | High |
| 3893 | Borgnolo–1993 | Louse-borne relapsing fever in Ethiopian children: a clinical study. | 1991—1992 | Case Series | Microscopy/Staining | Bacteria | 1 | High |
| 3896 | Shepherd–1985 | A nosocomial outbreak of Crimean-Congo haemorrhagic fever at Tygerberg Hospital. Part V. Virological and serological observations. | 1984—1984 | Case Series | Serological | Viruses | 1 | High |
| 3898 | Saluzzo–1985 | Haemorrhagic fever caused by Crimean Congo haemorrhagic fever virus in Mauritania. | 1983—1983 | Case Series | Serological | Viruses | 1 | High |
| 3900 | Ramos–2004 | Characteristics of louse-borne relapsing fever in Ethiopian children and adults. | 1997—2002 | Case Series | Microscopy/Staining | Bacteria | 1 | High |
| 3901 | Popkiss–1980 | Typhoid fever: A report on a point-source outbreak of 69 cases in Cape Town. | 1978—1978 | Case Series | Culture | Bacteria | 1 | High |
| 3902 | Onwubalili–1989 | Paratyphoid fever presenting with renal failure and nephrotic syndrome. | 1989—1989 | Case Series | Culture | Bacteria | 1 | High |
| 3903 | Okolo–1985 | Changing pattern of neonatal septicaemia in an African city. | 1978—1983 | Case Series | Culture | Bacteria | 16 | High |
| 3909 | Molyneux–2000 | Salmonella meningitis in children in Blantyre, Malawi, 1996-1999. | 1996—1999 | Case Series | Culture | Bacteria | 3 | High |
| 3911 | Moethilalh–1982 | Infective endocarditis in thirteen children: a retrospective study (1974-1981). | 1974—1981 | Case Series | Culture | Bacteria | 9 | High |
| 3914 | Loubser–1993 | Severe illness caused by Rickettsia conorii. | 1993—1993 | Case Series | Serological | Bacteria | 1 | High |
| 3916 | Liman–1996 | Unusual neurologic manifestation of enteric fever--case report and review of the literature. | 1996—1996 | Case Series | Serological | Bacteria | 1 | High |
| 3923 | Haimanot–1990 | Characteristics of serogroup A Neisseria meningitidis responsible for an epidemic in Ethiopia, 1988-89. | 1988—1989 | Fever Series | Culture | Bacteria | 1 | Low |
| 3925 | Gedebou–1987 | The bacteriology of nosocomial infections at Tikur Anbessa Teaching Hospital, Addis Ababa. | 1983—1984 | Case Series | Culture | Bacteria | 4 | High |
| 3926 | Olubodun–1994 | Typhoid fever associated with severe hepatitis. | 1994—1994 | Case Series | Culture | Bacteria | 1 | High |
| 3927 | Boomsma–1988 | Clinical aspects of typhoid fever in two rural Nigerian hospitals. A prospective study. | 1984—1985 | Case Series | Serological | Bacteria | 1 | High |
| 3928 | Davanzo–1992 | Neonatal and post-neonatal onset of early congenital syphilis: a report from Mozambique. | 1989—1990 | Case Series | Serological | Bacteria | 1 | High |
| 3929 | Do Rego–1988 | [Suppurative meningitis in the newborn infant: experience with 107 cases in the Ivory Coast]. | 1985—1986 | Case Series | Culture | Bacteria | 8 | High |
| 3930 | Udonsi–1986 | The status of human filariasis in relation to clinical signs in endemic areas of the Niger Delta. | 1984—1984 | Fever Series | Microscopy/Staining | Parasites | 1 | Low |
| 3931 | Nwadioha–2013 | Bacterial isolates from cerebrospinal fluid of children with suspected acute meningitis in a Nigerian tertiary hospital. | 2006—2009 | Fever Series | Culture | Bacteria | 9 | Low |
| 3932 | Sanusi–2015 | Epidemiology of bacteria colonization and ICU-acquired infection in a Nigerian tertiary hospital | 2011—2012 | Fever Series | Culture | Bacteria | 5 | Low |
| 3933 | Reed–1996 | Bacteraemia in malnourished rural African children. | 1992—1992 | Fever Series | Culture | Bacteria | 13 | Low |
| 3934 | Ojukwu–2006 | Neonatal septicemia in high risk babies in South-Eastern Nigeria. | 2002—2003 | Fever Series | Culture | Bacteria | 9 | Low |
| 3935 | Mahdi–2014 | Extended Spectrum beta-lactamase producing Klebsiella pneumoniae in Neonatal Units of Minya Governorate | 2014—2014 | Fever Series | Culture | Bacteria | 1 | Low |
| 3937 | Koegelenberg–2004 | Infective endocarditis: improving the diagnostic yield. | 1997—2000 | Fever Series | Culture | Bacteria/Fungi | 17 | Low |
| 3938 | Ibidapo–2000 | Acute sickle cell syndromes in Nigerian adults. | 1992—1993 | Fever Series | Culture | Bacteria | 1 | Low |
| 3943 | Bassily–1983 | Acute hepatitis non-A non-B in Cairo residents (a preliminary report). | 1980—1982 | Fever Series | Serological | Viruses | 2 | Moderate |
| 3945 | Akpede–1992 | Indications for lumbar puncture in children presenting with convulsions and fever of acute onset: experience in the Children's Emergency Room of the University of Benin Teaching Hospital, Nigeria. | 1988—1989 | Fever Series | Culture | Bacteria | 4 | Low |
| 3946 | Akinyanju–1987 | Acute illness in Nigerian children with sickle cell anaemia. | 1986—1986 | Fever Series | Not described | Bacteria | 6 | Unclear |
| 3947 | Adegoke–2013 | Rare bacterial meningitis among hospitalized children in a teaching Hospital | 2011—2011 | Fever Series | Culture | Bacteria | 2 | Low |
| 4003 | Johnson–1983 | Viral haemorrhagic fever surveillance in Kenya, 1980-1981. | 1980—1981 | Seroprevalance study | Serological | Viruses | 1 | Moderate |
| 4004 | Botros–1989 | Seroprevalence of murine typhus and fiÃ¨vre boutonneuse in certain human populations in Egypt. | 1984—1987 | Seroprevalance study | Serological | Bacteria | 2 | Moderate |
| 4005 | Van der Waals–1986 | Hemorrhagic fever virus infections in an isolated rainforest area of central Liberia. Limitations of the indirect immunofluorescence slide test for antibody screening in Africa. | 1981—1982 | Seroprevalance study | Serological | Viruses | 5 | Moderate |
| 4006 | Tignor–1993 | The yellow fever epidemic in Ethiopia, 1961-1962: retrospective serological evidence for concomitant Ebola or Ebola-like virus infection. | 1961—1962 | Seroprevalance study | Serological | Viruses | 1 | Moderate |
| 4007 | Monath–1987 | Type B hepatitis and yellow fever infections in West Africa. | 1979—1985 | Seroprevalance study | Serological | Viruses | 1 | Moderate |
| 4008 | Rey–1996 | [Fever of unknown origin (FUO) in the camps of Rwandan refugees in the Goma region of in Zaire (September 1994)]. | 1994—1994 | Seroprevalance study | Serological | Bacteria/Viruses | 5 | Moderate |
| 4009 | Ndumbe–1993 | Hepatitis C virus infection in different populations in Cameroon. | 1990—1992 | Seroprevalance study | Serological | Viruses | 2 | Moderate |
| 4012 | Nasidi–1993 | Urban yellow fever epidemic in western Nigeria, 1987. | 1987—1987 | Case Series | Serological | Viruses | 1 | High |
| 4013 | Morvan–1991 | Possible Rift Valley fever outbreak in man and cattle in Madagascar. | 1990—1990 | Seroprevalance study | Serological | Viruses | 1 | Moderate |
| 4014 | Morrill–1991 | Serological evidence of arboviral infections among humans of coastal Kenya. | 1987—1987 | Seroprevalance study | Serological | Viruses | 8 | Moderate |
| 4015 | Meunier–1988 | The 1987 yellow fever epidemic in Mali: viral and immunological diagnosis. | 1987—1987 | Seroprevalance study | Serological | Viruses | 1 | Moderate |
| 4016 | Baba–2001 | Serological evidence of brucellosis among predisposed patients with pyrexia of unknown origin in the north eastern Nigeria. | 2000—2000 | Seroprevalance study | Microscopy/Staining | Bacteria | 1 | Moderate |
| 4024 | Ndumbe–1991 | Hepatitis D in Yaounde, Cameroon. | 1990—1990 | Seroprevalance study | Serological | Viruses | 2 | Moderate |
| 4025 | Blackburn–1987 | The status of dengue fever virus in South Africa--serological studies and diagnosis of a case of dengue fever. | 1985—1985 | Seroprevalance study | Serological | Viruses | 3 | Moderate |
| 4026 | Arthur–1993 | Recurrence of Rift Valley fever in Egypt. | 1993—1993 | Seroprevalance study | Serological | Viruses | 1 | Moderate |
| 4028 | Chantal–1996 | [Serologic screening of certain zoonoses in the abattoir personnel in Djibouti]. | 1992—1992 | Seroprevalance study | Serological | Bacteria/viruses/parasites | 7 | Moderate |
| 4030 | Thonnon–1994 | [Evaluation of the immunological and entomological indices of yellow fever in the subprefecture of TaÃ¯, Ivory Coast]. | 1993—1993 | Seroprevalance study | Serological | Viruses | 1 | Moderate |
| 4032 | Letaief–1995 | Seroepidemiological survey of rickettsial infections among blood donors in central Tunisia. | 1993—1993 | Seroprevalance study | Serological | Bacteria | 3 | Moderate |
| 4037 | Botros–1995 | Coxiella burnetii antibody prevalences among human populations in north-east Africa determined by enzyme immunoassay. | 1987—1988 | Seroprevalance study | Serological | Bacteria | 1 | Moderate |
| 4038 | Wessels–1986 | Q-fever, OX19, OX2 and leptospirosis antibodies in patients with onyalai and in negroid, bushman and white inhabitants of Kavango, Namibia. | 1985—1985 | Seroprevalance study | Serological | Bacteria | 2 | Moderate |
| 4039 | Tomori–1986 | Antibody to Japanese strain of haemorrhagic fever with renal syndrome (HFRS) virus in Nigerian sera. | 1986—1986 | Seroprevalance study | Serological | Viruses | 1 | Moderate |
| 4040 | Tessier–1987 | Viral haemorrhagic fever survey in Chobe (Botswana). | 1985—1985 | Seroprevalance study | Serological | Viruses | 2 | Moderate |
| 4041 | Redus–1986 | Prevalence and distribution of spotted fever and typhus infections in Sierra Leone and Ivory Coast. | 1981—1981 | Seroprevalance study | Serological | Bacteria | 2 | Moderate |
| 4042 | Saleh–1981 | Antibodies to Rift Valley fever virus in the human population of Sudan. | 1981—1981 | Seroprevalance study | Serological | Viruses | 1 | Moderate |
| 4043 | Blackburn–1982 | Viral haemorrhagic fever antibodies in Zimbabwe schoolchildren. | 1980—1980 | Seroprevalance study | Serological | Viruses | 5 | Moderate |
| 4169 | Schwarz–2010 | Systemic bacteraemia in children presenting with clinical pneumonia and the impact of non-typhoid salmonella (NTS). | 2007—2009 | Fever Series | Culture | Bacteria | 13 | Low |
| 4170 | Almaviva–1993 | Louse-borne relapsing fever epidemic in Arssi Region, Ethiopia: a six months survey. | 1991—1991 | Case Series | Microscopy/Staining | Bacteria | 1 | High |
| 4171 | Barclay–1990 | Tick-borne relapsing fever in central Tanzania. | 1985—1986 | Case Series | Microscopy/Staining | Bacteria | 1 | High |
| 4172 | Onile–1990 | Neonatal septicemia resulting from group B streptococci: a case report. | 1979—1979 | Case Series | Culture | Bacteria | 1 | High |
| 4173 | Hashim–1994 | An outbreak of acute kala-azar in a nomadic tribe in western Sudan: features of the disease in a previously non-immune population. | 1990—1992 | Case Series | Culture | Parasites | 1 | High |
| 4174 | Morvan–1992 | First fatal human case of Rift Valley fever in Madagascar. | 1991—1991 | Case Series | Serological | Viruses | 1 | High |
| 4176 | Mikhail–1989 | Antibiotic-multiresistant Salmonella typhi in Egypt. | 1988—1988 | Case Series | Not described | Bacteria | 1 | High |
| 4177 | de Jong–1995 | Louse-borne relapsing fever in southern Sudan. | 1993—1993 | Case Series | Microscopy/Staining | Bacteria | 1 | High |

## **Section 2: Methodology for the assessment of risk of reporting bias in included studies toward urban settings**

In order to assess if there is a geographical bias towards reporting of data from urban settings or areas with easy access, we looked at the distance between the study location and the nearest city or the capital city of the country where the study was conducted.

The nearest major city to a given study site was defined as a place with a population greater than 100,000 as obtained from the “maps” package. The coordinates of the cities in Africa were obtained from the “maps” package.

The distance between the study location and the nearest major city was calculated using the Havernsine formula assuming the radius of earth to be 6,371 meters using the following function:

# R script for computing distance between latitude and longitude

haversine <- function( lat1, lon1, lat2, lon2, radius = 6371 ) {

# Convert decimal degrees to radians

lon1 = lon1 * pi / 180

lon2 = lon2 * pi / 180

lat1 = lat1 * pi / 180

lat2 = lat2 * pi / 180

# Haversine formula

dlon = lon2 - lon1

dlat = lat2 - lat1

a = sin(dlat/2)**2 + cos(lat1) * cos(lat2) * sin(dlon/2)**2

c = 2 * atan2(sqrt(a), sqrt(1-a))

return( radius * c * 1000 )

}

The assessment of risk of bias in reporting of results towards urban settings is presented in Table 2.

### **2.1 Assessment of geographical bias toward reporting of data from urban settings in studies included in the systematic review**


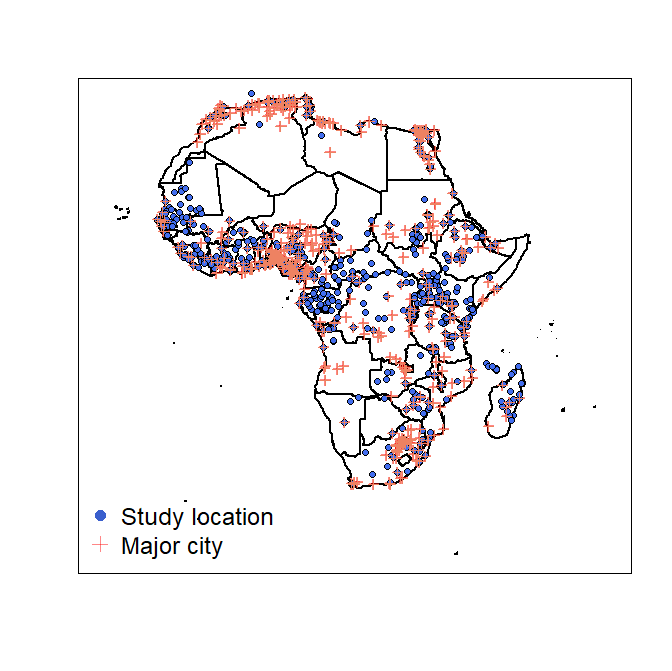


**Figure 1: Location of study sites, a systematic review of published aetiological studies and case reports from Africa, 1980-2015**

Legend: The blue dots indicate the study sites where the studies included in the review were conducted. The red dots depicts the location of major cities in Africa with population greater than 1000,000. The co-ordinates of the major cities were obtained from “*maps*” package in R software.


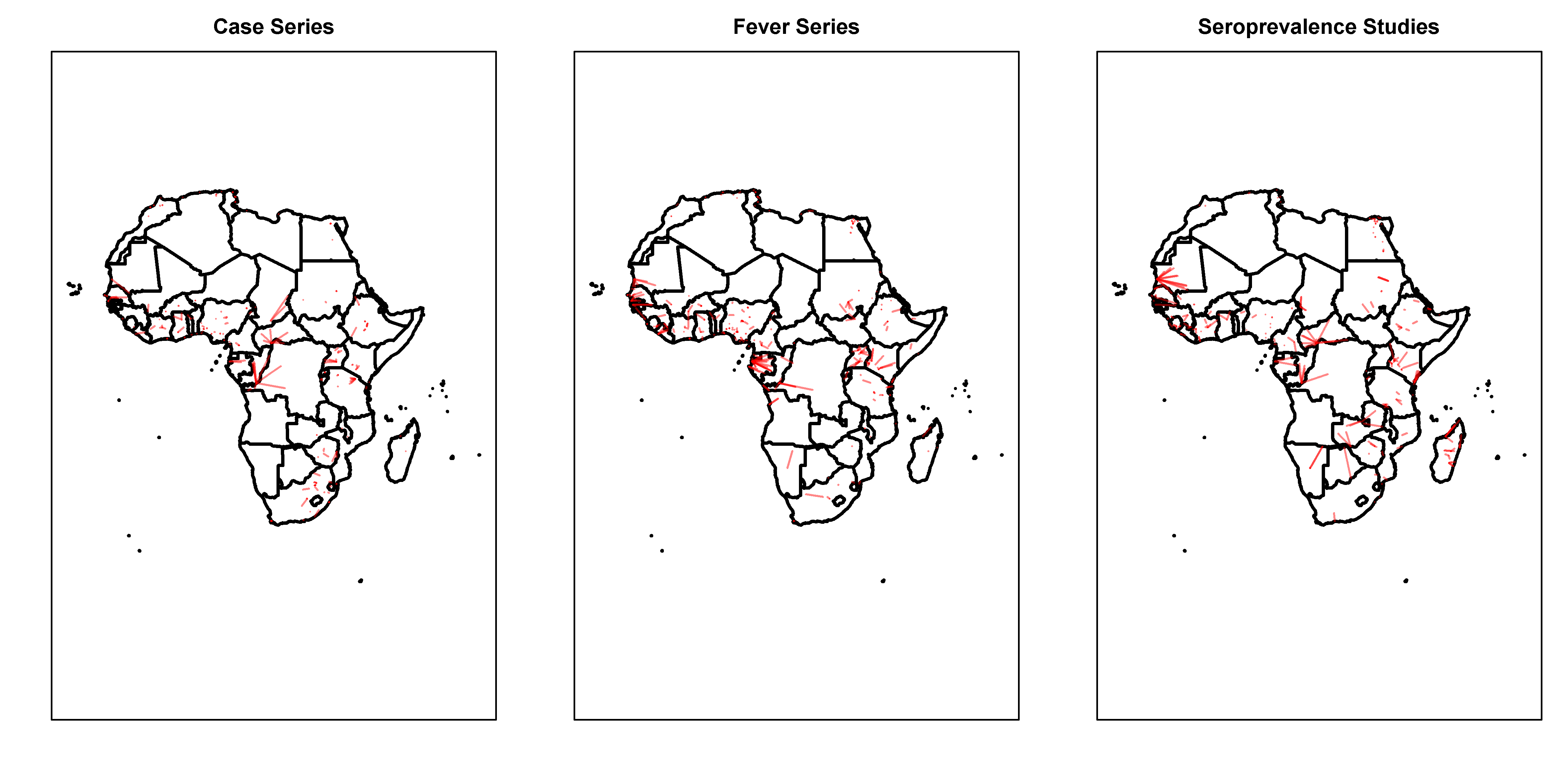


**Figure 2: Distance of the study sites from the nearest city, a systematic review of published aetiological studies and case reports from Africa, 1980-2015**

**Legend**: Nearest city was defined as a place with population greater than 100,000. The co-ordinates of cities were obtained from “*maps*” package in R software. The distance between the study location and the nearest city was calculated using Haversine formula assuming the radius of earth to be 6,371 meters. Case series included individual case reports or series of patients with the same condition. Studies were classed as fever series if the total population denominator tested was reported. Seroprevalence studies were those where serum samples were tested for one pathogen or a panel of pathogens simultaneously.
